# Cysteine‐Assisted Click‐Chemistry for Proximity‐Driven, Site‐Specific Acetylation of Histones

**DOI:** 10.1002/anie.202208543

**Published:** 2022-10-18

**Authors:** Cláudia F. Afonso, Marta C. Marques, João P. M. António, Carlos Cordeiro, Pedro M. P. Gois, Pedro M. S. D. Cal, Gonçalo J. L. Bernardes

**Affiliations:** ^1^ Instituto de Medicina Molecular João Lobo Antunes Faculdade de Medicina Universidade de Lisboa Avenida Professor Egas Moniz 1649-028 Lisboa Portugal; ^2^ Research Institute for Medicines (iMed.ULisboa) Faculdade de Farmácia Universidade de Lisboa Av. Prof. Gama Pinto 1649-003 Lisboa Portugal; ^3^ Laboratório de FT-ICR e Espectrometria de Massa Estrutural Faculdade de Ciências Universidade de Lisboa Campo Grande 1749-016 Lisboa Portugal; ^4^ Yusuf Hamied Department of Chemistry University of Cambridge Lensfield Road CB2 1EW Cambridge UK

**Keywords:** Click Chemistry, Cysteine Bioconjugation, Histones, Lysine Acetylation, Maleimide

## Abstract

Post‐translational modifications of histones are essential in the regulation of chromatin structure and function. Among these modifications, lysine acetylation is one of the most established. Earlier studies relied on the use of chromatin containing heterogeneous mixtures of histones acetylated at multiple sites. Differentiating the individual contribution of single acetylation events towards chromatin regulation is thus of great relevance. However, it is difficult to access homogeneous samples of histones, with a single acetylation, in sufficient quantities for such studies. By engineering histone H3 with a cysteine in proximity of the lysine of interest, we demonstrate that conjugation with maleimide‐DBCO followed by a strain‐promoted alkyne‐azide cycloaddition reaction results in the acetylation of a single lysine in a controlled, site‐specific manner. The chemical precision offered by our click‐to‐acetylate approach will facilitate access to and the study of acetylated histones.

## Graphical abstract

We present a proximity‐driven, lysine‐specific acetylation approach to construct precisely acetylated histone proteins. The approach is facilitated by a click reagent equipped with an acetate group donor to acetylate a nearby lysine in a sequence‐controlled manner.

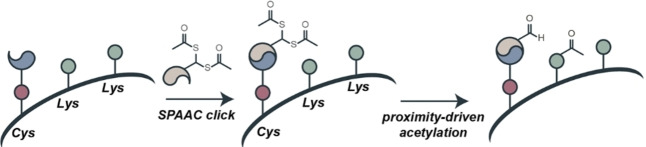

Histones are proteins that organize the negatively charged DNA into the repeating cored structures, termed nucleosomes, that form chromatin.^[1]^ The nucleosome structure is stabilized by electrostatic interactions, however histone PTMs, particularly at their flexible *N*‐terminal tail, constitute an essential epigenetic mechanism that regulates the accessibility of DNA to replication, transcription and repair machineries. Examples of these PTMs include acetylation, methylation and phosphorylation and the reversible nature of these modifications allows modulation of chromatin structure by directly altering histone interactions with DNA or by recruiting chromatin‐associated proteins.^[2]^

Acetylation is a common PTM that occurs at the *ϵ*‐amine side chain of lysine (Lys or K) residues in histones.^[3]^ The transfer of an acetyl group neutralizes the positive charge of lysine residues, which decreases the interaction of histones with DNA. As a result, the condensed chromatin is transformed into a more relaxed structure that facilitates accessibility of transcription factors to DNA and is associated with greater levels of gene expression. The acetylation of lysine 9 in histone H3 (H3K9Ac) is a particularly relevant PTM, because it is commonly found at the promoter regions of active genes and can induce transcription by promoting transcription factor TFIID binding.^[4]^

Although many acetylation marks have been characterized in histones, a biochemical understanding of how these modifications combine to regulate chromatin function is essential. However, access to homogeneous samples of histones containing a single acetyl group in the quantities required for these studies is difficult to achieve from biological sources or enzymatic methods.^[5]^ Several approaches have been developed to achieve site‐specific acetylation of histones, namely protein ligation, genetic code expansion, cysteine‐selective modification and the use of affinity ligands tethered to acyl transfer catalysts.^[6]^ Our technology seeks to overcome many of the disadvantages presented by these methods, namely the technically challenging nature of protein ligation methods;^[7]^ the lower yield of modified histones with genetic code expansion;^[8]^ mutagenesis of the amino acids of interest to generate acetyl‐lysine mimics, which may lead to sub‐optimal interactions with their natural binding partners in the case of cysteine conjugation methods;^[9]^ and the dependency on the existence of corresponding interacting ligands that can promote acetylation of particular lysines in the protein sequence.^[10]^

Herein, we present a novel strategy that couples cysteine conjugation with click‐chemistry for site‐specific acetylation of a single proximate lysine residue on histones. We introduce an artificial “acetyltransferase‐like” histone that is chemically and spatially controlled by the addition of a click handle counterpart (Scheme 1).

**Scheme 1:**
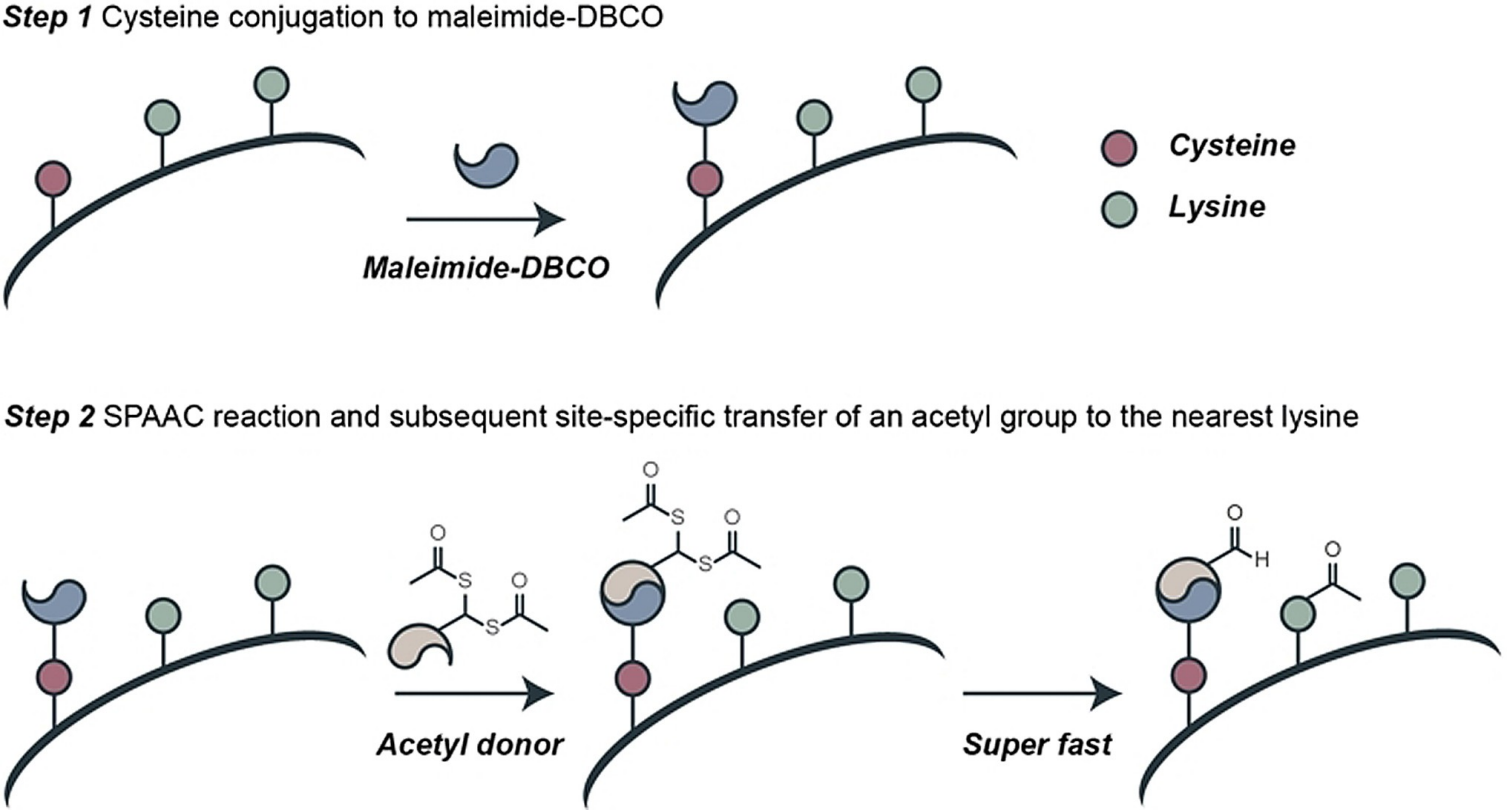
Overview of the site‐specific acetylation strategy presented in this work.

Considering the biological significance that acetylation of lysine 9 in histone H3 has, we decided to use it as a target to test our proximity‐driven acetylation strategy. For this purpose, the initial sequence of 15 amino acids of histone H3 was synthesized, in which single cysteine residues replaced both surrounding lysines of K9 to generate two different peptides—peptides K4C and K14C (Figures S1, S2)—which enables the installation of the clickable handles. Two chemical reagents were sought at this stage, the first a hetero‐crosslinker with a cysteine‐modifying handle (maleimide) and a strain‐promoted alkyne‐azide cycloaddition (SPAAC) clickable handle (cyclooctyne) that can connect the peptide to the second, a lysine modifying reagent. The lysine modifying reagent has a click counterpart for the SPAAC reaction (azide) and a stable acetyl donor (*gem*‐dithioacetate) with a fixed spacer. The first reagent is commercially available (maleimide‐DBCO) and the second was synthesized (Scheme 2).

**Scheme 2:**
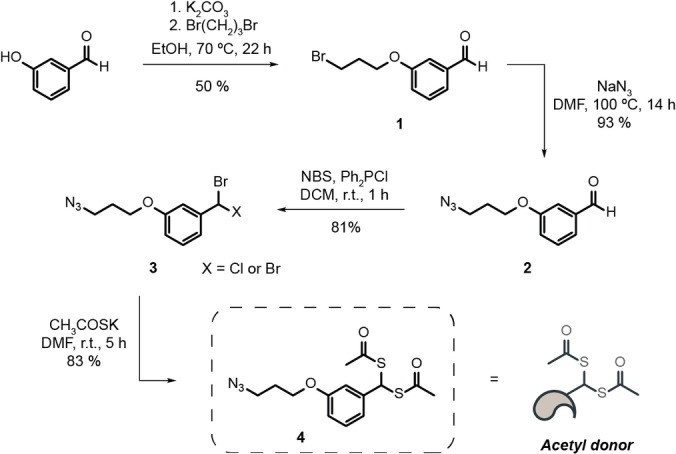
Synthesis of the acetyl donor that has an azide SPAAC counterpart and a *gem*‐dithioacetate. DMF=dimethyl formamide. NBS=*N*‐bromosuccinimide.

We started with an alkylation reaction between 3‐hydroxybenzaldehyde and a dibromoalkyl reagent, followed by a substitution of the remaining bromine by sodium azide. From the azide‐aldehyde bearing compound, we performed a bromination reaction of the aldehyde function, which was subsequently reacted with potassium thioacetate to afford **4** with an overall yield of 31 %. Smaller‐sized dibromoalkyl reagents were also trialled but did not afford the intended product. To assess if the synthesized chemical handle was stable for the intended purpose, that is a SPAAC with a cysteine derivative bearing a click handle, a doubly protected S‐propargyl‐cysteine derivative was used as a model. This reaction confirmed that the *gem*‐dithioacetate is stable under the conjugation reaction conditions, which demonstrates the viability of such a molecule. As reported,^[11]^ *gem*‐dithioacetate **4** by itself acetylates free cysteines in peptides under the same conditions (Figures S3, S4).

Next, we moved on to test our proposed strategy in H3 tail peptides. Maleimide‐DBCO was used to perform the initial cysteine bioconjugation method, which installed a clickable handle within five residues of the target K9 for acetylation. After optimization, peptides K4C and K14C reacted under mild conditions with a slight excess of maleimide‐DBCO (2 equiv, NH_4_^+^CH_3_CO_2_^−^ 20 mM, pH 8.0, 2 h at 25 °C, Figures S5, S6). With these modified peptides, the directed acetylation of K9 was attempted. Both peptides reacted with **4** to afford the click product from the SPAAC and acetylation of the nearest nucleophilic residue, in this case K9. The reaction conditions were optimized to give the expected product with complete conversion (8 equiv of **4**, NH_4_^+^CH_3_CO_2_^−^ 20 mM, pH 8.0, 1 h at 25 °C; Figures 1 and S7, S8). To ensure their stability, the acetylated peptides were evaluated after 1 month and no discernible degradation was seen (Figures S9, S10). To confirm that acetylation occurs in the nearest nucleophilic residue K9, we used high‐resolution FT‐ICR mass spectrometry and MS/MS fragmentation experiments to map the resulting peptides. For both peptides acetylation always occurred in the lysine closest to the modified cysteine (Figure 1 and Tables S1, S2). As a control, mono‐acetylated peptides were reacted with iodoacetamide to confirm that reaction with the maleimide had taken place exclusively at the cysteine. As expected, no changes were noted (Figures S11–S14). All the above‐mentioned experiments were performed with peptides K4C and K14C. Furthermore, by using a model peptide containing several reactive amino acids together with a cysteine residue, but without lysines, we demonstrated that under the same reaction conditions acetylation did not occur and that the resulting SPAAC product was stable to hydrolysis (Figures S15–S17).

**Figure 1:**
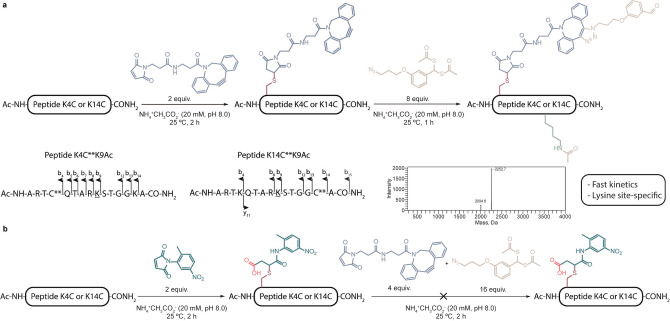
Strategy for acetylating histone H3 peptides. a) Sequential reaction of histone H3K4C and K14C peptides with maleimide‐DBCO and **4** results in site‐specific acetylation of the nearest lysine residue as confirmed by MS/MS analysis. b) Sequential reaction of histone H3K4C and K14C peptides with a maleimide reagent without an alkyne (maleimide‐dummy) and the click product formed between maleimide‐DBCO and **4** did not promote acetylation.

To establish that the SPAAC reaction is essential for orienting the acetylation reaction towards the nearby K9 residue, we conjugated both peptides with a maleimide‐dummy under the same reaction conditions as those described above and added either **4** by itself or the product resulting from the SPAAC between this compound and maleimide‐DBCO. As expected, acetylation was not detected in either of the K4C and K14C peptides (Figures 1 and S18–S21).

Given that the conjugation reactions and their respective controls in peptides supported our claims, we performed two additional assays to confer a greater biological significance to our strategy. First, we evaluated the possibility of removing the side product of the SPAAC reaction still attached to the cysteine residue by using thiol‐exchange via a retro‐Michael addition. β‐mercaptoethanol was used as the thiol compound and although somewhat successful, irreversible hydrolysis of the maleimide occurred during the process, which resulted in a significant amount (20–30 %) of the side product unaltered. In addition, hydrolysis of the acetylated K9 following the retro‐Michael addition was also observed (20 %), which affords the unmodified K4C and K14C peptides (Figures S22–S25). Together, these experiments demonstrate the feasibility of the cysteine‐assisted click‐chemistry for lysine acetylation as well as proof‐of‐concept for its potential for proximity‐driven residue‐specific acetylation on peptides.

Next, we assessed the ability of the acetylated peptides to be recognized as substrates by one of the NAD^+^‐dependent histone deacetylase enzymes. Under conditions suitable for deacetylation to occur, modified K4C and K14C peptides were incubated with Sirt6, which can remove the acetyl group of specific lysine residues of histone H3, namely K9 and K56.^[12]^ Although deacetylation was incomplete, a significant difference between the results of both peptides was observed for the first time. For the modified K4C peptide, conversion towards the deacetylated product was ≈40 % (Figure S26), whereas for the modified K14C peptide it was <20 % (Figure S27). These results serve as preliminary data to demonstrate the ability of the modified peptides to be recognized by histone deacetylases, even in the presence of the SPAAC side product at the nearby cysteine.

The same set of bioconjugation reactions as those previously described for the K4C and K14C peptides were performed for the full‐length protein, which was recombinantly expressed with a single cysteine mutant H3K4C (C110A) (Figure S28).[^9a^, ^9b^] Treatment of H3K4C with an excess of maleimide‐DBCO resulted in complete conversion of the protein (2.5 equiv, NH_4_^+^CH_3_CO_2_^−^ 20 mM, pH 7.0, 1 h at 25 °C; Figures 2 and S34). No reaction with Ellman's reagent confirmed that conjugation occurred at the free cysteine of H3K4C (10 equiv, NH_4_^+^CH_3_CO_2_^−^ 20 mM, pH 8.0, 30 min at 25 °C; Figure S62). After purification, site‐specific directed acetylation was attempted by treating the modified protein with **4**. Incubating the H3K4C‐maleimide‐DBCO protein with a 4‐equiv excess of the reagent yielded the respective mono‐acetylated product, H3K4C**K9Ac (NH_4_^+^CH_3_CO_2_^−^ 20 mM, 30 min at 25 °C; Figures 2, S37 and S43,S44). Analysis of the circular dichroism (CD) profiles of the three proteins (H3K4C, H3K4C** and H3K4C**K9Ac) showed that the overall secondary structure of H3K4C did not change following cysteine conjugation and subsequent SPAAC reaction (Figure S71). The site‐specificity of our approach was confirmed by high‐resolution FT‐ICR mass spectrometry and MS/MS fragmentation experiments which showed acetylation only at residue K9 (Figure S75).

**Figure 2:**
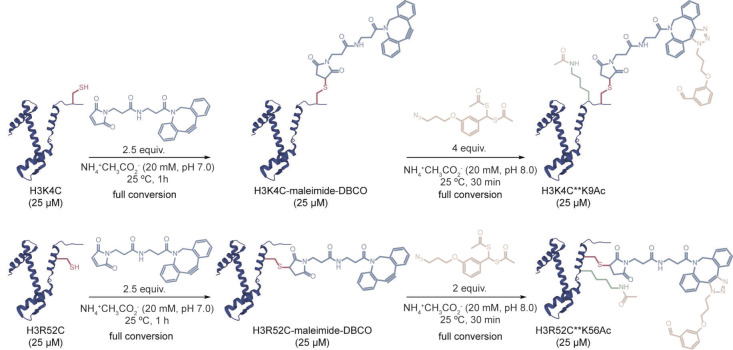
Strategy for acetylating histone H3 proteins. The sequential reaction of histones H3K4C and H3R52C with maleimide‐DBCO and **4** results in the mono‐acetylation of these proteins.

We then questioned if this strategy could be generalized to other lysine residues for which the biological role of acetylation is also important or unknown. To test this possibility, we chose the more internal residue K56 of histone H3 as the second target of our directed acetylation strategy. H3K56 acetylation (H3K56Ac) is a conserved epigenetic mark found in yeast and higher eukaryotes that plays a critical role in genomic stability by promoting efficient nucleosome assembly following DNA replication.^[13]^ Consequently, the single cysteine mutant H3R52C (C110A) was generated, recombinantly expressed and purified (Figure S29). The first conjugation reaction of H3R52C with maleimide‐DBCO was attempted using the same conditions as those described previously for the H3K4C protein, with complete conversion observed with 2.5 equiv of the reagent (Figures 2 and S35). The modified protein did not react with Ellman's reagent, which confirmed that conjugation of maleimide‐DBCO occurred at the free cysteine of H3R52C (10 equiv, NH_4_^+^CH_3_CO_2_^−^ 20 mM, pH 8.0, 30 min at 25 °C, Figure S64). Following size‐exclusion purification of the modified protein, treatment with 2 equiv of **4** yielded the corresponding mono‐acetylated product, H3R52C**K56Ac (NH_4_^+^CH_3_CO_2_^−^ 20 mM, 30 min at 25 °C; Figures 2, S38 and S45). Thus, attesting greater value to the strategy, not only easily accessible residues could be modified but also more hindered and constrained residues within the protein sequence. Furthermore, to establish that the SPAAC reaction is essential to direct the acetyl group towards the nearest lysine and thus for acetylation to occur in proteins, H3K4C and H3R52C were conjugated to the same maleimide‐dummy as the one described previously for the corresponding peptide experiments (Figures S50, S51). Following purification of the modified proteins, these were treated with **4** under the same conditions as those used for the SPAAC reactions. Similarly to K4C and K14C peptides, acetylation was not detected in either of the two proteins (Figures S53, S54, S56, S57).

We then checked whether the chemically acetylated H3K4C**K9Ac and H3R52C**K56Ac proteins are recognized as antigens by their cognate antibodies. Western blot and ELISA experiments showed that antibodies raised against the natural ϵ‐acetyl‐lysine at positions 9 and 56 of H3 bind to H3K4C**K9Ac and H3R52C**K56Ac, respectively, and that the presence of the SPAAC product at the nearby cysteine residue is not detrimental to this process (Figures 3, S76–S79 and S84). Importantly, western blot using the anti‐H3K9Ac antibody did not show a signal for the H3R52C**K56Ac protein (Figure S80). When a dual site‐specific H3K4C**K9AcR52C**K56Ac was produced (Figures S39 and S46), a blot signal was obtained for both acetylated lysines at positions 9 and 56 (Figures S81–S83). Together this data suggests that our cysteine‐assisted click‐chemistry approach allows for site‐specific installation of one or two acetyl groups at pre‐determined lysines in H3.

**Figure 3:**
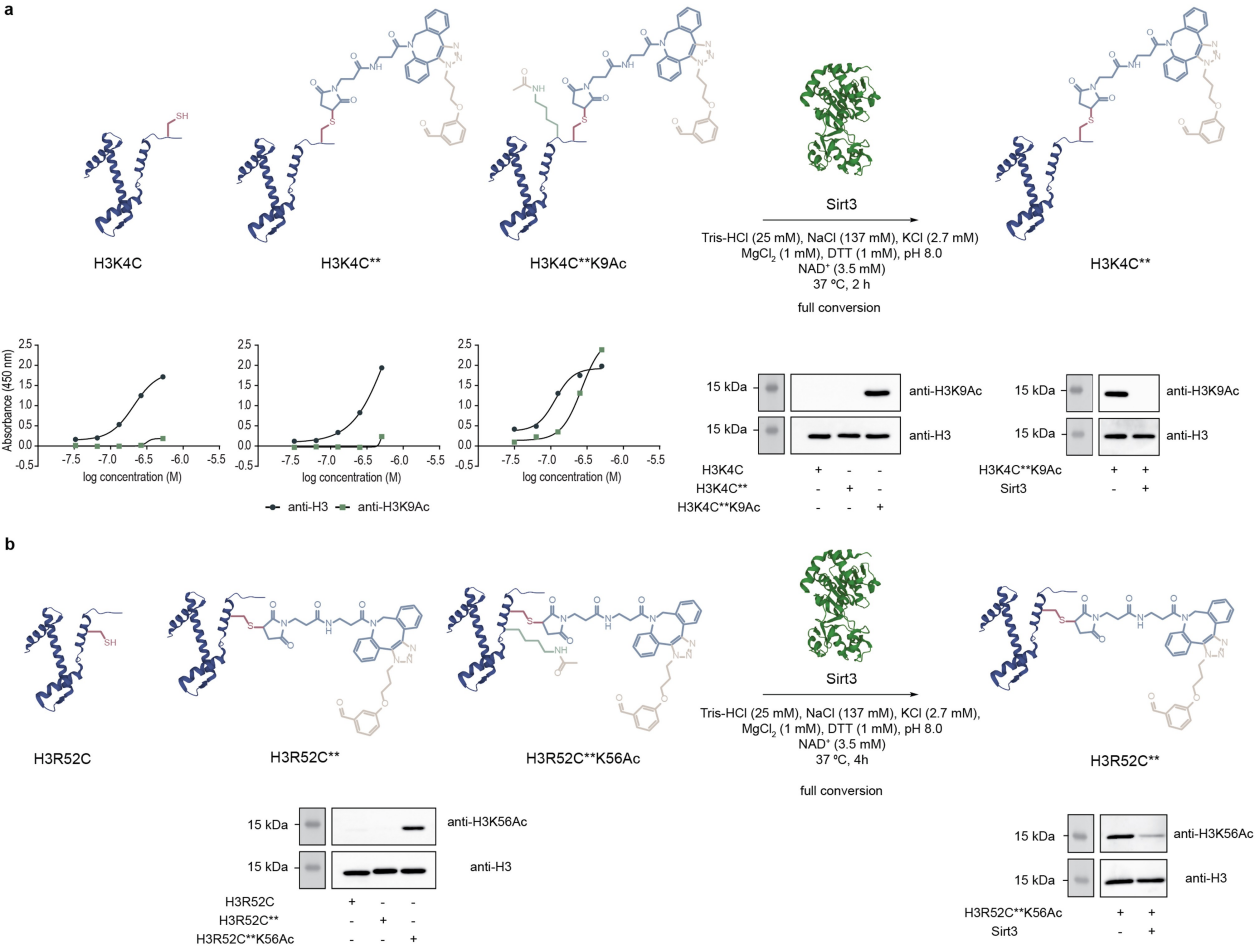
Western Blot and ELISA experiments confirmed the site‐specificity of our approach for acetylating a) position K9 and b) position K56 of histone H3. Deacetylation experiments with Sirt3 demonstrated that the chemically installed acetyl group in these proteins can be removed even in the presence of the SPAAC product at the nearby cysteine. The Western Blot images displayed here are duplicates of the full‐size images reported in the Supporting Information.

Finally, we wondered whether our acetylated H3K4C**K9Ac and H3R52C**K56Ac proteins can be used in the study of histone‐modifying enzymes. Both proteins were incubated with Sirt3, one of the NAD^+^‐dependent histone deacetylase enzymes that exhibits a high preference for removing acetyl groups of many lysine residues of histone H3.^[14]^ After incubating H3K4C**K9Ac with Sirt3 for 2 h at 37 °C, full removal of the acetyl group was observed as indicated by western blot and ELISA experiments (Figures 3 and S85, S86). Significant deacetylation of H3R52C**K56Ac was also seen following incubation with Sirt3 for 4 h at 37 °C (Figures 3 and S87). Collectively, this data shows that our method is useful to create functional H3 proteins with acetyl group at precise sites.

In summary, we describe an approach which combines cysteine conjugation with click chemistry for the oriented site‐specific acetylation of pre‐defined lysine residues. Our strategy comprises two sequential steps: a Michael addition reaction between the unique cysteine of the protein and the maleimide moiety of the heterocrosslinker reagent, followed by a SPAAC reaction between the DBCO clickable handle of the latter and an azide bearing an acetyl donor. Here, the SPAAC locks the acetyl donor in proximity to the lysine of interest. Spontaneous acetylation is then triggered by nucleophilic attack of the side‐chain amine of the nearest lysine on the carbonyl of the acetyl donor. By using this approach, we modified recombinant histones at the more external K9 residue or at the more internal K56 residue of histone H3. H3K4C and H3R52C mutants were mono‐acetylated (and H3K4CR52C di‐acetylated), without unspecific transfer of the acetyl group to other, more distant nucleophilic lysines nor by resorting to any complex biological or purification techniques. These acetylated histones interact with their relevant biological partners, even in the presence of the SPAAC product at the nearby cysteine. Removal of this by‐product may be achieved using known procedures such as Pd^II^‐promoted cleavage of the thiosuccinimide linkage^[15]^ or thiol exchange via retro‐Michael addition.^[16]^ The nucleophilicity of the resulting free cysteine could be blocked through alkylation or conversion to a non‐nucleophilic alanine using known desulfurization procedures.^[17]^ The strategy reported here can be used to probe the role of acetylation events at precise sites, which may help decipher their contribution towards a given biological function.

## Conflict of interest

The authors declare no conflict of interest.

## Supporting information

As a service to our authors and readers, this journal provides supporting information supplied by the authors. Such materials are peer reviewed and may be re‐organized for online delivery, but are not copy‐edited or typeset. Technical support issues arising from supporting information (other than missing files) should be addressed to the authors.

Supporting Information

## Data Availability

The data that support the findings of this study are available in the Supporting Information of this article.
