# Supplementary material for: Cysteine‐Assisted Click‐Chemistry for Proximity‐Driven, Site‐Specific Acetylation of Histones
Source: Angew Chem Int Ed Engl. 2022 Oct 18;61(46):e202208543. doi: 10.1002/anie.202208543 (PMC9828500; doi:10.1002/anie.202208543)
Supplement: Supplementary file 1 — Supporting Information [file ANIE-61-0-s001.pdf]

Angewandte Chemie
Eine Zeitschrift der Gesellschaft Deutscher Chemiker

Supporting Information

Cysteine-Assisted Click-Chemistry for Proximity-Driven, Site-Specific Acetylation of Histones

*C. F. Afonso, M. C. Marques, J. P. M. António, C. Cordeiro, P. M. P. Gois, P. M. S. D. Cal, G. J. L. Bernardes**

Table of Contents

1. General Experimental Information 3
2. Experimental Procedures for Chemical Synthesis 4
3. Conjugation experiments in histone peptides 6
4. Expression and purification of mutant histone proteins 35
5. Conjugation experiments in histone proteins 36
6. Circular dichroism (CD) spectroscopy 91
7. Confirmation of site-specific acetylation in the H3K4C**K9Ac protein by MS/MS analysis 93
8. Western blot analysis of K9 and K56 acetylation 96
9. Development of an ELISA protocol for the detection and quantitation of K9 acetylation 103
10. Deacetylation assays of the H3K4C**K9Ac and H3R52C**K56Ac proteins with Sirt3 104
11. NMR spectra 108
12. References 112

1. General Experimental Information

All chemical reagents and solvents were purchased from commercial sources and used without further purification. The protected histone H3 peptides K4C (ARTCQTARKSTGGKA) and K14C (ARTKQTARKSTGGCA) with *N*-terminal acetylation and *C*-terminal amidation were purchased from Biomatik, reconstituted as advised and used without any further purification steps. The ESBP-NoK peptide (GCDRDYTWFELWDMMQ) was purchased with a 5-carboxyfluorescein (5-FAM) fluorescent dye at the *N-terminus* and an amide at the *C-terminus* from Biomatik. This peptide was reconstituted as advised and purification was attempted with Pur-A-Lyzer dialysis units (1 kDa) from Sigma.

Proton (^{1}H) and carbon (^{13}C) nuclear magnetic resonance (NMR) spectra were taken on a Bruker Fourier 300 MHz Spectrometer in the stated solvents. When possible, NMR spectra were assigned according to the reported characterization in the literature. Chemical shifts are reported on the δ scale in parts per million (ppm) and the spectra were calibrated using the residual solvent peaks as internal standards (^{1}H-NMR: $CDCl_3$ δ 7.26 ppm; ^{13}C-NMR: $CDCl_3$ δ 77.16 ppm). Multiplicities are described as s (*singlet*), d (*doublet*), t (*triplet*), q (*quartet*), quint (*quintuplet*), m (*multiplet*), dd (*doublet of doublets*), dt (*doublet of triplets*), tdd (*triplet of doublet of doublets*) and ddd (*doublet of doublet of doublets*). Coupling constants (*J*) are reported in hertz (Hz) to 1 decimal place using MestreNova for signal processing. The centre of each peak is reported except for multiplet signals where a range of ppm values is given. Structural identification was made with the aid of COSY, HMQC and HMBC experiments.

High-resolution mass spectrometry (HRMS) of the synthesized organic compounds was performed by Unidade de Espectrometria de Masas e Proteómica, Universidade de Santiago de Compostela using a micrOTOF spectrometer and equipped with an electrospray ion (ESI) source.

Low-resolution mass spectra (LRMS) for the peptide experiments was recorded on an ion trap mass analyser (Thermo Scientific LCQ Fleet Ion Trap LC-MS) equipped with an electrospray interface operating in positive mode.

Liquid chromatography-mass spectrometry (LC-MS) for the protein experiments was performed on a Waters QDa mass spectrometer coupled to a Waters UPLC using a Waters XBridge Protein BEH C4 column (300 Å, 3.5 µm, 2.1 mm x 100 mm) operated at 40 ºC. Water (solvent A) and acetonitrile (solvent B) containing 0.1% and 0.01% formic acid by volume, respectively, were used as the mobile phases at a flow rate of 0.2 mL/min. The gradient elution was programmed as follows: 90% A for 5 min and then a linear gradient to 100% for 15 min. A gradient of 5 min back to 90% A was used to re-equilibrate the column. The ESI source was operated in positive mode with a capillary voltage of 1.5 kV and a cone voltage of 20 V. Nitrogen was used as the nebulizer and desolvation gas. Probe temperature was set to 400 ºC. Total mass spectra were reconstructed from the ion series using the MaxEnt algorithm preinstalled on MassLynx software (version 4.1 from Waters)

Protein concentrations were determined by Nanodrop and the Bradford assay (Bio-Rad).

2. Experimental Procedures for Chemical Synthesis

Synthesis of 3-(3-bromopropoxy)benzaldehyde

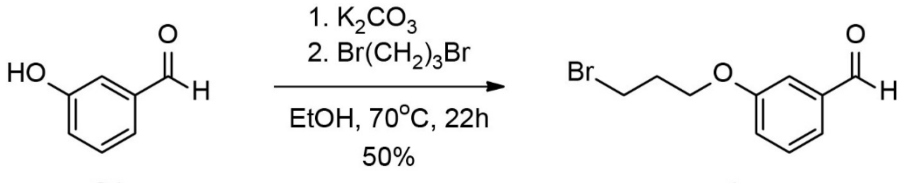

S1 1

Literature Basis: *Chem. Sci.,* **2014**, *5*, 489
In a round-bottom flask, under inert atmosphere, 3-hydroxybenzaldehyde (1.0 g, 8.2 mmol) and potassium carbonate (1.7 g, 12.0 mmol) were dissolved in ethanol (47 mL). The reaction mixture was left to react at 70 ºC for 30 min, after which it was cooled to room temperature and 1,3-dibromopropane added. The mixture was then stirred at 70 ºC for over 22 h. After cooling to room temperature, the reaction mixture was filtrated under vacuum using ethanol as the wash solvent. Concentration under vacuum afforded the crude product that was purified with a flash chromatography to isolate compound **1** in 50% yield (1.0 g). ^{1}H-NMR (300 MHz, $CDCl_3$): δ = 9.93 (s, 1H), 7.45-7.35 (m, 3H), 7.14 (dt, *J* = 6.4, 2.7 Hz, 1H), 4.12 (t, *J* = 5.8 Hz, 2H), 3.58 (t, *J* = 6.4 Hz, 2H), 2.30 (quint, *J* = 6.1 Hz, 2H). ^{13}C-NMR (75 MHz, $CDCl_3$): δ = 192.03, 159.19, 137.75, 130.11, 123.63, 121.76, 112.80, 65.50, 32.10, 29.87.

Synthesis of 3-(3-azidopropoxy)benzaldehyde

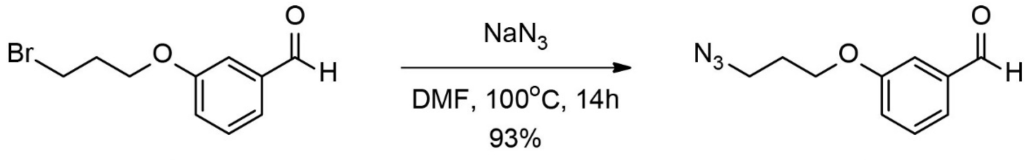

1 2

Literature Basis: *Chem. Sci.,* **2014**, *5*, 489
In a round-bottom flask, compound **1** (0.89 g, 3.7 mmol) was dissolved in DMF (10.2 mL) at room temperature. Sodium azide (0.315 g, 4.8 mmol) was then added to this solution and the reaction mixture heated to 100 ºC. After 14h, the mixture was poured to water and extracted with dichloromethane. Concentration under vacuum afforded the crude product that was purified with a flash chromatography to isolate compound **2** in 93% yield (0.7 g). ^{1}H-NMR (300 MHz, $CDCl_3$): δ = 9.92 (s, 1H), 7.43-7.34 (m, 3H), 7.13 (dt, *J* = 6.3, 2.7 Hz, 1H), 4.06 (t, *J* = 5.9 Hz, 2H), 3.48 (t, *J* = 6.6 Hz, 2H), 2.03 (quint, *J* = 6.3 Hz, 2H). ^{13}C-NMR (75 MHz, $CDCl_3$): δ = 192.02, 159.19, 137.76, 130.09, 123.62, 121.74, 112.73, 64.74, 48.07, 28.59. TOF HRMS ESI+ for $[M+Na]^+$: calculated m/z = 228.0749; observed m/z = 228.0689.

Synthesis of 1-(3-azidopropoxy)-3-(dibromomethyl)benzene and 1-(3-azidopropoxy)-3-(bromochloromethyl)benzene

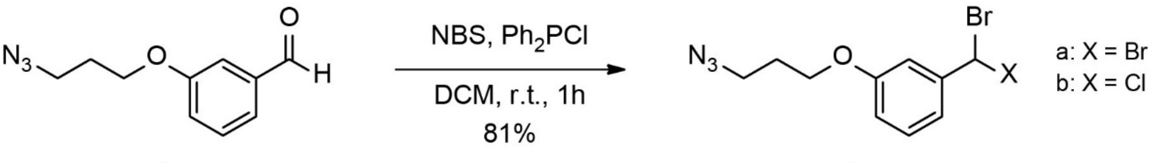

2 3

Literature Basis: *Syn. Comm.,* **2008**, *38(22)*, 4023
In a flame-dried round-bottom flask, under inert atmosphere, *N*-bromosuccinimide (0.35 g, 1.95 mmol) was dissolved in previously distilled dichloromethane (4 mL), followed by the addition of chlorodiphenylphosphine (0.36 mL, 1.95 mmol) at room temperature. A solution of compound **2** (0.2 g, 0.975 mmol) in freshly distilled dichloromethane (0.9 mL) was added to the reaction mixture and left to stir for 1 h. Then, 5 mL of water were added to the mixture and a multiple extraction was performed with dichloromethane. The combined organic layers were washed with brine, dried over anhydrous magnesium sulfate, filtered and concentrated under vacuum. A flash chromatography was performed to isolate a mixture of compounds **3a** and **3b** in 81% yield (0.28 g, 25% of **3a** and 75% of **3b**). ^{1}H-NMR (300 MHz, $CDCl_3$): δ = 7.26-7.19 (m, 1H), 7.10-7.05 (m, 2H), 6.82 (tdd, *J* = 8.3, 2.5, 0.9 Hz, 1H), 6.56 (s, 1H), 4.02 (t, *J* = 5.9 Hz, 2H), 3.47 (t, *J* = 6.6 Hz, 2H), 2.01 (quint, *J* = 6.4 Hz, 2H). ^{13}C-NMR (75 MHz, $CDCl_3$): δ = 158.83, 158.71, 143.32, 142.63, 129.83, 129.72, 118.73, 118.55, 116.15, 112.90, 112.44, 112.28, 64.74, 57.30, 48.21, 40.93, 28.76. TOF HRMS ESI+ $[M+Na]^+$: calculated m/z = 325.9672; observed m/z = 325.9424.

Synthesis of the acetyl donor (3-(3-azidopropoxy)phenyl)methylenediethanethioate

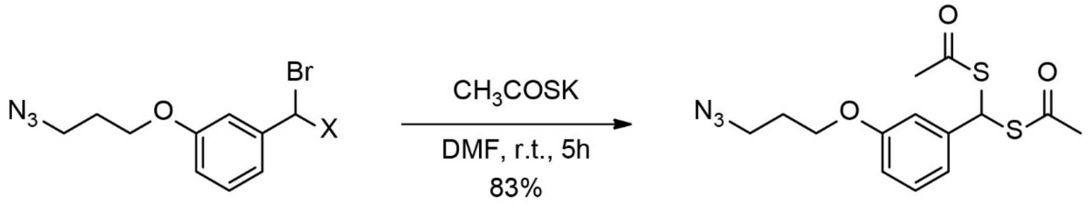

3

4

Literature Basis: *Org. Lett.,* **2014**, *16*, 4536

In a round-bottom flask, under inert atmosphere, compound **3** (0.23 g, 0.66 mmol) was mixed with potassium thioacetate (0.23 g, 2.0 mmol) dissolved in dried DMF (3.4 mL) at room temperature. The reaction was stirred for 5 h, after which 12 mL of water were added. A multiple extraction was performed using dichloromethane and the combined organic layers were dried over anhydrous sodium sulfate, filtered and concentrated under vacuum. A flash chromatography was performed to isolate compound **4** in 83% yield (0.18 g). ^{1}H-NMR (300 MHz, $CDCl_3$): δ = 7.21 (t, *J* = 7.9 Hz, 1H), 7.03-6.96 (m, 2H), 6.79 (ddd, *J* = 8.3, 2.5, 0.9 Hz, 1H), 6.21 (s, 1H), 4.03 (t, *J* = 5.9 Hz, 2H), 3.50 (t, *J* = 6.6 Hz, 2H), 2.31 (s, 6H), 2.03 (quint, *J* = 6.5 Hz, 2H). ^{13}C-NMR (75 MHz, $CDCl_3$): δ = 192.59, 158.75,140.88, 129.85, 120.28, 114.29, 113.91, 64.52, 48.21, 48.01, 30.04, 28.75. TOF HRMS ESI $[M+Na]^+$: calculated m/z = 362.0609; observed m/z = 362.0601.

3. Conjugation experiments in histone peptides

Stock solutions of peptides K4C and K14C (1.26 mM in ammonium acetate buffer, pH 8), maleimide-DBCO (2.33 mM in acetonitrile), maleimide-dummy (43 mM in acetonitrile), compound **4** (44 mM in acetonitrile) and ammonium acetate buffer (20mM) were freshly prepared prior to use.

Histone K4C and K14C peptide assessment and cysteine acetylation promoted by compound 4

To confirm the purity of the peptides and the reactivity of their cysteine residues, independent aliquots of K4C and K14C were diluted to 20 µM in ammonium acetate buffer (20 mM, pH 8.0) and LRMS analysis was performed (**Figures S1** and **S2**). This was followed by incubation with the previously synthesized acetyl donor (compound **4**), which is known from the literature to rapidly acetylate cysteine residues.[1] The latter was performed using an excess of 10 eq. of reagent for 1 h and 350 rpm at 25 ºC in the same buffer. As expected, the cysteine residue of both peptides was fully acetylated under these reaction conditions (**Figures S3** and **S4**).

General procedure to generate acetylated histone peptides

Conjugation at the free cysteine of K4C and K14C peptides with the maleimide-DBCO clickable handle and subsequent site-specific acetylation following SPAAC reaction with compound **4**

Independent aliquots of histone peptides K4C and K14C were diluted to 20 µM in ammonium acetate buffer (20 mM, pH 8.0) and 2 eq. of maleimide-DBCO were then added. The mixtures were left to react for 2 h at 25 ºC and LRMS analysis was then performed (**Figures S5** and **S6**). Subsequently, 8 eq. of compound **4** (160 µM) were added and the reaction mixtures were shaken for an additional hour at the same temperature. The resulting solutions of modified K4C and K14C peptides were filtered and injected in the mass spectrometer (**Figures S7** and **S8**). To evaluate the stability of the obtained product, a reassessment by mass spectrometry was made after one month, with the aliquots being kept in refrigerated conditions at 4 ºC in the meantime. Results are shown in **Figures S9** and **S10** for the modified K4C and K14C peptides, respectively, with no significant degradation observed.

Confirmation of K9 acetylation in the modified K4C and K14C peptides by MS/MS analysis

High resolution MS/MS was performed using a SolariX XR 7T Fourier Transform Ion Cyclotron Resonance (FT-ICR) mass spectrometer (Bruker Daltonics) to confirm that acetylation occurred at position K9 instead of K14 (for peptide K4C) or K4 (for peptide K14C). To this end, a 250 µL solution of the acetylated K4C or K14C peptides (20 µM) was diluted two-fold with acetonitrile and injected. Data was processed in Data Analysis (Bruker) and the following molecular weight information was provided: reduced Cys residue; N-terminal acetyl substituent; C-terminal amide substituent; acetyl Lys residue; molecular structure of the SPAAC reaction product attached to the Cys residue. The expected values for both *b* and *z* ions for peptide K4C and *b* and *y* ions for peptide K14C can be found in Tables **S1** and **S2**. In both cases, the peptides which were found with an error below 3 or 28 ppm are highlighted in red.

Acetylated K4C Histone Peptide

ARTC**QTAR<u>K</u>STGGKA (underlined – acetylated Lys residue, double asterisk – deacetylated SPAAC product)
MH^+ (mono) = 2251.083; MH^+ (avg) = 2252.496; MS/MS margin = 3 ppm

Table S1. MS/MS results for the acetylated K4C peptide

	Ala	Arg	Thr	Cys	Gln	Thr	Ala	Arg	Lys	Ser	Thr	Gly	Gly	Lys	Ala
**b ions**	114.055	270.156	371.204	1106.451	1234.510	1335.558	1406.595	1562.696	1732.801	1819.833	1920.881	1977.902	2034.924	2163.019	2233.072
**z ions**	72.044	200.139	257.161	314.182	415.230	502.262	672.368	828.469	899.506	1000.553	1128.612	1863.860	1964.907	2121.008	2234.056
	Ala	Lys	Gly	Gly	Thr	Ser	Lys	Arg	Ala	Thr	Gln	Cys	Thr	Arg	Ala

Acetylated K14C Histone Peptide

ARTKQTAR<u>K</u>STGGC**A (underlined – acetylated Lys residue, double asterisk - deacetylated SPAAC product)
MH^+ (mono) = 2251.083; MH^+ (avg) = 2252.496; MS/MS margin = 28 ppm

Table S2. MS/MS results for the acetylated K14C peptide

	Ala	Arg	Thr	Lys	Gln	Thr	Ala	Arg	Lys	Ser	Thr	Gly	Gly	Cys	Ala
**b ions**	114.055	270.156	371.204	499.299	627.357	728.405	799.442	955.543	1125.649	1212.681	1313.728	1370.750	1427.771	2163.019	2233.072
**y ions**	89.071	824.318	881.340	938.361	1039.409	1126.441	1296.547	1452.648	1523.685	1624.733	1752.791	1880.886	1981.934	2138.035	2251.083
	Ala	Cys	Gly	Gly	Thr	Ser	Lys	Arg	Ala	Thr	Gln	Lys	Thr	Arg	Ala

Control reactions (1) of the acetylated K4C and K14C peptides with iodoacetamide

Following the general procedure for maleimide-DBCO conjugation and subsequent mono-acetylation, we set out to prove that the cysteine had reacted with the maleimide reagent and that in fact no subsequent reaction could be performed on this residue. For this purpose, an aliquot of the acetylated product was incubated with 250 µM of iodoacetamide in ammonium acetate buffer (20 mM, pH 8.0) for up to 21 h at 25 ºC. In this assay, we searched for a possible product of cysteine reaction with iodoacetamide (calculated mass = 2308.57 Da). However, this was never detected, therefore proving the lack of reactivity of the modified K4C (**Figures S11** and **S12**) and K14C (**Figures S13** and **S14**) peptides against cysteine-modifying agents.

Control reactions (2) with a peptide bearing no lysine residues

We decided to perform a second set of control reactions using the same protocol of maleimide-DBCO conjugation and SPAAC reaction conditions with the acetyl donor but in a peptide without lysine residues. For this purpose, we used a commercial peptide ESBP-NoK available in the lab. As before, we decided to evaluate the peptide prior to chemical modification. Despite a few purification attempts using Pur-A-Lyzer dialysis units, this peptide presented +45 adducts (up to 4), possibly related with the formic acid complexation, which we were not able to circumvent (**Figure S15**). However, since the goal was only to determine if acetylation could occur or not, we decided to move on and evaluate the peptide's behaviour during the described protocol. An aliquot of the ESBP-NoK peptide was diluted to 20 µM in ammonium acetate buffer (20 mM, pH 8.0) and 2 eq. of maleimide-DBCO were added. The mixture was shaken for 2 h at 25 ºC (**Figure S16**). Subsequently, 8 eq. (160 µM) of the acetyl donor were added and the mixture was shaken for an additional hour at the same temperature. The resulting mass spectrum (**Figure S17**) showed no degradation of the acetyl donor, thus proving the importance of nearby lysine residues for acetylation to occur.

Control reactions (3) of the K4C and K14C peptides with a maleimide-dummy

To attest the importance of the spatially controlled SPAAC reaction in promoting acetylation at the nearest lysine residue, we also modified the histone K4C and K14C peptides with a maleimide reagent without an alkyne click counterpart to the azide. For this purpose, we decided to use *N*-(5-nitro-*ortho*-tolyl)-maleimide, henceforth referred to as maleimide-dummy, since it was readily available in the lab. We then incubated these modified peptides with the click product formed between maleimide-DBCO and compound **4** and searched for the presence of acetylation. Independent aliquots of both peptides were diluted to 20 µM in ammonium acetate buffer (20 mM, pH 8.0) and 2 eq. of maleimide-dummy were added. The mixture was left to react for 2 h and 350 rpm at 25 ºC and LRMS analysis was performed (**Figures S18** and **S19**). Subsequently, 4 eq. of maleimide-DBCO (80 µM) together with 16 eq. of compound **4** (320 µM) were added and the reaction mixture was shaken for an additional two hours at the same temperature. This excess was used to assure that no reaction would occur even under more aggressive conditions. Acetylation was not detected in both peptides, as shown in **Figures S20** and **S21** for K4C and K14C peptides, respectively.

Retro-Michael addition with β-mercaptoethanol

Following conjugation with maleimide-DBCO and subsequent mono-acetylation with **4**, the modified histone K4C and K14C peptides were incubated with 12.5 eq. of *β*-mercaptoethanol (250 µM) in ammonium acetate buffer (20 mM, pH 8.0) for up to 23 h at 25 ºC. In this assay, we searched for the presence of the acetylated peptide products with a free cysteine (calculated mass = 1618.84 Da), which were a minor product after 3 h to 4 h (**Figures S22** and **S23**). Although these became the major products after 23 h, the reaction did not reach completion due to irreversible hydrolysis of the maleimide that confers resistance to the retro-Michael addition (**Figures S24** and **S25**).

Deacetylation experiments of the modified histone K4C and K14C peptides using Sirt6

The acetylated histone K4C and K14C peptides (20 µM) containing the SPAAC product at the cysteine were incubated with human Sirt6 (1 mg/mL in 50 mM Tris, 100 mM NaCl, pH 8.0 with 20% glycerol from BioVision) to a final concentration of 30 µg/mL in ammonium acetate buffer (20 mM, pH 8.0) for 3 h at 25 ºC. In this assay, we searched for the presence of the deacetylated peptide products (calculated mass = 2209.48 Da), which were a minor product after 3 h (**Figures S26** and **S27**). Degradations started to occur after longer periods of incubation (data not shown). Nonetheless, Sirt6 was indeed able to recognize and partially deacetylate the peptides in the presence of the SPAAC product.

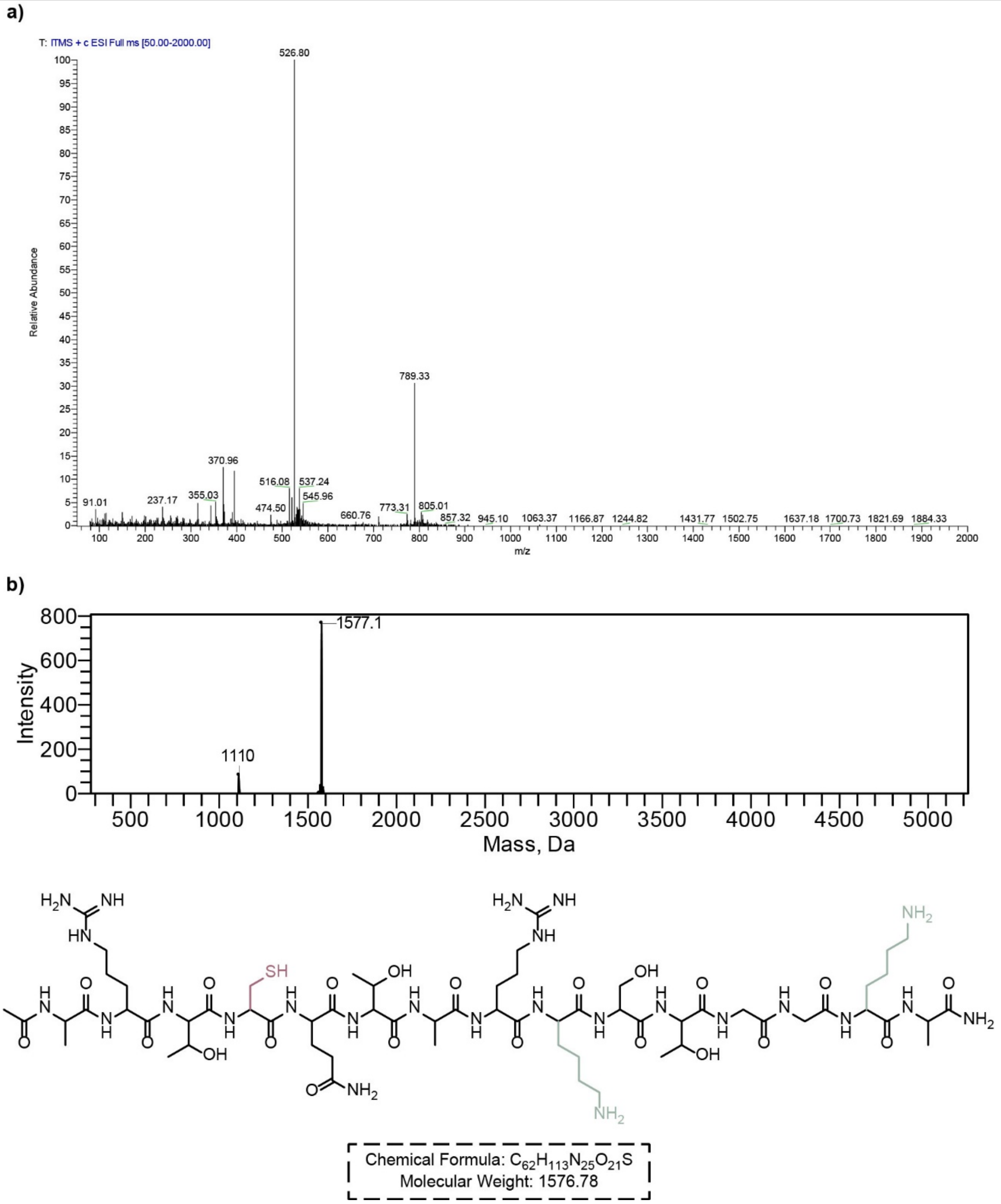

Figure S1. a) Low-resolution mass spectrum of the histone K4C peptide (20 µM) in ammonium acetate buffer (20 mM, pH 8.0) and **b)** subsequent deconvolution with representation of the major product identified: Peptide K4C [526.80 (+3), 789.33 (+2)].

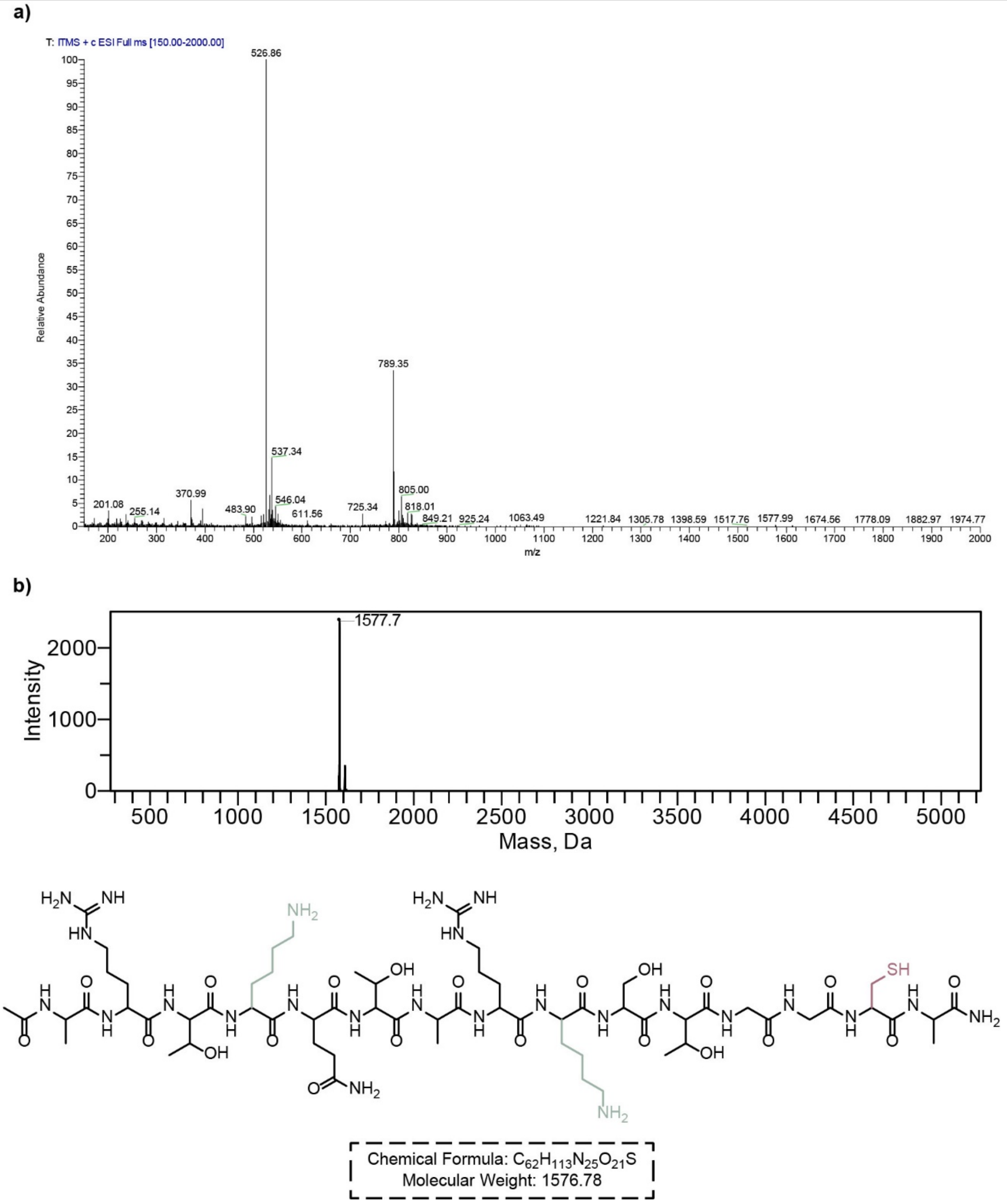

Figure S2. a) Low-resolution mass spectrum of the histone K14C peptide (20 µM) in ammonium acetate buffer (20 mM, pH 8.0) and **b)** subsequent deconvolution with structural representation of the major product identified: Peptide K14C [526.86 (+3), 789.35 (+2)].

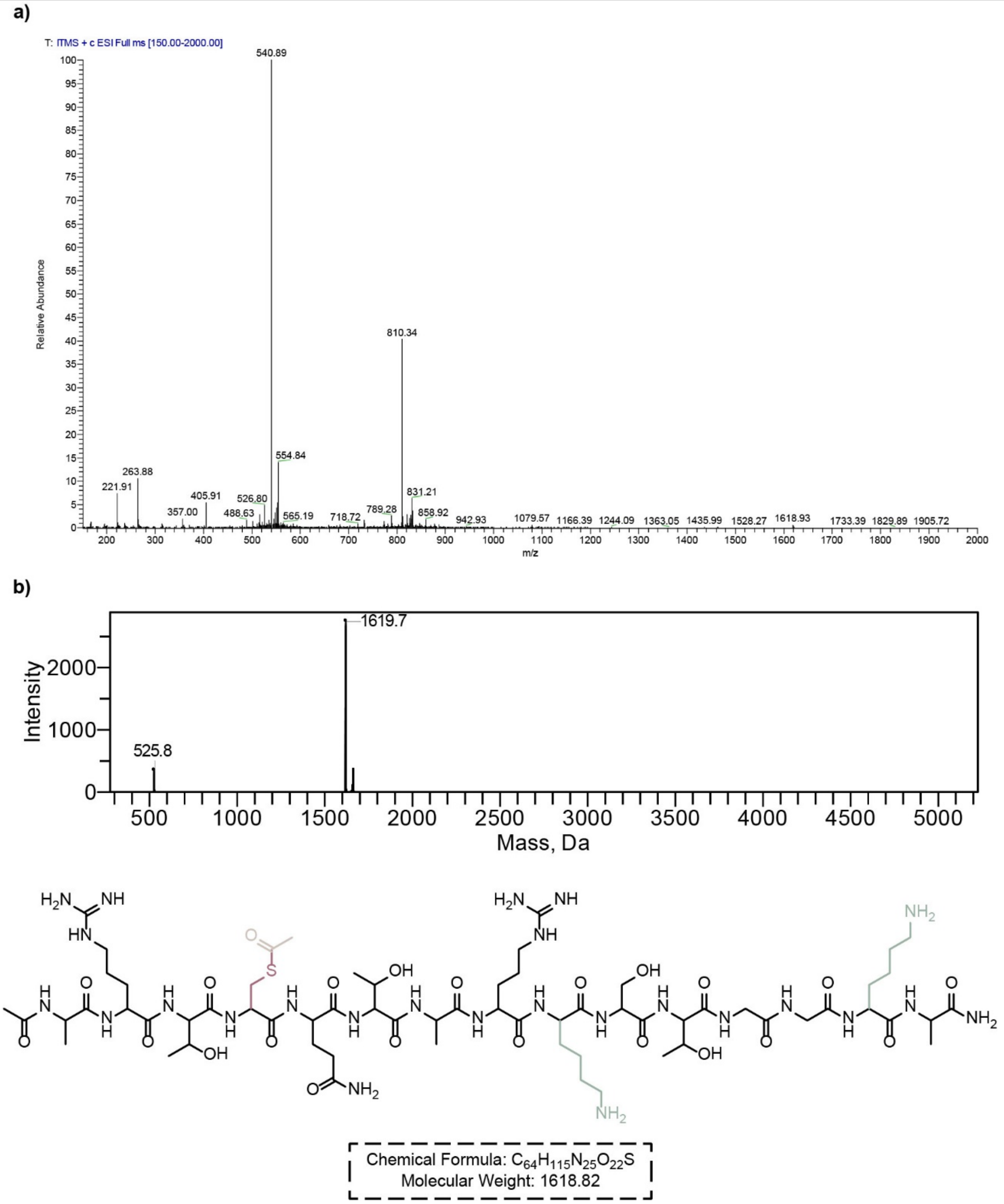

Figure S3. a) Low-resolution mass spectrum of the histone K4C peptide (20 µM) following reaction with compound **4** (200 µM) in ammonium acetate buffer (20 mM, pH 8.0) for 1 h at 25 ºC and **b)** subsequent deconvolution with structural representation of the major product identified: Peptide K4C + mono-acetylation [540.89 (+3), 810.34 (+2)].

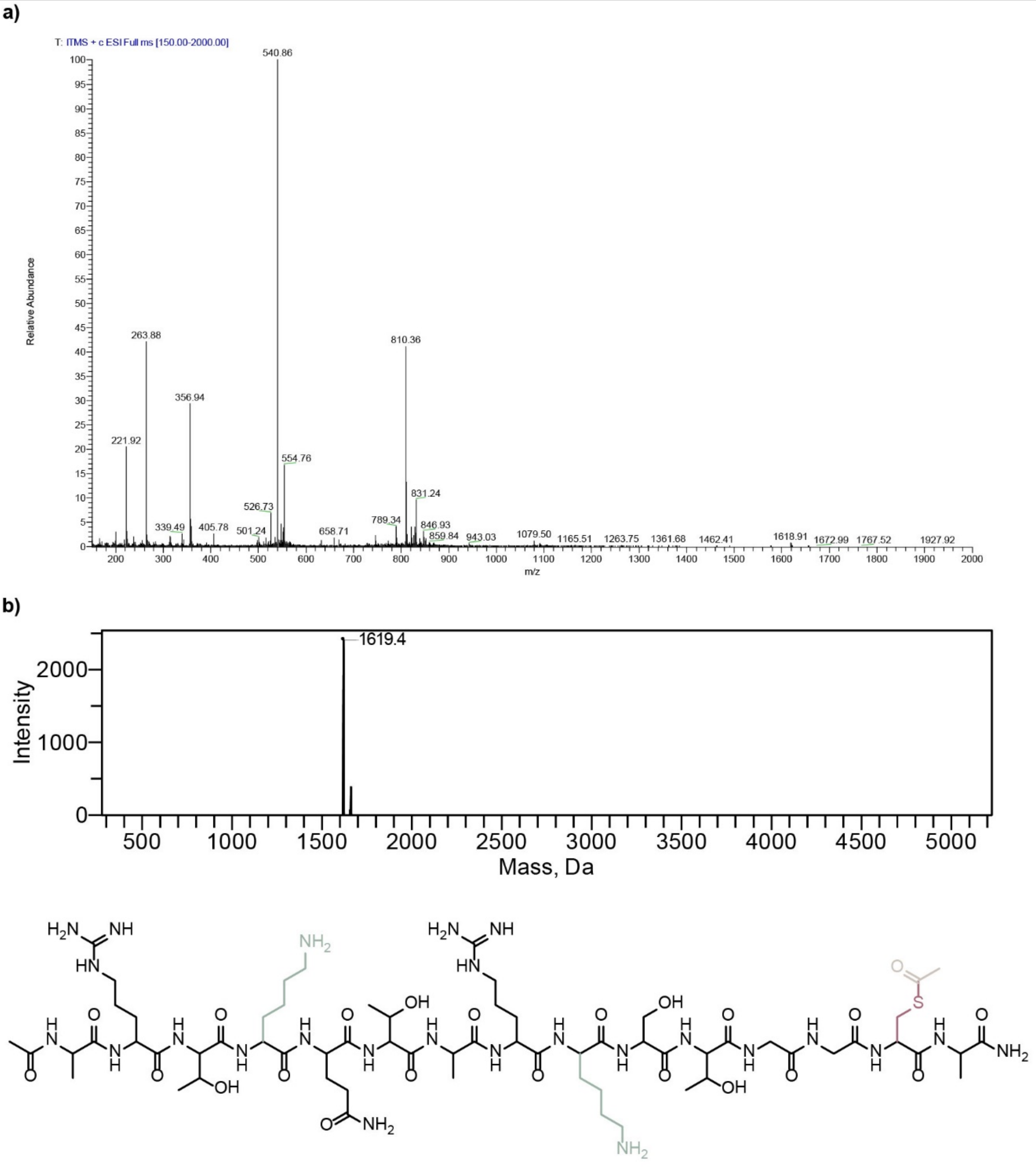

Figure S4. a) Low-resolution mass spectrum of the histone K14C peptide (20 µM) following reaction with compound **4** (200 µM) in ammonium acetate buffer (20 mM, pH 8.0) for 1 h at 25 ºC and **b)** subsequent deconvolution with structural representation of the major product identified: Peptide K14C + mono-acetylation [540.86 (+3), 810.36 (+2)].

a)

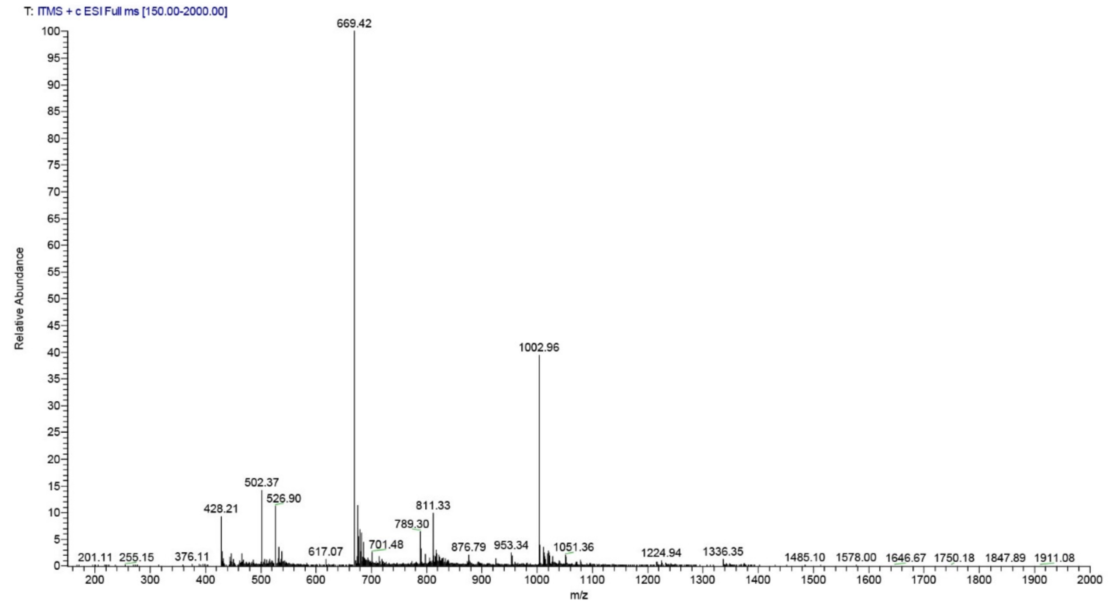

b)

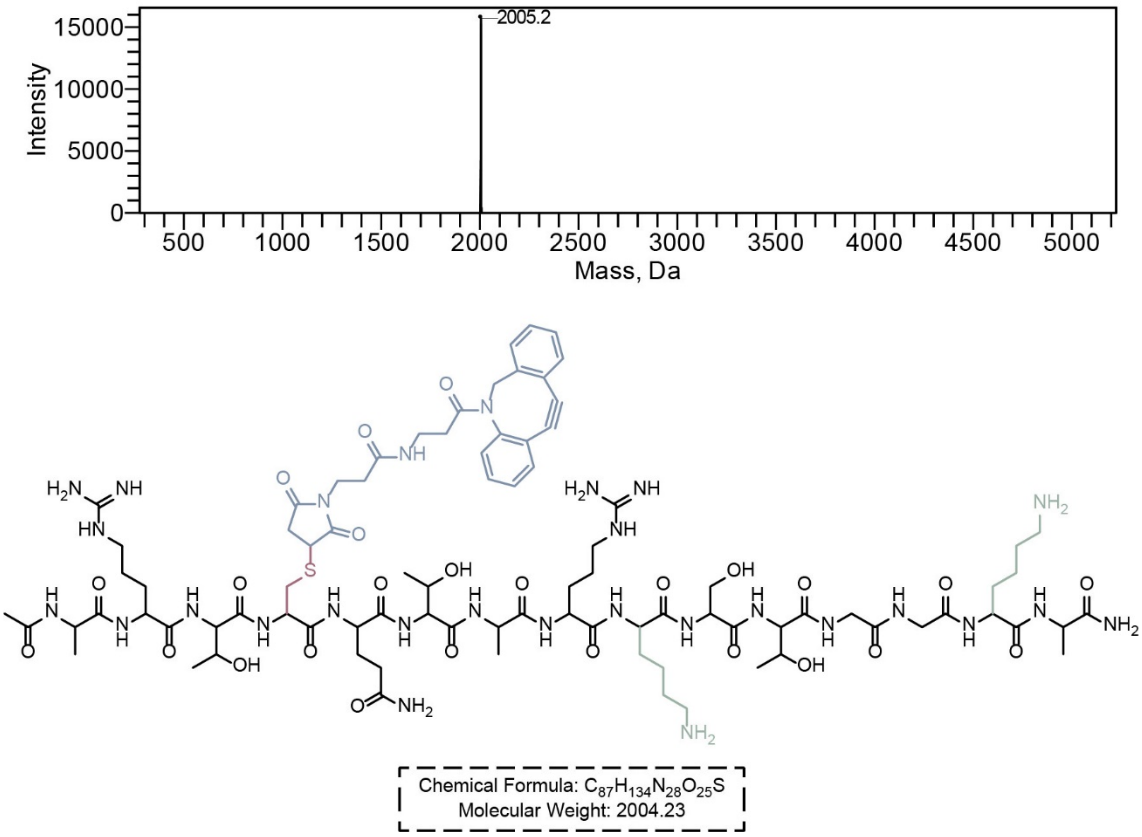

Figure S5. a) Low-resolution mass spectrum of the histone K4C peptide (20 µM) following incubation with maleimide-DBCO (40 µM) in ammonium acetate buffer (20 mM, pH 8.0) for 2 h at 25 ºC and **b)** subsequent deconvolution with structural representation of the major product identified: Peptide K4C + one maleimide-DBCO [669.42 (+3), 1002.96 (+2)].

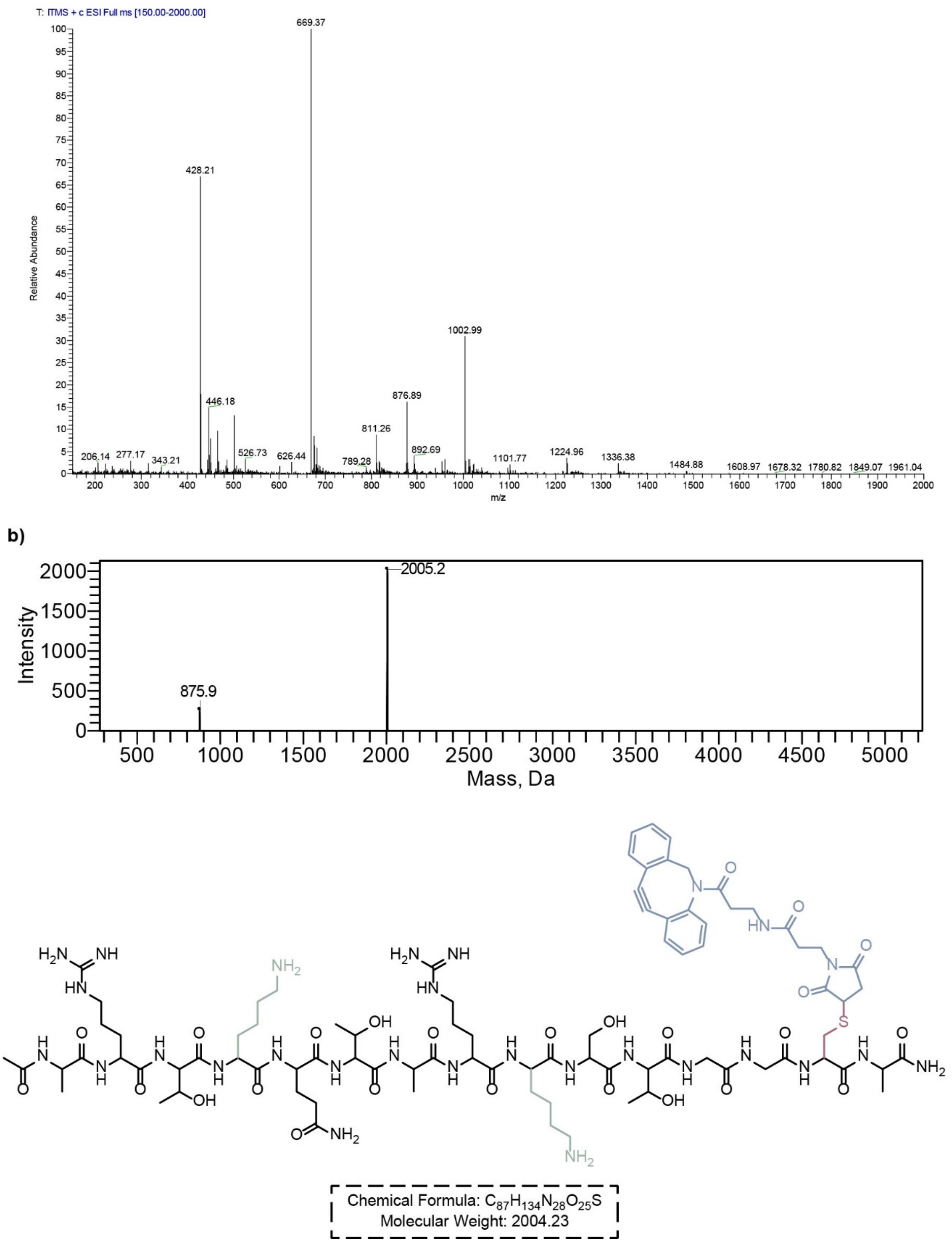

Figure S6. a) Low-resolution mass spectrum of the histone K14C peptide (20 µM) following incubation with maleimide-DBCO (40 µM) in ammonium acetate buffer (20 mM, pH 8.0) for 2 h at 25 ºC and **b)** subsequent deconvolution with structural representation of the major product identified: Peptide K14C + one maleimide-DBCO [669.37 (+3), 1002.99 (+2)].

a)

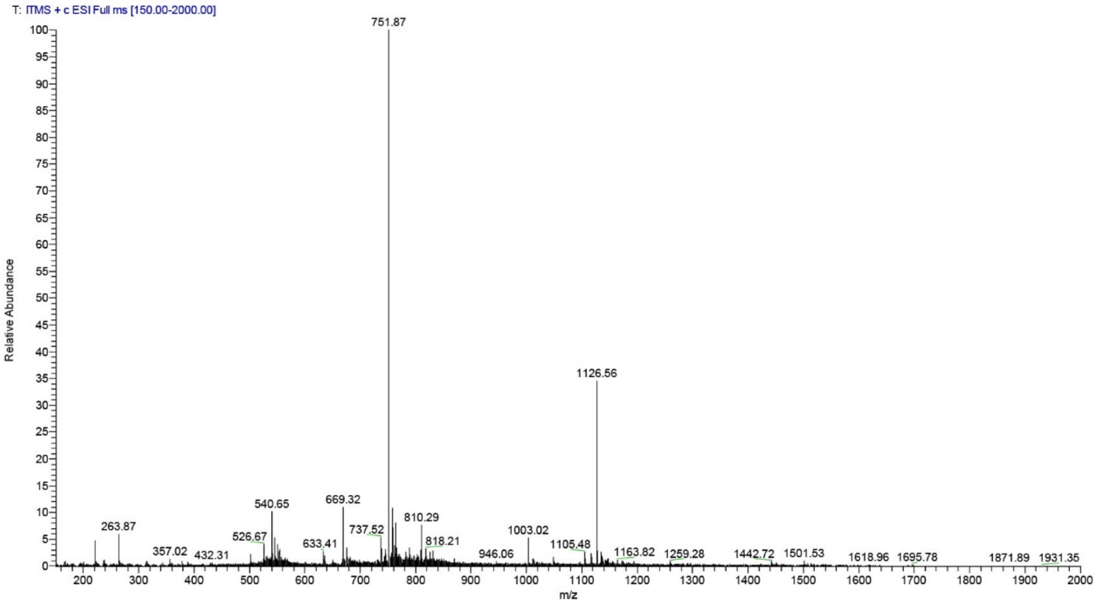

b)

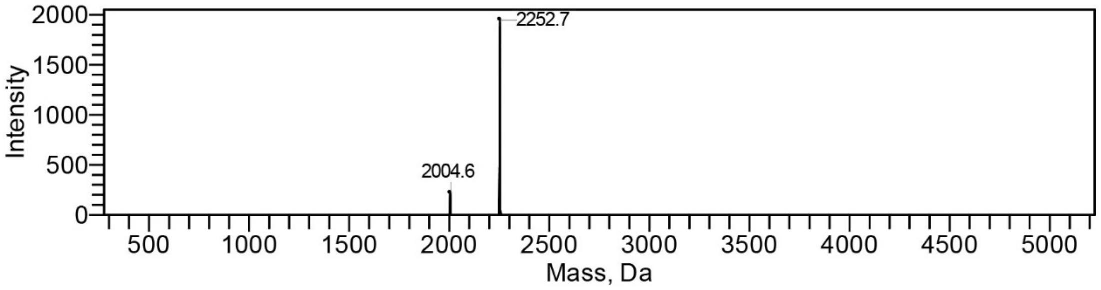

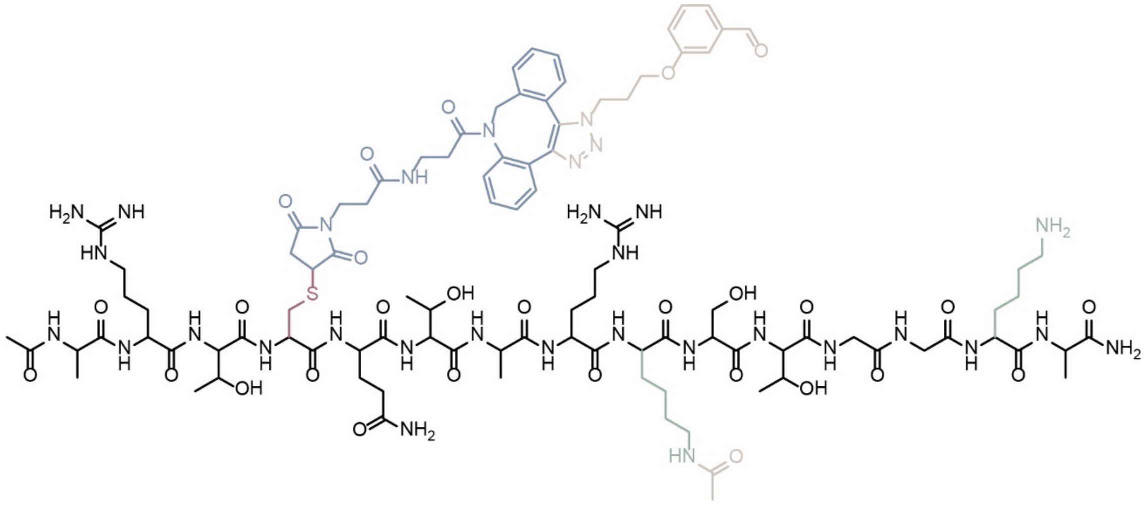

Figure S7. a) Low-resolution mass spectrum of the histone K4C peptide (20 µM) following sequential incubation with maleimide-DBCO (40 µM) and compound **4** (160 µM) in ammonium acetate buffer (20 mM, pH 8.0) for 2 h and 1 h, respectively, at 25 ºC and **b)** subsequent deconvolution with structural representation of the major product identified: Peptide K4C + one maleimide-DBCO + one deacetylated SPAAC product + mono-acetylation [751.87 (+3), 1126.56 (+2)]; Minor product identified: Peptide K4C + one maleimide-DBCO [669.32 (+3), 1003.02 (+2)].

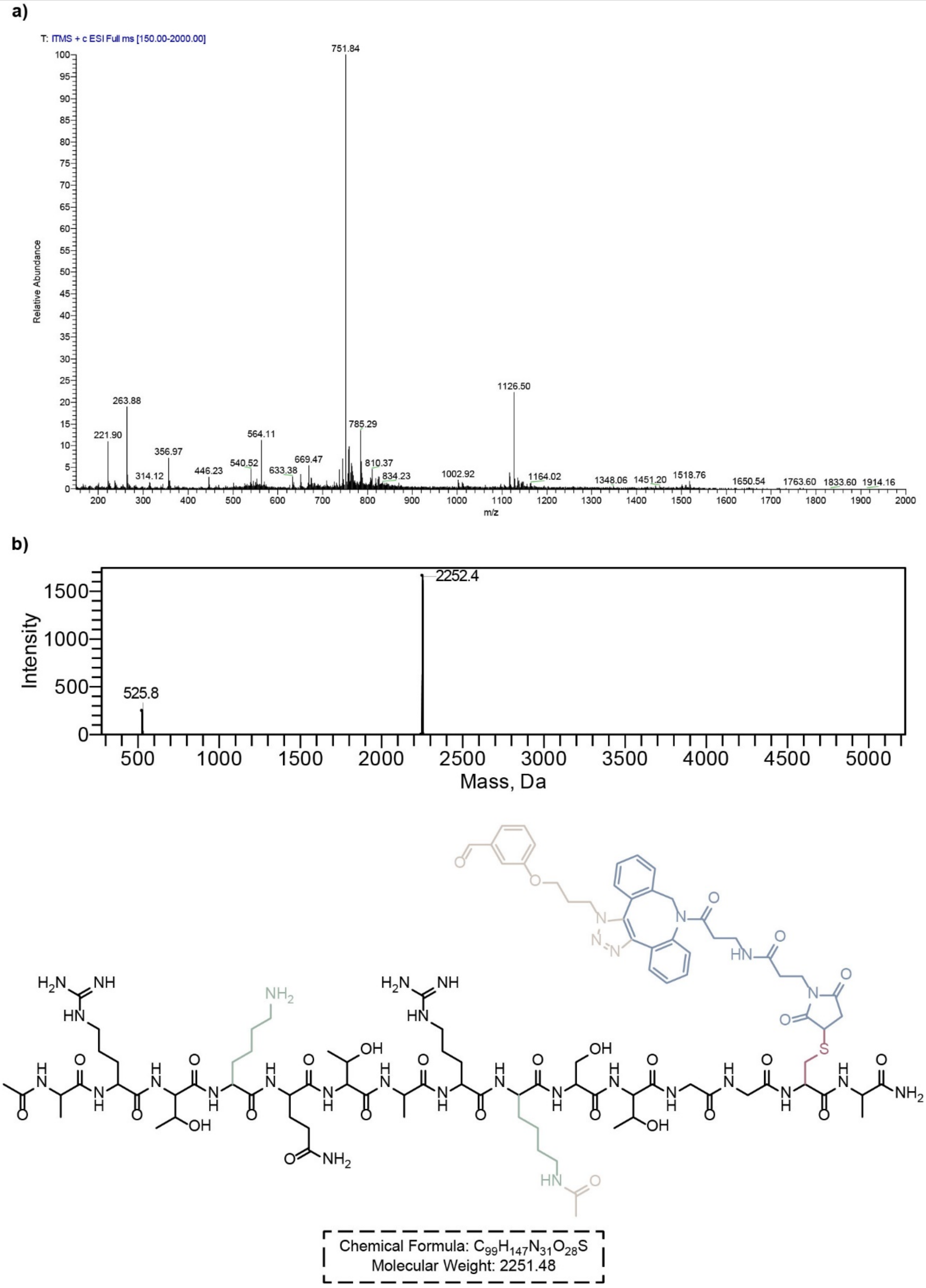

Figure S8. a) Low-resolution mass spectrum of the histone K14C peptide (20 μM) following sequential incubation with maleimide-DBCO (40 μM) and compound **4** (160 μM) in ammonium acetate buffer (20 mM, pH 8.0) for 2 h and 1 h, respectively, at 25 ºC and **b)** subsequent deconvolution with structural representation of the major product identified: Peptide K14C + one maleimide-DBCO + one deacetylated SPAAC product + mono-acetylation [751.84 (+3), 1126.50 (+2)].

a)

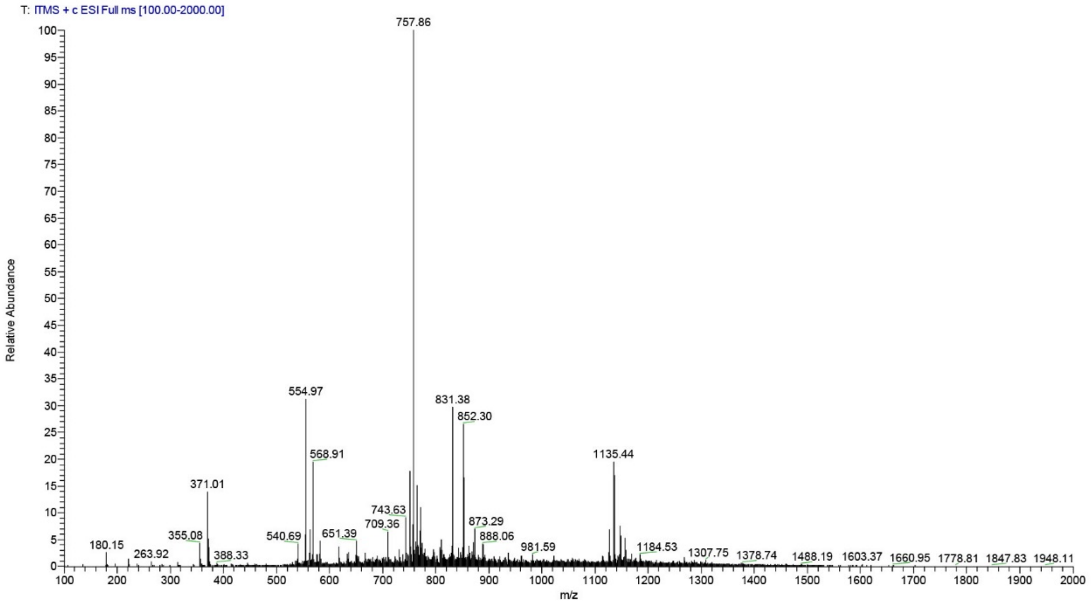

b)

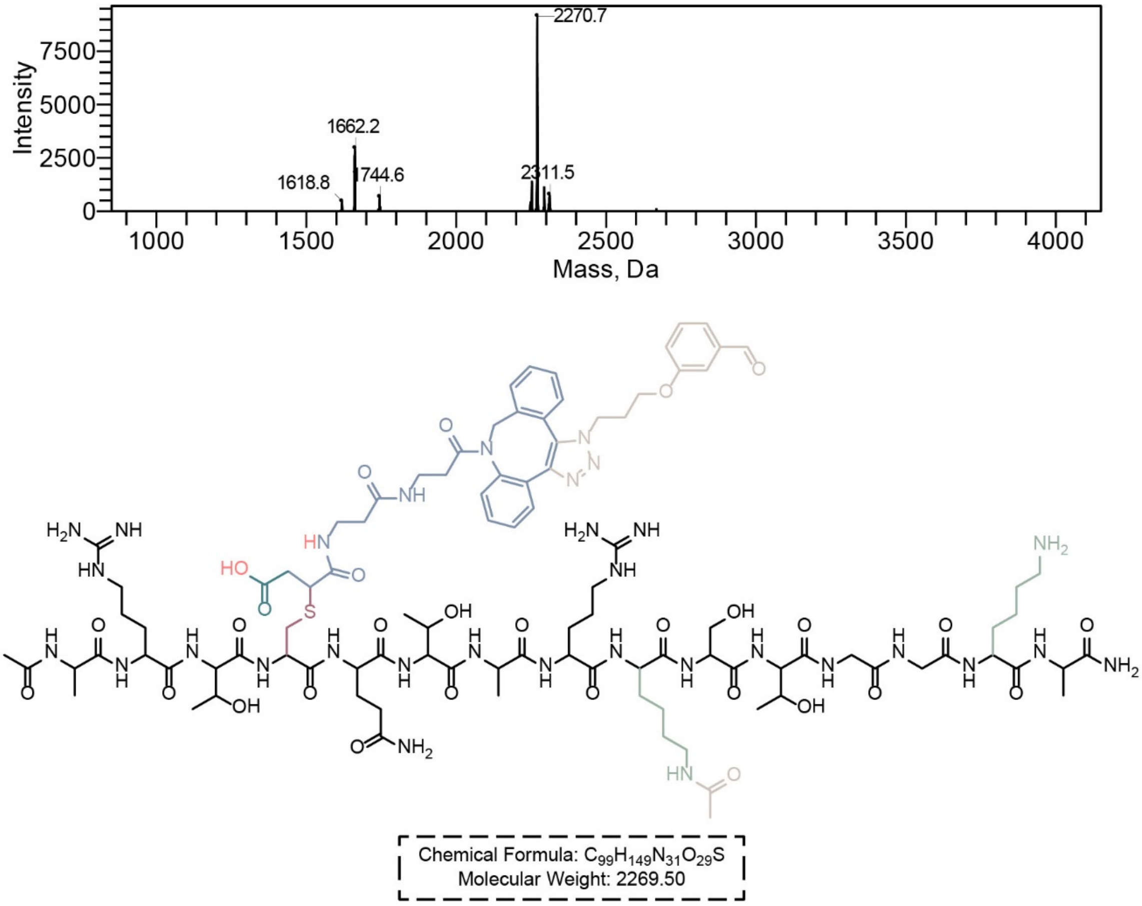

Figure S9. a) Low-resolution mass spectrum of the acetylated histone K4C peptide (20 µM) following storage at 4 ºC for 1 month and **b)** subsequent deconvolution with structural representation of the major product identified: Peptide K4C + one maleimide-DBCO + one deacetylated SPAAC product + mono-acetylation + one hydration [568.91 (+4), 757.86 (+3), 1135.44 (+2)] – hydration most likely resulting from maleimide hydrolysis.

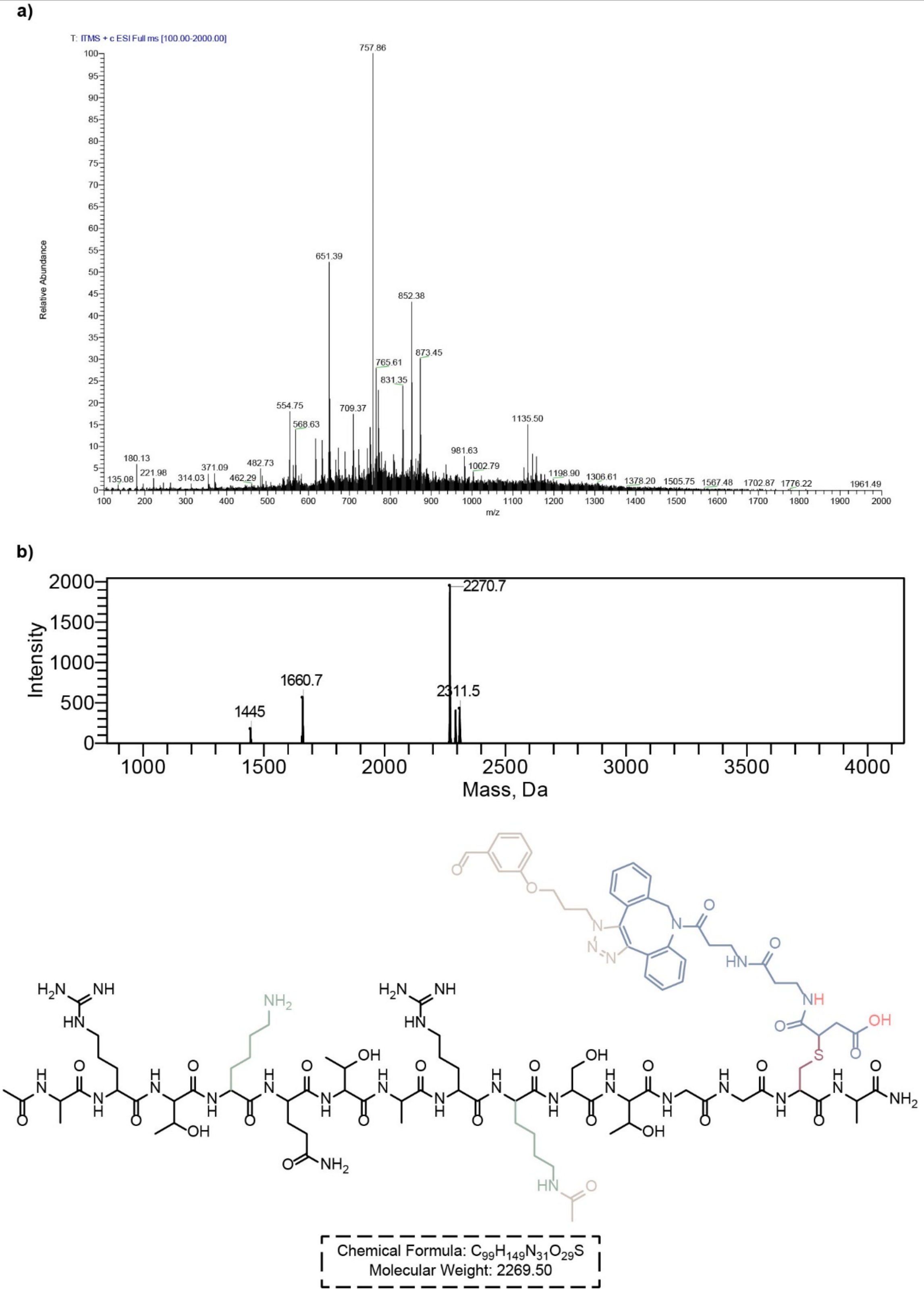

Figure S10. a) Low-resolution mass spectrum of the acetylated histone K14C peptide (20 µM) following storage at 4 ºC for 1 month and **b)** subsequent deconvolution with structural representation of the major product identified: Peptide K14C + one maleimide-DBCO + one deacetylated SPAAC product + mono-acetylation + one hydration [568.63 (+4), 757.86 (+3), 1135.50 (+2)] – hydration most likely resulting from maleimide hydrolysis.

a)

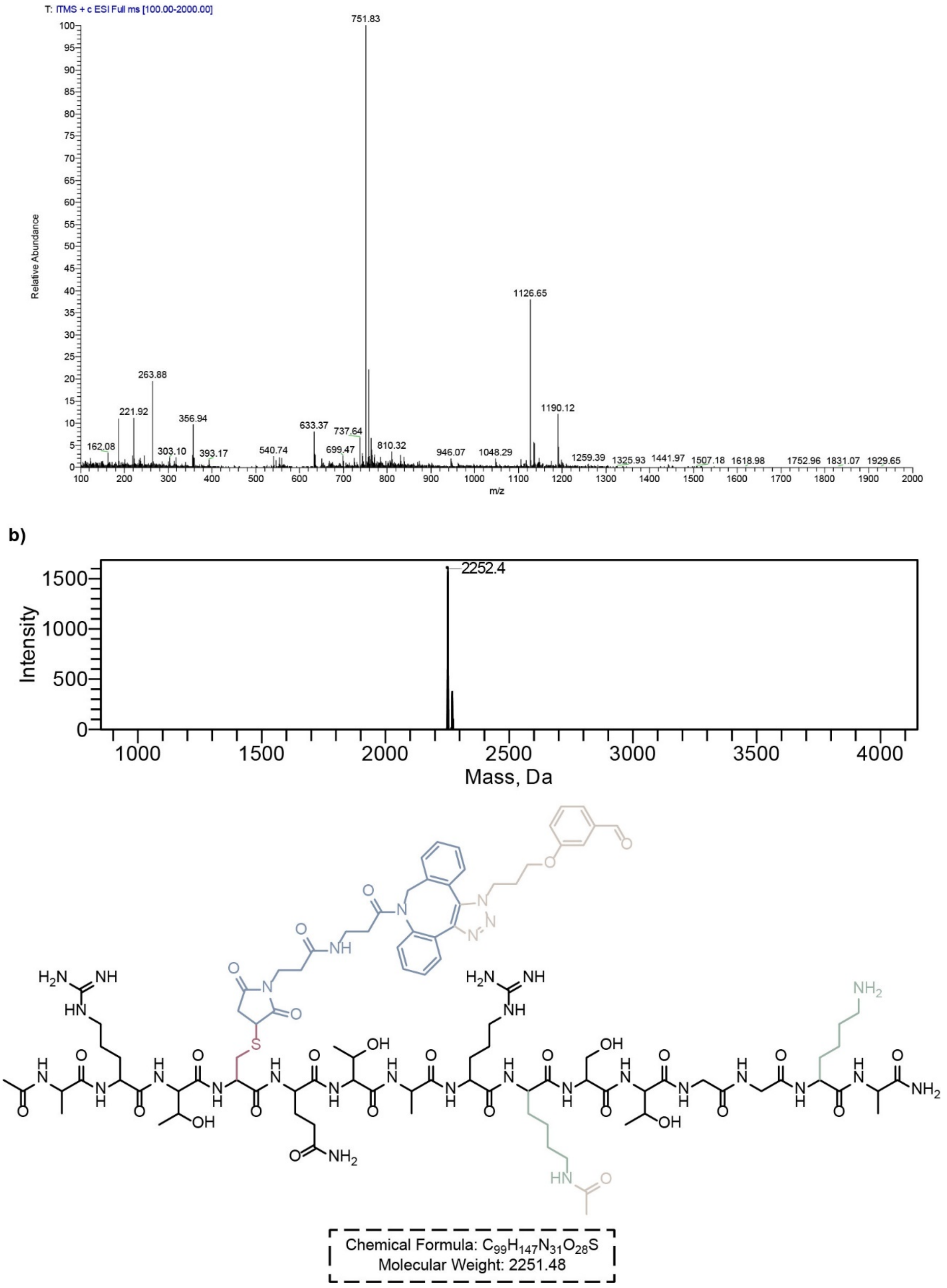

Figure S11. a) Low-resolution mass spectrum of the acetylated histone K4C peptide (20 µM) following incubation with iodoacetamide (250 µM) in ammonium acetate buffer (20 mM, pH 8.0) for 1.5 h at 25 ºC and **b)** subsequent deconvolution with structural representation of the major product identified: Peptide K4C + one maleimide-DBCO + one deacetylated SPAAC product + mono-acetylation [751.83 (+3), 1126.65 (+2)].

a)

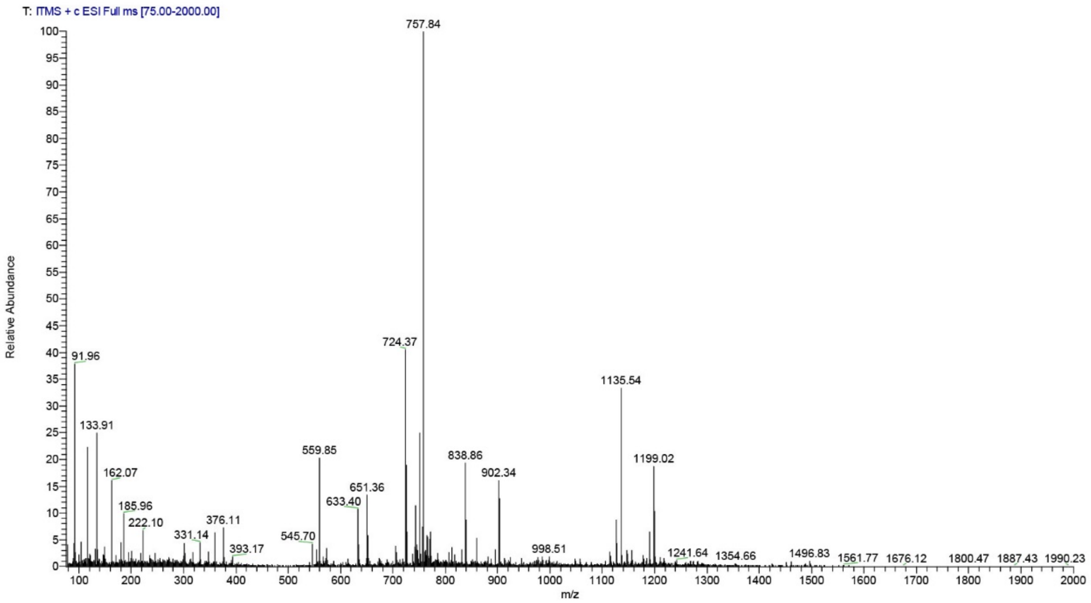

b)

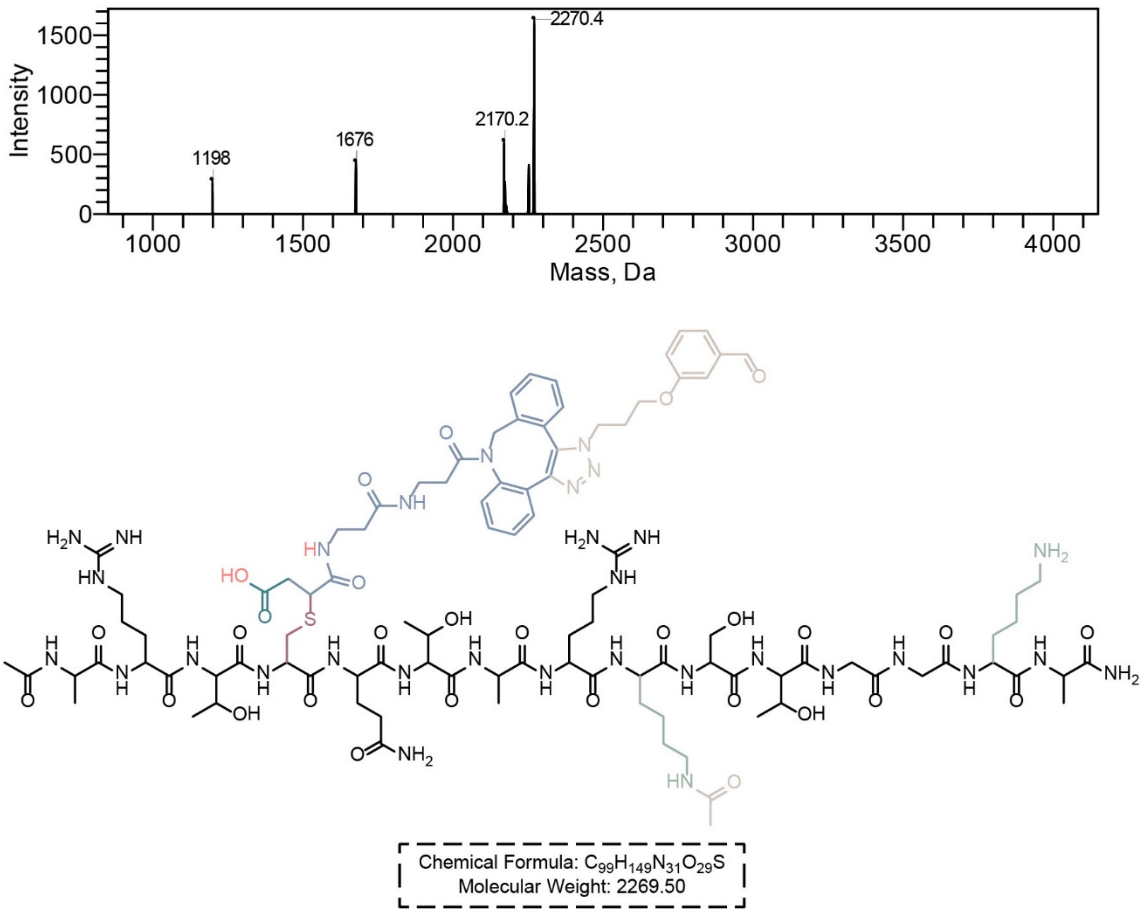

Figure S12. a) Low-resolution mass spectrum of the acetylated histone K4C peptide (20 μM) following incubation with iodoacetamide (250 μM) in ammonium acetate buffer (20 mM, pH 8.0) for 21 h at 25 ºC and **b)** subsequent deconvolution with structural representation of the major product identified: Peptide K4C + one maleimide-DBCO + one deacetylated SPAAC product + mono-acetylation + one hydration [757.84 (+3), 1135.54 (+2)] – hydration most likely resulting from maleimide hydrolysis. Some degradation products were also detected given the long reaction time.

a)

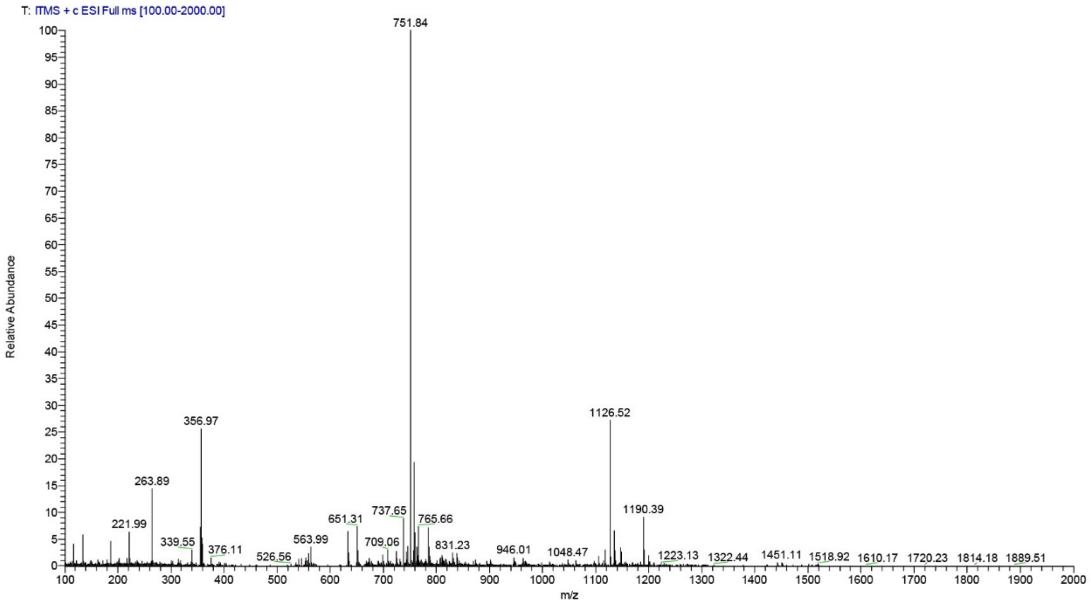

b)

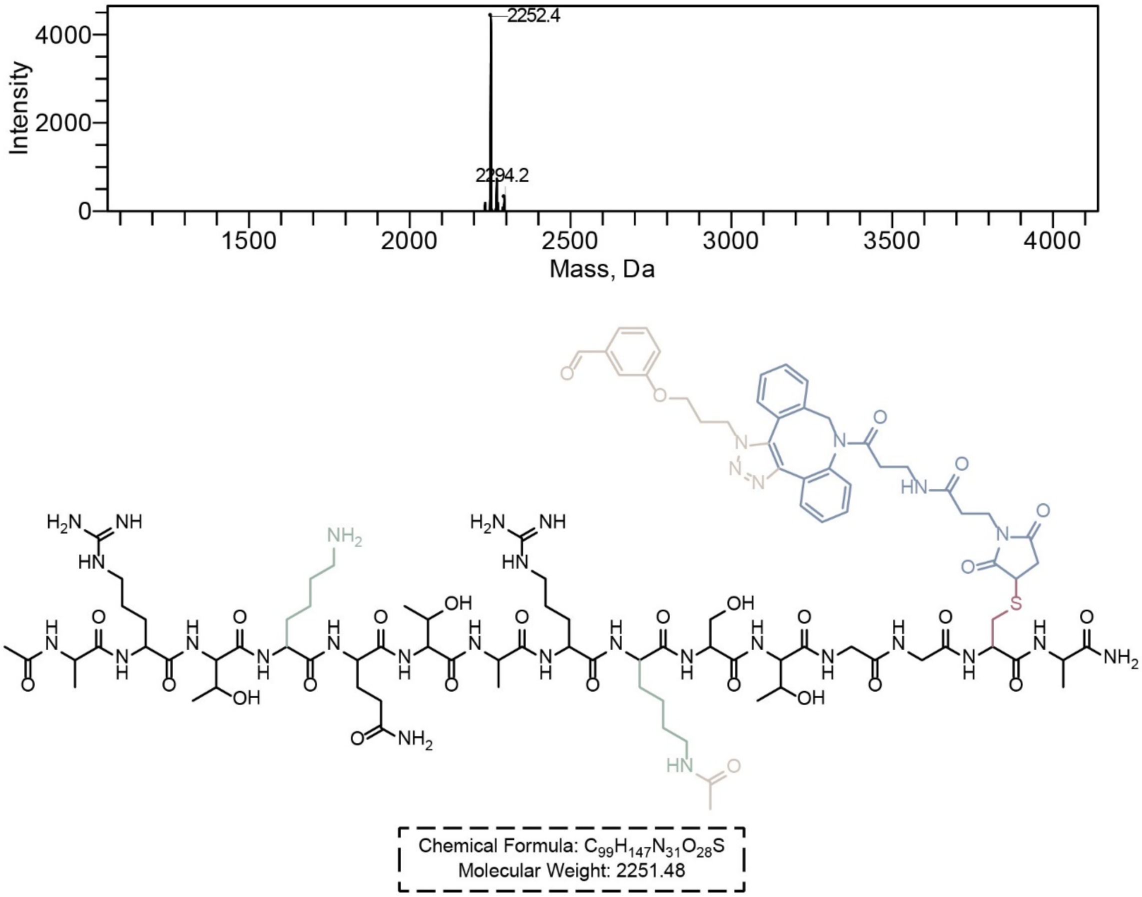

Figure S13. a) Low-resolution mass spectrum of the acetylated histone K14C peptide (20 µM) following incubation with iodoacetamide (250 µM) in ammonium acetate buffer (20 mM, pH 8.0) for 2 h at 25 ºC and **b)** subsequent deconvolution with structural representation of the major product identified: Peptide K14C + one maleimide-DBCO + one deacetylated SPAAC product + mono-acetylation [751.84 (+3), 1126.52 (+2)].

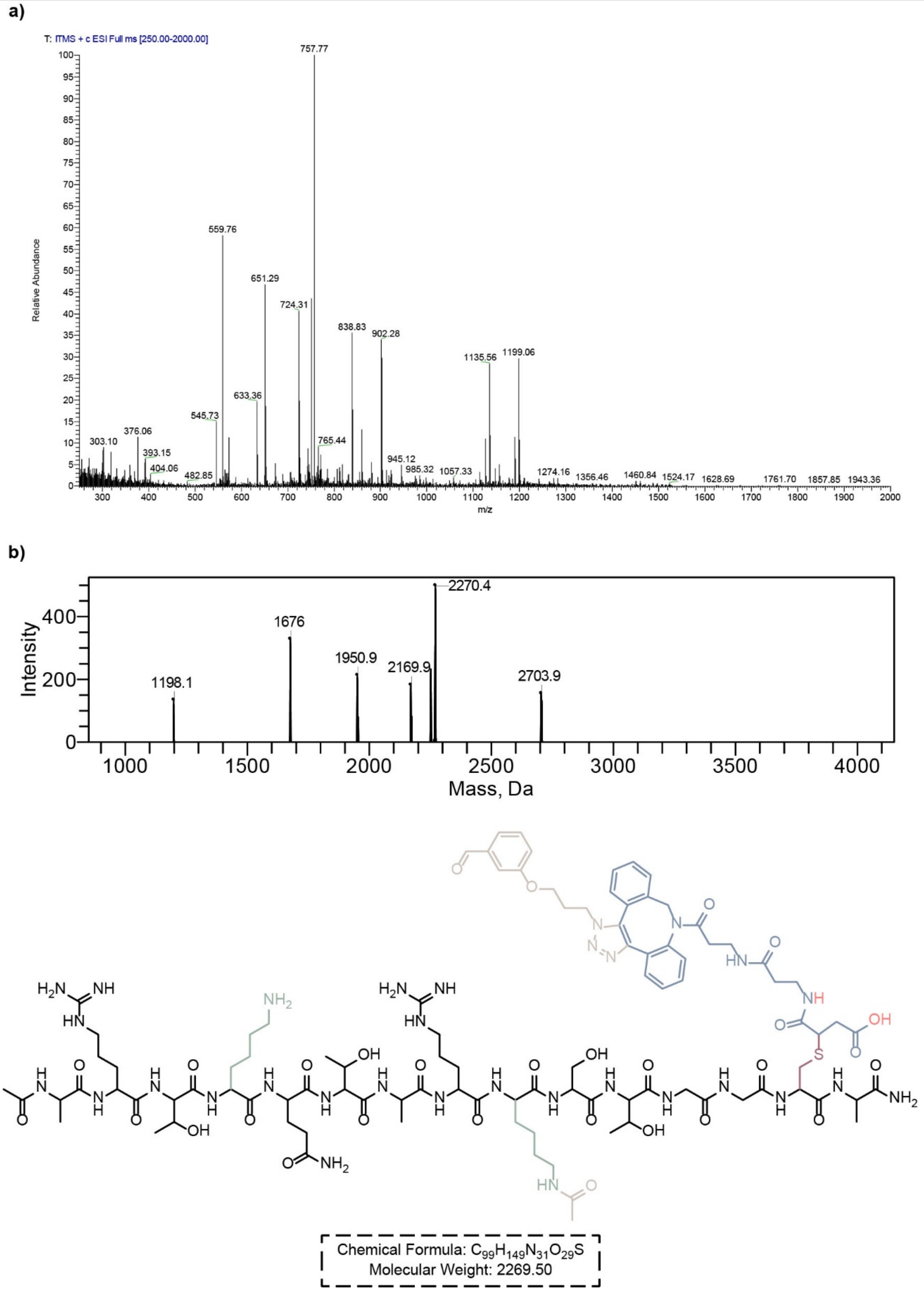

Figure S14. a) Low-resolution mass spectrum of the acetylated histone K14C peptide (20 µM) following incubation with iodoacetamide (250 µM) in ammonium acetate buffer (20 mM, pH 8.0) for 21 h at 25 ºC and **b)** subsequent deconvolution with structural representation of the major product identified: Peptide K14C + one maleimide-DBCO + one deacetylated SPAAC product + mono-acetylation + one hydration [757.77 (+3), 1135.56 (+2)] – hydration most likely resulting from maleimide hydrolysis. Some degradation products were also detected given the long reaction time.

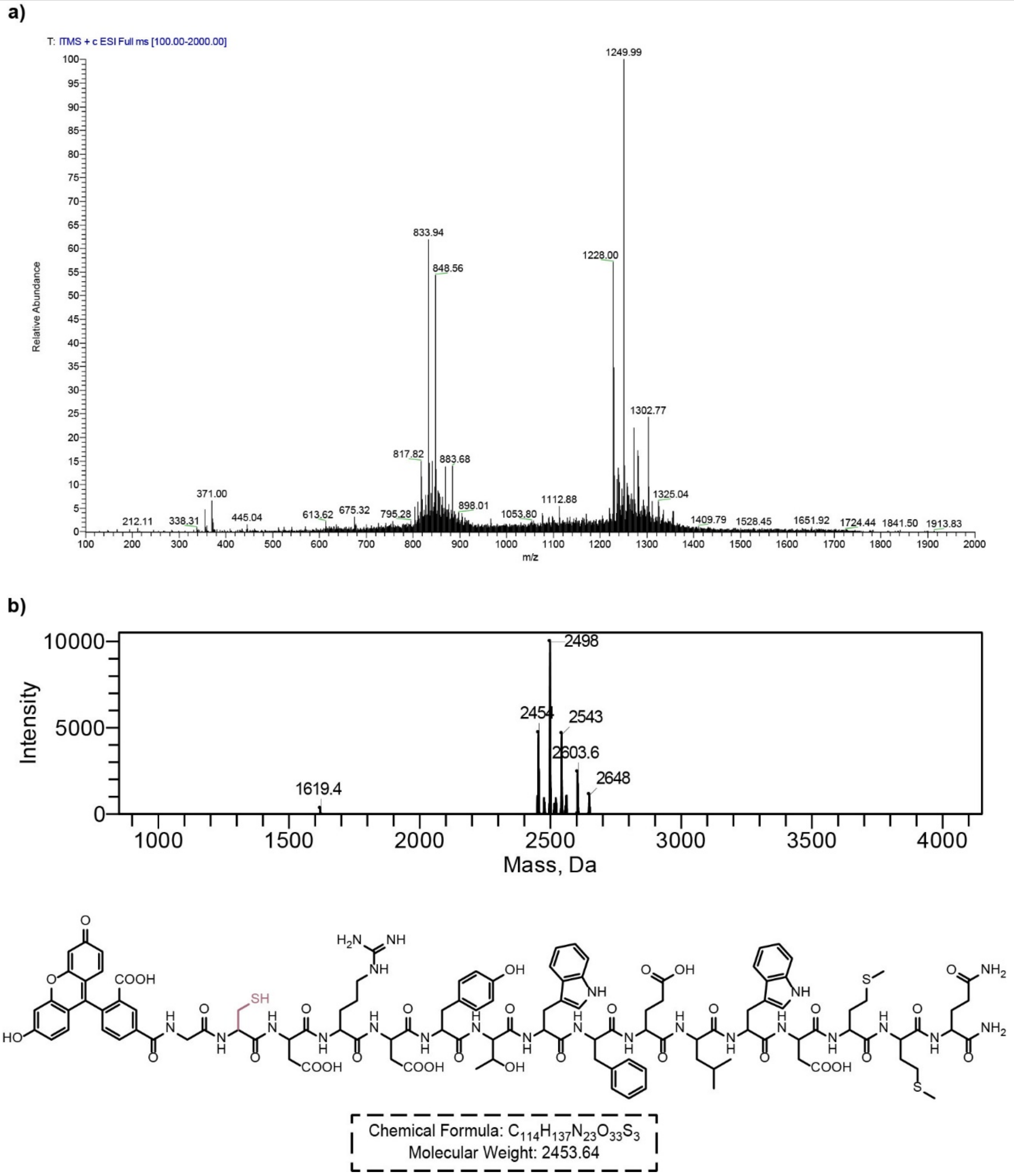

Figure S15. a) Low-resolution mass spectrum of the ESBP-NoK peptide (20 µM) in ammonium acetate buffer (20 mM, pH 8.0) and **b)** subsequent deconvolution with structural representation of the major product identified: Peptide ESBP-NoK + 45 Da [833.94 (+3), 1249.99 (+2)].

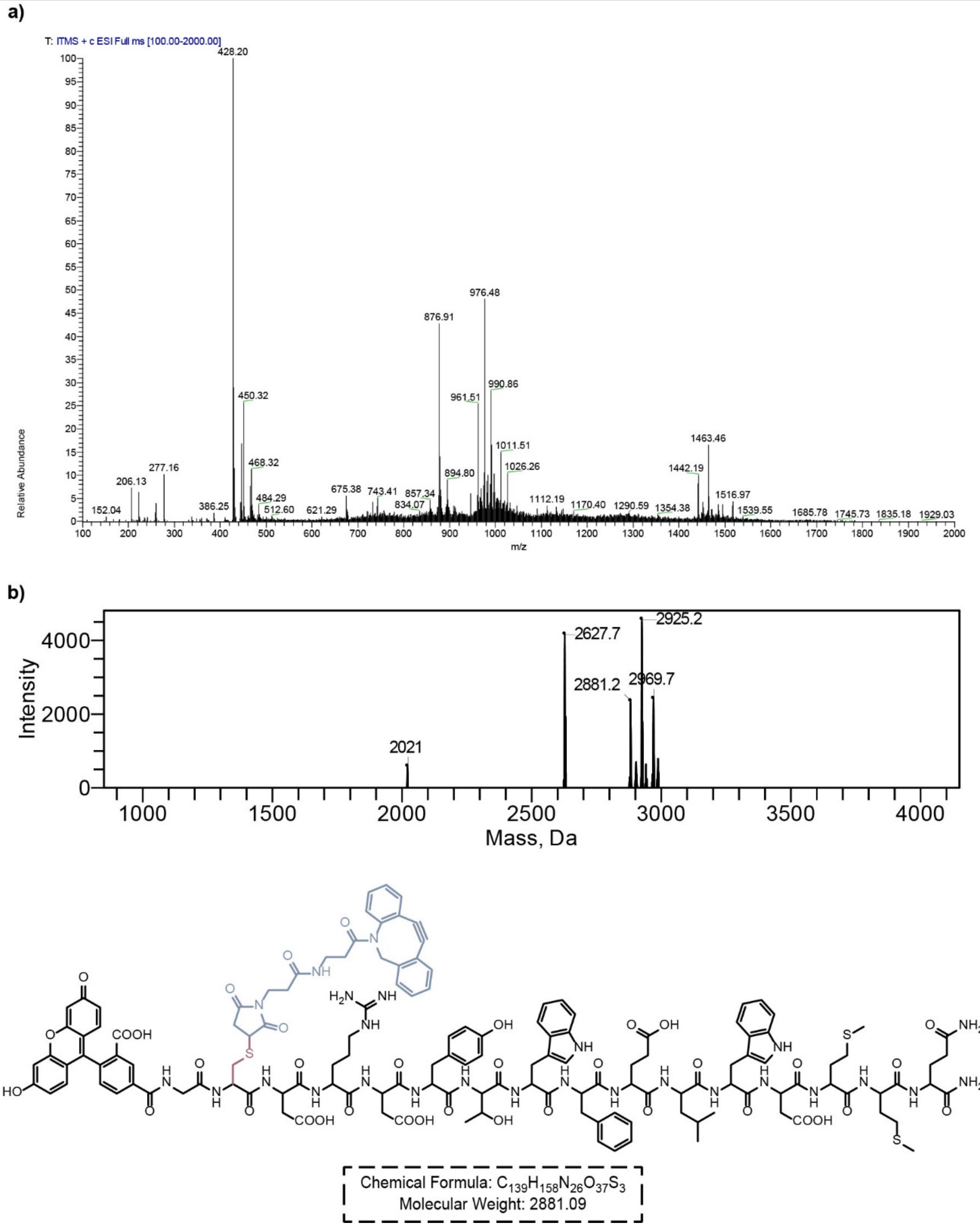

Figure S16. a) Low-resolution mass spectrum of the ESBP-NoK peptide (20 µM) following incubation with maleimide-DBCO (40 µM) in ammonium acetate buffer (20 mM, pH 8.0) for 2 h at 25 ºC and **b)** subsequent deconvolution with structural representation of the major product identified: Peptide ESBP-NoK + one maleimide-DBCO + 45 Da [976.48 (+3), 1463.46 (+2)]. A degradation product was detected presumably due to a hydrolysis of the secondary amide of maleimide-DBCO.

a)

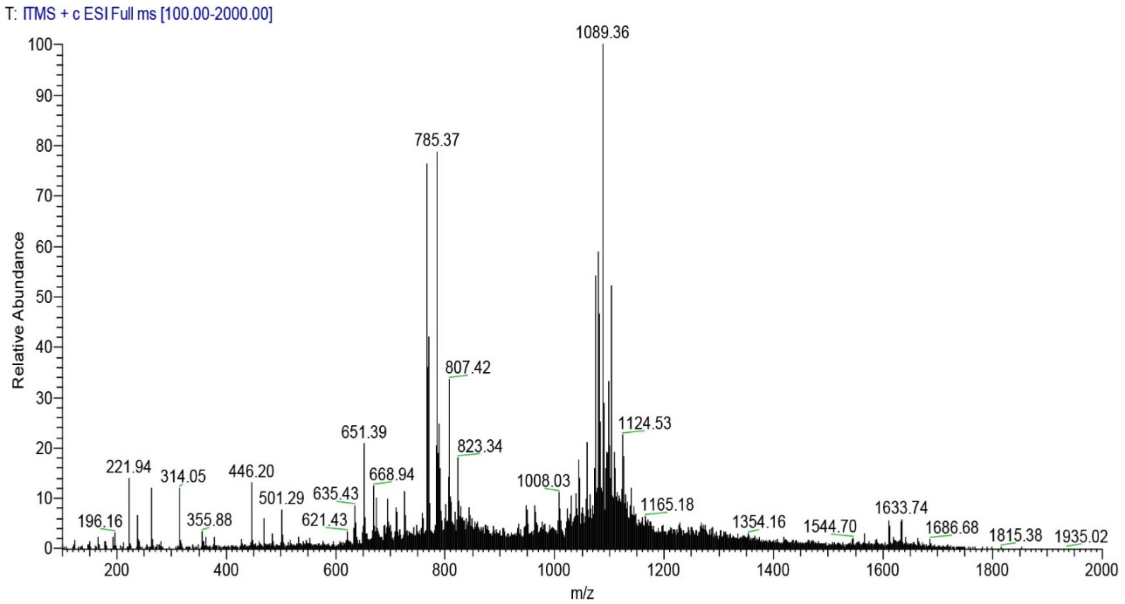

b)

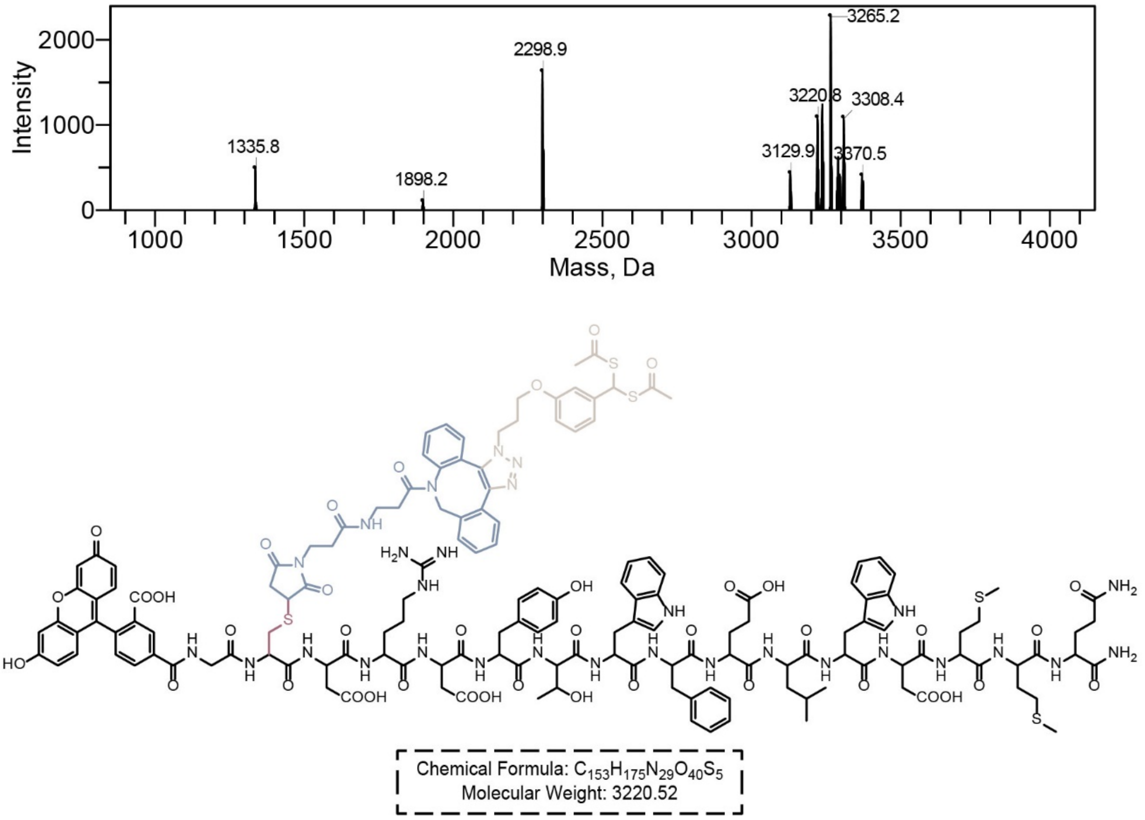

Figure S17. a) Low-resolution mass spectrum of the ESBP-NoK peptide (20 µM) following sequential incubation with maleimide-DBCO (40 µM) and compound **4** (160 µM) in ammonium acetate buffer (20 mM, pH 8.0) for 2 h and 1 h, respectively, at 25 ºC and **b)** subsequent deconvolution with structural representation of the major product identified: Peptide ESBP-NoK + one maleimide-DBCO + one di-acetylated SPAAC product + 45 [1089.36 (+3)].

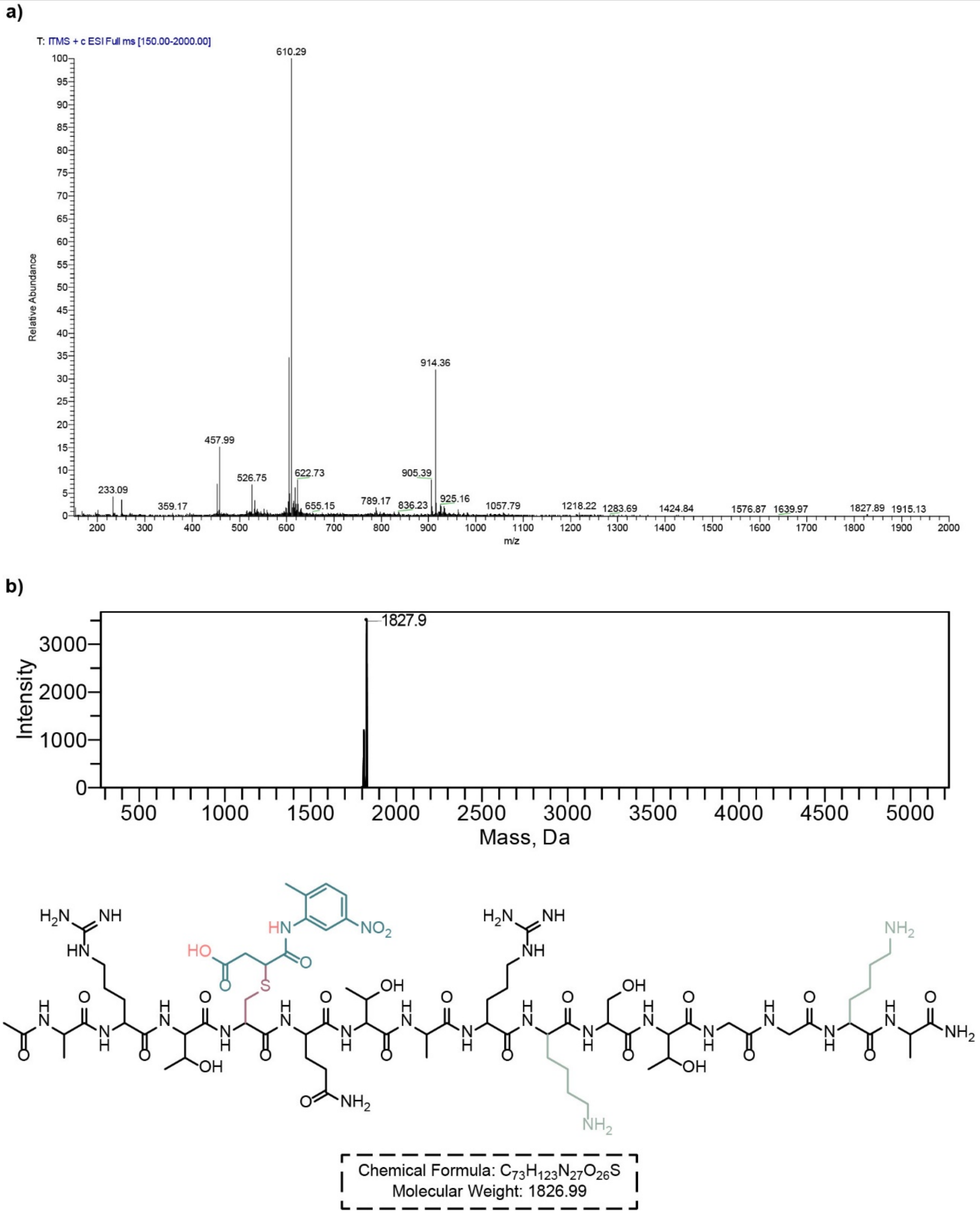

Figure S18. a) Low-resolution mass spectrum of the histone K4C peptide (20 µM) following incubation with the maleimide-dummy (40 µM) in ammonium acetate buffer (20 mM, pH 8.0) for 2 h at 25 ºC and **b)** subsequent deconvolution with structural representation of the major product identified: Peptide K4C + one maleimide-dummy + one hydration [457.99 (+4), 610.29 (+3), 914.36 (+2)] – hydration most likely resulting from maleimide hydrolysis promoted by the strong electron withdrawing nature of the nitro group.

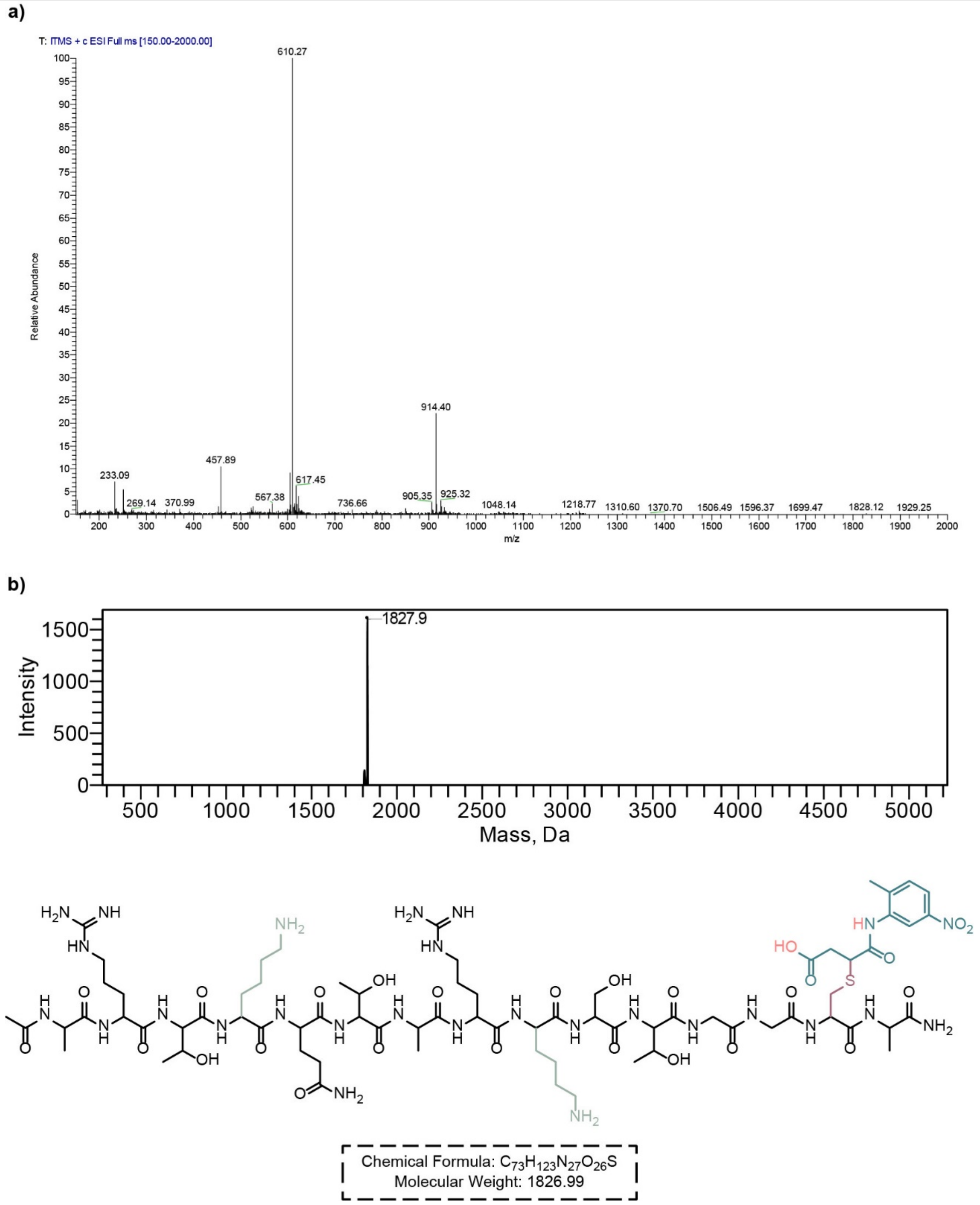

Figure S19. a) Low-resolution mass spectrum of the histone K14C peptide (20 μM) following incubation with maleimide-dummy (40 μM) in ammonium acetate buffer (20 mM, pH 8.0) for 2 h at 25 ºC and **b)** subsequent deconvolution with structural representation of the major product identified: Peptide K14C + one maleimide-dummy + one hydration [610.27 (+3), 914.40 (+2)] – hydration most likely resulting from maleimide hydrolysis promoted by the strong electron withdrawing nature of the nitro group.

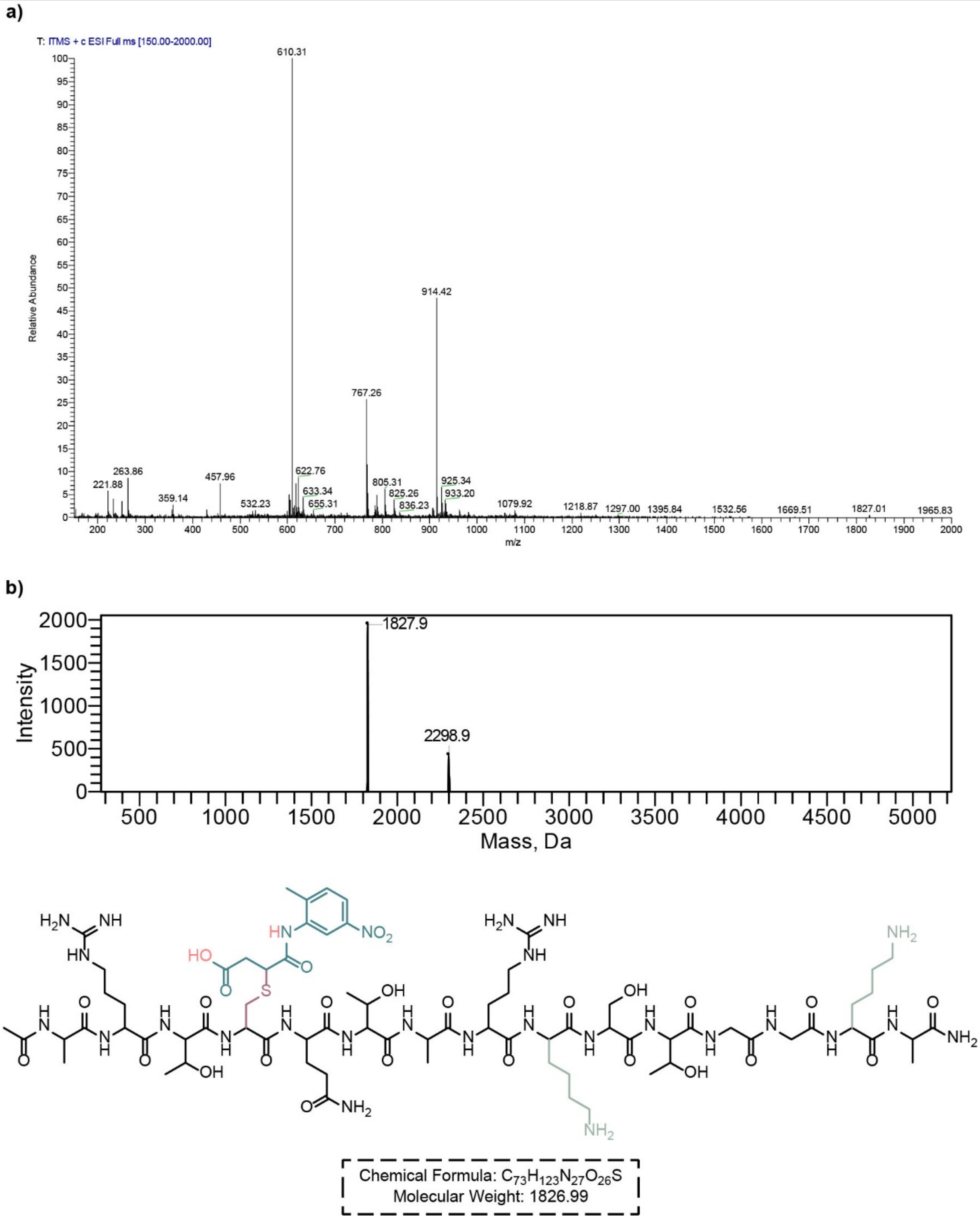

Figure S20. a) Low-resolution mass spectrum of the histone K4C peptide (20 µM) following sequential incubation with maleimide-dummy (40 µM) and the SPAAC product resulting from maleimide-DBCO (80 µM) and compound **4** (320 µM) in ammonium acetate buffer (20 mM, pH 8.0) for 2 h and 2 h, respectively, at 25 ºC and **b)** subsequent deconvolution with structural representation of the major product identified: Peptide K4C + one maleimide-dummy + one hydration [610.31 (+3), 914.42 (+2)] – hydration most likely resulting from maleimide hydrolysis promoted by the strong electron withdrawing nature of the nitro group.

a)

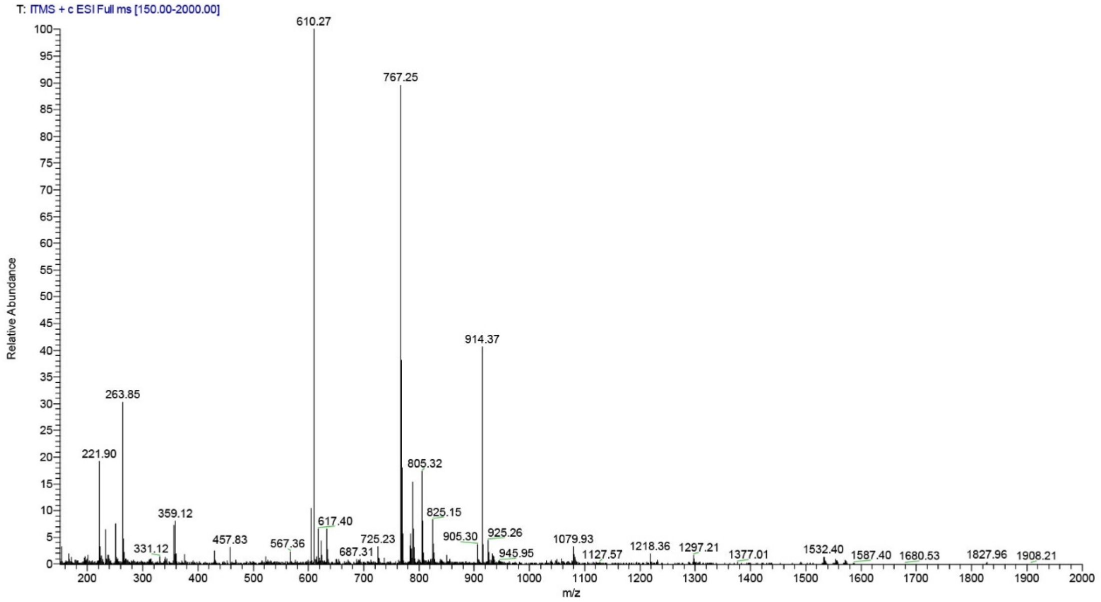

b)

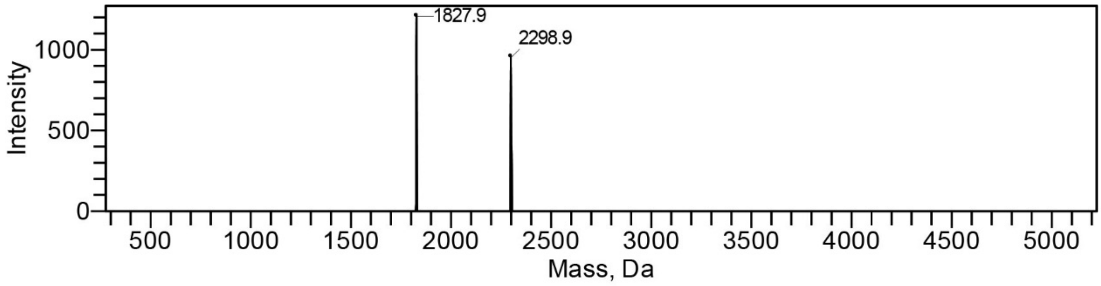

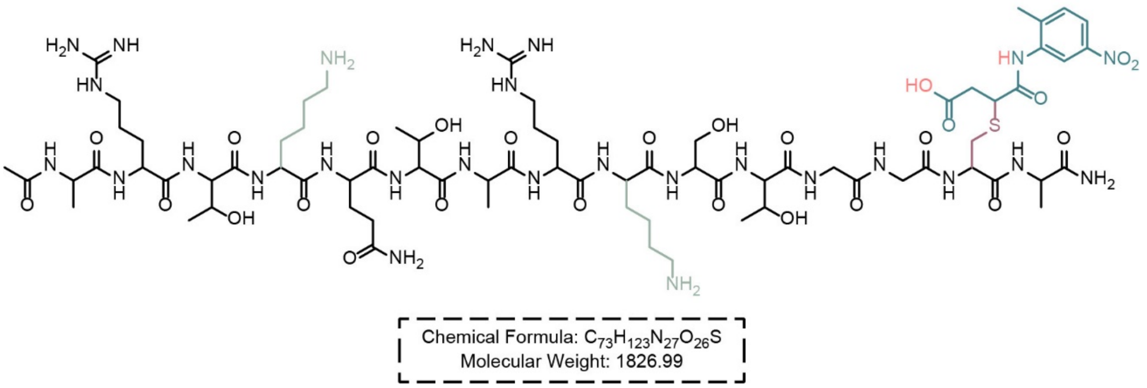

Figure S21. a) Low-resolution mass spectrum of the histone K14C peptide (20 μM) following sequential incubation with maleimide-dummy (40 μM) and the SPAAC product resulting from maleimide-DBCO (80 μM) and compound **4** (320 μM) in ammonium acetate buffer (20 mM, pH 8.0) for 2 h and 2 h, respectively, at 25 ºC and **b)** subsequent deconvolution with structural representation of the major product identified: Peptide K14C + one maleimide-dummy + one hydration [610.27 (+3), 914.37 (+2)] – hydration most likely resulting from maleimide hydrolysis promoted by the strong electron withdrawing nature of the nitro group.

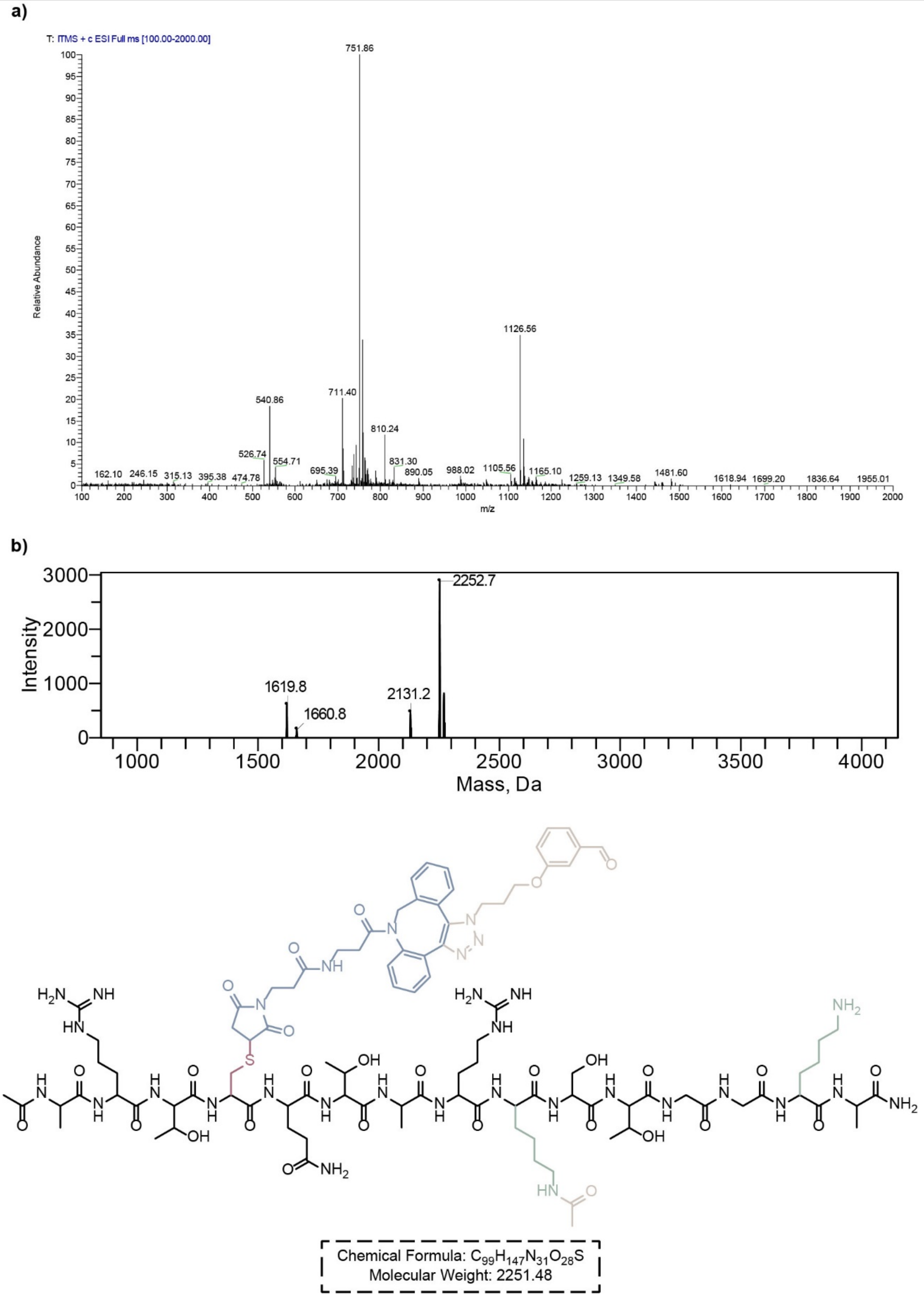

Figure S22. a) Low-resolution mass spectrum of the histone K4C peptide (20 µM) following sequential incubation with maleimide-DBCO (40 µM), compound **4** (160 µM) and *β*-mercaptoethanol (250 µM) in ammonium acetate buffer (20 mM, pH 8.0) for 3 h at 25 ºC and **b)** subsequent deconvolution with structural representation of the major product identified: Peptide K4C + one maleimide-DBCO + one deacetylated SPAAC product + mono-acetylation [751.86 (+3), 1126.56 (+2)].

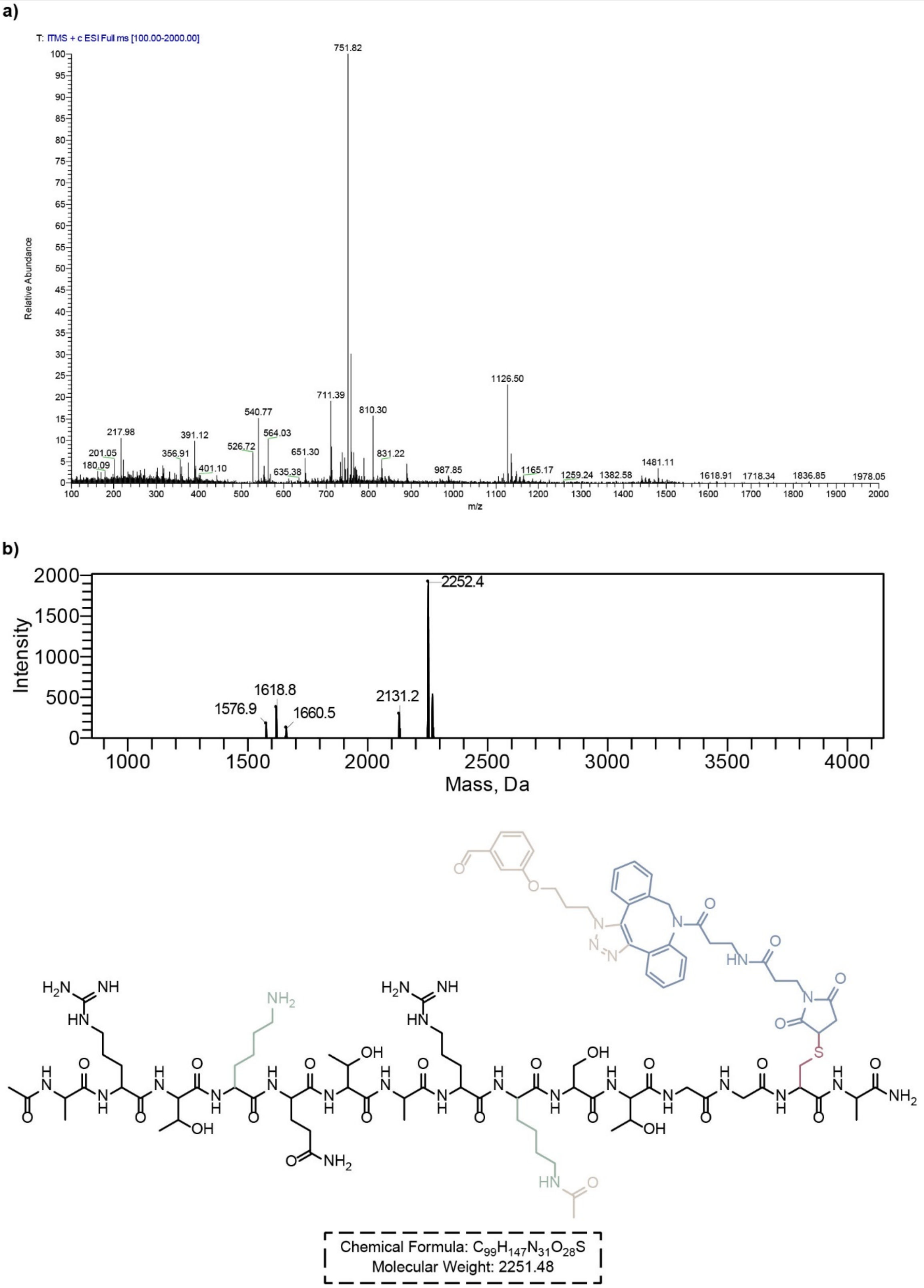

Figure S23. a) Low-resolution mass spectrum of the histone K14C peptide (20 µM) following sequential incubation with maleimide-DBCO (40 µM), compound **4** (160 µM) and *β*-mercaptoethanol (250 µM) in ammonium acetate buffer (20 mM, pH 8.0) for 4 h at 25 ºC and **b)** subsequent deconvolution with structural representation of the major product identified: Peptide K14C + one maleimide-DBCO + one deacetylated SPAAC product + mono-acetylation [751.82 (+3), 1126.50 (+2)].

a)

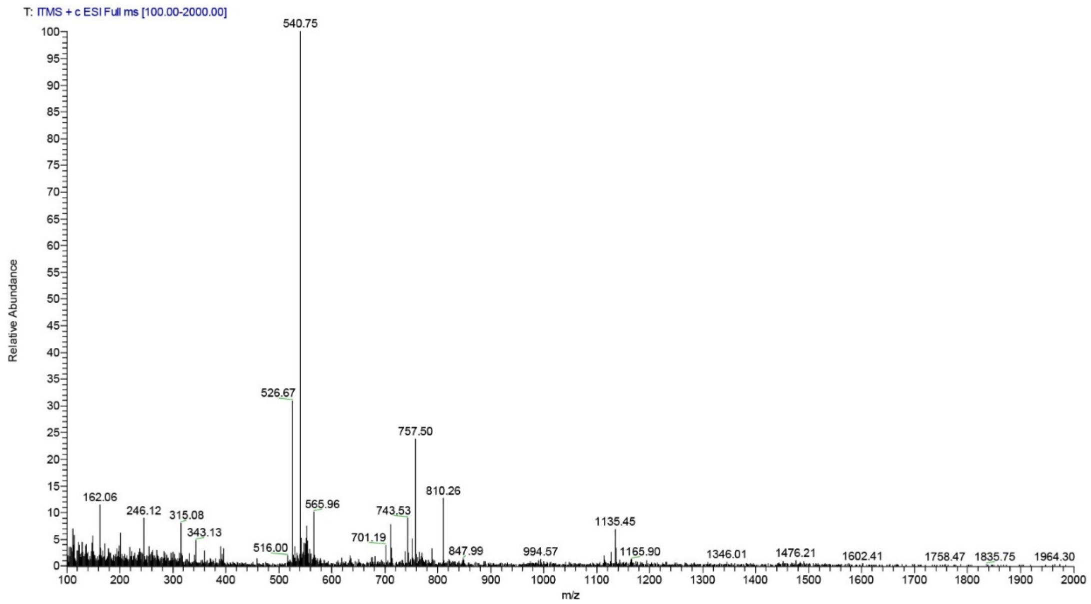

b)

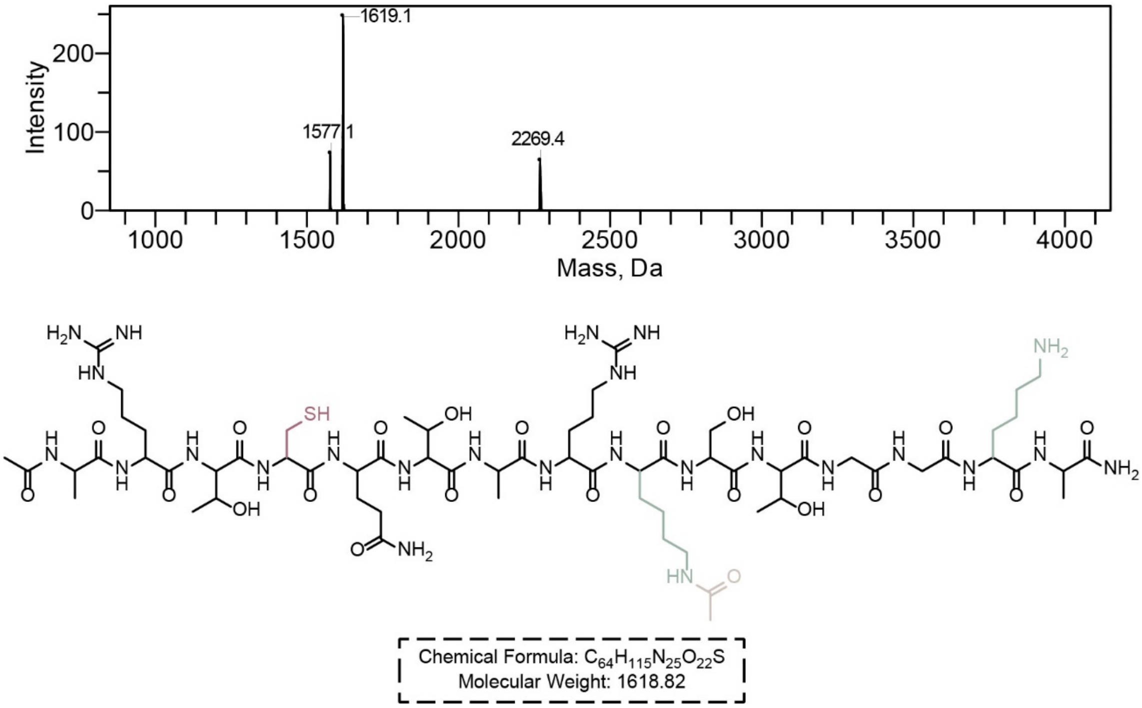

Figure S24. a) Low-resolution mass spectrum of the histone K4C peptide (20 µM) following sequential incubation with maleimide-DBCO (40 µM), compound **4** (160 µM) and *β*-mercaptoethanol (250 µM) in ammonium acetate buffer (20 mM, pH 8.0) for 23 h at 25 ºC and **b)** subsequent deconvolution with structural representation of the major product identified: Peptide K4C + mono-acetylation [540.75 (+3), 810.26 (+2)]. Due to the prolonged reaction time, intramolecular rearrangements could have occurred and instead of an acetylated amine, one would detect an acetylated cysteine thiol (considering that the unmodified peptide K4C is also detected).

a)

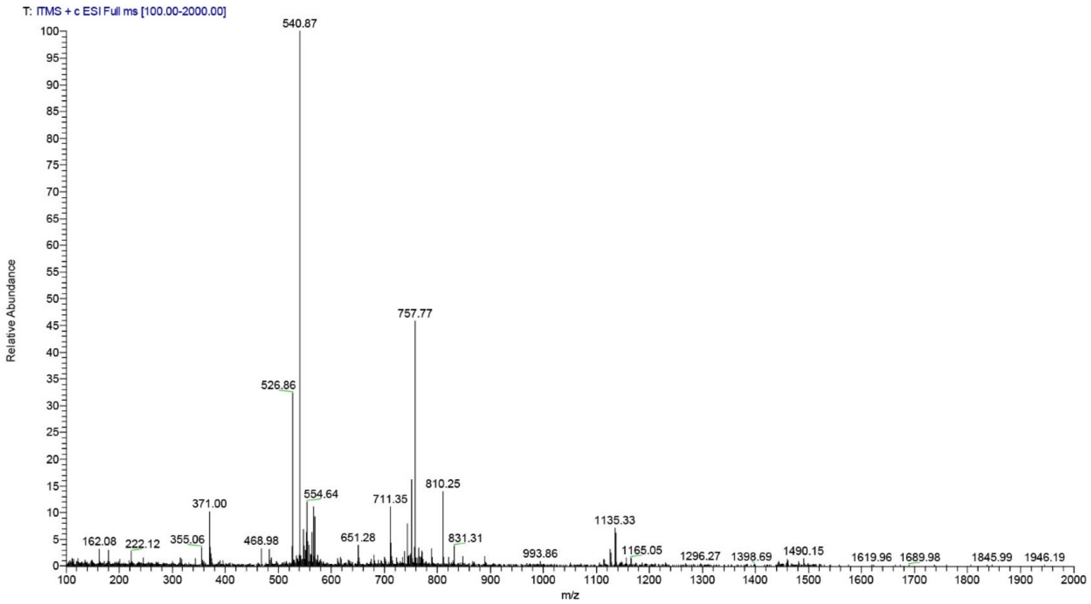

b)

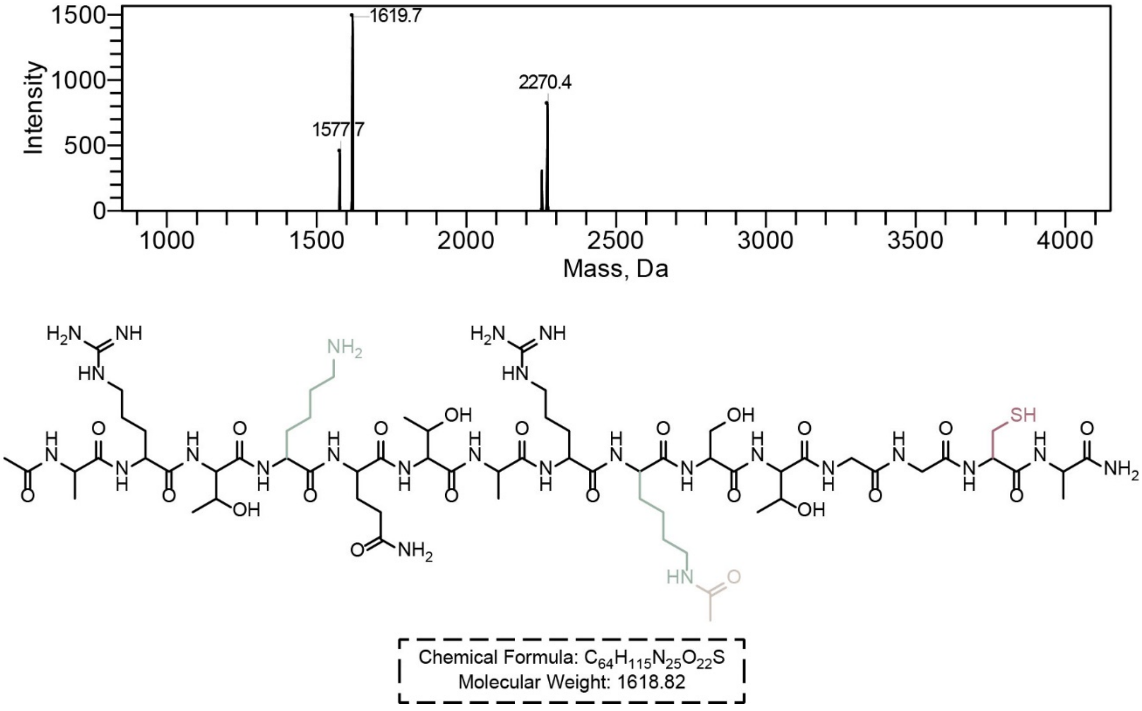

Figure S25. a) Low-resolution mass spectrum of the histone K14C peptide (20 µM) following sequential incubation with maleimide-DBCO (40 µM), compound **4** (160 µM) and *β*-mercaptoethanol (250 µM) in ammonium acetate buffer (20 mM, pH 8.0) for 23 h at 25 ºC and **b)** subsequent deconvolution with structural representation of the major product identified: Peptide K14C + mono-acetylation [540.87 (+3), 810.25 (+2)]. Due to the prolonged reaction time, intramolecular rearrangements could have occurred and instead of an acetylated amine, one would detect an acetylated cysteine thiol (considering that the unmodified peptide K14C is also detected).

a)

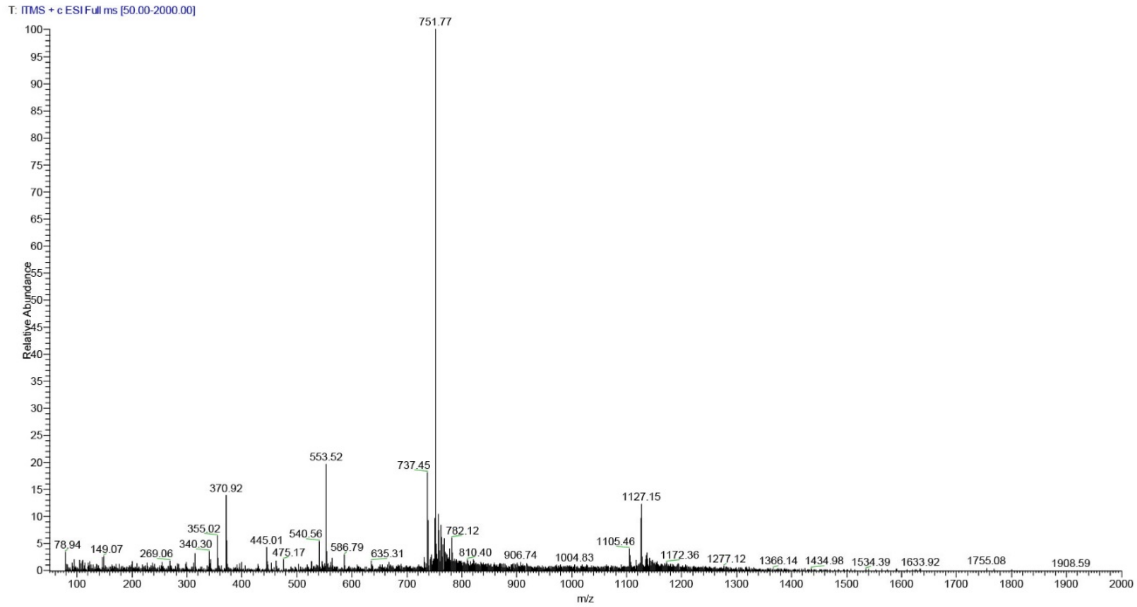

b)

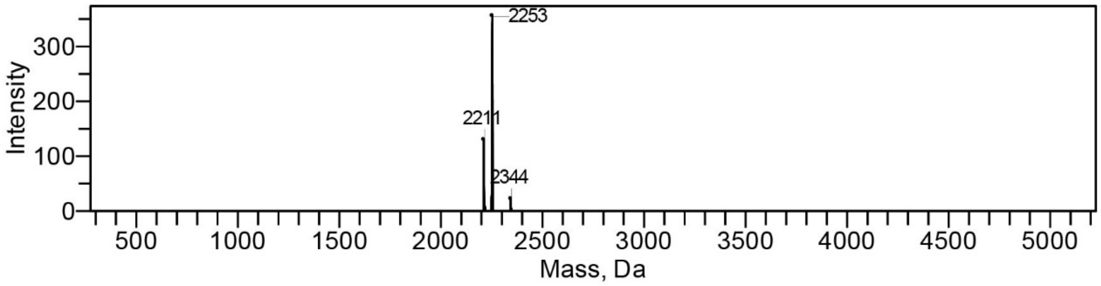

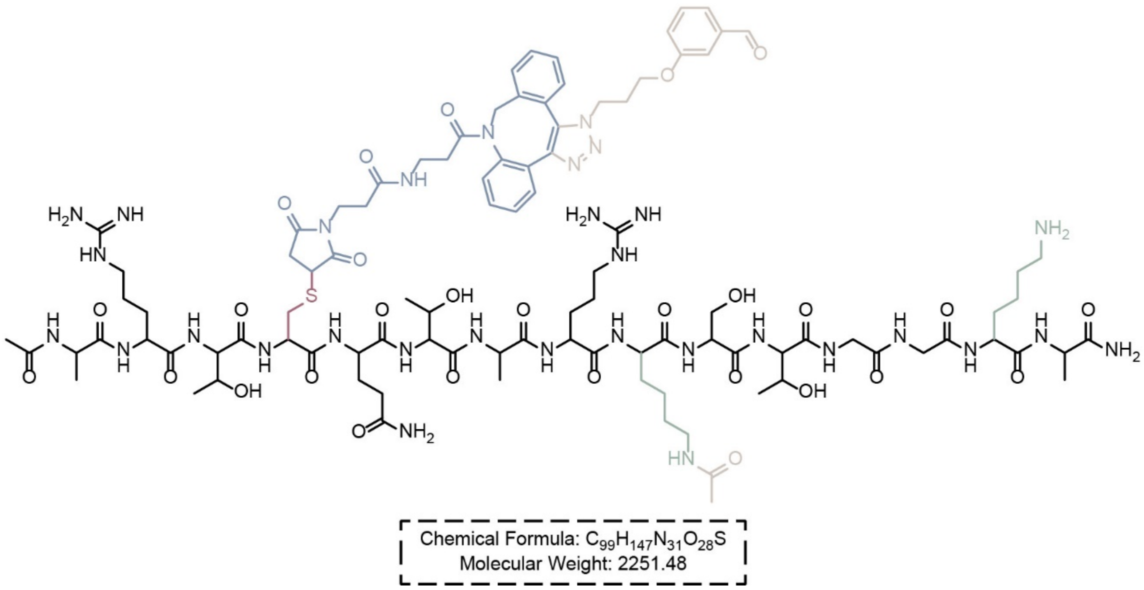

Figure S26. a) Low resolution mass spectrum of the histone K4C peptide (20 µM) following sequential incubation with maleimide-DBCO (40 µM), compound **4** (160 µM) and Sirt6 (30 µg/mL) in ammonium acetate buffer (20 mM, pH 8.0) for 3 h at 25 ºC and **b)** subsequent deconvolution with structural representation of the major product identified: Peptide K4C + one maleimide-DBCO + one deacetylated SPAAC product + mono-acetylation [751.77 (+3), 1127.15 (+2)]. Sirt6-deacetylated product: Peptide K4C + one maleimide-DBCO + one deacetylated SPAAC product [737.45 (+3), 1105.46 (+2)].

a)

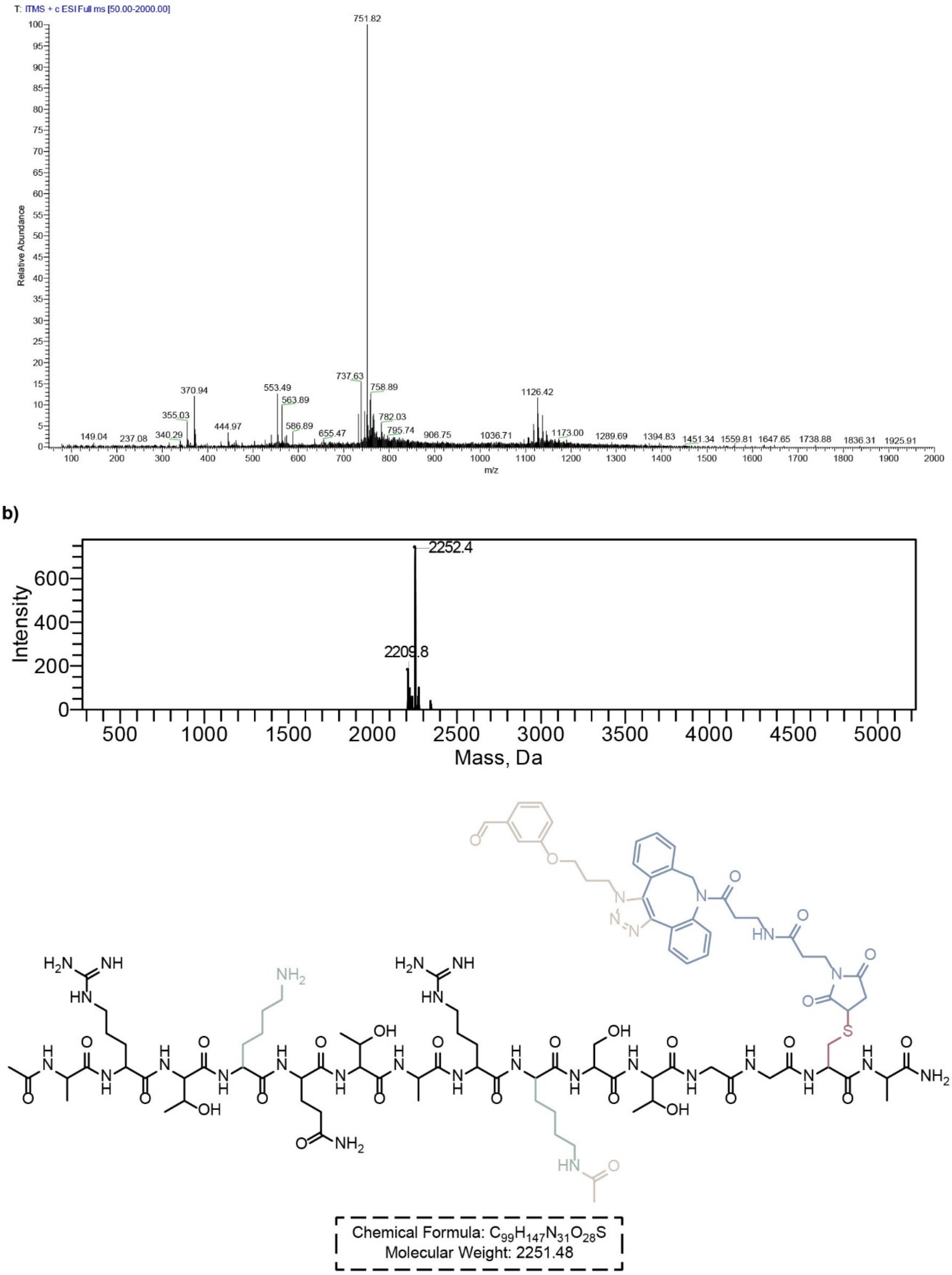

Figure S27. a) Low-resolution mass spectrum of the histone K14C peptide (20 µM) following sequential incubation with maleimide-DBCO (40 µM), compound **4** (160 µM) and Sirt6 (30 µg/mL) in ammonium acetate buffer (20 mM, pH 8.0) for 3 h at 25 ºC and **b)** subsequent deconvolution with structural representation of the major product identified: Peptide K14C + one maleimide-DBCO + one deacetylated SPAAC product + mono-acetylation [751.82 (+3), 1126.42 (+2)]. Sirt6-deacetylated product: Peptide K14C + one maleimide-DBCO + one deacetylated SPAAC product [737.63 (+3)].

4. Expression and purification of mutant histone proteins

The pET-3a plasmid encoding histone H3 (C110A) from *Xenopus laevis* was a kind gift of Professor Ernest Laue (Department of Biochemistry, University of Cambridge). The H3K4C, H3R52C and H3K4CR52C mutants were constructed by site-directed mutagenesis using the NZYMutagenesis kit (NZYTech) according to the manufacturer's instructions. Primers were obtained from Sigma and the mutations were confirmed by sequencing. The following primer sequences were used to introduce the K4C and R52C mutations in the pET-3a-H3 plasmid by PCR:

<u>K4C</u>
5'- ATGGCCCGTACCTGCCAGACCGCCCGT-3'
5'- ACGGGCGGTCTGGCAGGTACGGGCCAT-3'

<u>R52C</u>
5'-CTCTCCGCGAGATCTGCCGCTACCAGAAA-3'
5'-TTTCTGGTAGCGGCAGATCTCGCGGAG-3'

The mutant H3K4C, H3R52C and H3K4CR52C proteins were recombinantly expressed in *E. coli BL21(DE3)* competent cells (NZYTech) following bacterial transformation with their respective expression plasmids. Cells were grown at 37 ºC and 220 rpm in LB containing carbenicillin (100 µg/mL) until the optical density reached 0.5-0.7. Protein expression was then induced with 1 mM IPTG for 2 h at 37 ºC and 220 rpm. Cells were harvested by centrifugation at 7000 rpm and 4 ºC for 10 min and stored at -80 ºC. Protein expression was confirmed by SDS-PAGE.
Histone H3K4C, H3R52C and H3K4CR52C mutants were purified by anion and cation exchange chromatography in tandem, essentially as described in the literature.[2] Briefly, the bacterial pellet was resuspended in SAU buffer (40 mM NaOAc pH 5.2, 7 M urea, 10 mM lysine, 1 mM EDTA pH 8.0, 5 mM β-mercaptoethanol) containing 200 mM NaCl, protease inhibitors and DNase. Defined buffer conditions were achieved by directly adding to the pellet DNase I (Roche) and protease inhibitors (Roche) in powder, followed by the addition of 10x SAU buffer (400 mM NaOAc pH 5.2, 10 mM EDTA pH 8.0, 100 mM lysine), *β*-mercaptoethanol and NaCl to a final concentration of 5 mM and 200 mM, respectively. Once the cells were resuspended, urea was added to a concentration of 7M and the suspension filled up to its final volume with water. All steps during lysis were performed on ice.
The suspension was sonicated on ice with an amplitude of 20% using 20 pulses of 15 seconds, each followed by a pause of 30 seconds. The extract was cleared by centrifugation at 40000g and 4 ºC for 30 min. The resulting supernatant was filtered and loaded into a HiTrap Q HP column (GE Healthcare, 5 mL) stacked on top of a HiTrap SP HP column (GE Healthcare, 1 mL) pre-equilibrated with SAU-200 and connected to an FLPC system (ÄKTA, GE Healthcare). When the extract had passed completely through the Q column, the latter was removed from the FPLC system and the SP column washed with 200 mM NaCl for several column volumes. Histones were then eluted with a NaCl gradient. Histone-containing fractions were pooled, buffer-exchanged to Tris (15 mM, pH 7.5) with Amicon Ultra-15 mL centrifugal filter units (Merck Millipore) and lyophilized. Representative ESI-MS spectra of the purified H3K4C (calculated mass = 15214 Da; observed mass = 15219 Da), H3R52C (calculated mass = 15186 Da; observed mass = 15186 Da) and H3K4CR52C (calculated mass = 15161 Da; observed mass = 15165 Da) proteins at a concentration of 25 µM are shown in Figures S28, S29 and S30, respectively.

Histone H3K4C (C110A)

ART<u>**C**</u>QTARKSTGGKAPRKQLATKAARKSAPATGGVKKPHRYRPGTVALREIRRYQKSTELLIRKLPFQRLVREIAQDFKTDLRFQSSAV
MALQEASEAYLVALFEDTNLAAIHAKRVTIMPKDIQLARRIRGERA

Histone H3R52C (C110A)

ARTKQTARKSTGGKAPRKQLATKAARKSAPATGGVKKPHRYRPGTVALREI<u>**C**</u>RYQKSTELLIRKLPFQRLVREIAQDFKTDLRFQSSAV
MALQEASEAYLVALFEDTNLAAIHAKRVTIMPKDIQLARRIRGERA

Histone H3K4CR52C (C110A)

ART<u>**C**</u>QTARKSTGGKAPRKQLATKAARKSAPATGGVKKPHRYRPGTVALREI<u>**C**</u>RYQKSTELLIRKLPFQRLVREIAQDFKTDLRFQSSAV
MALQEASEAYLVALFEDTNLAAIHAKRVTIMPKDIQLARRIRGERA

5. Conjugation experiments in histone proteins

Stock solutions of maleimide-DBCO (2.5 mM), maleimide-dummy (2.5 mM), compound **2** (1 mM and 2 mM), compound **4** (1 mM and 2 mM) and Ellman's reagent (2.5 mM) were freshly prepared in anhydrous DMF prior to use.

General procedure to generate acetylated histone proteins

Preparation of histone H3K4C, H3R52C and H3K4CR52C samples for conjugation reactions

The preparation of histone samples for subsequent chemical modification was based on the work described in the literature.[3] Following lyophilization, approximately 2 to 4 mg of histones were dissolved in 400 µL of milli-Q water / NH_4CH_3COO (20 mM, pH 7.0) and treated with a slight excess of TCEP for 10 to 30 min at 21 ºC to reduce any contaminant disulfide. The reduced sample was then passed through a PD MiniTrap G25 column (Cytiva) and eluted with 1.0 mL of milli-Q water. The resulting protein sample was kept on ice and quantified by the Bradford assay. Representative ESI-MS spectra obtained for H3K4C (calculated mass = 15214 Da; observed mass = 15218 Da), H3R52C (calculated mass = 15186 Da; observed mass = 15185 Da) and H3K4CR52C (calculated mass = 15161 Da; observed mass = 15167 Da) are shown in Figures S31, S32 and S33.

Conjugation at the free cysteine of H3K4C, H3R52C and H3K4CR52C with the maleimide-DBCO clickable handle

Aliquots of reduced H3K4C, H3R52C and H3K4CR52C were diluted to 25 µM with ammonium acetate buffer (20 mM, pH 7.0). Then, an aliquot of 2.5 eq. of maleimide-DBCO was added to each mixture and the reactions were shaken at 400 rpm and 25 ºC. After 1 h, the reaction mixtures were analysed by LC-MS and complete conversion to the H3K4C (calculated mass = 15641 Da; observed mass = 15639 Da), H3R52C (calculated mass = 15613 Da; observed mass = 15611 Da) and H3K4CR52C (calculated mass = 16016 Da; observed mass = 16022 Da) proteins containing the maleimide-DBCO clickable handle was observed. The resulting ESI-MS spectra are shown in Figures S34, S35 and S36. The independent aliquots of H3K4C-maleimide-DBCO, H3R52C-maleimide-DBCO and H3K4CR52C-maleimide-DBCO were desalted to water and concentrated using Amicon centrifugal filter units (Merck Millipore). Protein concentrations were then determined by the Bradford assay.

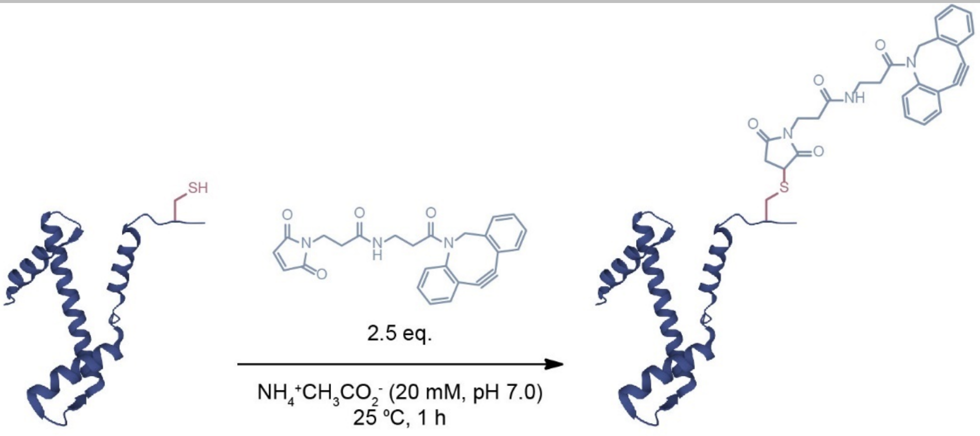

H3K4C
(25 µM)

H3K4C-maleimide-DBCO
(25 µM)
full conversion

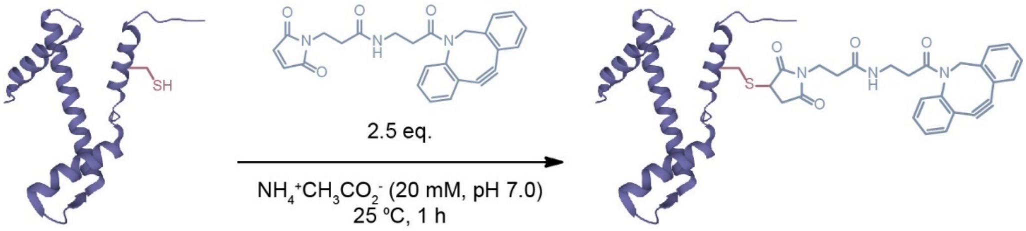

H3R52C
(25 µM)

H3R52C-maleimide-DBCO
(25 µM)
full conversion

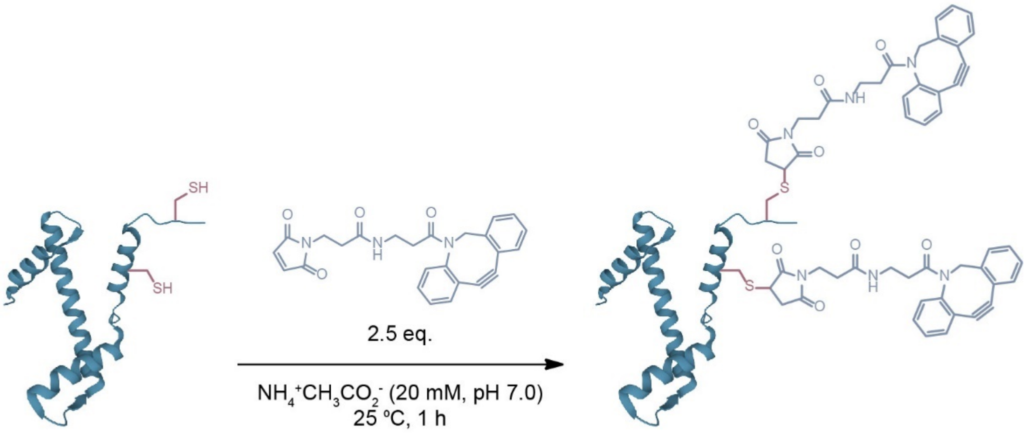

H3K4CR52C
(25 µM)

H3K4CR52C-maleimide-DBCO
(25 µM)
full conversion

Site-specific acetylation of H3K4C, H3R52C and H3K4CR52C following SPAAC reaction with compound **4**

Initially, the SPAAC reaction was performed at a basic pH condition. In this context, independent aliquots of the H3K4C, H3R52C and H3K4CR52C proteins containing the maleimide-DBCO clickable handle were diluted to 25 µM in ammonium acetate buffer (20 mM, pH 8.0). An aliquot of 4 eq. of compound **4** was added to the diluted H3K4C-maleimide-DBCO solution, while an aliquot of 2 eq. of the same compound was added to the solution containing the diluted H3R52C-maleimide-DBCO protein. The reactions were shaken at 400 rpm and 25 °C. After 30 min, the reaction mixtures were analysed by LC-MS and complete conversion to the respective mono-acetylated products was observed (H3K4C**K9Ac: calculated mass = 15889 Da; observed mass = 15890 Da; H3R52C**K56Ac: calculated mass = 15861 Da; observed mass = 15862 Da). An aliquot of 4 eq. of compound 4 was added to the diluted H3K4CR52C-maleimide-DBCO solution and the reaction was shaken at 400 rpm and 25 °C. After 45 min, the reaction mixture was analysed by LC-MS and complete conversion to the respective di-acetylated product was observed (H3K4C**K9AcR52C**K56Ac: calculated mass = 16510 Da; observed mass = 16513 Da). The resulting ESI-MS spectra are shown in Figures S37, S38 and S39. The independent

aliquots of H3K4C**K9Ac, H3R52C**K56Ac and H3K4C**K9AcR52C**K56Ac were desalted and concentrated using Amicon centrifugal filter units (Merck Millipore). Protein concentrations were then determined by the Bradford assay.

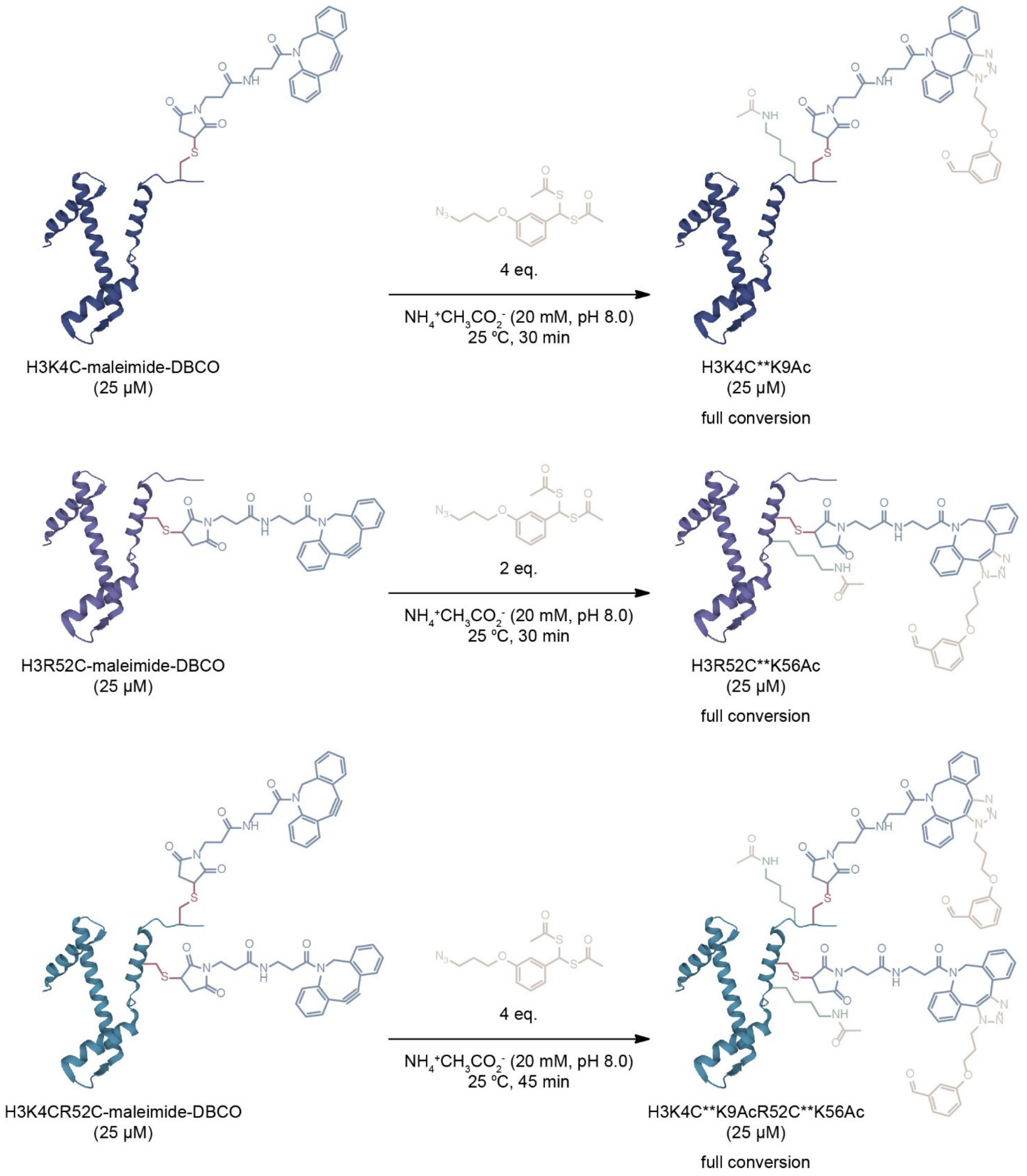

The SPAAC reaction was also performed at an acidic pH. Thus, independent aliquots of the H3K4C, H3R52C and H3K4CR52C proteins containing the maleimide-DBCO clickable handle were instead diluted to 25 µM in ammonium acetate buffer (20 mM, pH 4.9). An aliquot of 4 eq. of compound **4** was added to the diluted H3K4C-maleimide-DBCO and H3K4CR52C-maleimide-DBCO solutions, while an aliquot of 2 eq. of the same compound was added to the solution containing the diluted H3R52C-maleimide-DBCO protein. The reactions were shaken at 400 rpm and 25 ºC. After 30 min, the reaction mixtures were analysed by LC-MS and complete conversion to the respective unacetylated H3K4C, H3R52C and H3K4CR52C SPAAC products (with the acetyl donor still left to react) was observed (unAcH3K4C**: calculated mass = 15981 Da; observed mass = 15983 Da; unAcH3R52C**: calculated mass = 15953 Da;

observed mass = 15956 Da; unAcH3K4C**R52C**: calculated mass = 16695 Da; observed mass = 16699 Da). The resulting ESI-MS spectra are shown in Figures S40, S41 and S42.

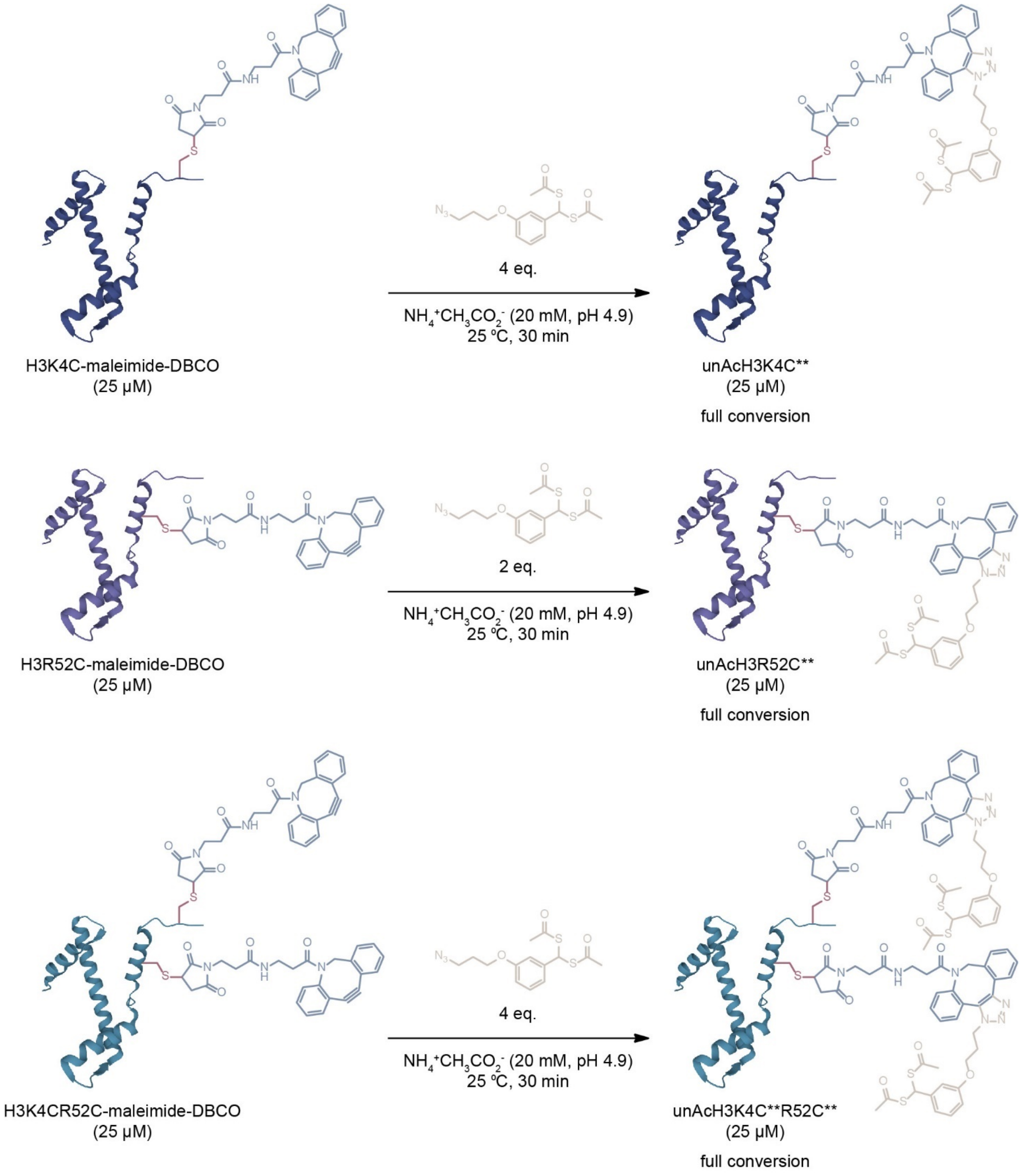

At this point, two approaches were pursued to promote acetylation at K9. In the first approach, an aliquot of unAcH3K4C** was progressively buffer exchanged to ammonium acetate buffer at a basic pH (20 mM, pH 8.0) and then concentrated using Amicon centrifugal filter units (Merck Millipore). Protein concentration was then determined by the Bradford assay and conversion to the corresponding acetylated product was complete as monitored by LC-MS. The resulting ESI-MS spectra of the H3K4C**K9Ac protein is shown in Figure S43 (H3K4C**K9Ac: calculated mass = 15889 Da; observed mass = 15882 Da).

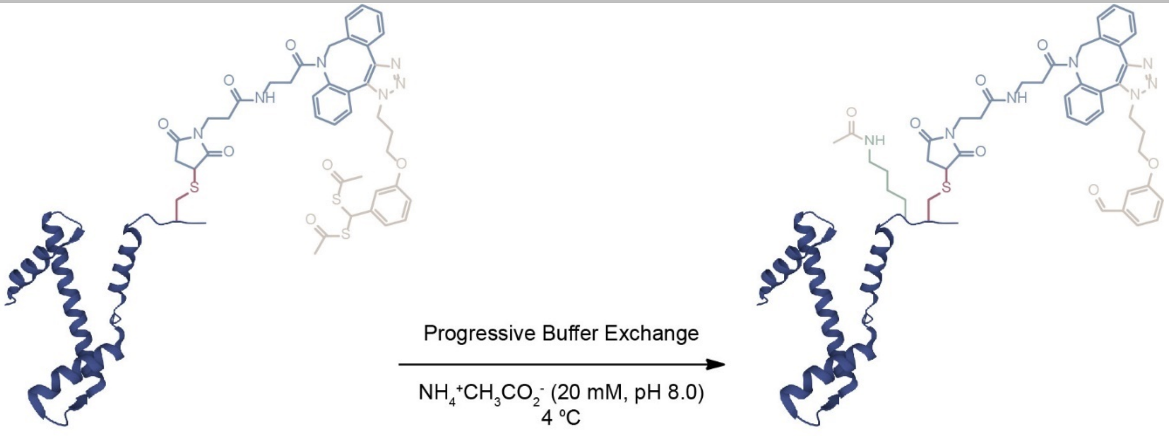

unAcH3K4C** (25 µM) → H3K4C**K9Ac (25 µM) full conversion

In the second approach, which was also used to promote acetylation at K56 in addition to K9, independent aliquots of unAcH3K4C**, unAcH3R52C** and unAcH3K4C**R52C** were buffer exchanged to ammonium acetate buffer at a slightly less acidic pH (20 mM, pH 5.8) and concentrated using Amicon centrifugal filter units (Merck Millipore). Protein concentrations were then determined by the Bradford assay. The unAcH3K4C**, unAcH3R52C** and unAcH3K4C**R52C** protein samples were placed on ice for three to four days until the spontaneous conversion to their corresponding acetylated products was complete as monitored by LC-MS. The resulting ESI-MS spectra of the H3K4C**K9Ac, H3R52C**K56Ac and H3K4C**K9AcR52C**K56Ac proteins are shown in Figures S44, S45 and S46, respectively (H3K4C**K9Ac: calculated mass = 15889 Da; observed mass = 15882 Da; H3R52C**K56Ac: calculated mass = 15861 Da; observed mass = 15862 Da; H3K4C**K9AcR52C**K56Ac: calculated mass = 16510 Da; observed mass = 16513 Da). The acetylated proteins were then desalted to water, quantified by the Bradford assay and evaluated by LC-MS. The resulting ESI-MS spectra are shown in Figures S47, S48 and S49 (H3K4C**K9Ac: calculated mass = 15889 Da; observed mass = 15889 Da; H3R52C**K56Ac: calculated mass = 15861 Da; observed mass = 15862 Da; H3K4C**K9AcR52C**K56Ac: calculated mass = 16510 Da; observed mass = 16513 Da). Finally, the aliquots of H3K4C**K9Ac, H3R52C**K56Ac and H3K4C**K9AcR52C**K56Ac were lyophilized and frozen at -80 ºC for further studies.

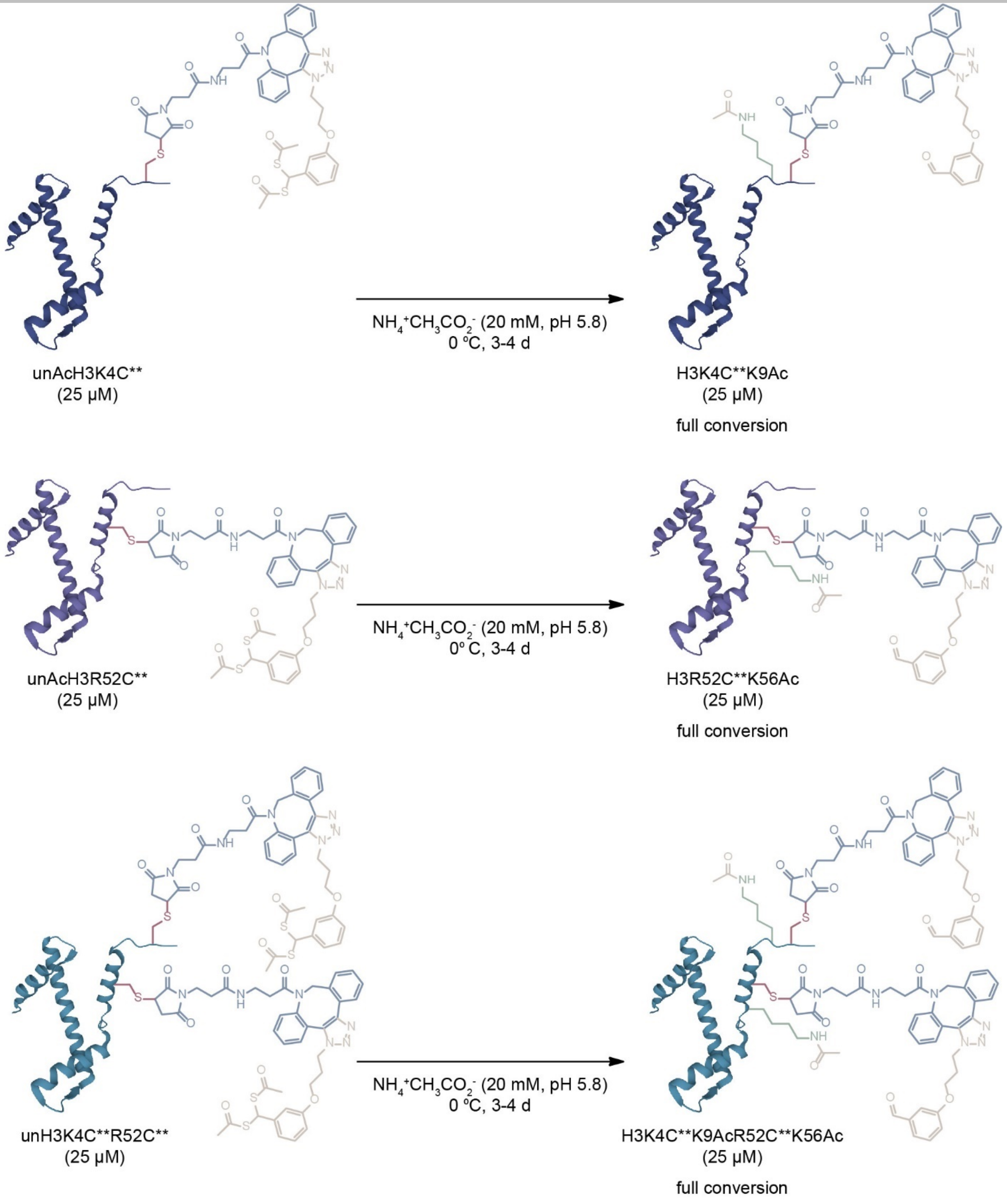

Control reactions (1) of the H3K4C, H3R52C and H3K4CR52C proteins with a maleimide-dummy

To demonstrate that the SPAAC reaction is essential in promoting acetylation at the nearest lysine residue, the H3K4C, H3R52C and H3K4CR52C proteins were also modified with the maleimide-dummy. For this purpose, aliquots of histones H3K4C, H3R52C and H3K4CR52C were diluted to 25 µM with ammonium acetate buffer (20 mM, pH 7.0). An aliquot of 2.5 eq. of maleimide-dummy was added to each mixture and the reactions were shaken at 400 rpm and 25 ºC. After 1 h, the reaction mixtures were analysed by LC-MS and complete conversion to the H3K4C, H3R52C and H3K4CR5C proteins containing the maleimide-dummy was observed (H3K4C-maleimide-dummy: calculated mass = 15446 Da; observed mass = 15450 Da; H3R52C-maleimide-dummy: calculated mass = 15418 Da; observed mass = 15415 Da; H3K4CR52C-maleimide-dummy: calculated mass = 15625 Da; observed mass = 15632 Da). The resulting ESI-MS spectra are shown in Figures S50, S51 and S52. The independent aliquots of H3K4C-maleimide-dummy, H3R52C-

maleimide-dummy and H3K4CR52C-maleimide-dummy were desalted and concentrated using Amicon centrifugal filter units (Merck Millipore). Protein concentrations were then determined by the Bradford assay.

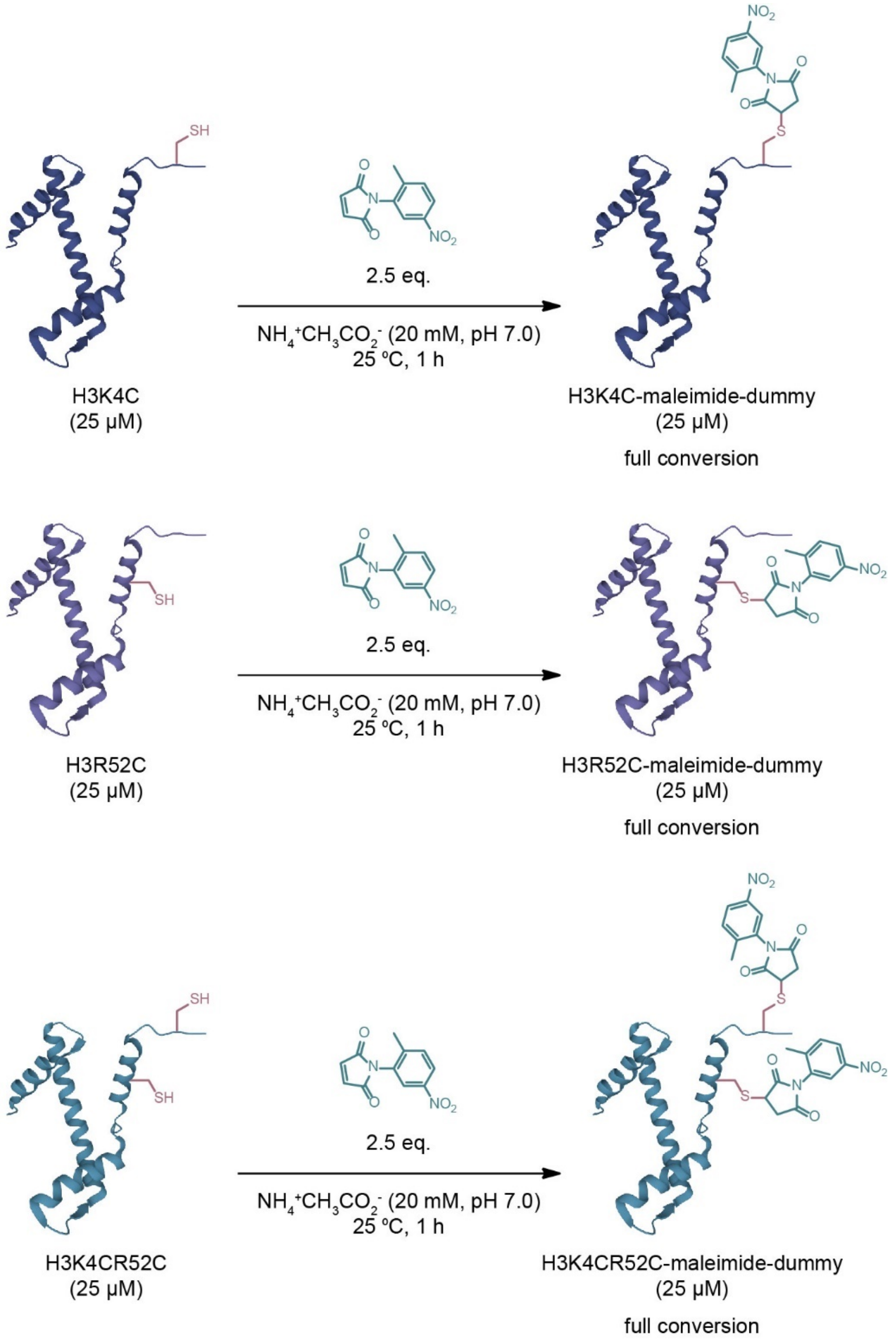

Subsequently, we incubated these modified proteins with compound **4** at a basic pH using the same reaction parameters described previously for the SPAAC reaction and searched for the presence of acetylation. Thus, the independent aliquots of H3K4C-maleimide-dummy, H3R52C-maleimide-dummy and H3K4CR52C-maleimide-dummy were diluted to 25 µM in ammonium acetate buffer (20 mM, pH 8.0). The resulting ESI-MS spectra of the diluted H3K4C-maleimide-dummy, H3R52C-maleimide-dummy and H3K4CR52C-maleimide-dummy proteins in ammonium acetate buffer (20 mM, pH 8.0) are shown in Figures S53, S54 and S55, respectively. The mass difference observed in these spectra compared to those in Figures S50, S51 and S52 likely results from maleimide hydrolysis promoted by the strong electron withdrawing nature of the nitro group (H3K4C-hydrolysed-maleimide-dummy: calculated mass = 15464 Da; observed mass = 15458 Da; H3R52C-hydrolysed-maleimide-dummy: calculated = 15436 Da; observed = 15430 Da; H3K4CR52C-hydrolysed-maleimide-dummy: calculated mass = 15661 Da; observed mass = 15663 Da).

An aliquot of 4 eq. of compound **4** was added to the diluted H3K4C-maleimide-dummy solution, while an aliquot of 2 eq. of the same compound was added to the solution containing the diluted H3R52C-maleimide-dummy protein. The reactions were shaken at 400 rpm and 25 ºC. After 30 min, the reaction mixtures were analysed by LC-MS and acetylation was not detected in any of the two modified

proteins (H3K4C-hydrolysed-maleimide-dummy: calculated mass = 15464 Da; observed mass = 15458 Da; H3R52C-hydrolysed-maleimide-dummy: calculated mass = 15436 Da; observed mass = 15430 Da). An aliquot of 4 eq. of compound **4** was added to the diluted H3K4CR52C-maleimide-dummy solution and the reaction was shaken at 400 rpm and 25 ºC. After 45 min, the reaction mixture was analysed by LC-MS and acetylation was also not observed (H3K4CR52C-hydrolysed-maleimide-dummy: calculated mass = 15661 Da; observed mass = 15664 Da). The resulting ESI-MS spectra for the H3K4C-hydrolysed-maleimide-dummy, H3R52C-hydrolysed-maleimide-dummy and H3K4CR52C-hydrolysed-maleimide-dummy proteins are shown in Figures S56, S57 and S58, respectively.

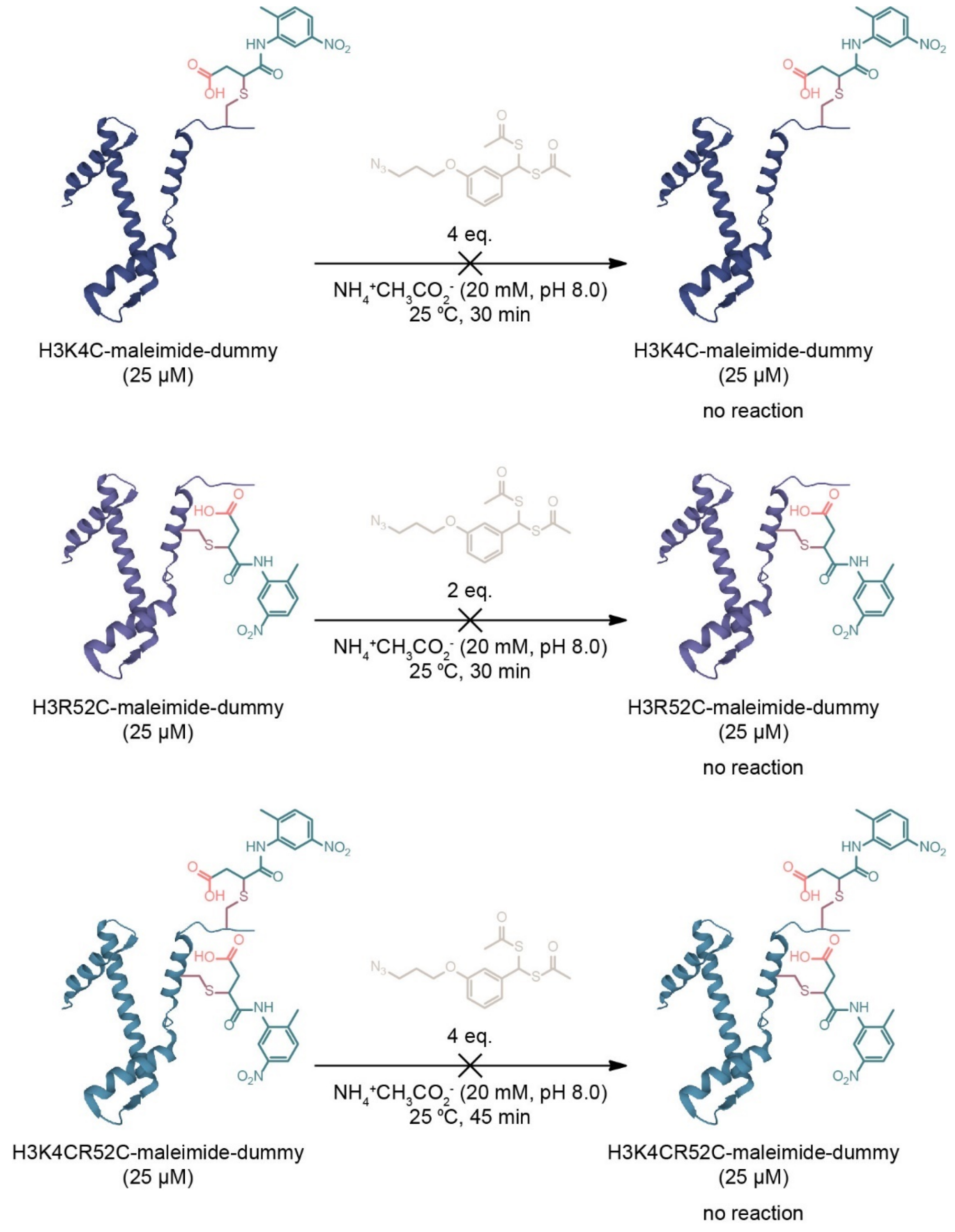

Control reactions (2) with Ellman's reagent to confirm cysteine availability in the H3K4C, H3R52C and H3K4CR52C proteins before and after maleimide conjugation

Evaluation of cysteine availability before maleimide conjugation

The H3K4C, H3R52C and H3K4CR52C proteins were incubated with Ellman's reagent to establish that one (in the case of H3K4C and H3R52C) or two (in the case of H3K4CR52C) free cysteines were present and available for chemical conjugation. For this purpose, independent aliquots of reduced H3K4C, H3R52C and H3K4CR52C were diluted to 25 μM with ammonium acetate buffer (20 mM, pH 8.0). Then, 10 eq. of Ellman's reagent was added to each mixture and the reactions were shaken at 400 rpm and 25 ºC. After 30 min, the reaction mixtures were analysed by LC-MS and complete conversion to the respective Ellman disulfides of H3K4C (calculated mass

= 15411 Da; observed mass = 15409 Da), H3R52C (calculated mass = 15383 Da; observed mass = 15387 Da) and H3K4CR52C (calculated mass = 15555 Da; observed mass = 15560 Da) was observed. The resulting ESI-MS spectra are shown in Figures S59, S60 and S61.

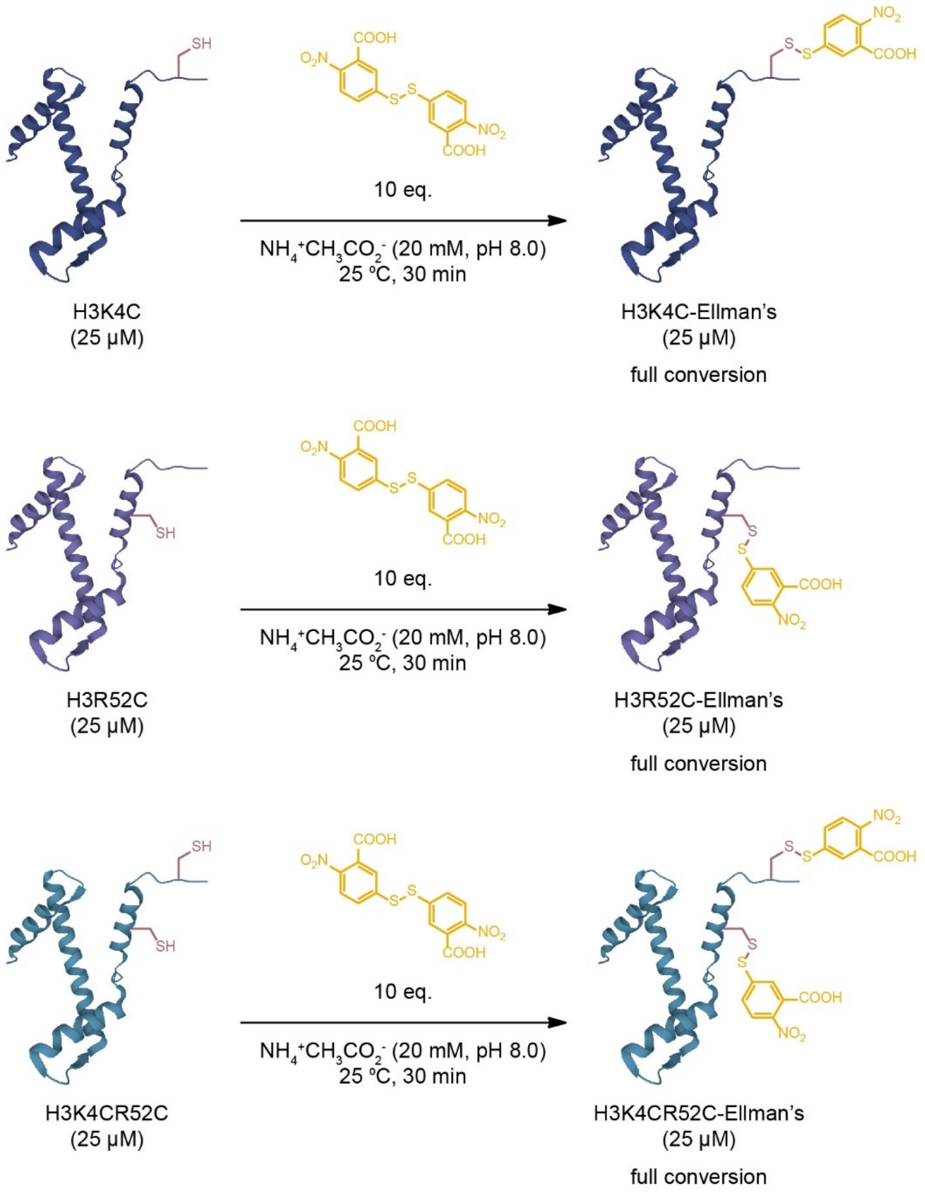

Evaluation of cysteine availability after maleimide conjugation

The H3K4C, H3R52C and H3K4CR52C proteins bearing the two maleimides described in this work (maleimide-DBCO and maleimide-dummy) were incubated with Ellman's reagent to establish that conjugation had occurred at the free cysteines of these histones. For this purpose, independent aliquots of H3K4C-maleimide-DBCO, H3R52C-maleimide-DBCO, H3K4CR52C-maleimide-DBCO, H3K4C-maleimide-dummy, H3R52C-maleimide-dummy and H3K4CR52C-maleimide-dummy were diluted to 25 μM with ammonium acetate buffer (20 mM, pH 8.0). Then, 10 eq. of Ellman's reagent was added to each mixture and the reactions were shaken at 400 rpm and 25 ºC. After 30 min, the reaction mixtures were analysed by LC-MS and conversion to the corresponding Ellman disulfides was not observed, indicating that maleimide conjugation had occurred at the cysteine residues of H3K4C (H3K4C-maleimide-DBCO: calculated mass = 15641 Da; observed mass= 15640 Da; H3K4C-hydrolysed-maleimide-dummy: calculated mass = 15464 Da; observed mass = 15459 Da), H3R52C (H3R52C-maleimide-DBCO: calculated mass = 15613 Da; observed mass = 15611 Da; H3R52C-hydrolysed-maleimide-dummy: calculated mass = 15436 Da; observed mass = 15430 Da) and H3K4CR52C (H3K4CR52C-maleimide-DBCO: calculated mass = 16016 Da; observed mass = 16021 Da; H3K4CR52C-hydrolysed-maleimide-dummy: calculated mass = 15661 Da; observed mass = 15664 Da). The resulting ESI-MS spectra are shown in Figures S62, S63, S64, S65, S66 and S67.

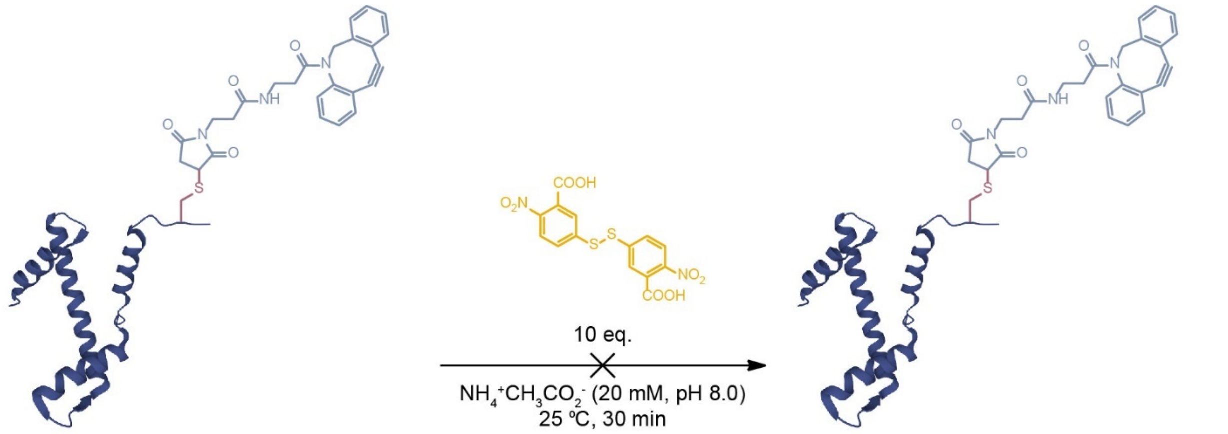

H3K4C-maleimide-DBCO (25 μM) | H3K4C-maleimide-DBCO (25 μM)

no reaction

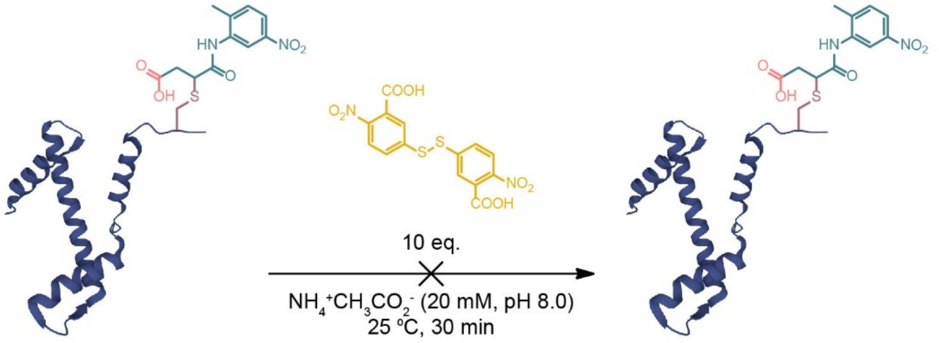

H3K4C-maleimide-dummy (25 μM) | H3K4C-maleimide-dummy (25 μM)

no reaction

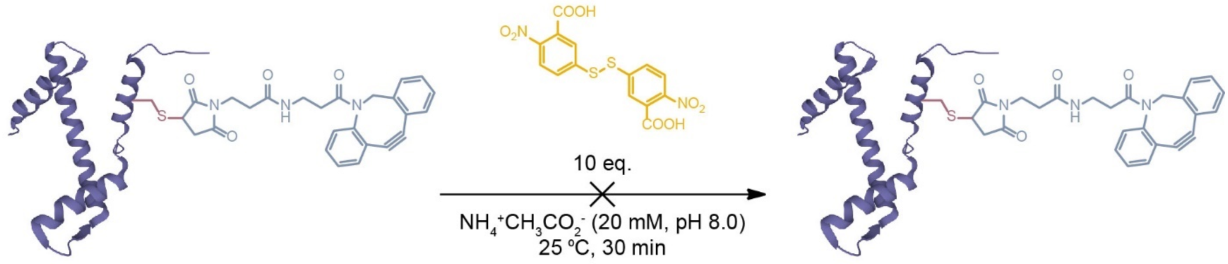

H3R52C-maleimide-DBCO (25 μM) | H3R52C-maleimide-DBCO (25 μM)

no reaction

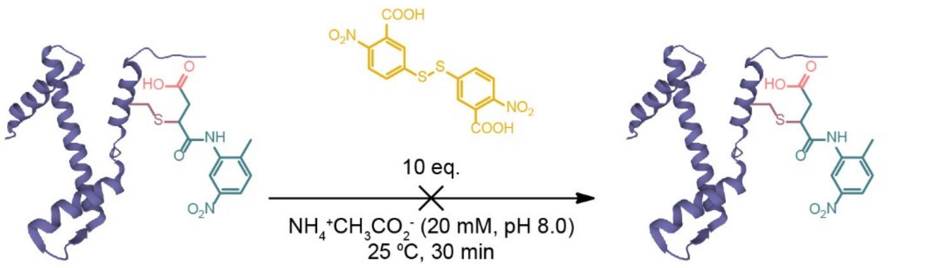

H3R52C-maleimide-dummy (25 μM) | H3R52C-maleimide-dummy (25 μM)

no reaction

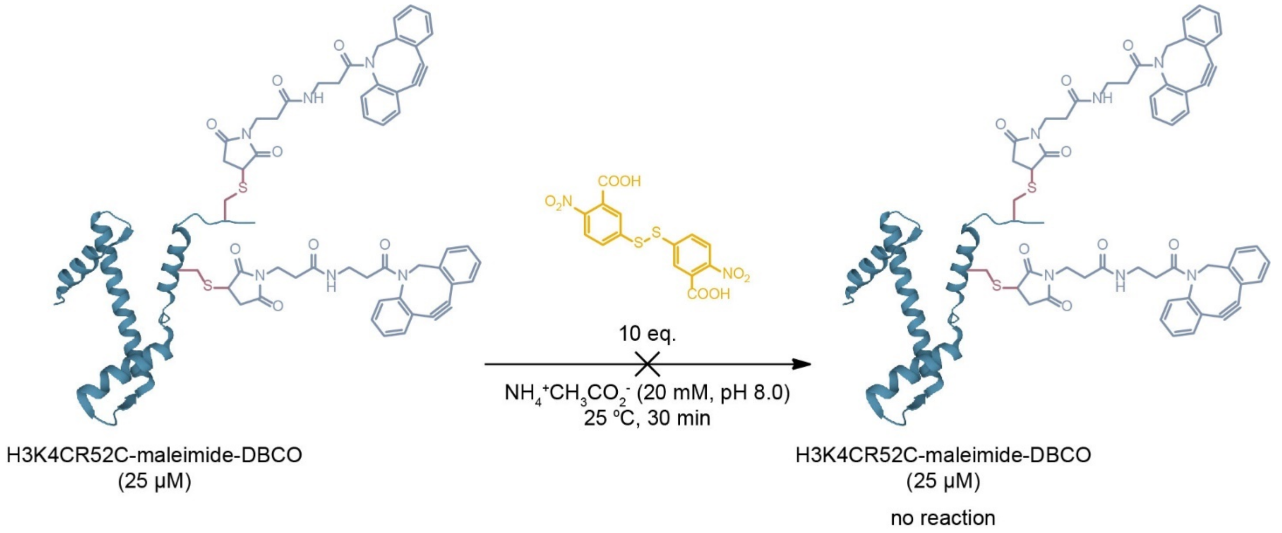

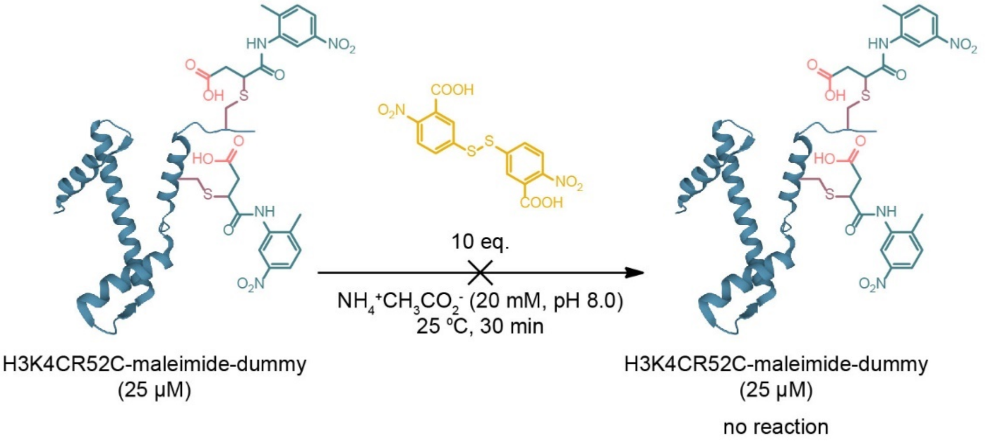

General procedure to generate unacetylated histones containing the SPAAC product at the cysteine

To serve as control proteins in further studies, we also prepared unacetylated H3K4C, H3R52C and H3K4CR52C histones bearing the SPAAC product that remains attached at the cysteine. Thus, H3K4C, H3R52C and H3K4CR52C proteins were reduced and conjugated to maleimide-DBCO according to the protocols already described. Subsequently, the SPAAC reaction was performed by incubating the modified proteins with compound **2** using the same reaction parameters reported previously for compound **4**. More specifically, independent aliquots of the H3K4C, H3R52C and H3K4CR52C proteins containing the maleimide-DBCO clickable handle were diluted to 25 µM in ammonium acetate buffer (20 mM, pH 4.9). An aliquot of 4 eq. of compound **2** was added to the diluted H3K4C-maleimide-DBCO and H3K4CR52C-maleimide-DBCO solutions, while an aliquot of 2 eq. of the same compound was added to the solution containing the diluted H3R52C-maleimide-DBCO protein. The reactions were shaken at 400 rpm and 25 ºC. After 30 min, the reaction mixtures were analysed by LC-MS and complete conversion to the respective H3K4C**, H3R52C** and H3K4C**R52C** SPAAC products was observed (H3K4C**: calculated mass = 15847 Da; observed mass = 15842 Da; H3R52C**: calculated mass = 15819 Da; observed mass = 15816 Da; H3K4C**R52C**: calculated mass = 16426 Da; observed mass = 16431 Da). The resulting ESI-MS spectra are shown in Figures S68, S69 and S70. The proteins were buffer exchanged to ammonium acetate buffer (20 mM, pH 5.8), desalted to water and concentrated using Amicon centrifugal filter units (Merck Millipore). Protein concentrations were then determined by the Bradford assay. Finally, the aliquots of H3K4C**, H3R52C** and H3K4C**R52C** were lyophilized and frozen at -80 ºC for further studies.

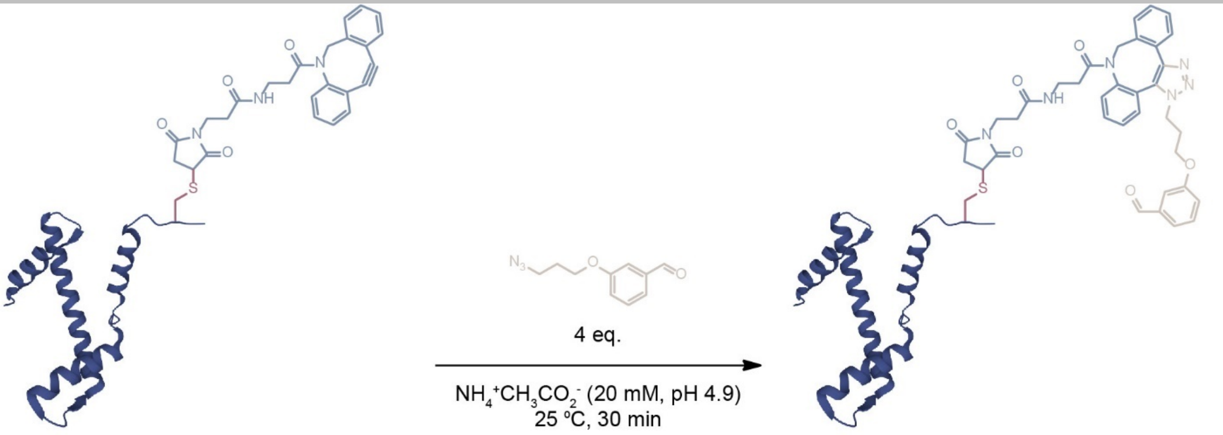

H3K4C-maleimide-DBCO (25 µM) → H3K4C** (25 µM) full conversion

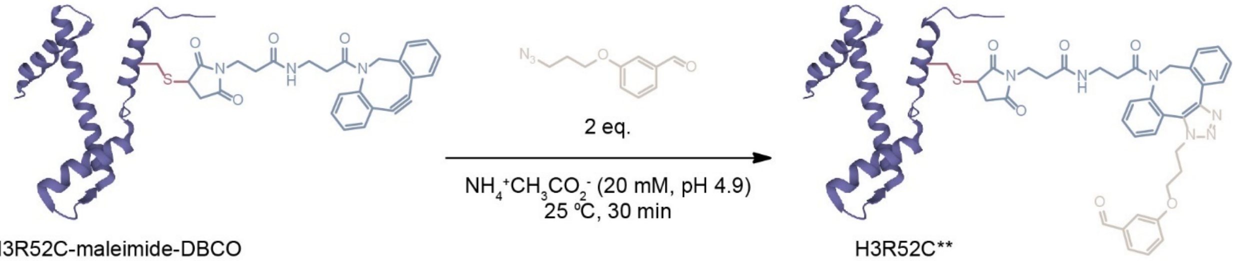

H3R52C-maleimide-DBCO (25 µM) → H3R52C** (25 µM) full conversion

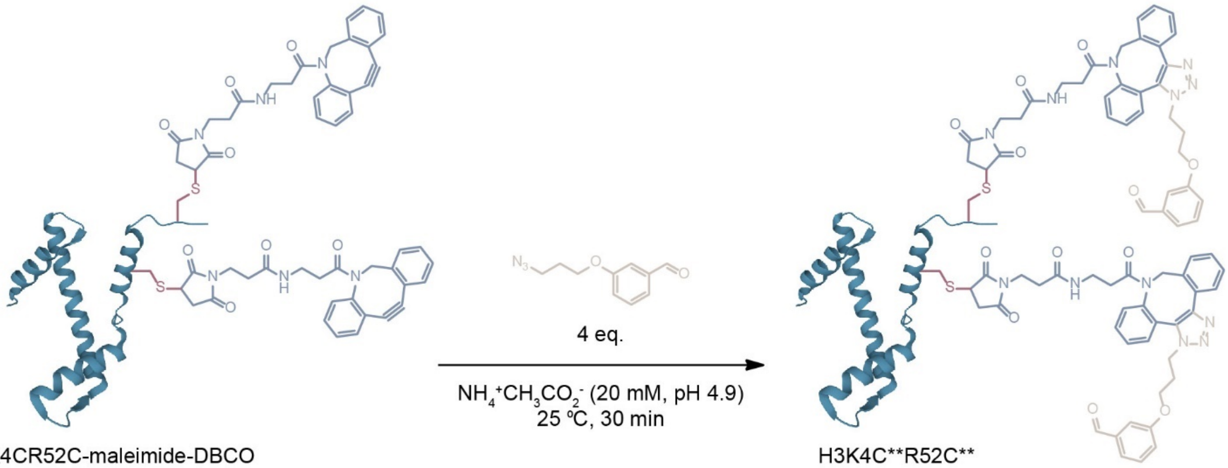

H3K4CR52C-maleimide-DBCO (25 µM) → H3K4C**R52C** (25 µM) full conversion

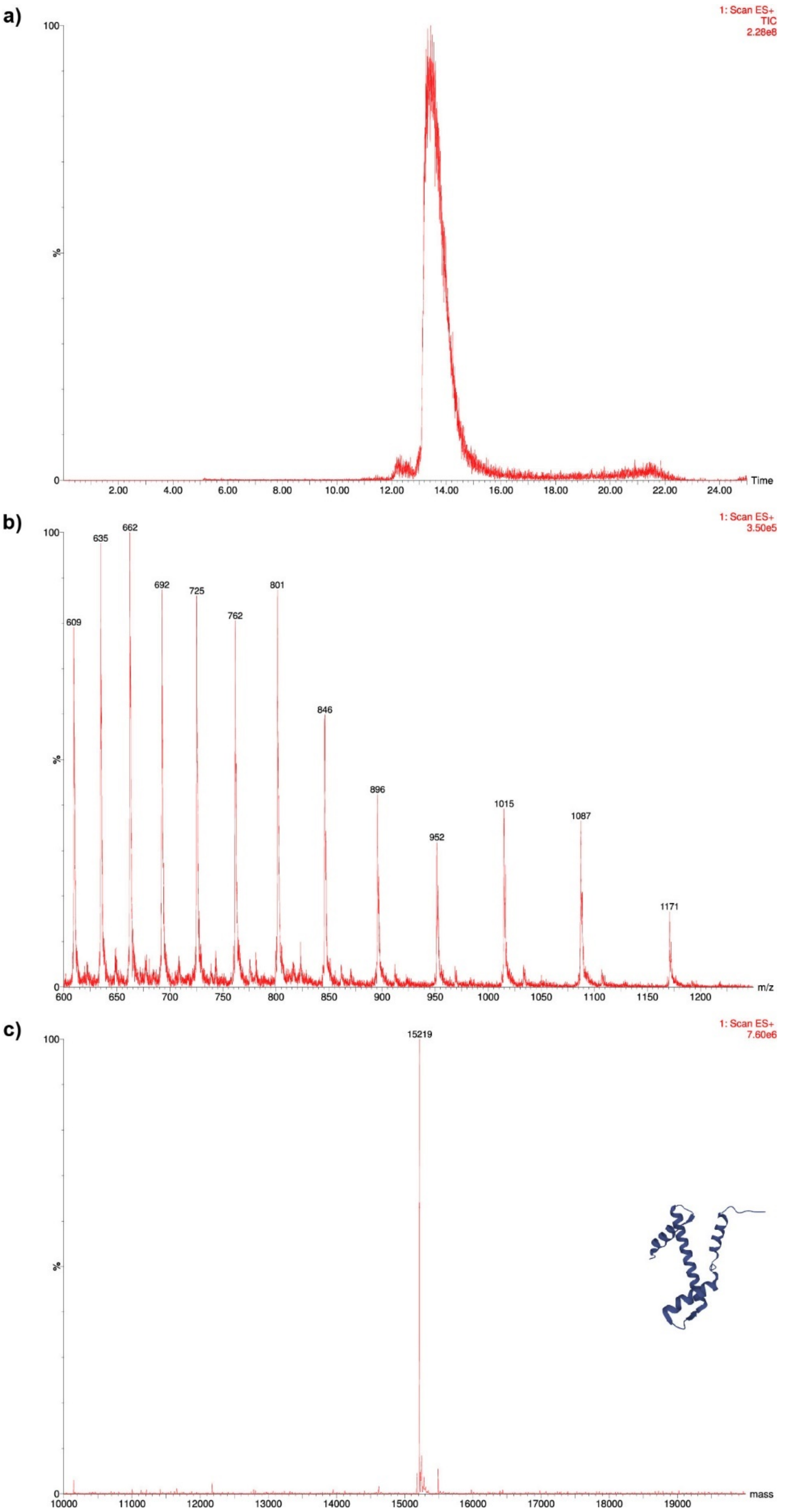

Figure S28. LC-MS of the purified H3K4C protein (25 μM); **a)** total ion current chromatogram, **b)** ion series spectrum and **c)** deconvoluted mass spectrum with structural representation of the major product identified: Protein H3K4C (H3K4C: 15219 Da).

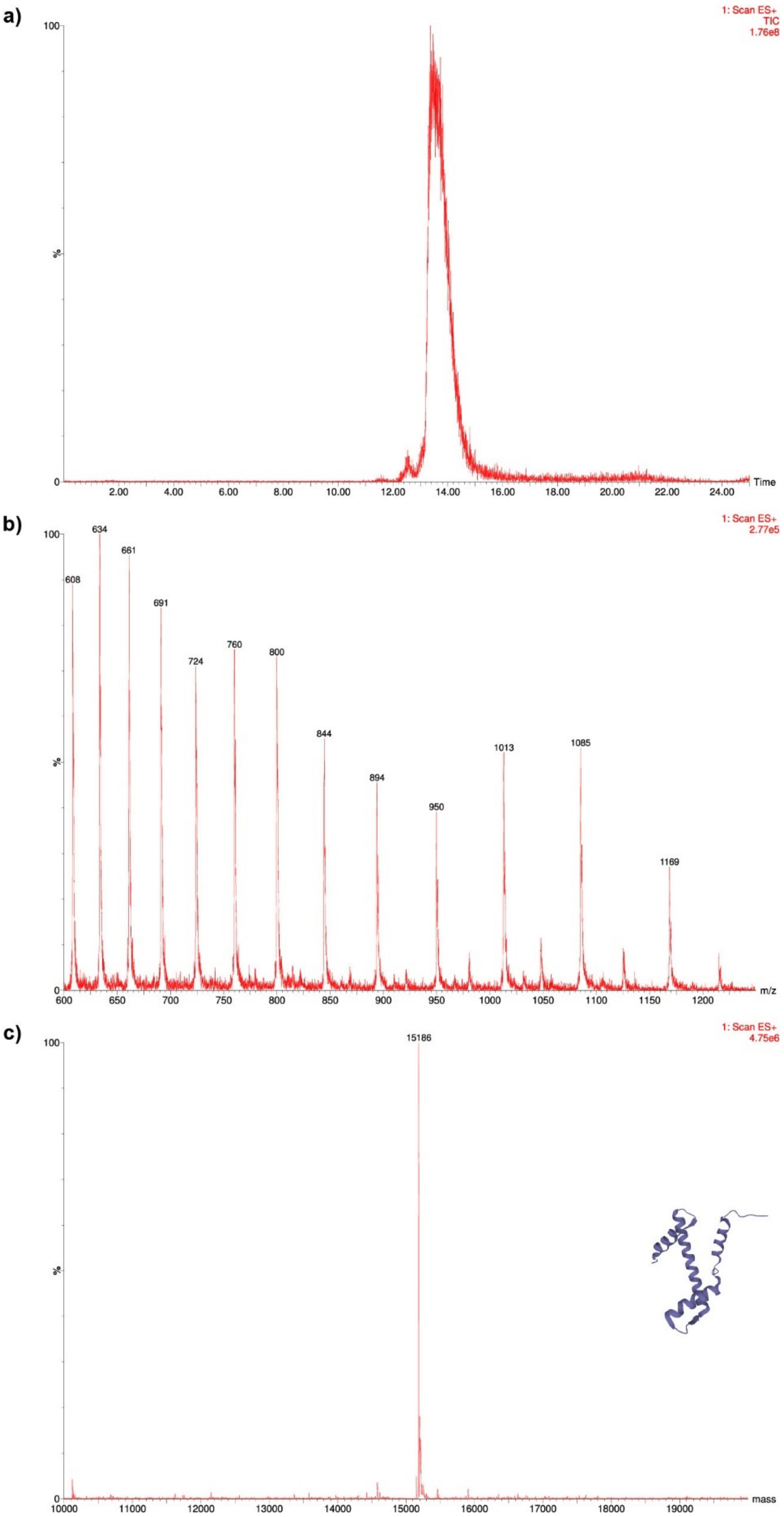

Figure S29. LC-MS of the purified H3R52C protein (25 µM); **a)** total ion current chromatogram, **b)** ion series spectrum and **c)** deconvoluted spectrum with structural representation of the major product identified: Protein H3R52C (H3R52C: 15186 Da).

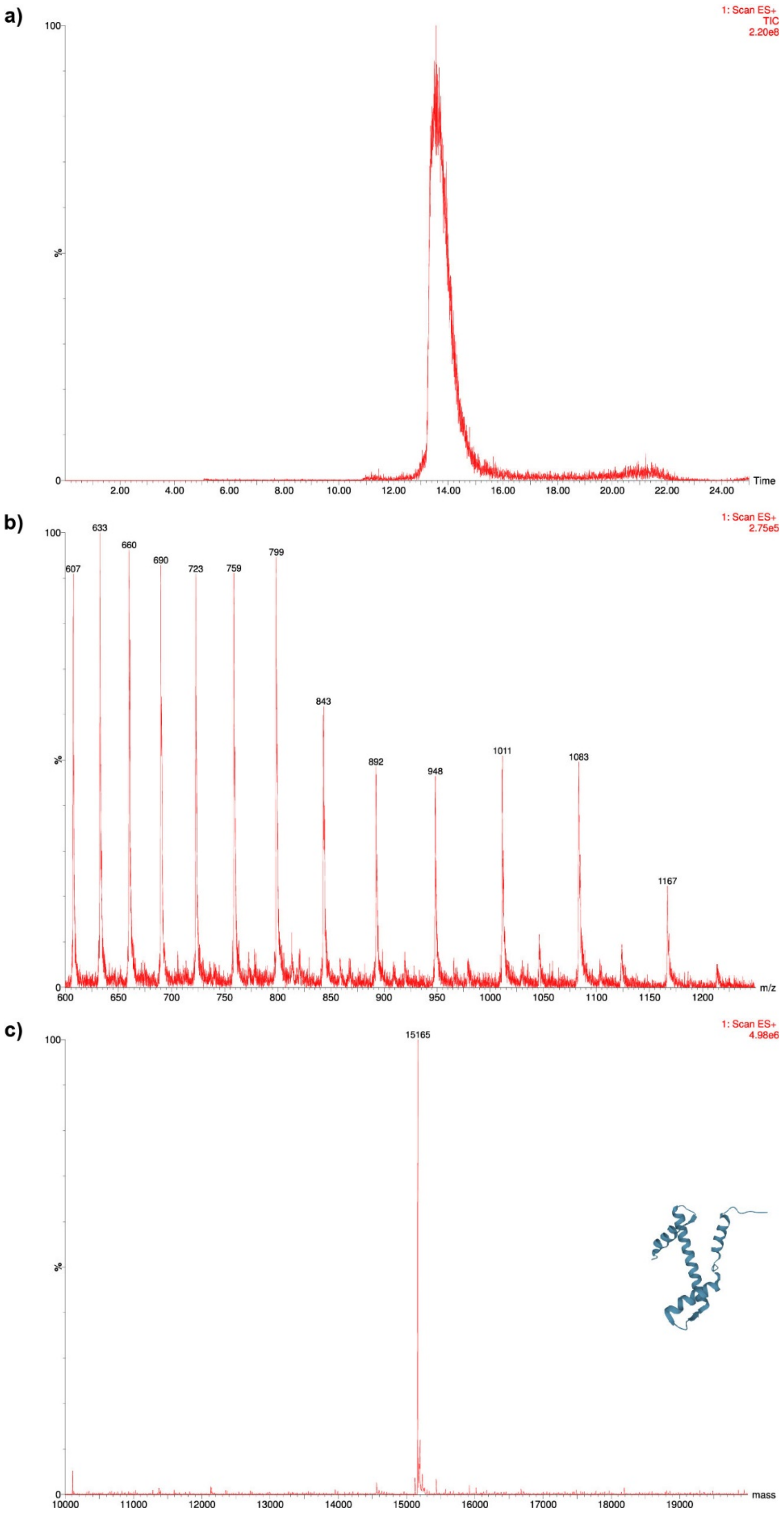

Figure S30. LC-MS of the purified H3K4CR52C protein (25 µM); **a)** total ion current chromatogram, **b)** ion series spectrum and **c)** deconvoluted spectrum with structural representation of the major product identified: Protein H3K4CR52C (H3K4CR52C: 15165 Da).

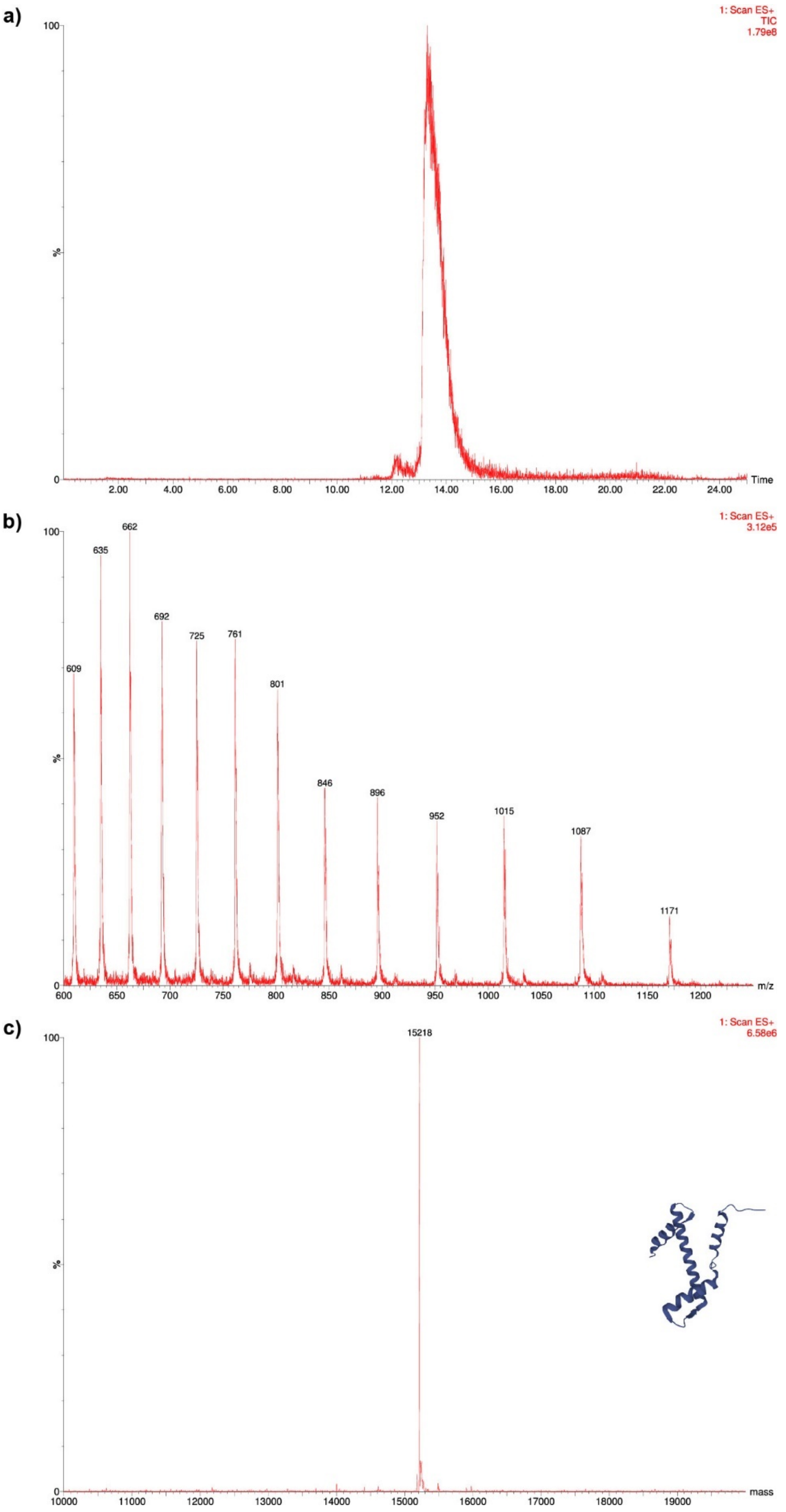

Figure S31. LC-MS of the reduced H3K4C protein (25 µM) in ammonium acetate buffer (20 mM, pH 7.0); **a)** total ion current chromatogram, **b)** ion series spectrum and **c)** deconvoluted mass spectrum with structural representation of the major product identified: Protein H3K4C (H3K4C: 15218 Da).

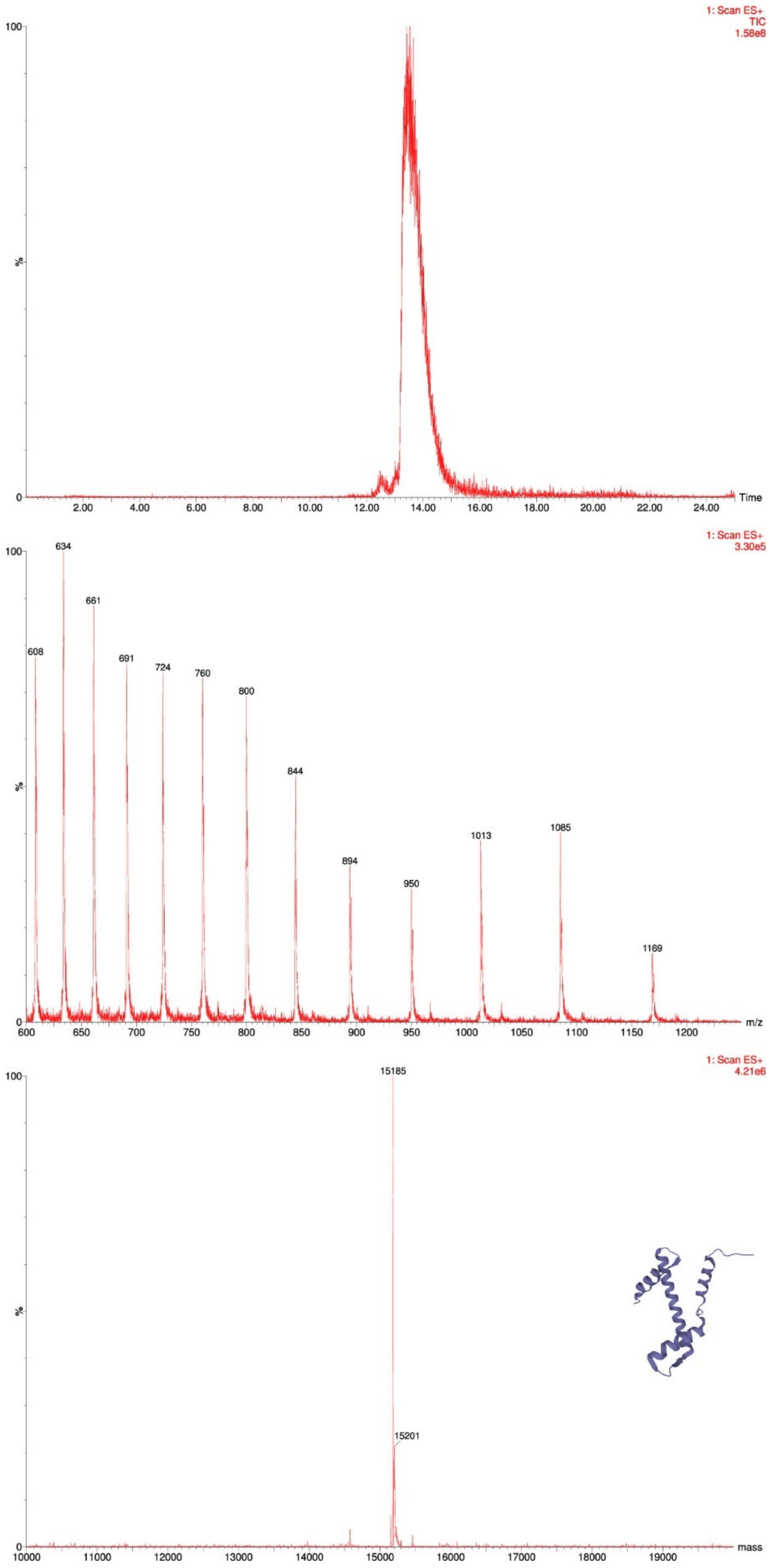

Figure S32. LC-MS of the reduced H3R52C protein (25 µM) in ammonium acetate buffer (20 mM, pH 7.0); **a)** total ion current chromatogram, **b)** ion series spectrum and **c)** deconvoluted spectrum with structural representation of the major product identified: Protein H3R52C (H3R52C: 15185 Da).

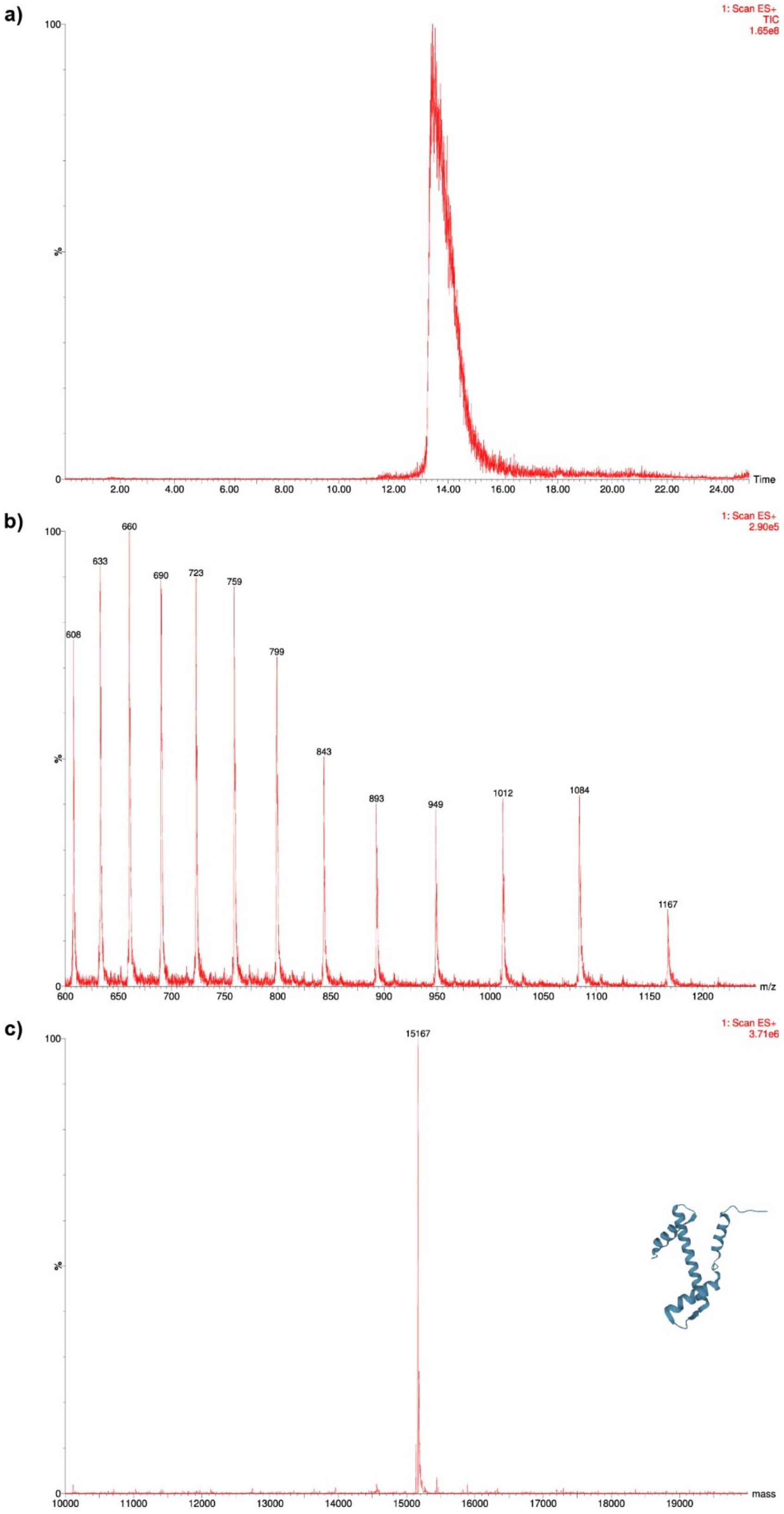

Figure S33. LC-MS of the reduced H3K4CR52C protein (25 µM) in ammonium acetate buffer (20 mM, pH 7.0); **a)** total ion current chromatogram, **b)** ion series spectrum and **c)** deconvoluted spectrum with structural representation of the major product identified: Protein H3K4CR52C (H3K4CR52C: 15167 Da).

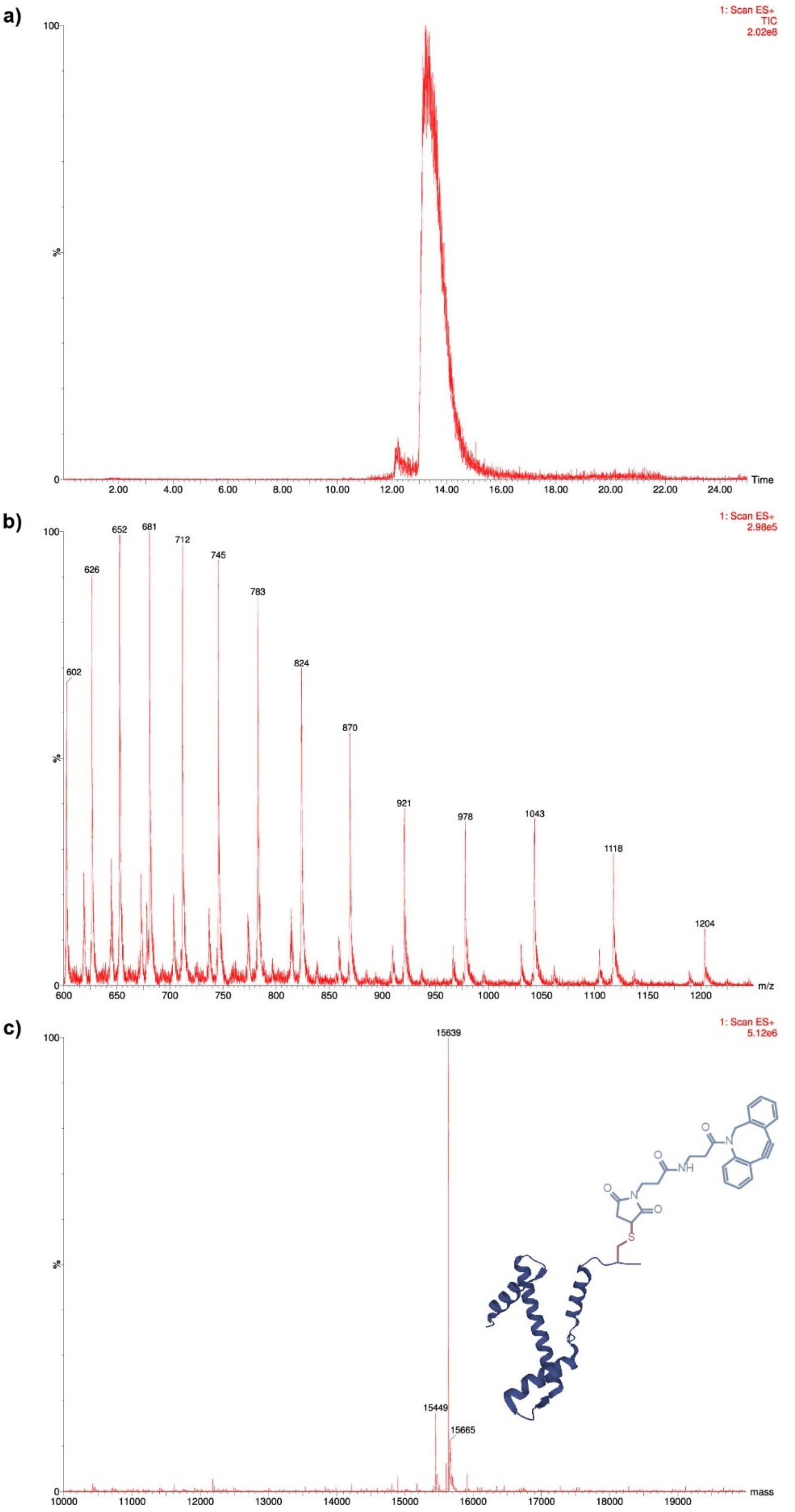

Figure S34. LC-MS of the reduced H3K4C protein (25 µM) following maleimide-DBCO incubation (62.5 µM) in ammonium acetate buffer (20 mM, pH 7.0) for 1 h at 25 ºC and 400 rpm; **a)** total ion current chromatogram, **b)** ion series spectrum and **c)** deconvoluted spectrum with structural representation of the major product identified: Protein H3K4C + one maleimide-DBCO (H3K4C-maleimide-DBCO: 15639 Da).

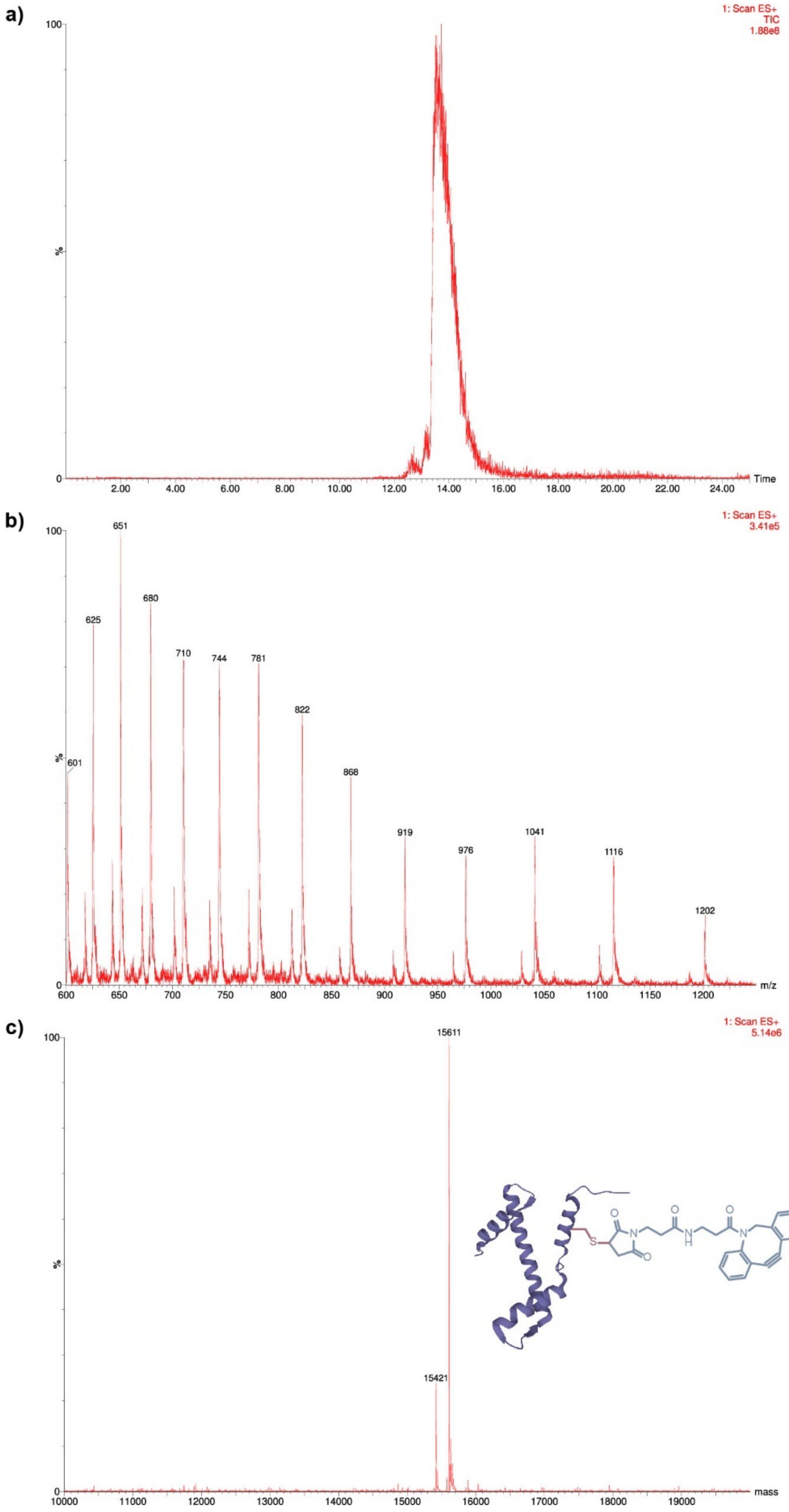

Figure S35. LC-MS of the reduced H3R52C protein (25 µM) following maleimide-DBCO incubation (62.5 µM) in ammonium acetate buffer (20 mM, pH 7.0) for 1 h at 25 ºC and 400 rpm; **a)** total ion current chromatogram, **b)** ion series spectrum and **c)** deconvoluted spectrum with structural representation of the major product identified: H3R52C protein + one maleimide-DBCO (H3R52C-maleimide-DBCO: 15611 Da).

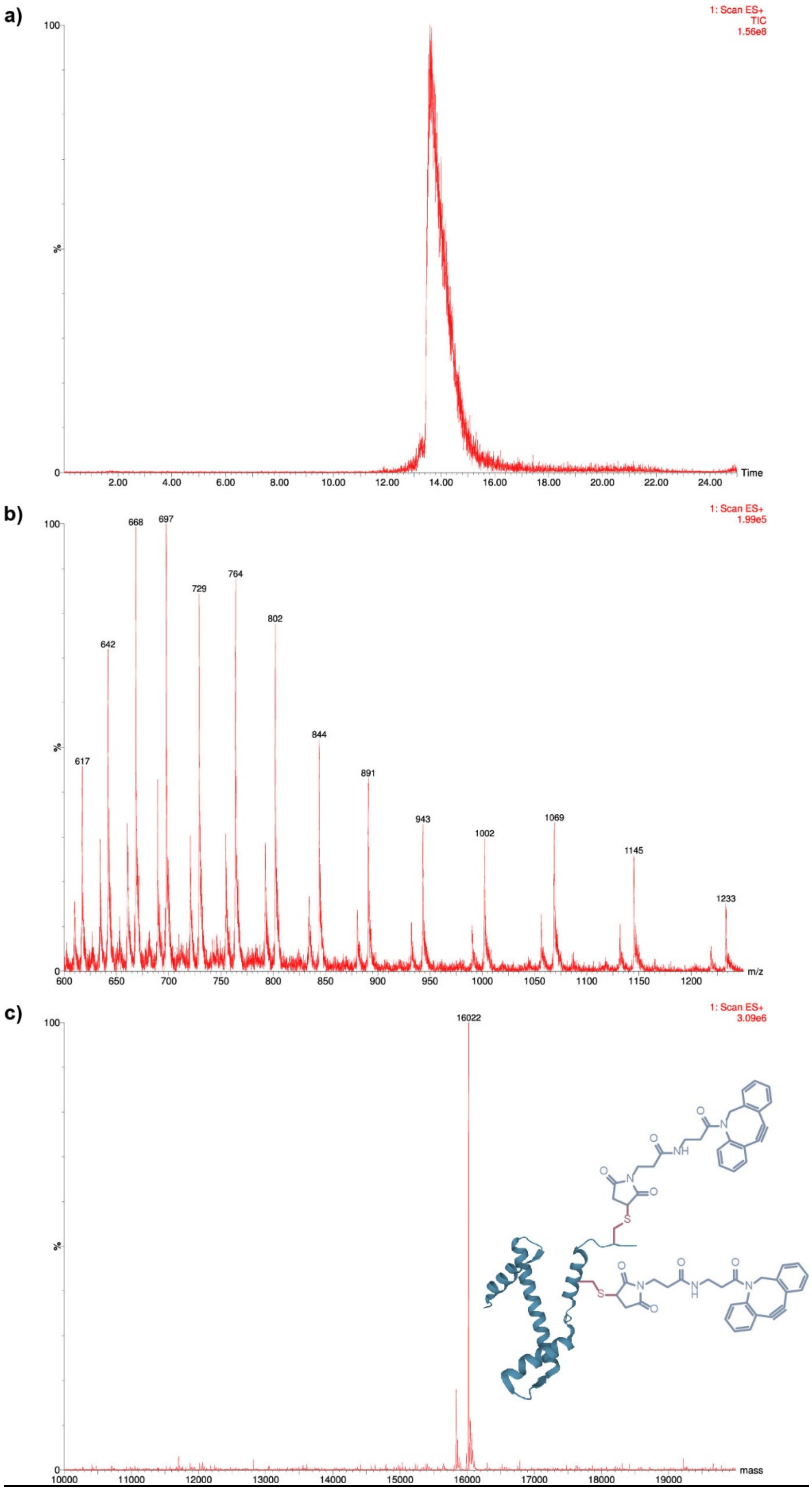

Figure S36. LC-MS of the reduced H3K4CR52C protein (25 µM) following maleimide-DBCO incubation (62.5 µM) in ammonium acetate buffer (20 mM, pH 7.0) for 1 h at 25 ºC and 400 rpm; **a)** total ion current chromatogram, **b)** ion series spectrum and **c)** deconvoluted spectrum with structural representation of the major product identified: H3K4CR52C protein + two maleimide-DBCOs (H3K4CR52C-maleimide-DBCO: 16022 Da).

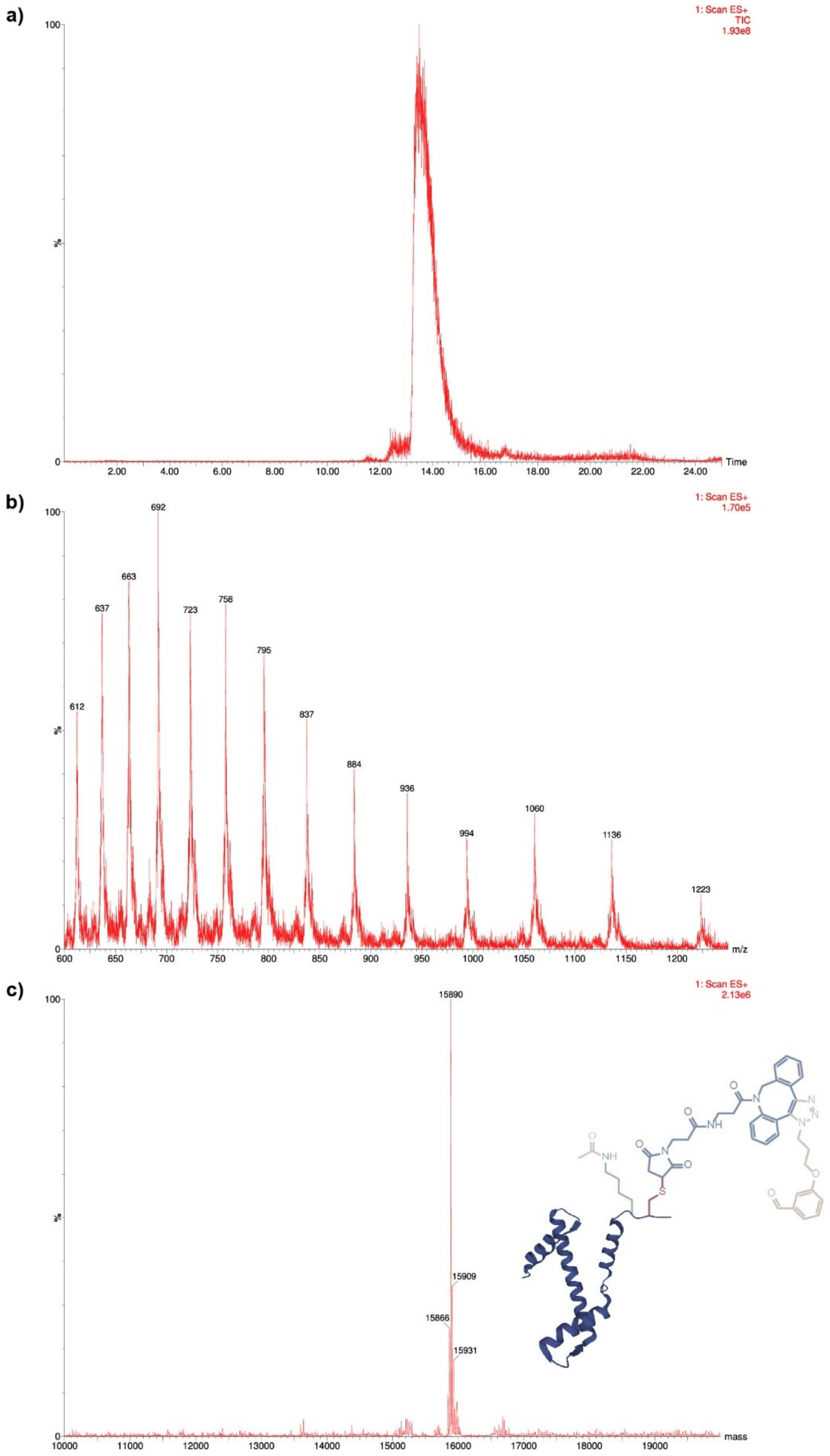

Figure S37. LC-MS of the H3K4C-maleimide-DBCO protein (25 µM) following incubation with compound **4** (100 µM) in ammonium acetate buffer (20 mM, pH 8.0) for 30 min at 25 ºC and 400 rpm; **a)** total ion current chromatogram, **b)** ion series spectrum and **c)** deconvoluted spectrum with structural representation of the major product identified: Protein H3K4C + one maleimide-DBCO + one deacetylated SPAAC product + mono-acetylation (H3K4C**K9Ac: 15890 Da).

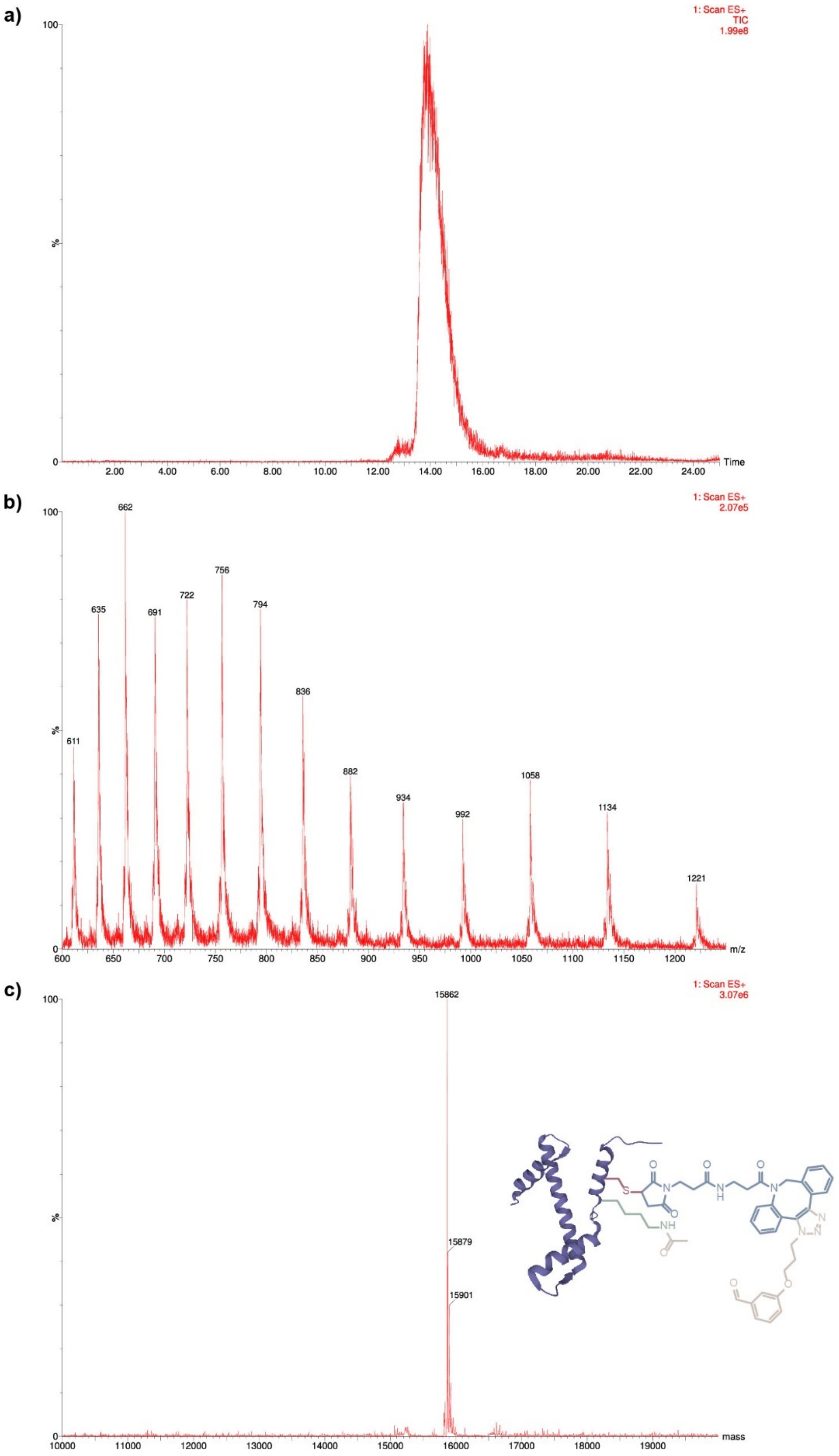

Figure S38. LC-MS of the H3R52C-maleimide-DBCO protein (25 µM) following incubation with compound **4** (50 µM) in ammonium acetate buffer (20 mM, pH 8.0) for 30 min at 25 ºC and 400 rpm; **a)** total ion current chromatogram, **b)** ion series spectrum and **c)** deconvoluted spectrum with structural representation of the major product identified: Protein H3R52C + one maleimide-DBCO + one deacetylated SPAAC product + mono-acetylation (H3R52**K56Ac: 15862 Da).

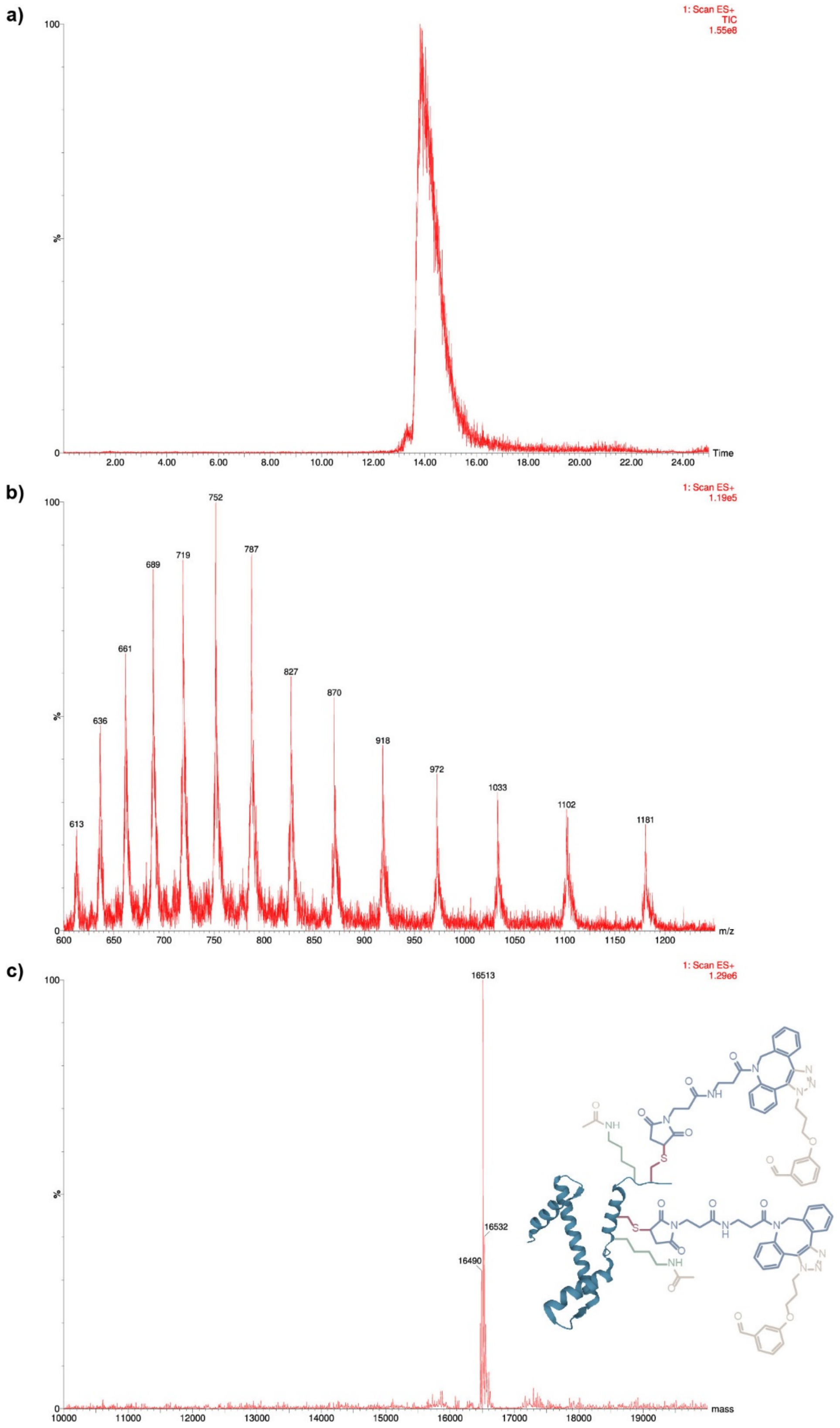

Figure S39. LC-MS of the H3K4CR52C-maleimide-DBCO protein (25 µM) following incubation with compound **4** (100 µM) in ammonium acetate buffer (20 mM, pH 8.0) for 45 min at 25 ºC and 400 rpm; **a)** total ion current chromatogram, **b)** ion series spectrum and **c)** deconvoluted spectrum with structural representation of the major product identified: Protein H3K4CR52C + two maleimide-DBCOs + two deacetylated SPAAC products + di-acetylation (H3K4C**K9AcR52C**K56Ac: 16513 Da).

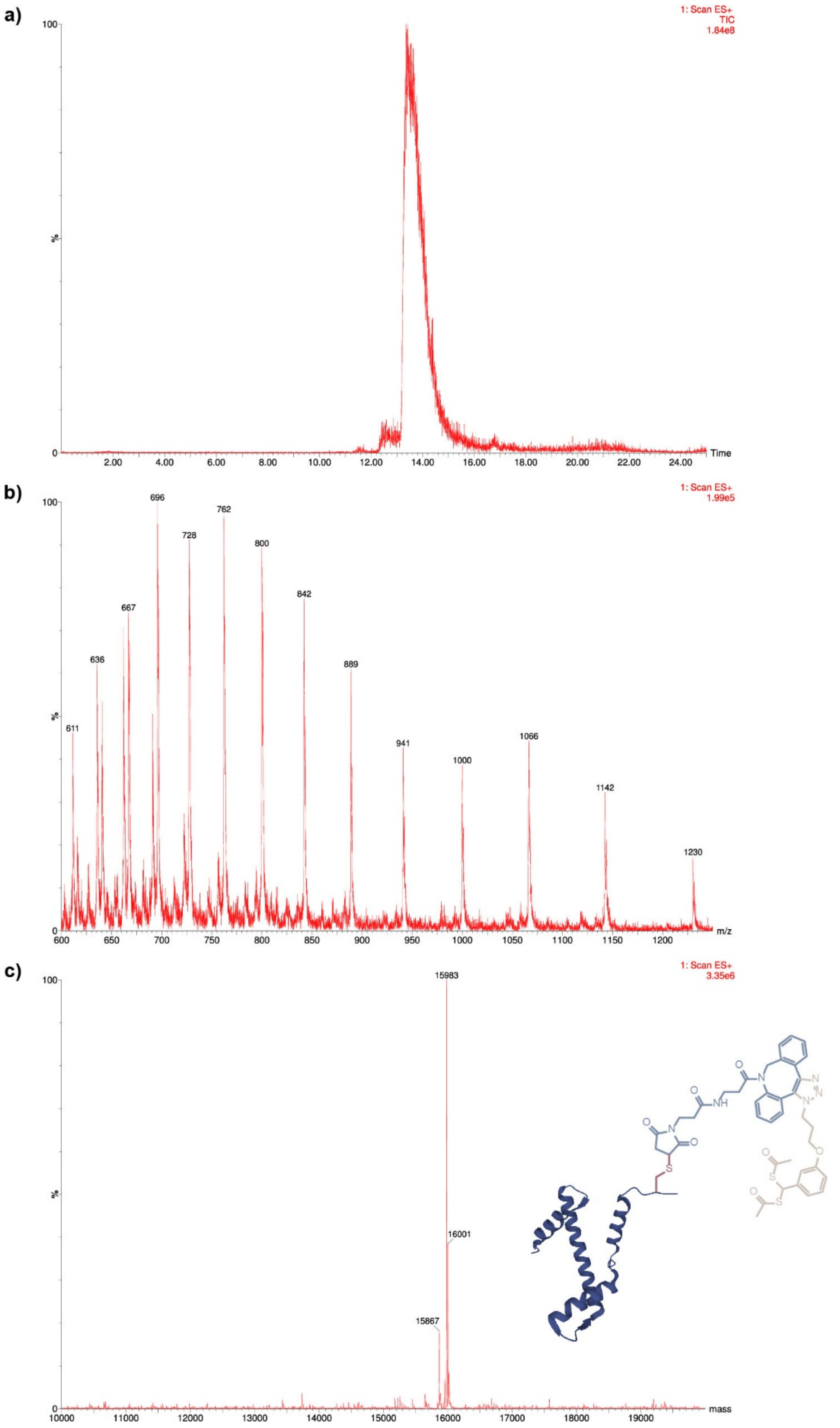

Figure S40. LC-MS of the H3K4C-maleimide-DBCO protein (25 µM) following incubation with compound **4** (100 µM) in ammonium acetate buffer (20 mM, pH 4.9) for 30 min at 25 ºC and 400 rpm; **a)** total ion current chromatogram, **b)** ion series spectrum and **c)** deconvoluted spectrum with structural representation of the major product identified: Protein H3K4C + one maleimide-DBCO + one di-acetylated SPAAC product (unAcH3K4C**: 15983 Da).

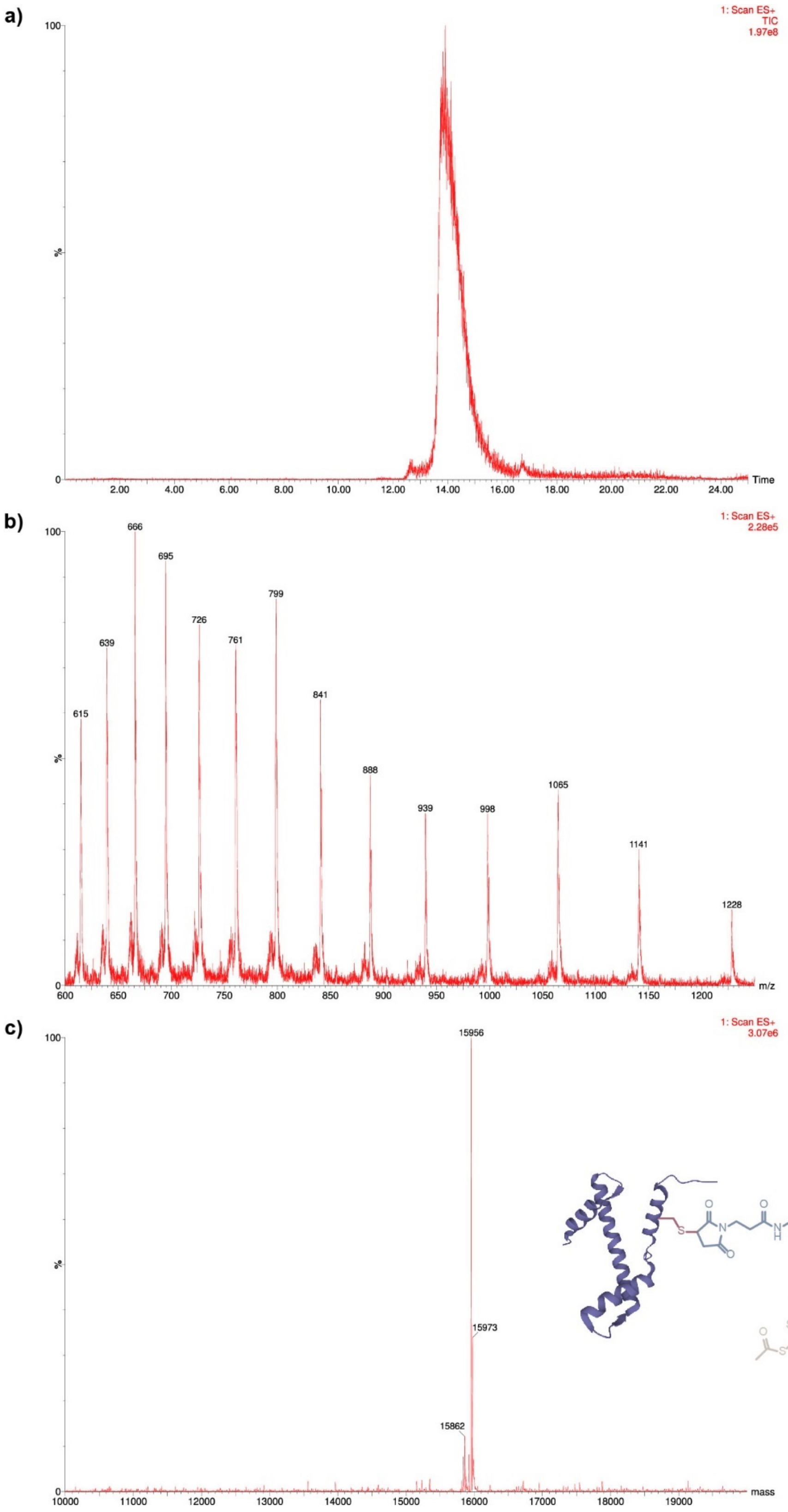

Figure S41. LC-MS of the H3R52C-maleimide-DBCO protein (25 µM) following incubation with compound **4** (50 µM) in ammonium acetate buffer (20 mM, pH 4.9) for 30 min at 25 ºC and 400 rpm; **a)** total ion current chromatogram, **b)** ion series spectrum and **c)** deconvoluted spectrum with structural representation of the major product identified: Protein H3R52C + one maleimide-DBCO + one di-acetylated SPAAC product (unAcH3R52C**: 15956 Da).

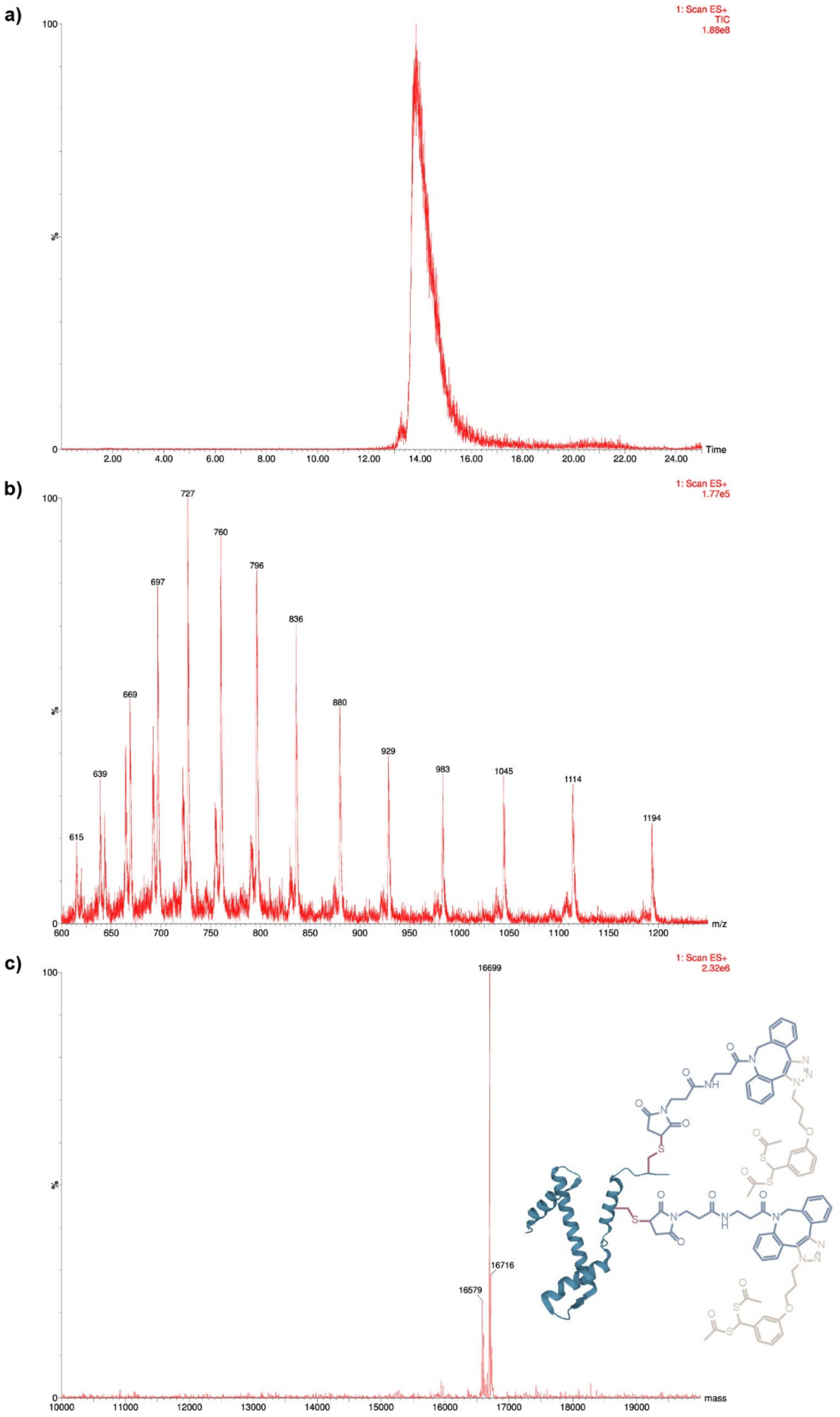

Figure S42. LC-MS of the H3K4CR52C-maleimide-DBCO protein (25 μM) following incubation with compound **4** (100 μM) in ammonium acetate buffer (20 mM, pH 4.9) for 30 min at 25 ºC and 400 rpm; **a)** total ion current chromatogram, **b)** ion series spectrum and **c)** deconvoluted spectrum with structural representation of the major product identified: Protein H3K4CR52C + two maleimide-DBCOs + two di-acetylated SPAAC products (unAcH3K4C**R52C**: 16699 Da).

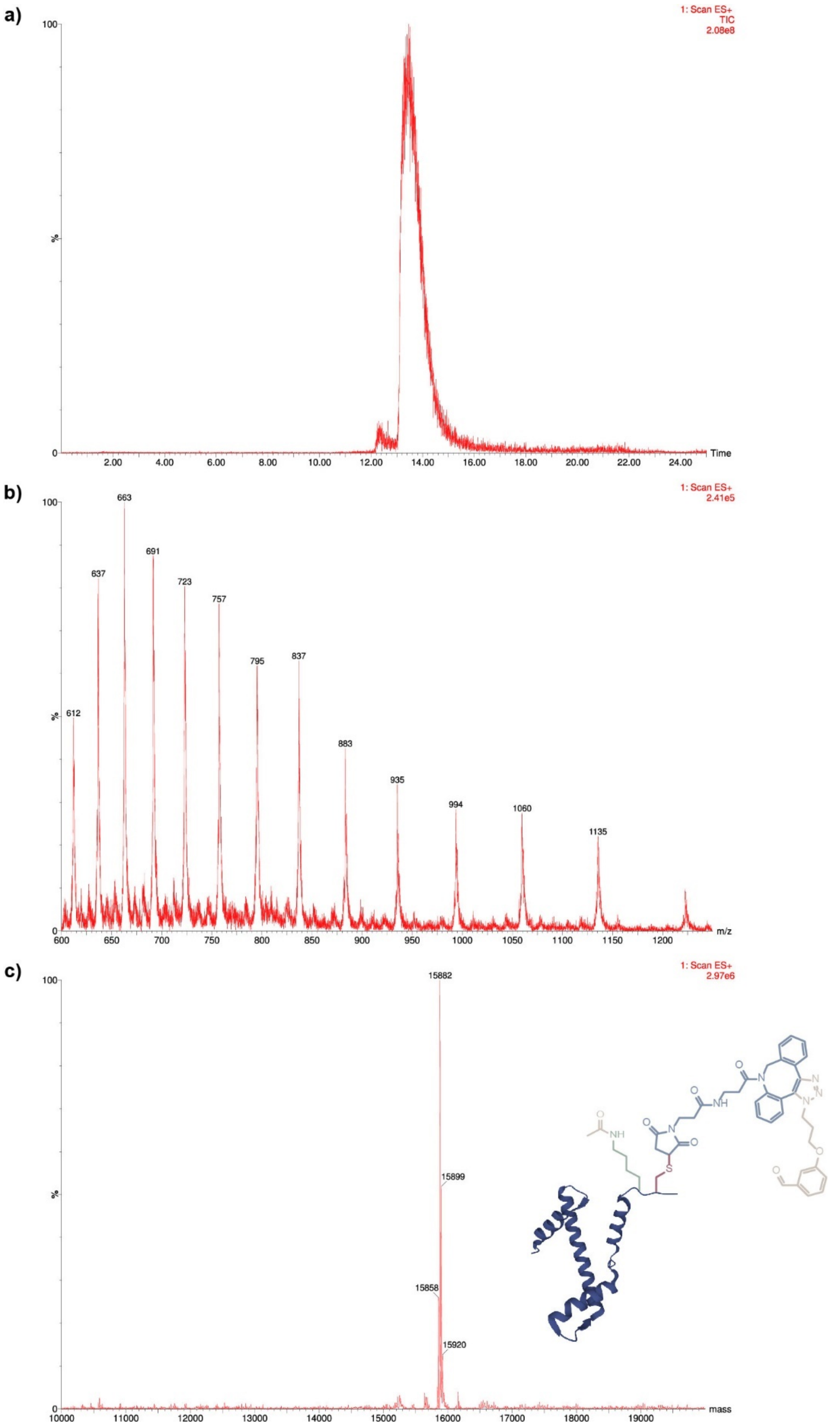

Figure S43. LC-MS of the H3K4C**K9Ac protein (25 µM) following the buffer exchange of unAcH3K4C** to ammonium acetate buffer (20 mM, pH 8.0); **a)** total ion current chromatogram, **b)** ion series spectrum and **c)** deconvoluted spectrum with structural representation of the major product identified: Protein H3K4C + one maleimide-DBCO + one deacetylated SPAAC product + mono-acetylation (H3K4C**K9Ac: 15882 Da).

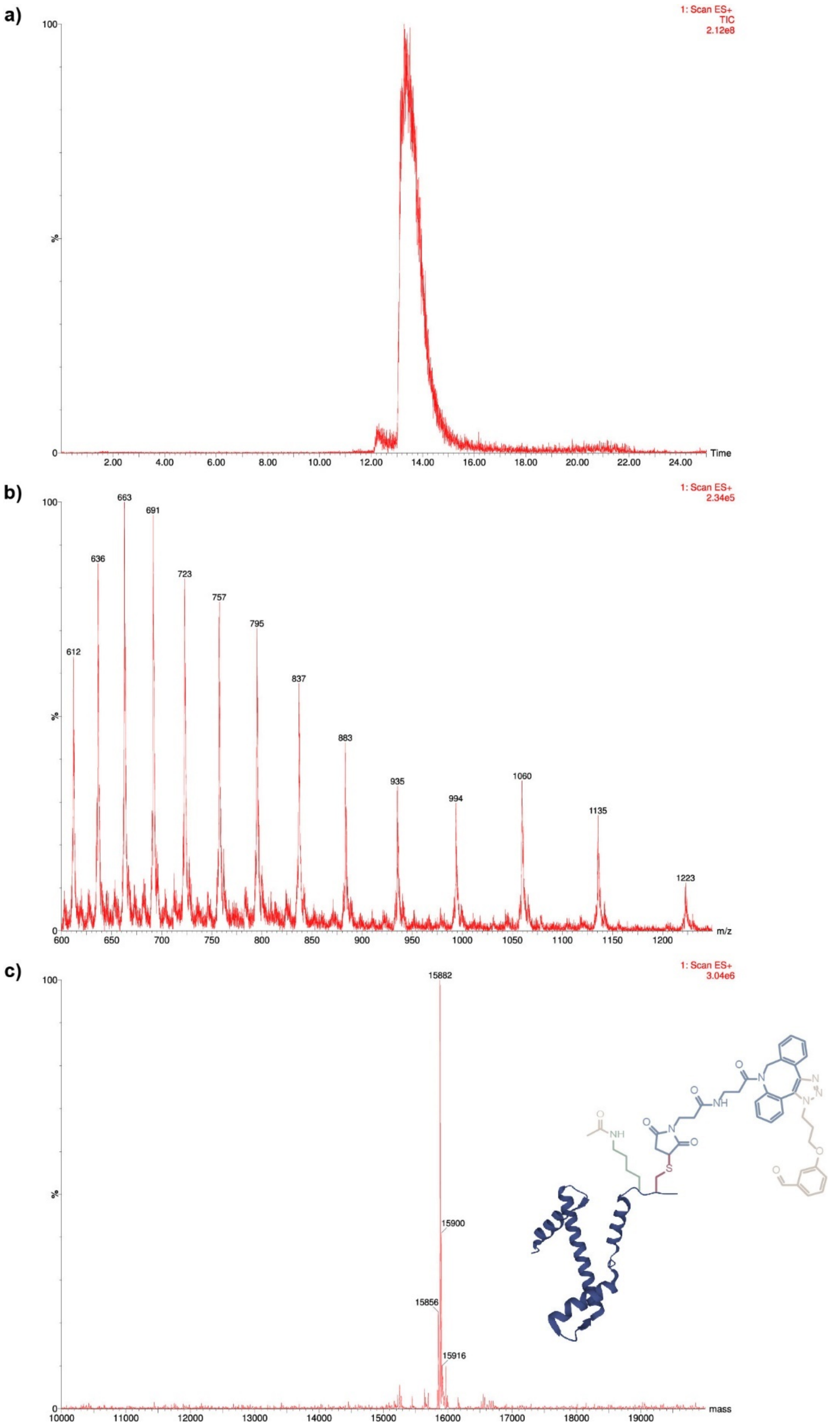

Figure S44. LC-MS of the H3K4C**K9Ac protein (25 µM) following the spontaneous acetylation of unAcH3K4C** in ammonium acetate buffer (20 mM, pH 5.8); **a)** total ion current chromatogram, **b)** ion series spectrum and **c)** deconvoluted spectrum with structural representation of the major product identified: Protein H3K4C + one maleimide-DBCO + one deacetylated SPAAC product + mono-acetylation (H3K4C**K9Ac: 15882 Da).

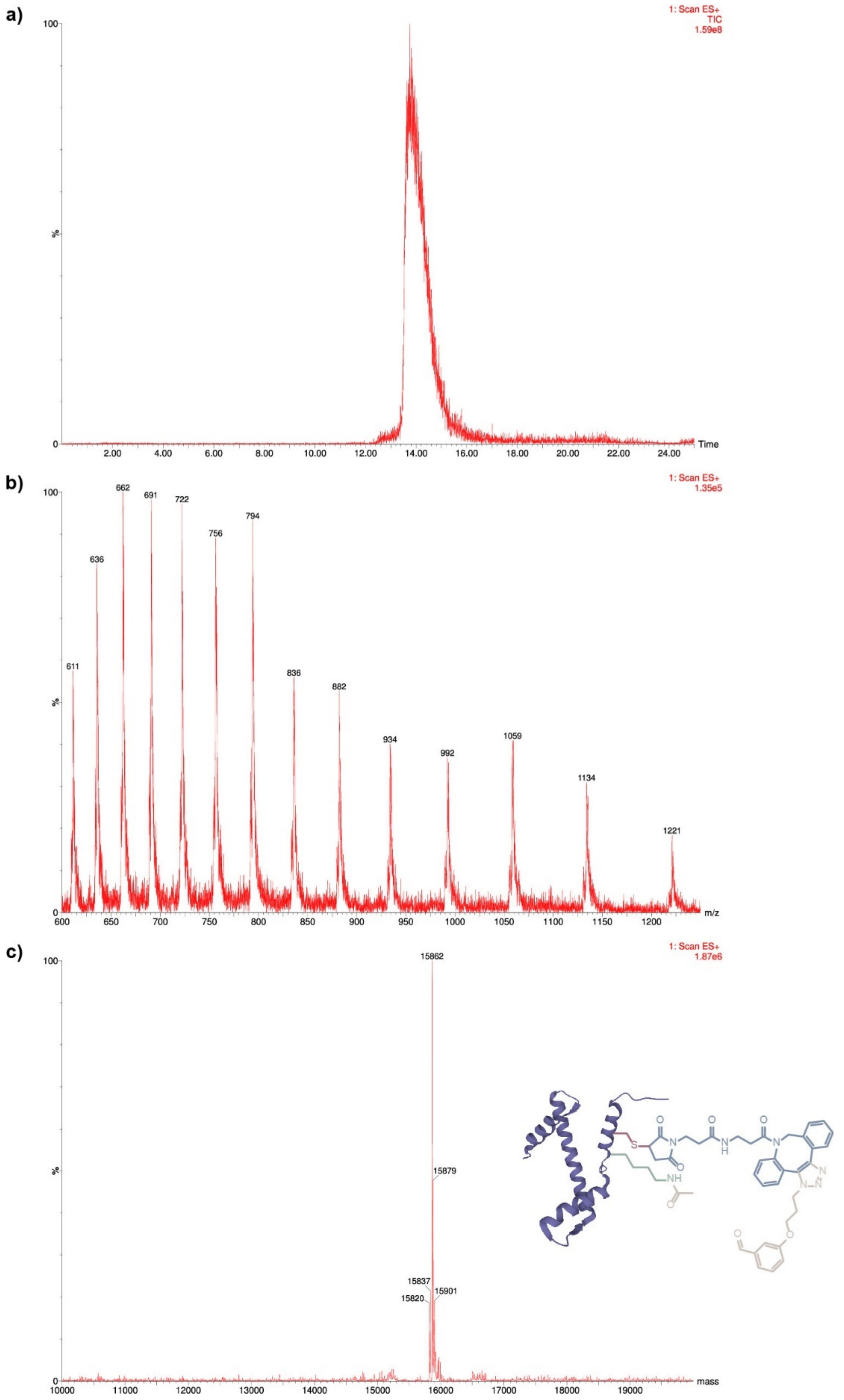

Figure S45. LC-MS of the H3R52C**K56Ac protein (25 µM) following the spontaneous acetylation of unAcH3R52C** in ammonium acetate buffer (20 mM, pH 5.8); **a)** total ion current chromatogram, **b)** ion series spectrum and **c)** deconvoluted spectrum with structural representation of the major product identified: Protein H3R52C + one maleimide-DBCO + one deacetylated SPAAC product + mono-acetylation (H3R52C**K56Ac: 15862 Da).

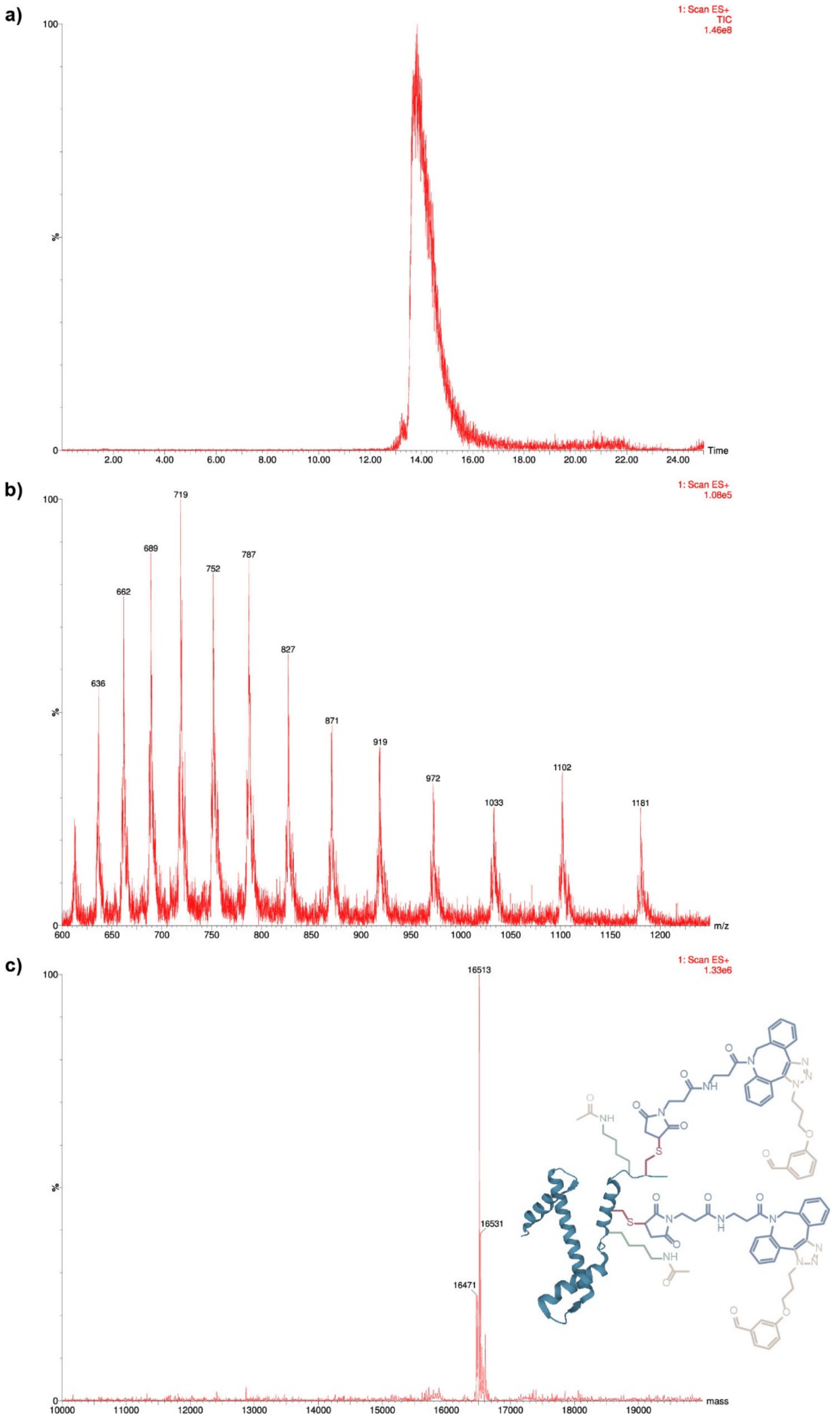

Figure S46. LC-MS of the H3K4C**K9AcR52C**K56Ac protein (25 µM) following the spontaneous acetylation of unAcH3K4C**R52C** in ammonium acetate buffer (20 mM, pH 5.8); **a)** total ion current chromatogram, **b)** ion series spectrum and **c)** deconvoluted spectrum with structural representation of the major product identified: Protein H3K4CR52C + two maleimide-DBCOs + two deacetylated SPAAC products + di-acetylation (H3K4C**K9AcR52C**K56Ac: 16513 Da).

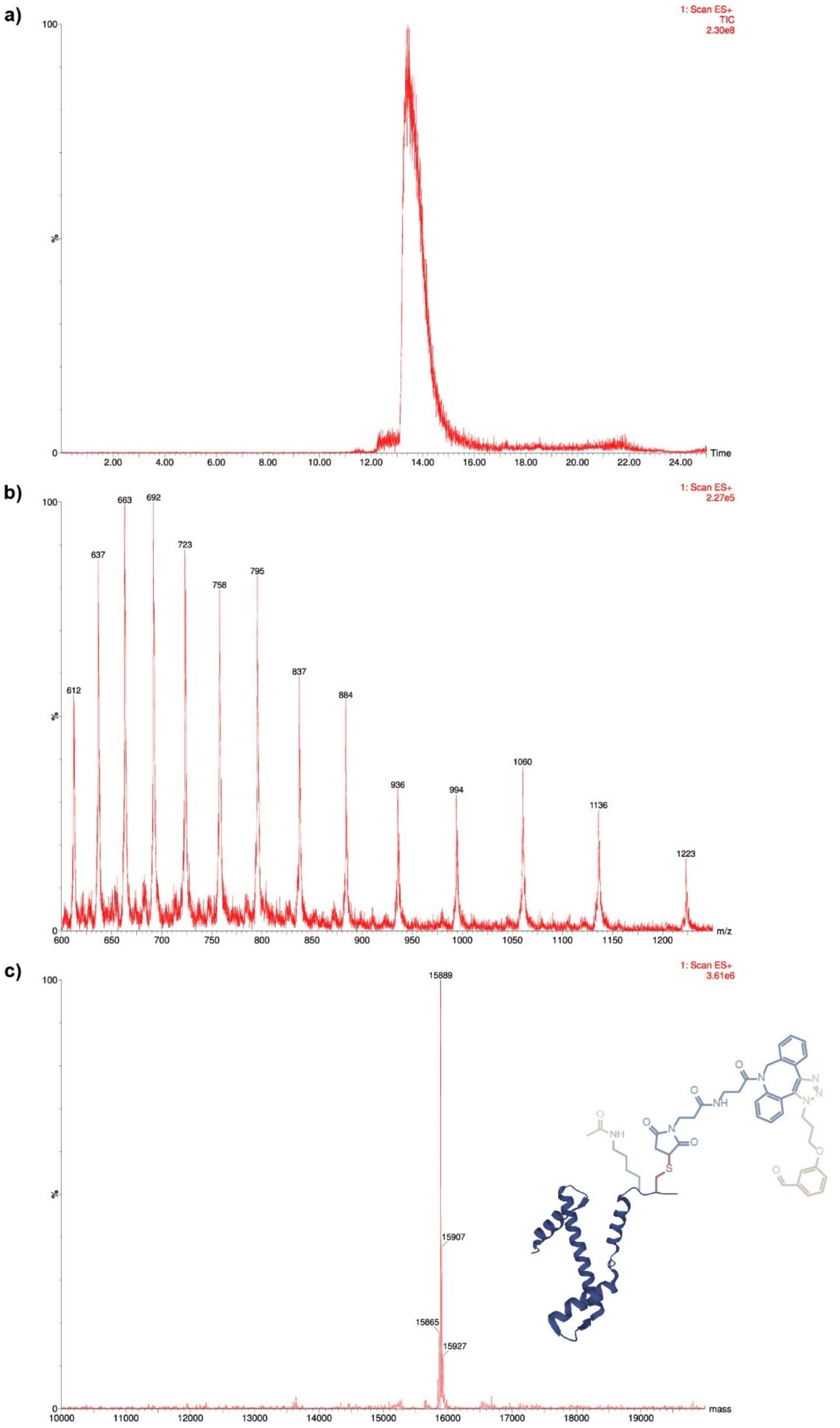

Figure S47. LC-MS of the H3K4C**K9Ac protein (25 µM) in water following spontaneous acetylation of unAcH3K4C** in ammonium acetate buffer (20 mM, pH 5.8) and desalting; **a)** total ion current chromatogram, **b)** ion series spectrum and **c)** deconvoluted spectrum with structural representation of the major product identified: Protein H3K4C + one maleimide-DBCO + one deacetylated SPAAC product + mono-acetylation (H3K4C**K9Ac: 15889 Da).

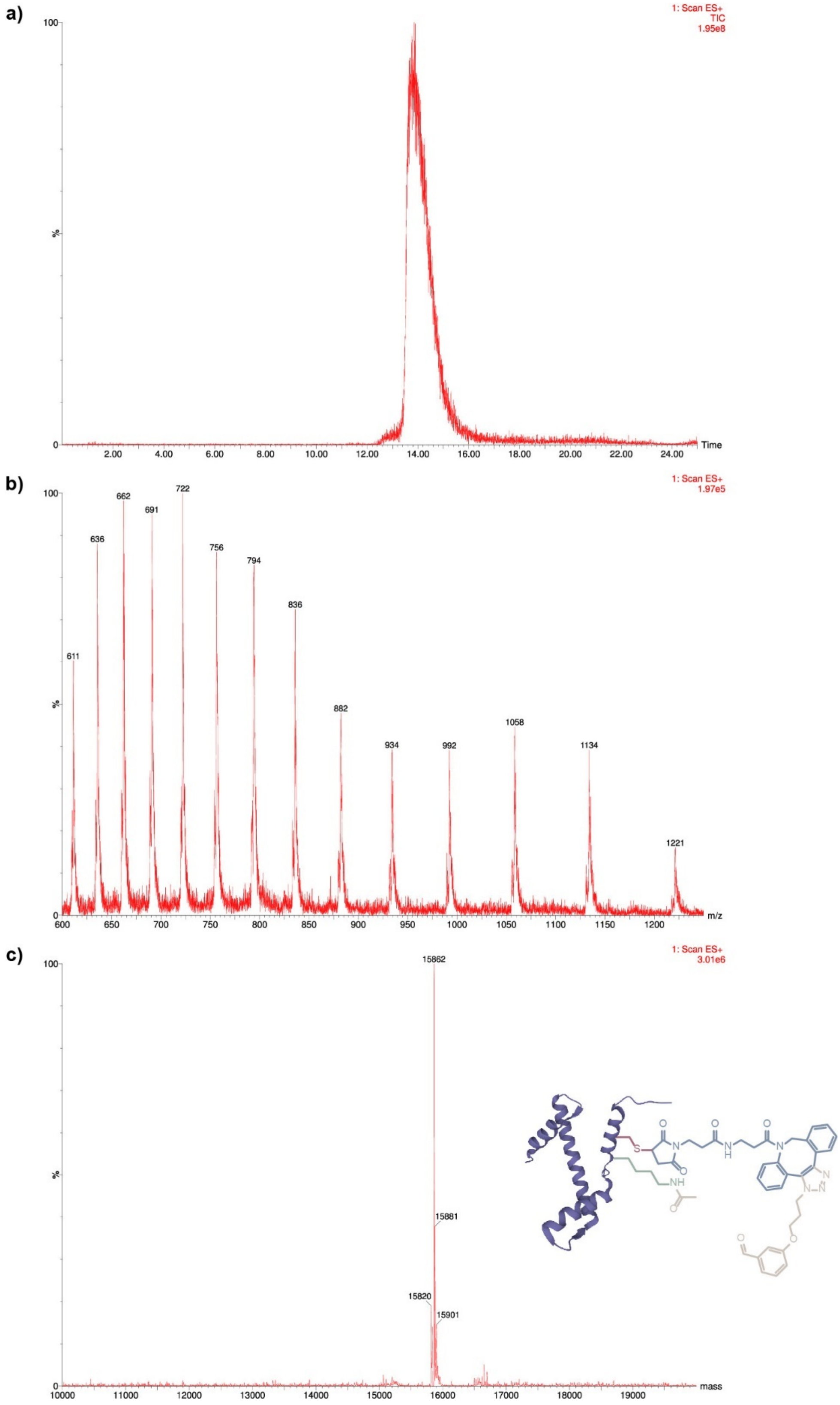

Figure S48. LC-MS of the H3R52C**K56Ac protein (25 µM) in water following spontaneous acetylation of unAcH3R52C** in ammonium acetate buffer (20 mM, pH 5.8) and desalting; **a)** total ion current chromatogram, **b)** ion series spectrum and **c)** deconvoluted spectrum with structural representation of the major product identified: Protein H3R52C + one maleimide-DBCO + one deacetylated SPAAC product + mono-acetylation (H3R52C**K56Ac: 15862 Da).

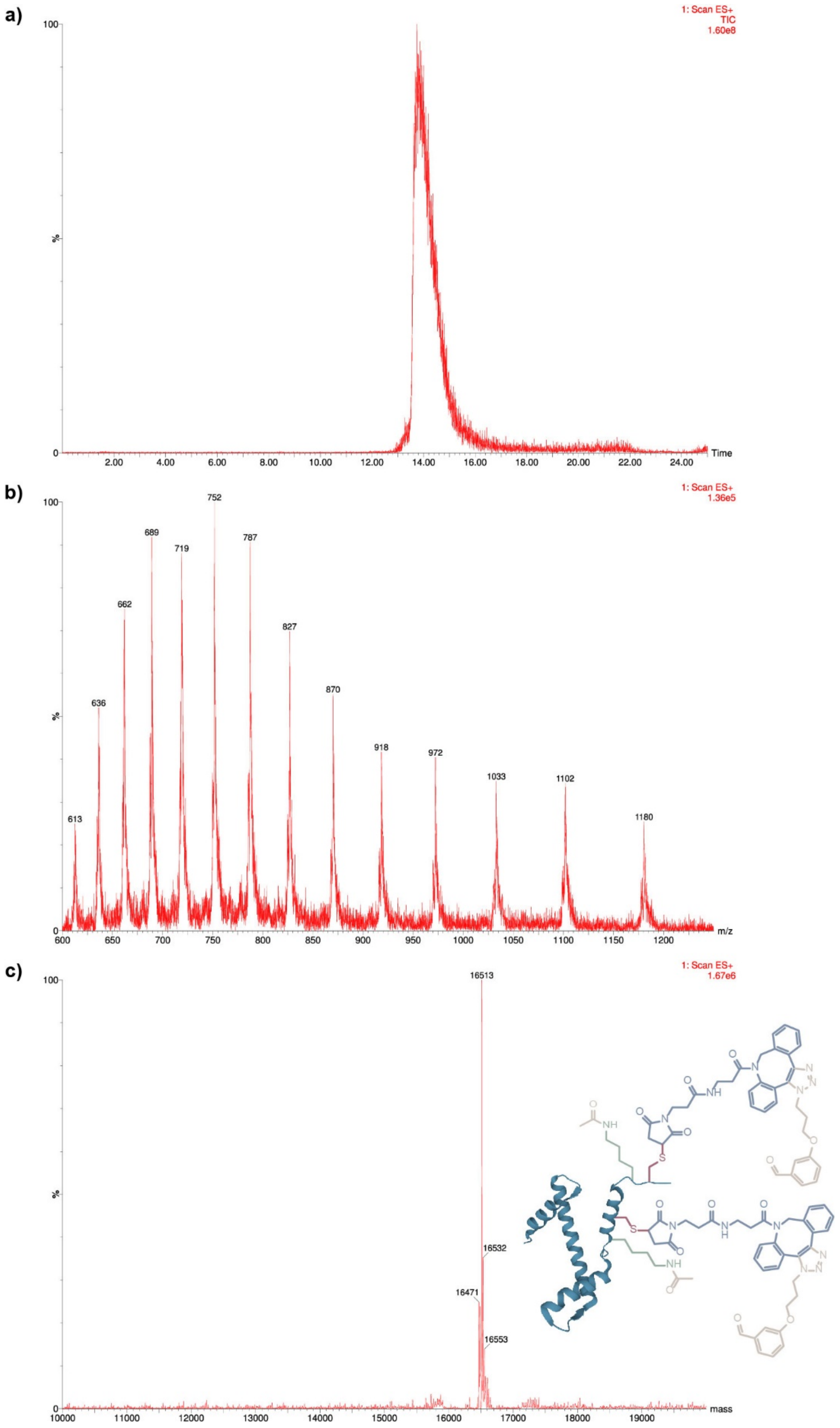

Figure S49. LC-MS of the H3K4C**K9AcR52C**K56Ac protein (25 µM) in water following spontaneous acetylation of unAcH3K4C**R52C** in ammonium acetate buffer (20 mM, pH 5.8) and desalting; **a)** total ion current chromatogram, **b)** ion series spectrum and **c)** deconvoluted spectrum with structural representation of the major product identified: Protein H3K4CR52C + two maleimide-DBCOs + two deacetylated SPAAC products + di-acetylation (H3K4C**K9AcR52C**K56Ac: 16513 Da).

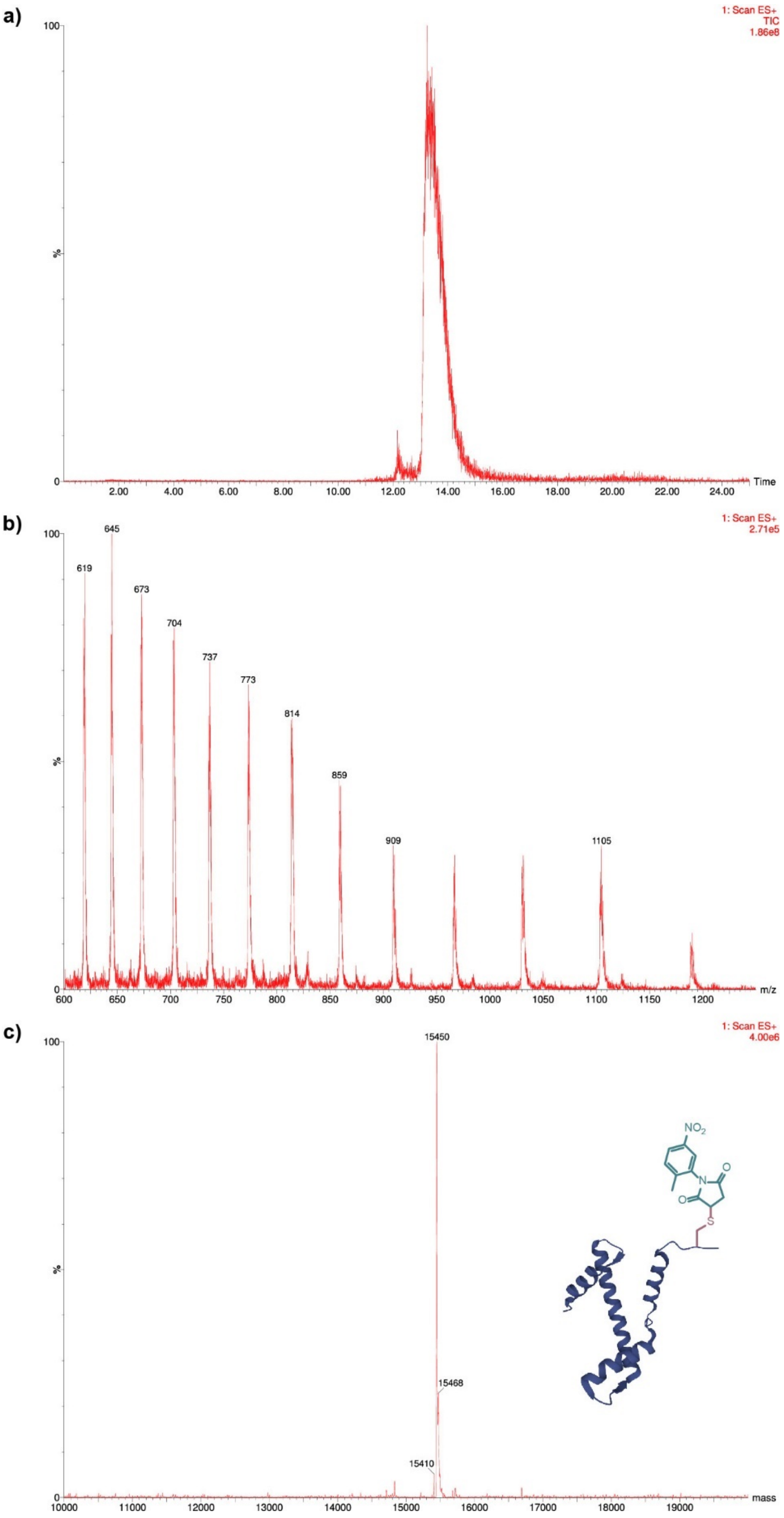

Figure S50. LC-MS of the reduced H3K4C protein (25 µM) following maleimide-dummy incubation (62.5 µM) in ammonium acetate buffer (20 mM, pH 7.0) for 1 h at 25 ºC and 400 rpm; **a)** total ion current chromatogram, **b)** ion series spectrum and **c)** deconvoluted spectrum with structural representation of the major product identified: Protein H3K4C + one maleimide-dummy (H3K4C-maleimide-dummy: 15450 Da).

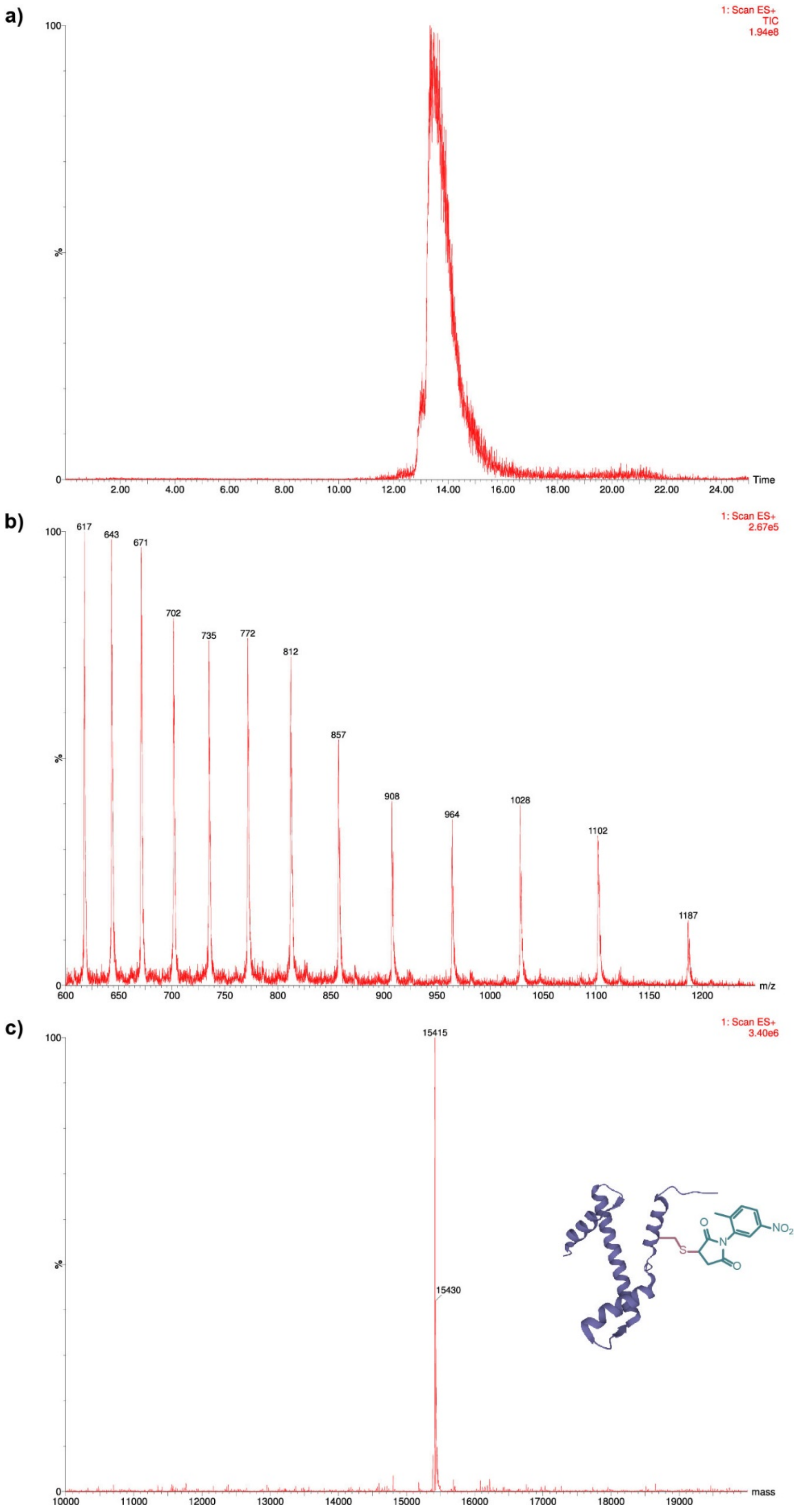

Figure S51. LC-MS of the reduced H3R52C protein (25 µM) following maleimide-dummy incubation (62.5 µM) in ammonium acetate buffer (20 mM, pH 7.0) for 1 h at 25 ºC and 400 rpm; **a)** total ion current chromatogram, **b)** ion series spectrum and **c)** deconvoluted spectrum with structural representation of the major product identified: Protein H3R52C + one maleimide-dummy (H3R52C-maleimide-dummy: 15415 Da).

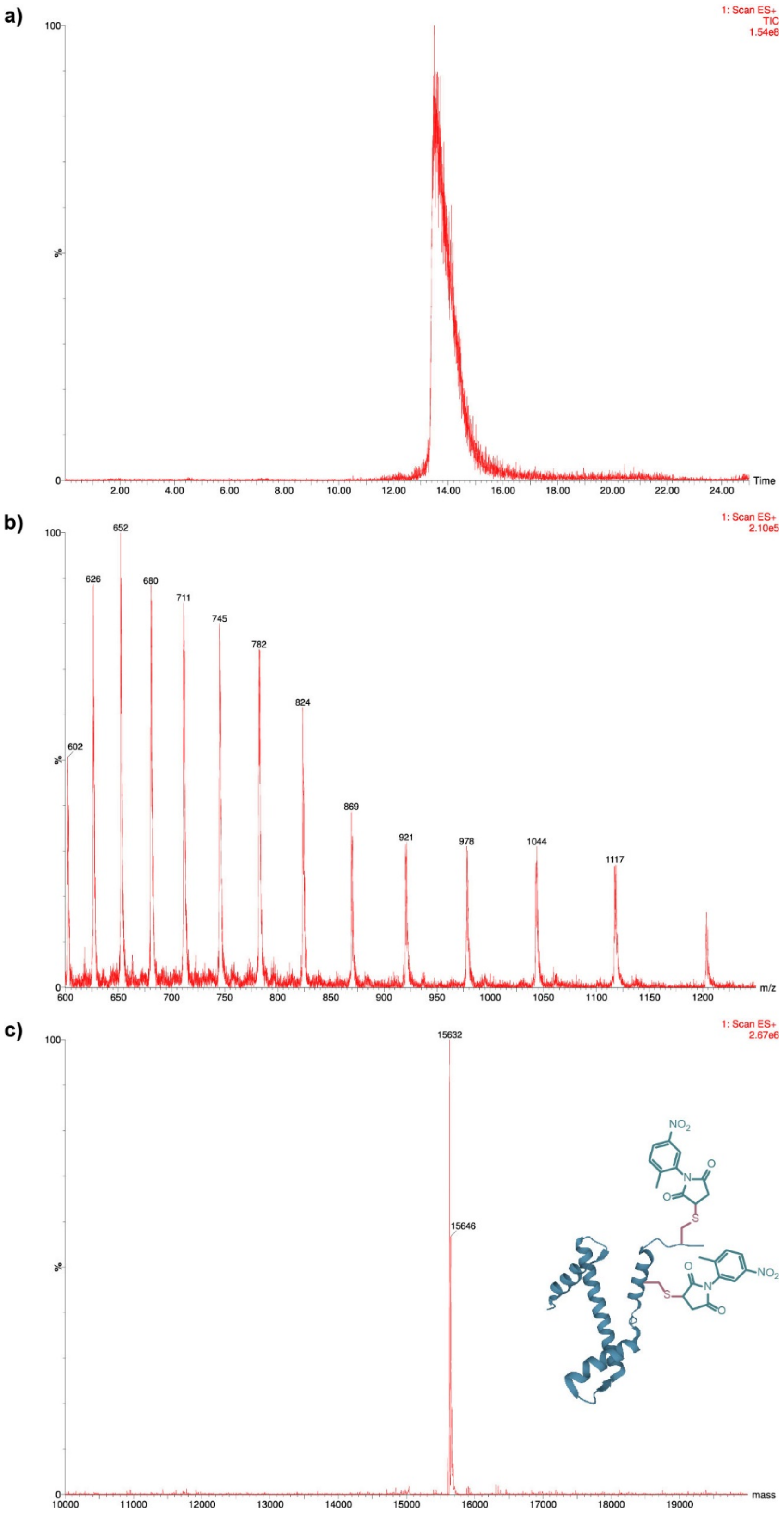

Figure S52. LC-MS of the reduced H3K4CR52C protein (25 µM) following maleimide-dummy incubation (62.5 µM) in ammonium acetate buffer (20 mM, pH 7.0) for 1 h at 25 ºC and 400 rpm; **a)** total ion current chromatogram, **b)** ion series spectrum and **c)** deconvoluted spectrum with structural representation of the major product identified: Protein H3K4CR52C + two maleimide-dummies (H3K4CR52C-maleimide-dummy: 15632 Da).

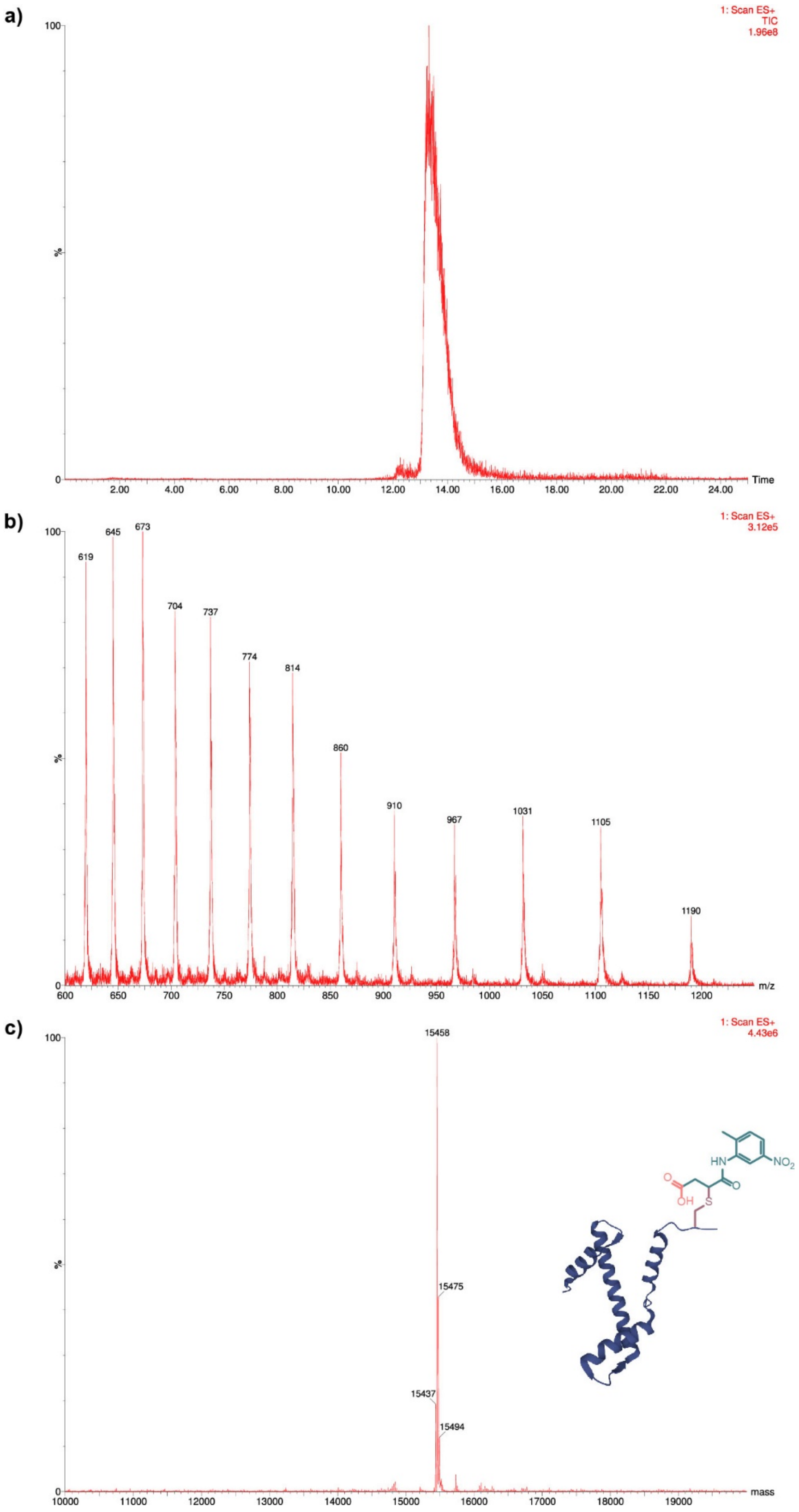

Figure S53. LC-MS of the H3K4C-maleimide-dummy protein (25 µM) diluted in ammonium acetate buffer (20 mM, pH 8.0); **a)** total ion current chromatogram, **b)** ion series spectrum and **c)** deconvoluted spectrum with structural representation of the major product identified: Protein H3K4C + one maleimide-dummy + one hydration (H3K4C-hydrolysed-maleimide-dummy: 15458 Da) – hydration most likely resulting from maleimide hydrolysis.

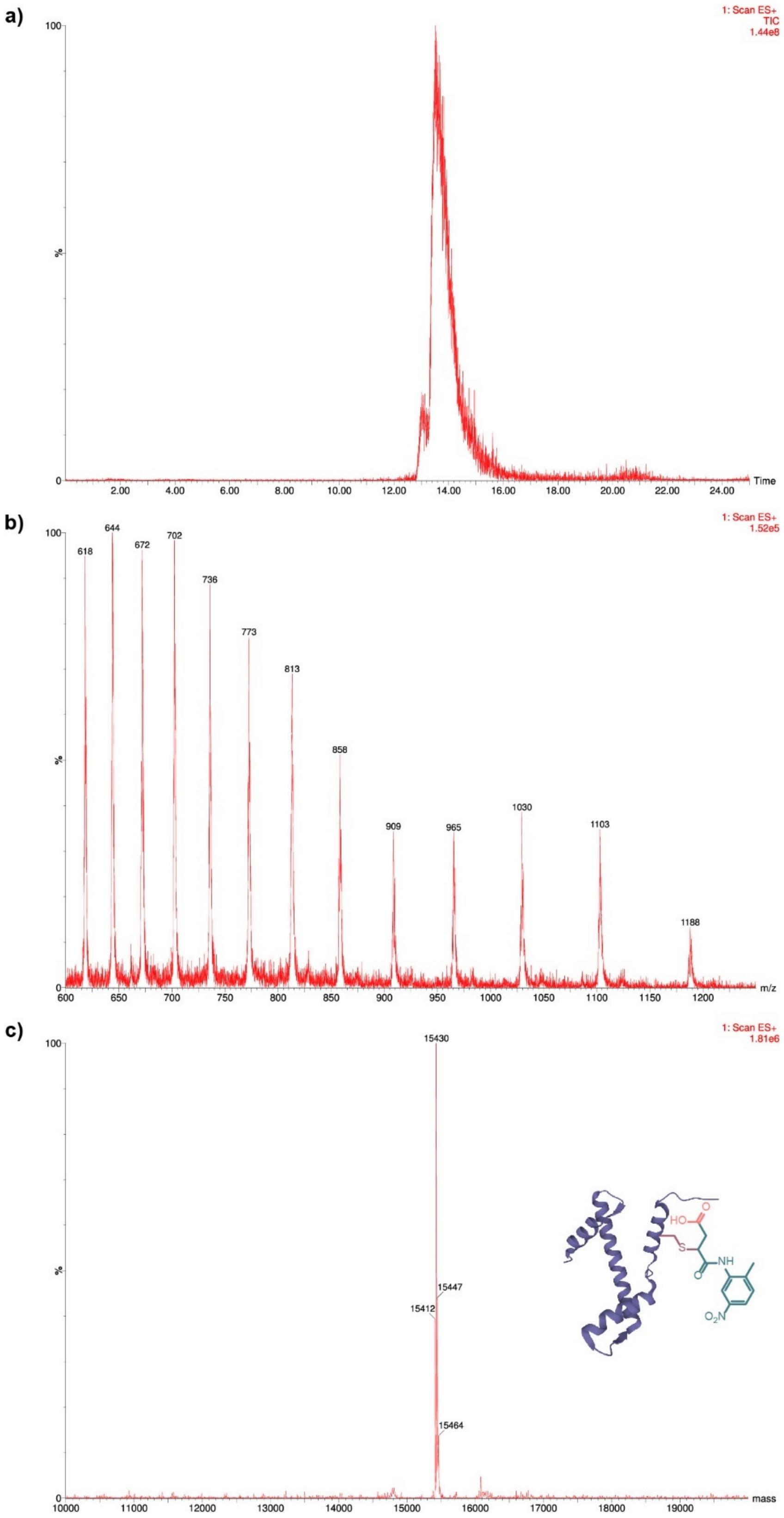

Figure S54. LC-MS of the H3R52C-maleimide-dummy protein (25 µM) diluted in ammonium acetate buffer (20 mM, pH 8.0); **a)** total ion current chromatogram, **b)** ion series spectrum and **c)** deconvoluted spectrum with structural representation of the major product identified: Protein H3R52C + one maleimide-dummy + one hydration (H3R52C-hydrolysed-maleimide-dummy: 15430 Da) – hydration most likely resulting from maleimide hydrolysis.

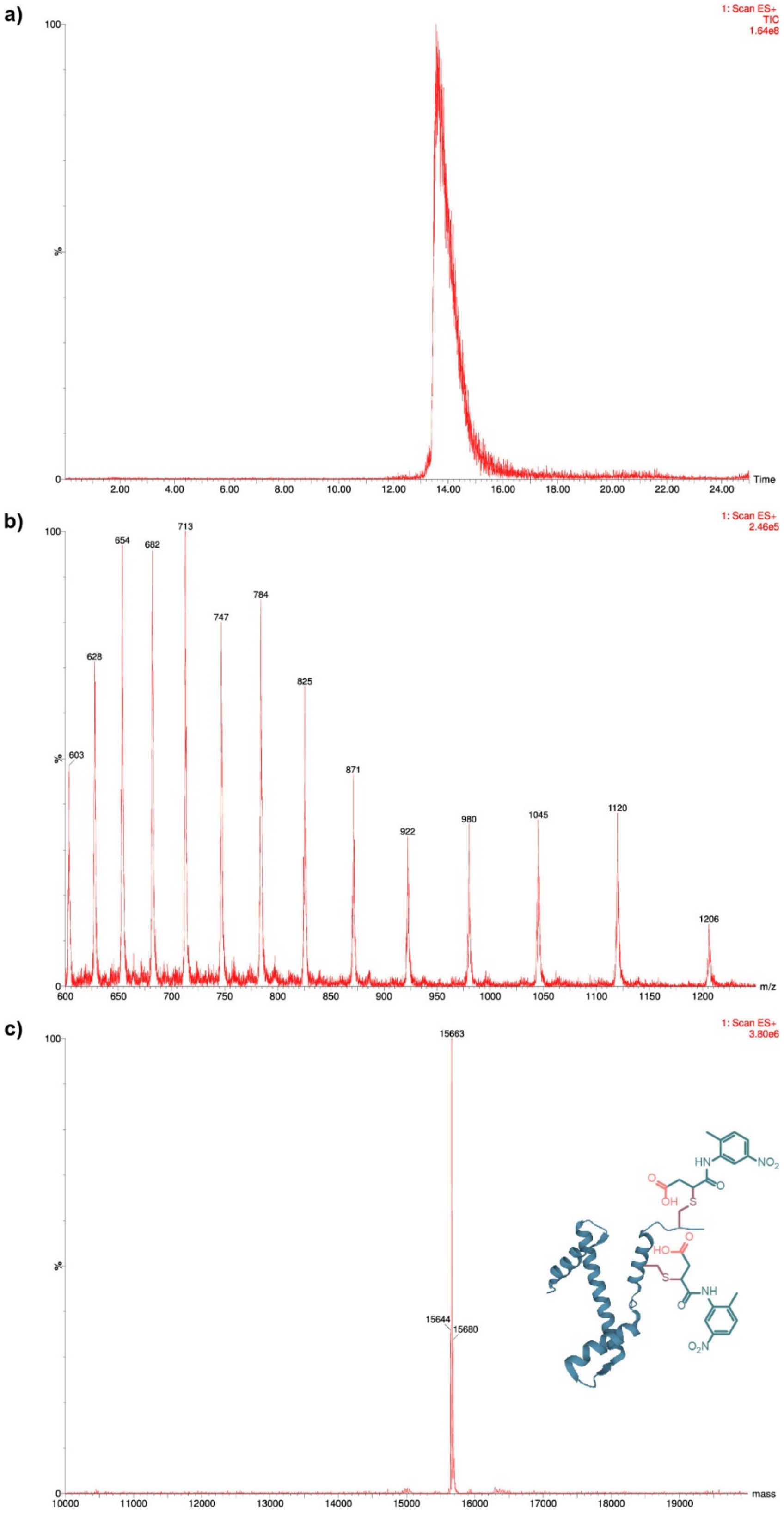

Figure S55. LC-MS of the H3K4CR52C-maleimide-dummy protein (25 µM) diluted in ammonium acetate buffer (20 mM, pH 8.0); **a)** total ion current chromatogram, **b)** ion series spectrum and **c)** deconvoluted spectrum with structural representation of the major product identified: Protein H3K4CR52C + two maleimide-dummies + two hydrations (H3K4CR52C-hydrolysed-maleimide-dummy: 15663 Da) – hydration most likely resulting from maleimide hydrolysis.

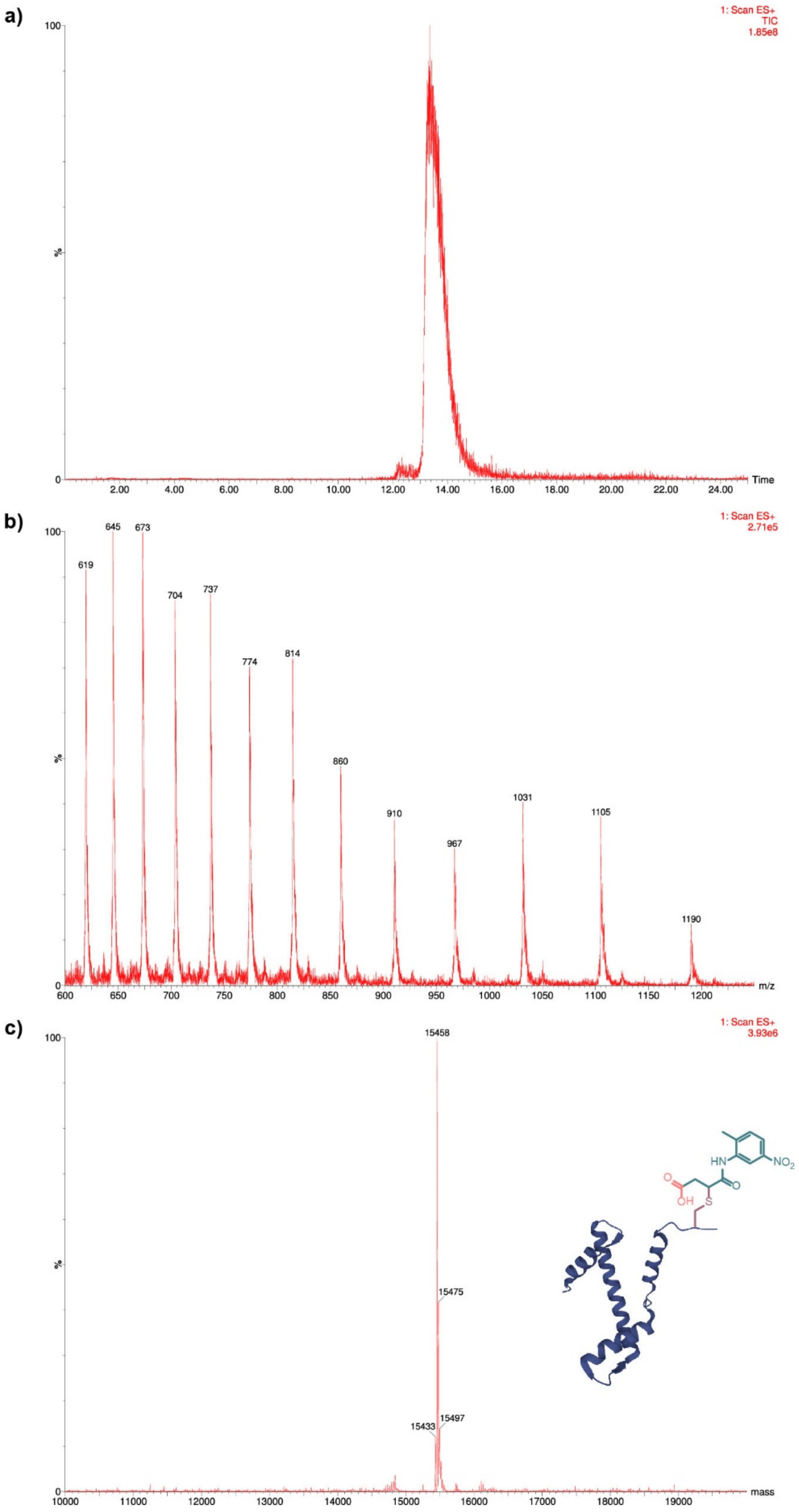

Figure S56. LC-MS of the H3K4C-hydrolysed-maleimide-dummy protein (25 µM) following incubation with compound **4** (100 µM) in ammonium acetate buffer (20 mM, pH 8.0) for 30 min at 25 ºC and 400 rpm; **a)** total ion current chromatogram, **b)** ion series spectrum and **c)** deconvoluted spectrum with structural representation of the major product identified: Protein H3K4C + one maleimide-dummy + one hydration (H3K4C-hydrolysed-maleimide-dummy: 15458 Da).

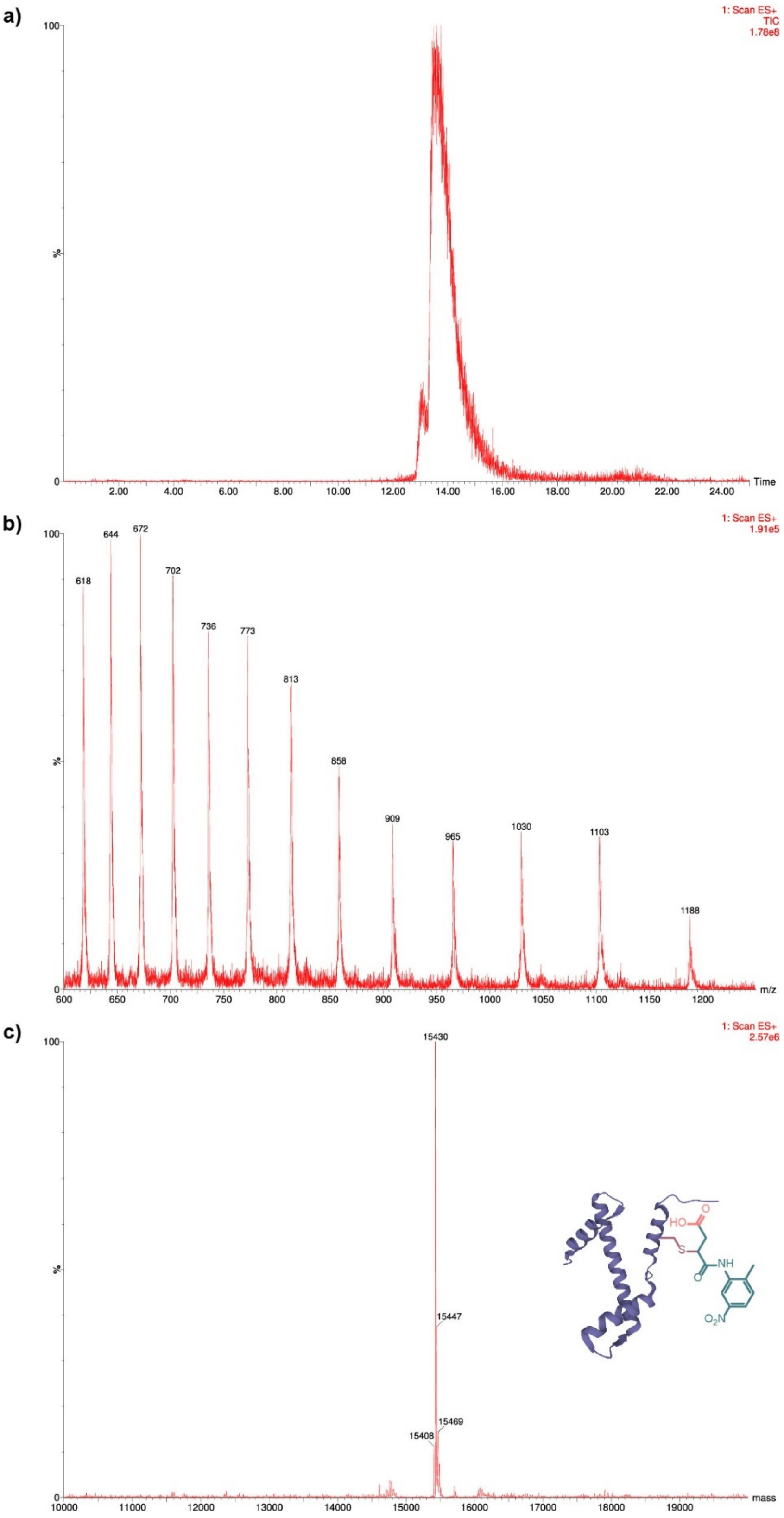

Figure S57. LC-MS of the H3R52C-hydrolysed-maleimide-dummy protein (25 µM) following incubation with compound **4** (50 µM) in ammonium acetate buffer (20 mM, pH 8.0) for 30 min at 25 ºC and 400 rpm; **a)** total ion current chromatogram, **b)** ion series spectrum and **c)** deconvoluted spectrum with structural representation of the major product identified: Protein H3R52C + one maleimide-dummy + one hydration (H3R52C-hydrolysed-maleimide-dummy: 15430 Da).

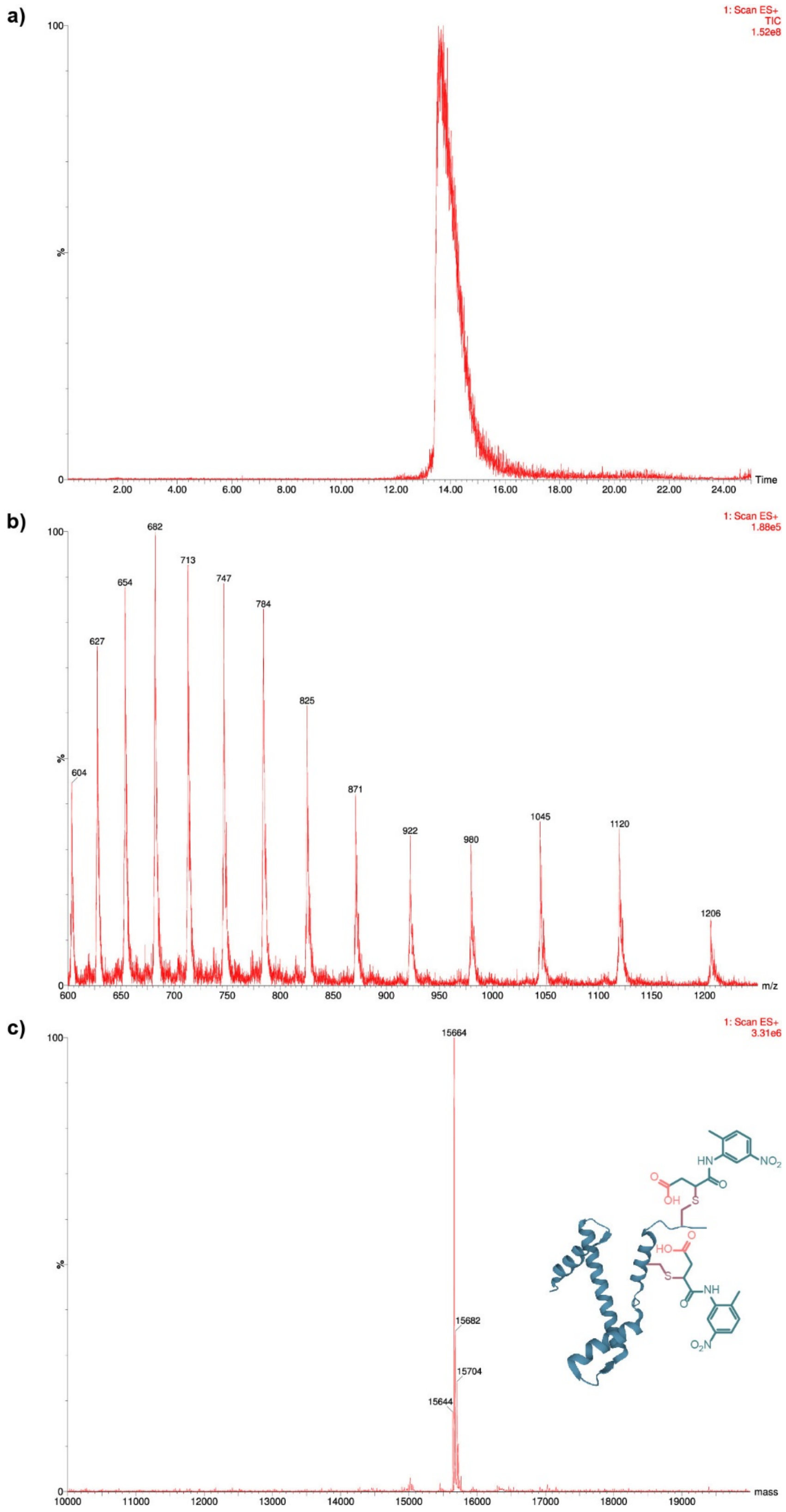

Figure S58. LC-MS of the H3K4CR52C-hydrolysed-maleimide-dummy protein (25 µM) following incubation with compound **4** (100 µM) in ammonium acetate buffer (20 mM, pH 8.0) for 45 min at 25 ºC and 400 rpm; **a)** total ion current chromatogram, **b)** ion series spectrum and **c)** deconvoluted spectrum with structural representation of the major product identified: Protein H3K4CR52C + two maleimide-dummies + two hydrations (H3K4CR52C-hydrolysed-maleimide-dummy: 15664 Da).

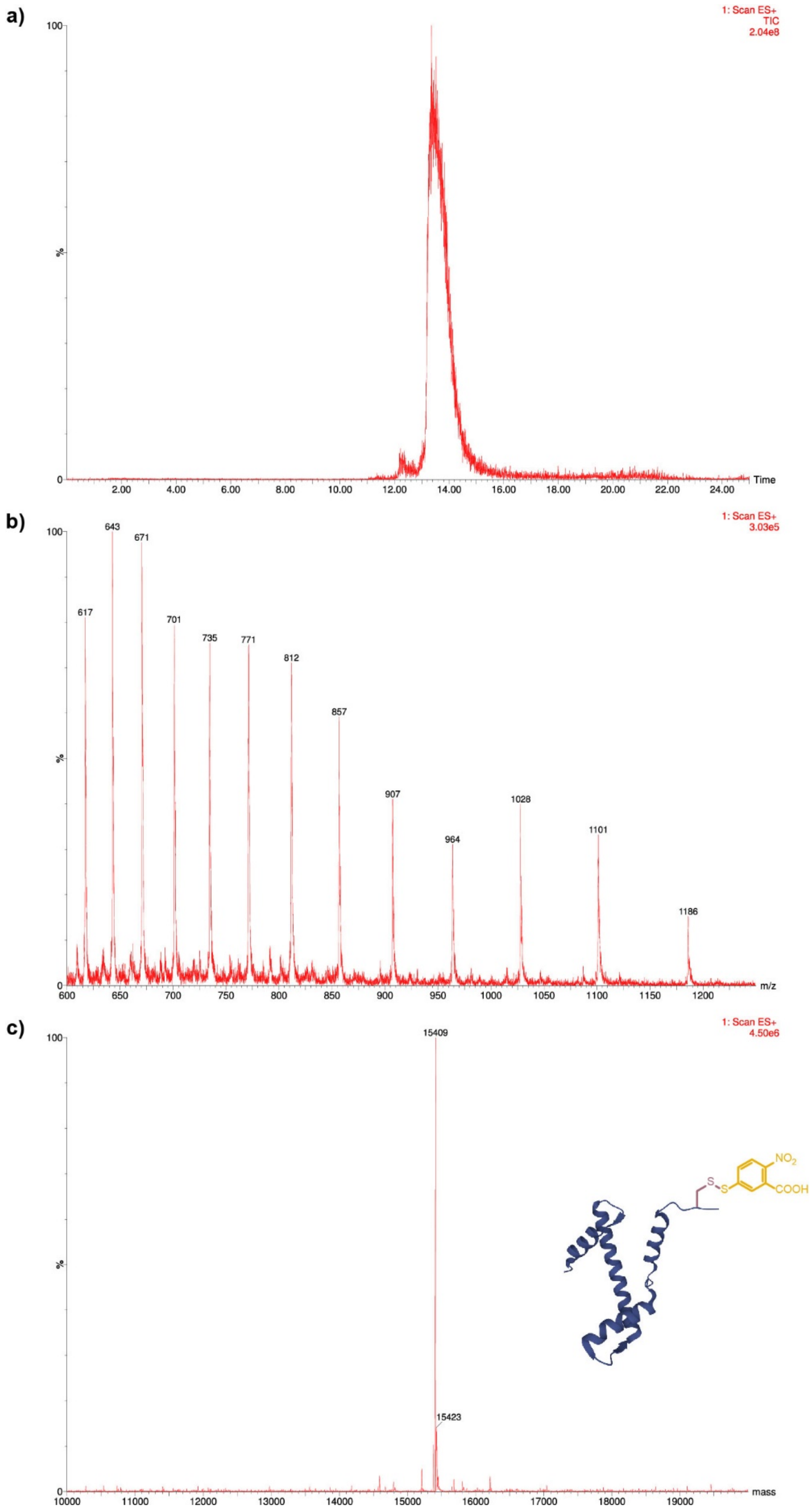

Figure S59. LC-MS of the reduced H3K4C protein (25 µM) following incubation with Ellman's reagent (250 µM) in ammonium acetate buffer (20 mM, pH 8.0) for 30 min at 25 ºC and 400 rpm; **a)** total ion current chromatogram, **b)** ion series spectrum and **c)** deconvoluted spectrum with structural representation of the major product identified: Protein H3K4C + ½ Ellman's reagent (H3K4C-Ellman's: 15409 Da).

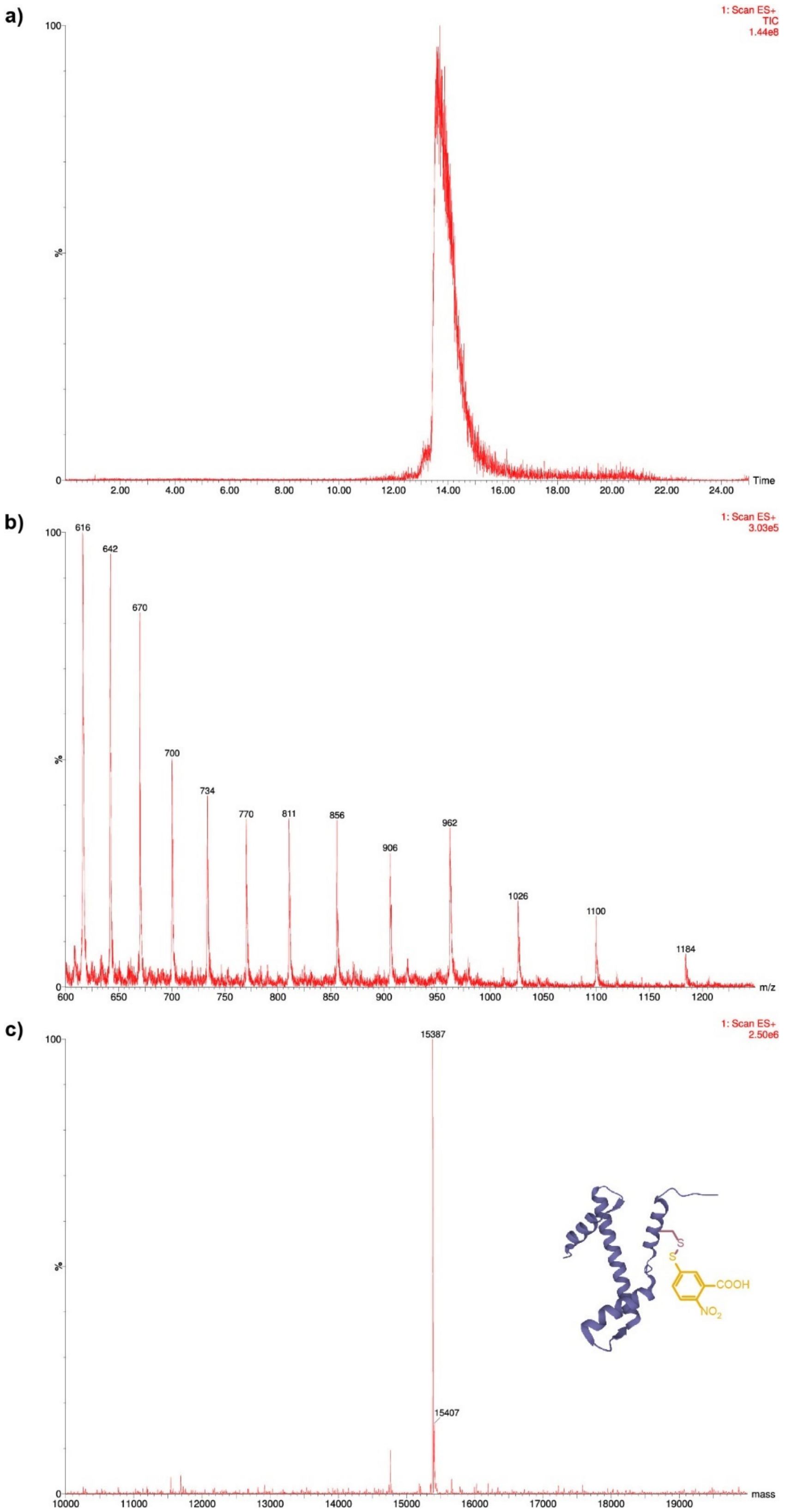

Figure S60. LC-MS of the reduced H3R52C protein (25 µM) following incubation with Ellman's reagent (250 µM) in ammonium acetate buffer (20 mM, pH 8.0) for 30 min at 25 ºC and 400 rpm; **a)** total ion current chromatogram, **b)** ion series spectrum and **c)** deconvoluted spectrum with structural representation of the major product identified: Protein H3R52C + ½ Ellman's reagent (H3R52C-Ellman's: 15387 Da).

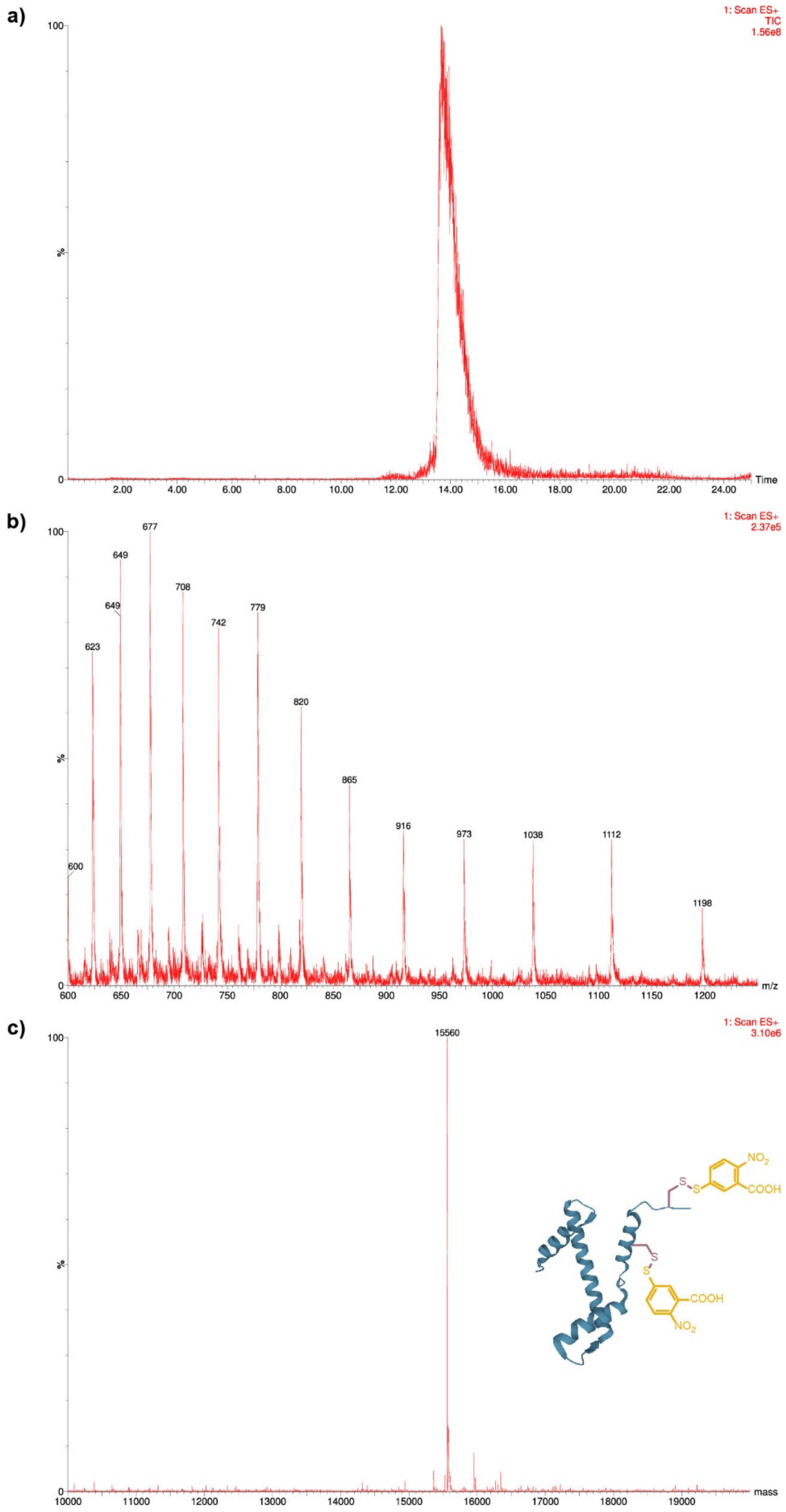

Figure S61. LC-MS of the reduced H3K4CR52C protein (25 µM) following incubation with Ellman's reagent (250 µM) in ammonium acetate buffer (20 mM, pH 8.0) for 30 min at 25 ºC and 400 rpm; **a)** total ion current chromatogram, **b)** ion series spectrum and **c)** deconvoluted spectrum with structural representation of the major product identified: Protein H3K4CR52C + one Ellman's reagent (H3K4CR52C-Ellman's: 15560 Da).

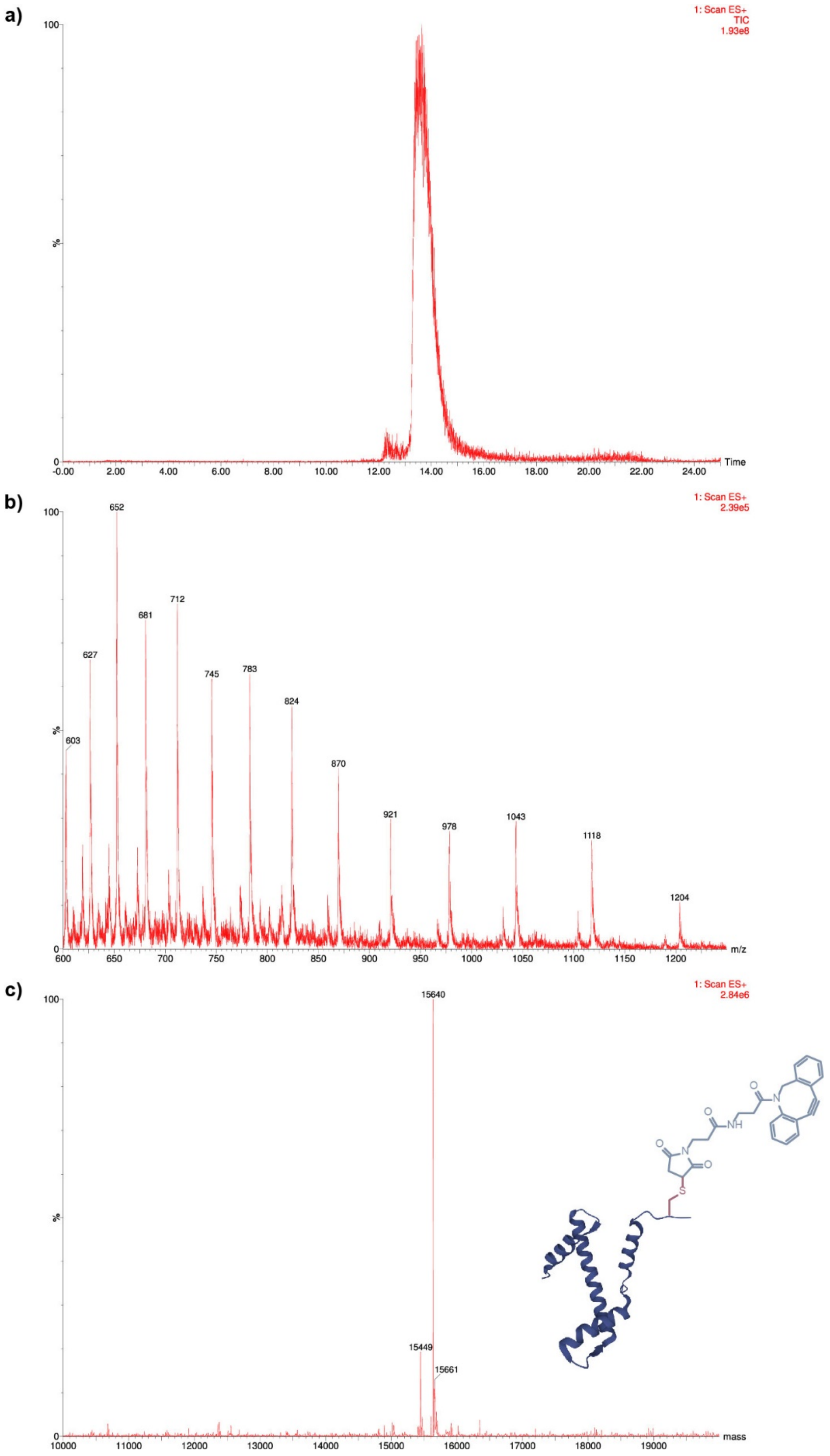

Figure S62. LC-MS of the H3K4C-maleimide-DBCO protein (25 µM) following incubation with Ellman's reagent (250 µM) in ammonium acetate buffer (20 mM, pH 8.0) for 30 min at 25 ºC and 400 rpm; **a)** total ion current chromatogram, **b)** ion series spectrum and **c)** deconvoluted spectrum with structural representation of the major product identified: Protein H3K4C + one maleimide-DBCO (H3K4C-maleimide-DBCO: 15640 Da).

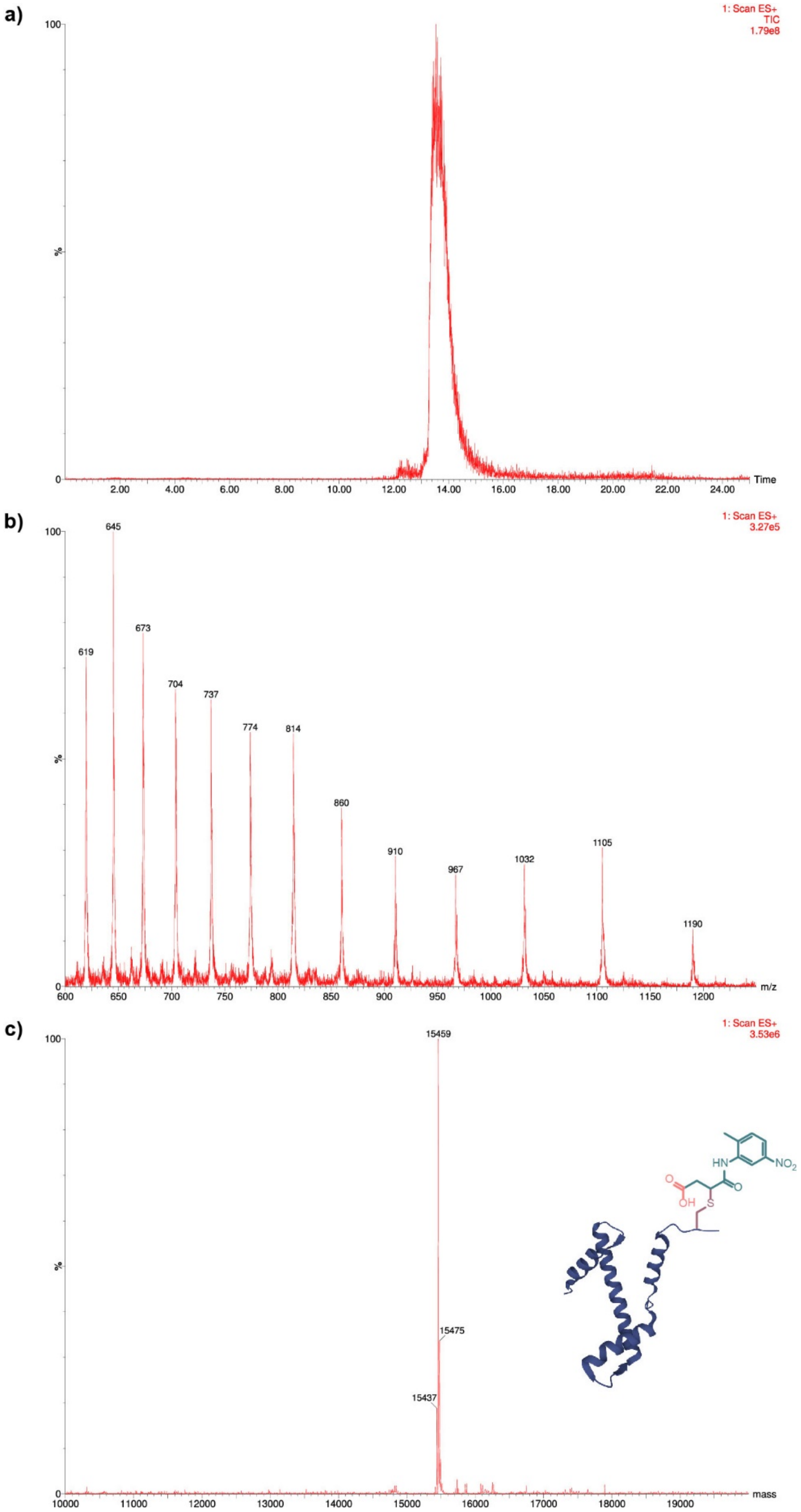

Figure S63. LC-MS of the H3K4C-hydrolysed-maleimide-dummy protein (25 µM) following incubation with Ellman's reagent (250 µM) in ammonium acetate buffer (20 mM, pH 8.0) for 30 min at 25 ºC and 400 rpm; **a)** total ion current chromatogram, **b)** ion series spectrum and **c)** deconvoluted spectrum with structural representation of the major product identified: Protein H3K4C + one maleimide-dummy + one hydration (H3K4C-hydrolysed-maleimide-dummy: 15459 Da).

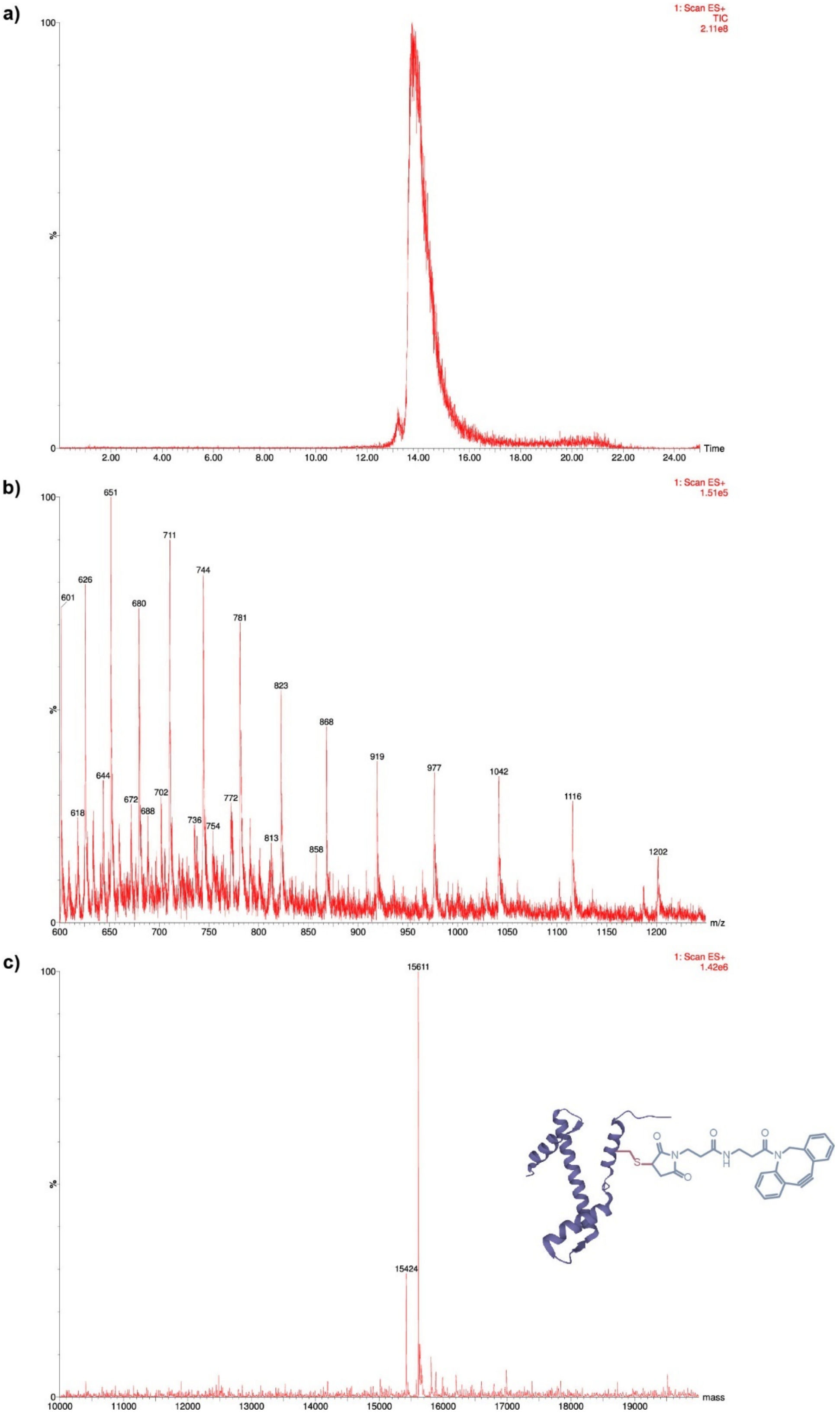

Figure S64. LC-MS of the H3R52C-maleimide-DBCO protein (25 μM) following incubation with Ellman's reagent (250 μM) in ammonium acetate buffer (20 mM, pH 8.0) for 30 min at 25 ºC and 400 rpm; **a)** total ion current chromatogram, **b)** ion series spectrum and **c)** deconvoluted spectrum with structural representation of the major product identified: Protein H3R52C + one maleimide-DBCO (H3R52C-maleimide-DBCO: 15611 Da).

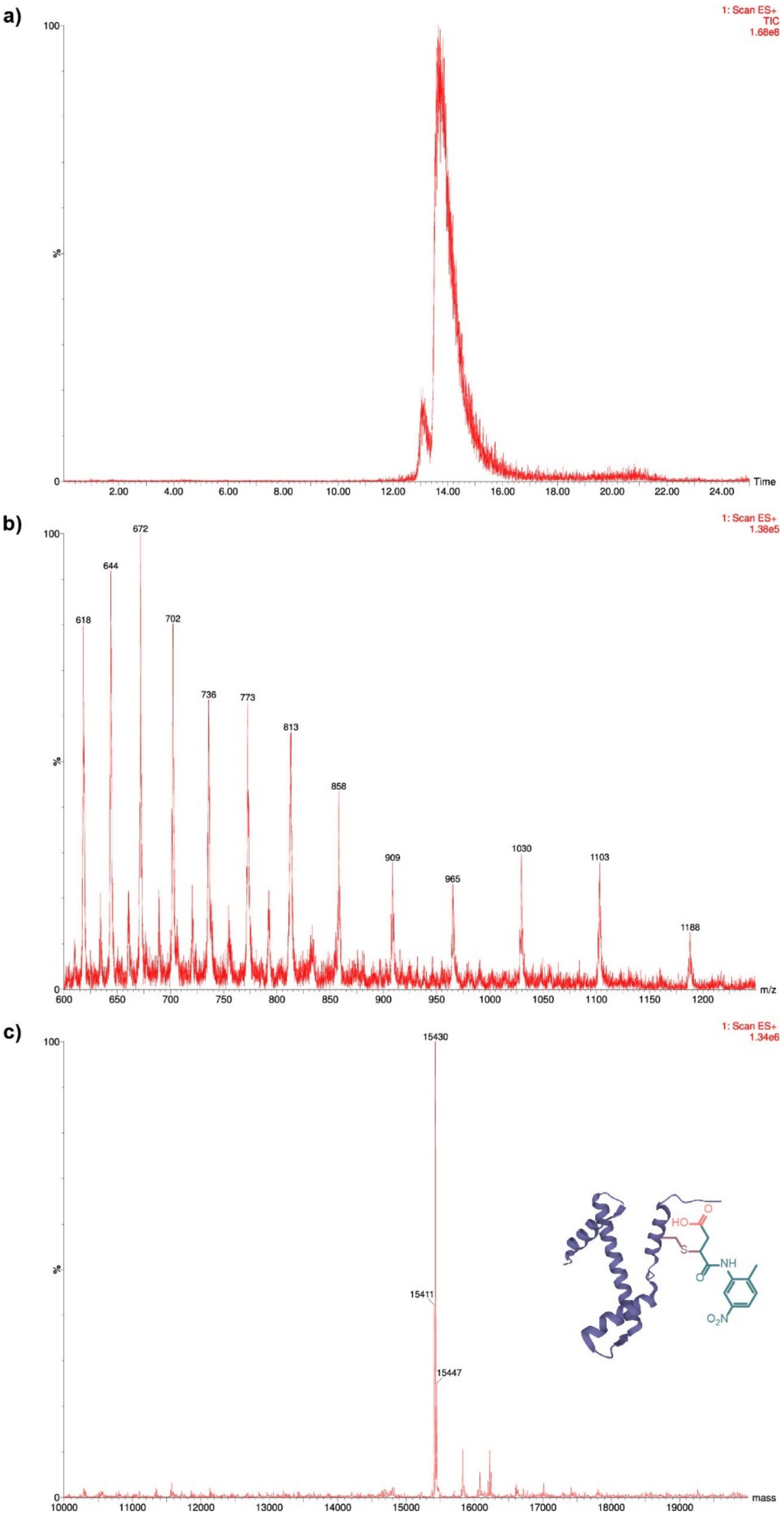

Figure S65. LC-MS of the H3R52C-hydrolysed-maleimide-dummy protein (25 µM) following incubation with Ellman's reagent (250 µM) in ammonium acetate buffer (20 mM, pH 8.0) for 30 min at 25 ºC and 400 rpm; **a)** total ion current chromatogram, **b)** ion series spectrum and **c)** deconvoluted spectrum with structural representation of the major product identified: Protein H3R52C + one maleimide-dummy + one hydration (H3R52C-hydrolysed-maleimide-dummy: 15430 Da).

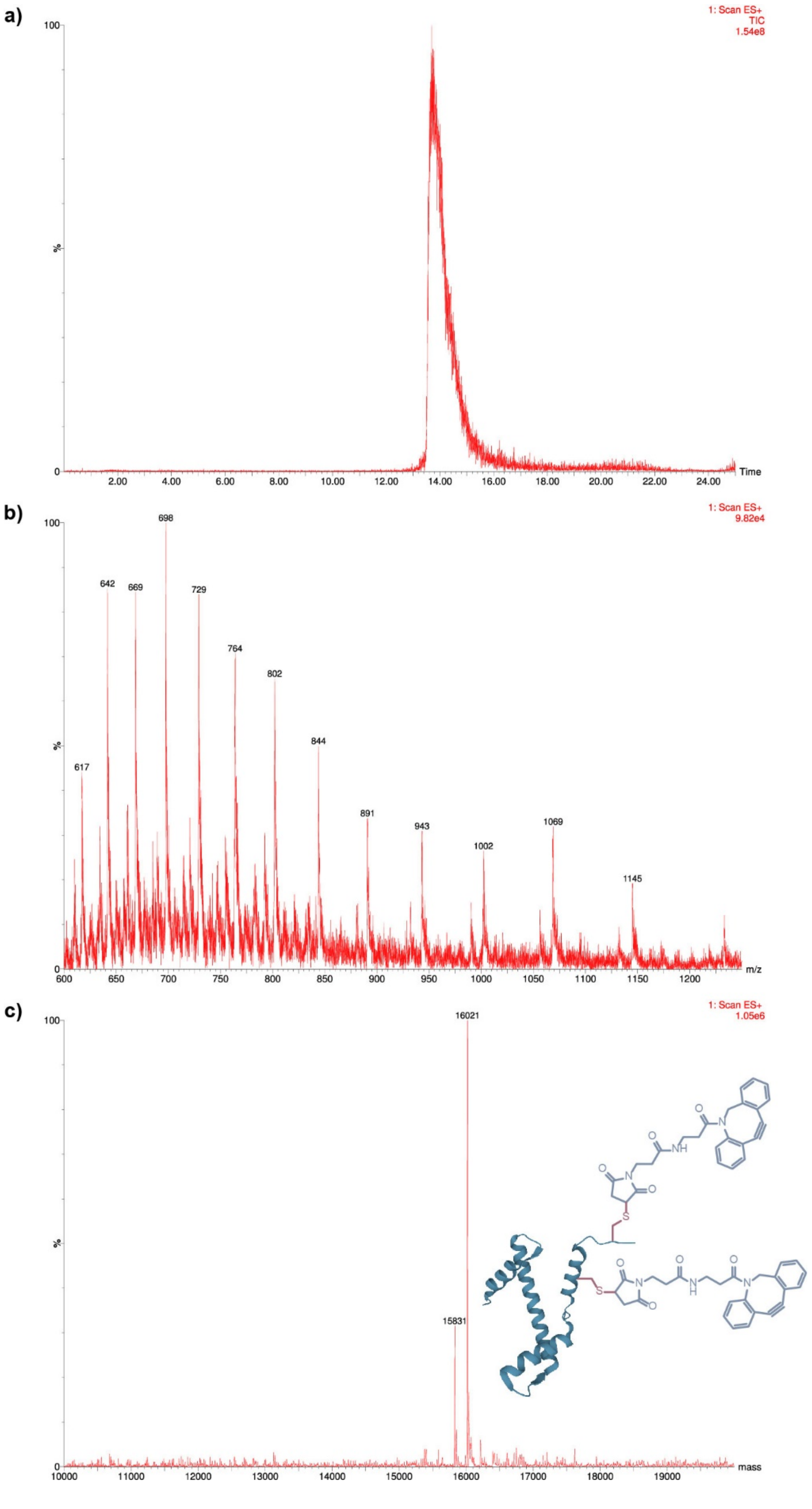

Figure S66. LC-MS of the H3K4CR52C-maleimide-DBCO protein (25 µM) following incubation with Ellman's reagent (250 µM) in ammonium acetate buffer (20 mM, pH 8.0) for 30 min at 25 ºC and 400 rpm; **a)** total ion current chromatogram, **b)** ion series spectrum and **c)** deconvoluted spectrum with structural representation of the major product identified: Protein H3K4CR52C + two maleimide-DBCOs (H3K4CR52C-maleimide-DBCO: 16021 Da).

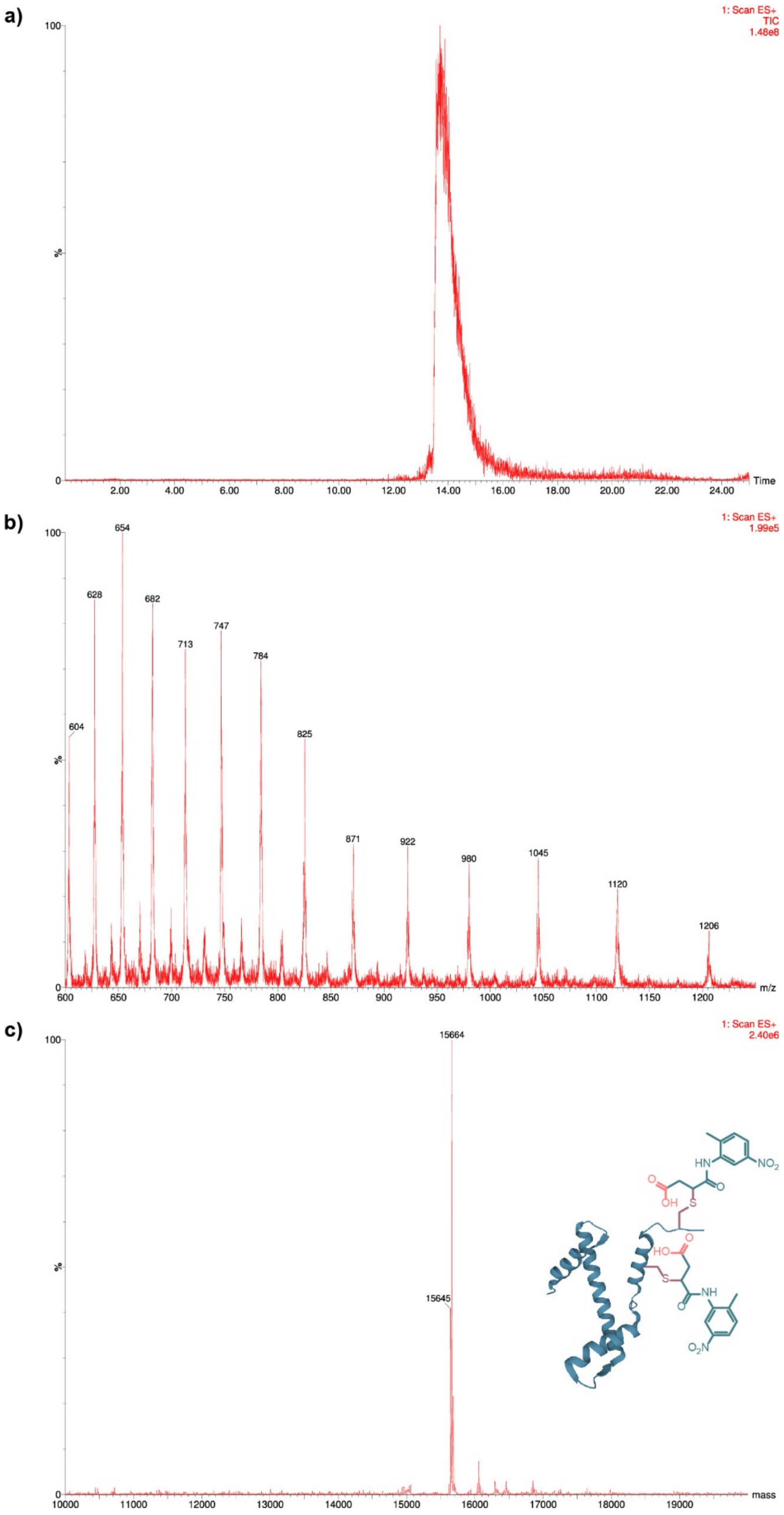

Figure S67. LC-MS of the H3K4CR52C-hydrolysed-maleimide-dummy protein (25 µM) following incubation with Ellman's reagent (250 µM) in ammonium acetate buffer (20 mM, pH 8.0) for 30 min at 25 ºC and 400 rpm; **a)** total ion current chromatogram, **b)** ion series spectrum and **c)** deconvoluted spectrum with structural representation of the major product identified: Protein H3K4CR52C + two maleimide-dummies + two hydrations (H3K4CR52C-hydrolysed-maleimide-dummy: 15664 Da).

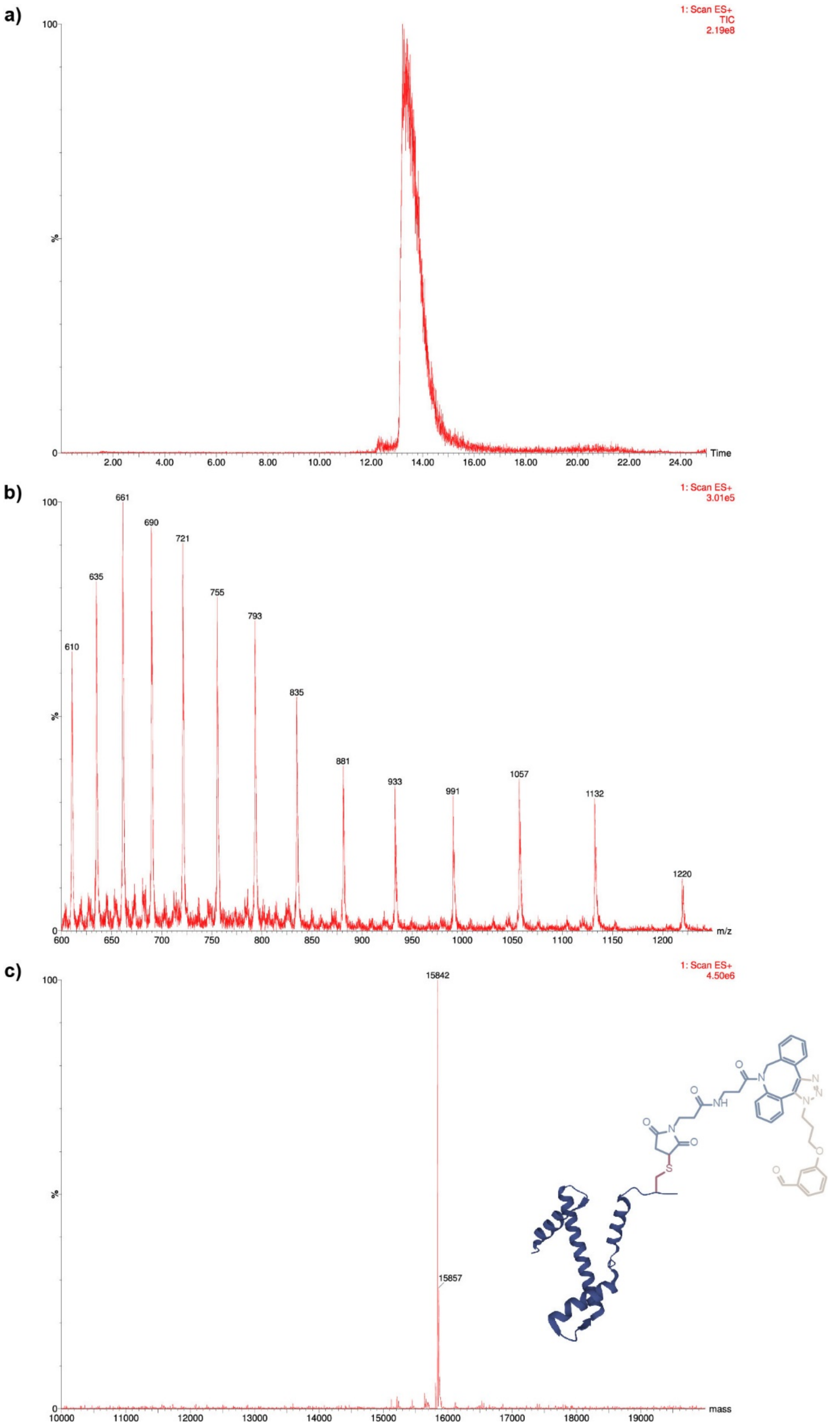

Figure S68. LC-MS of the H3K4C-maleimide-DBCO protein (25 µM) following incubation with compound **2** (100 µM) in ammonium acetate buffer (20 mM, pH 4.9) for 30 min at 25 ºC and 400 rpm; **a)** total ion current chromatogram, **b)** ion series spectrum and **c)** deconvoluted spectrum with structural representation of the major product identified: Protein H3K4C + one maleimide-DBCO + one deacetylated SPAAC product (H3K4C**: 15842 Da).

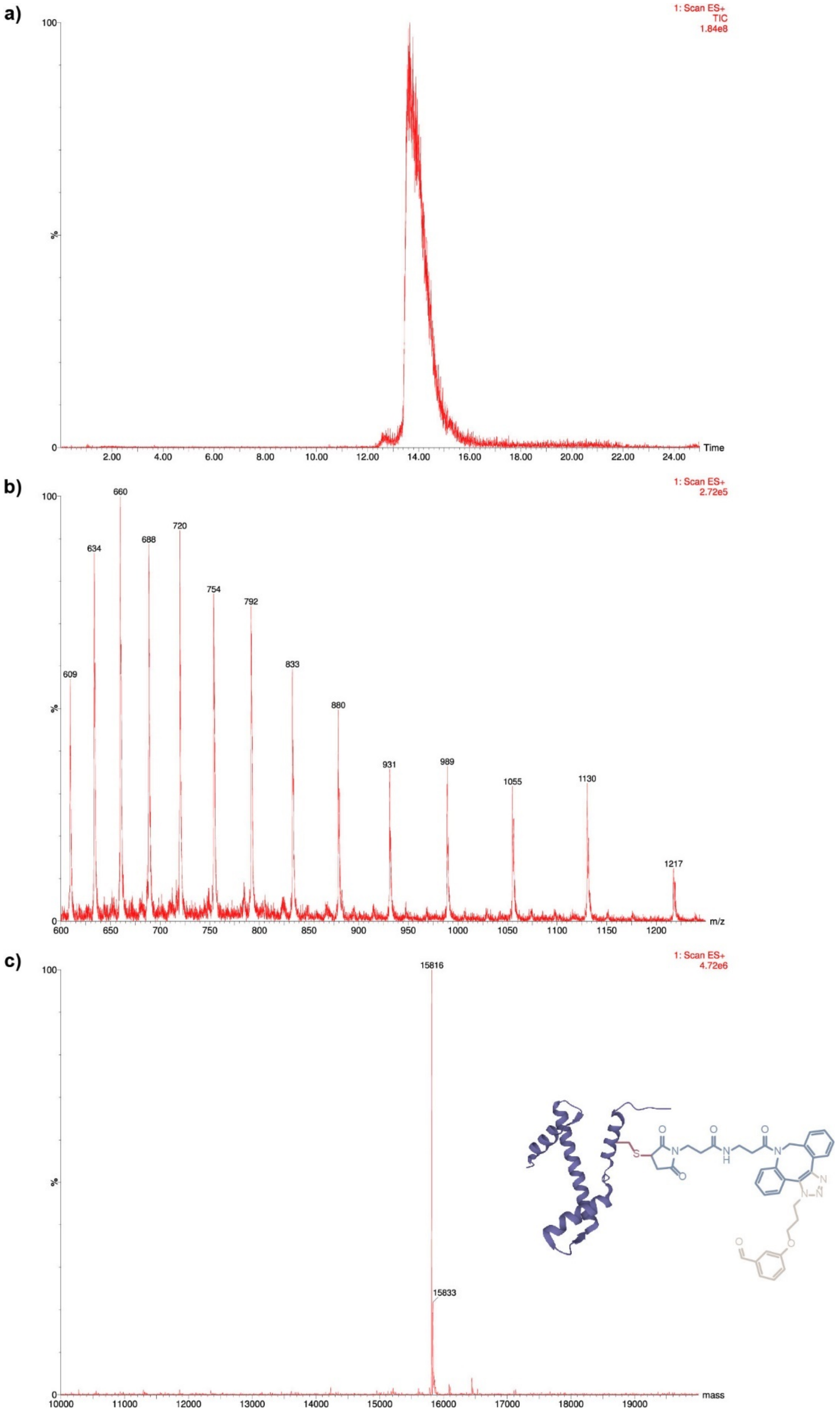

Figure S69. LC-MS of the H3R52C-maleimide-DBCO protein (25 µM) following incubation with compound **2** (50 µM) in ammonium acetate buffer (20 mM, pH 4.9) for 30 min at 25 ºC and 400 rpm; **a)** total ion current chromatogram, **b)** ion series spectrum and **c)** deconvoluted spectrum with structural representation of the major product identified: Protein H3R52C + one maleimide-DBCO + one deacetylated SPAAC product (H3R52C**: 15816 Da).

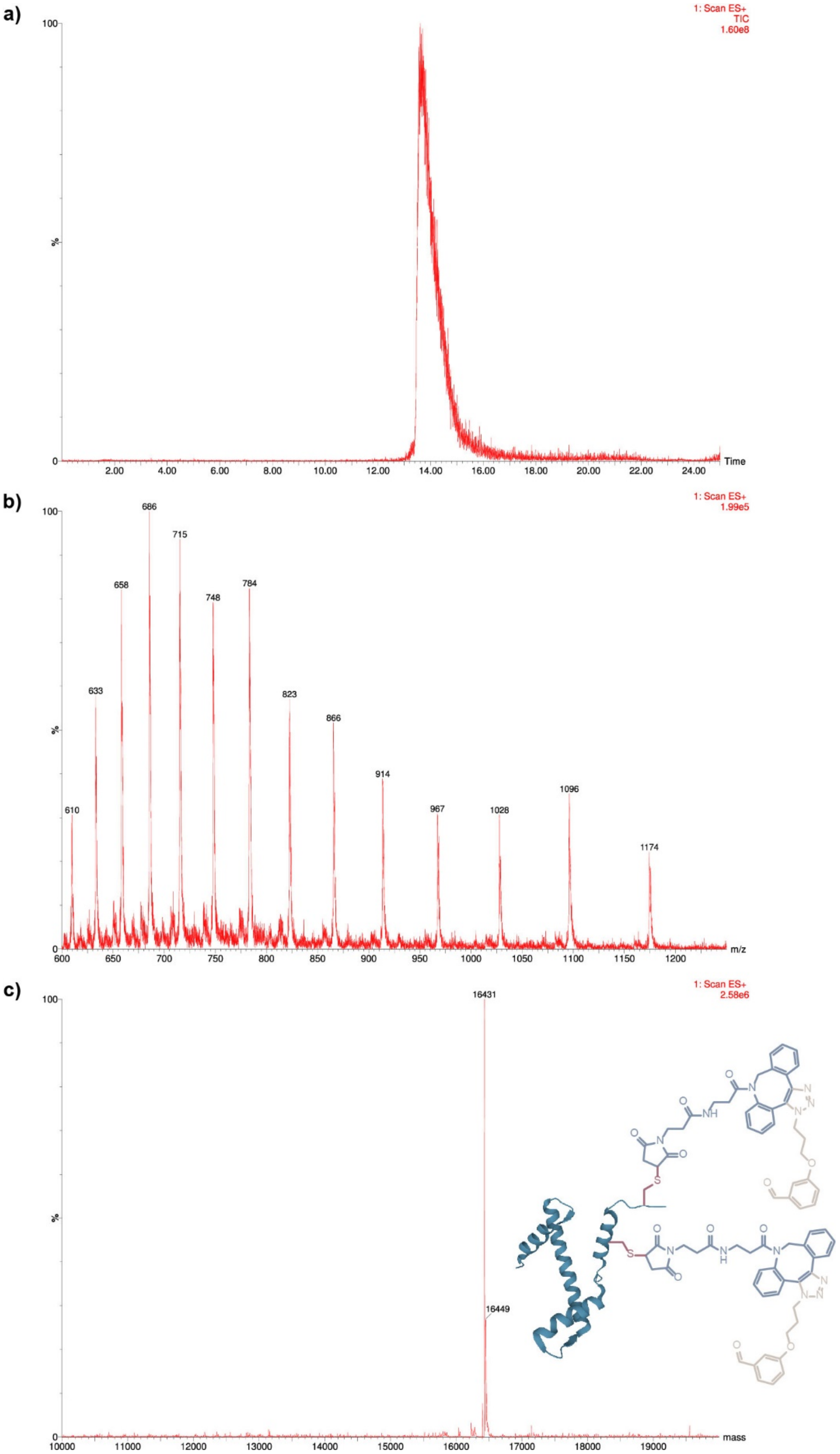

Figure S70. LC-MS of the H3K4CR52C-maleimide-DBCO protein (25 µM) following incubation with compound **2** (100 µM) in ammonium acetate buffer (20 mM, pH 4.9) for 30 min at 25 ºC and 400 rpm; **a)** total ion current chromatogram, **b)** ion series spectrum and **c)** deconvoluted spectrum with structural representation of the major product identified: Protein H3K4CR52C + two maleimide-DBCOs + two deacetylated SPAAC products (H3K4C**R52C**: 16431 Da).

6. Circular dichroism (CD) spectroscopy

Circular dichroism (CD) spectroscopy was performed to establish whether the H3K4C protein maintains its overall secondary structure following cysteine conjugation and subsequent SPAAC reaction. Lyophilized H3K4C, H3K4C** and H3K4C**K9Ac proteins were resuspended in milli-Q water and quantified using the Bradford assay. Samples were then diluted to 20 μM in filtered sodium phosphate buffer (20 mM, pH 7.4) immediately prior to CD analysis. Measurements were recorded using a JASCO spectropolarimeter J-815 (Tokyo, Japan) equipped with a PTC-423S/15 Peltier temperature control unit (25 °C). The data was acquired at a scanning speed of 200 nm per minute in a quartz cell with a 0.1 cm path length. The data integration time (DIT) was set to 2 s, the data pitch to 0.5 nm and the bandwidth to 1 nm. Spectra were averaged over five scans, in a wavelength range from 190 nm to 300 nm. The spectrum from a blank sample containing only buffer was subtracted from the averaged ellipticity data.

CD spectroscopy was also performed to evaluate whether differences exist in the secondary structure of the various H3 mutants generated in this work. Lyophilized H3K4C, H3R52C and H3K4CR52C proteins were resuspended in milli-Q water and quantified using the Bradford assay. Samples were then diluted to 10 μM in filtered sodium phosphate buffer (20 mM, pH 7.4) immediately prior to CD analysis. Measurements were recorded using a JASCO spectropolarimeter J-815 (Tokyo, Japan) equipped with a PTC-423S/15 Peltier temperature control unit (20 °C). The data was acquired at a scanning speed of 200 nm per minute in a quartz cell with a 0.1 cm path length. The data integration time (DIT) was set to 2 s, the data pitch to 0.5 nm and the bandwidth to 1 nm. Spectra were averaged over five scans, in a wavelength range from 190 nm to 300 nm. The spectrum from a blank sample containing only buffer was subtracted from the averaged ellipticity data.

CD raw data in ellipticity (θ) was normalized to the molar concentration of the samples and number of protein residues, according to the following equations:[4]

$$[\theta]_{molar,\lambda} = 100 \times \frac{\theta_\lambda}{m \times d}$$

$$[\theta]_{mrw,\lambda} = MRW \times \frac{\theta_\lambda}{10 \times d \times c}$$

where θ_λ is the observed ellipticity (in degrees) at wavelength λ, *m* is the molar concentration, *d* is the pathlength (cm) and *c* is the concentration (g/ml). *MRW* represents the mean residue weight and is equal to $M/(N-1)$, where *M* is the molecular weight (in Da) of the protein and *N* is the number of residues.

As shown in Figure S71, the CD profiles of the H3K4C, H3K4C** and H3K4C**K9Ac proteins are quite similar, indicating that the overall secondary structure of this histone mutant is maintained throughout the conjugation reactions. Furthermore, appreciable differences in the CD profiles were also not observed between the H3K4C, H3R52C and H3K4CR52C proteins, suggesting that all three mutants have similar secondary structures irrespective of having single or dual cysteine residues at positions 4 and/or 52.

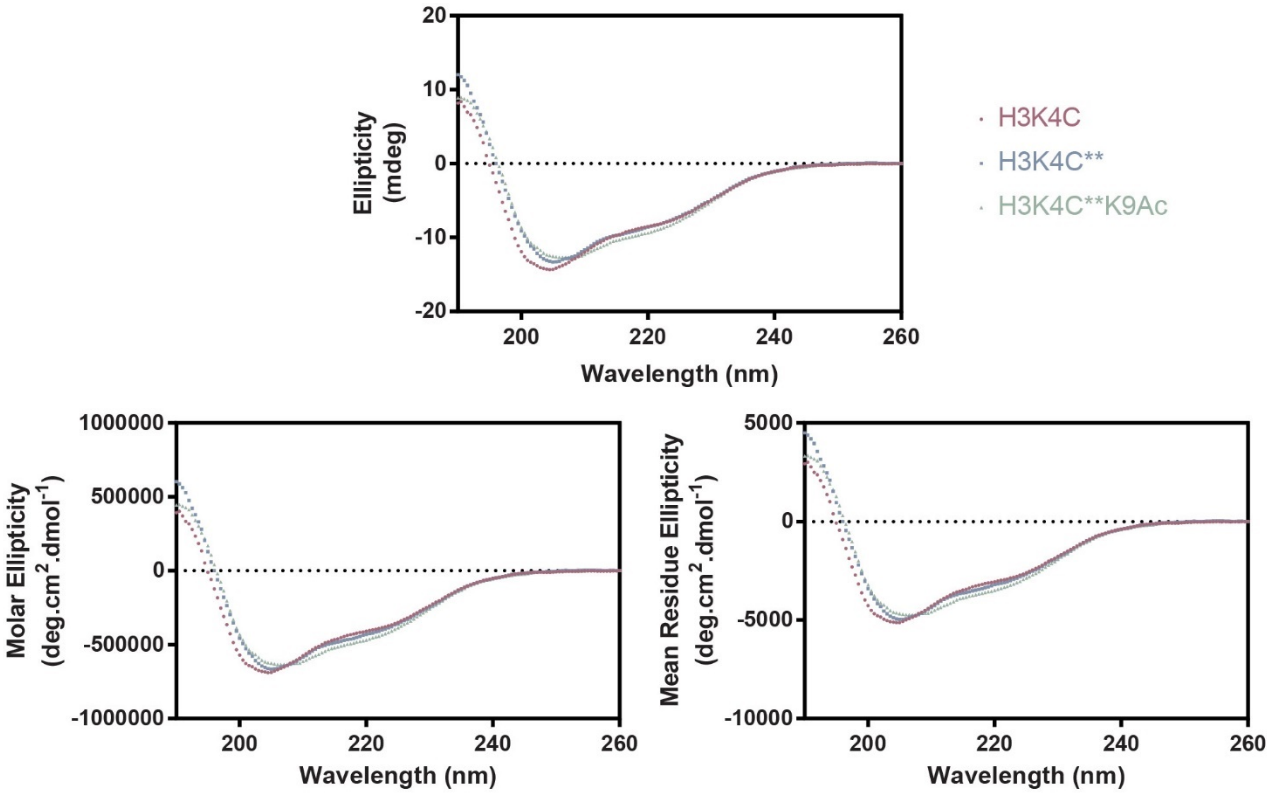

Figure S71. CD profiles of the H3K4C, H3K4C** and H3K4C**K9Ac proteins (20 μM) in sodium phosphate buffer (20 mM, pH 7.4) at 25 °C represented as ellipticity (θ), molar ellipticity ($[\theta]_{molar,\lambda}$) and mean residue ellipticity ($[\theta]_{mrw,\lambda}$).

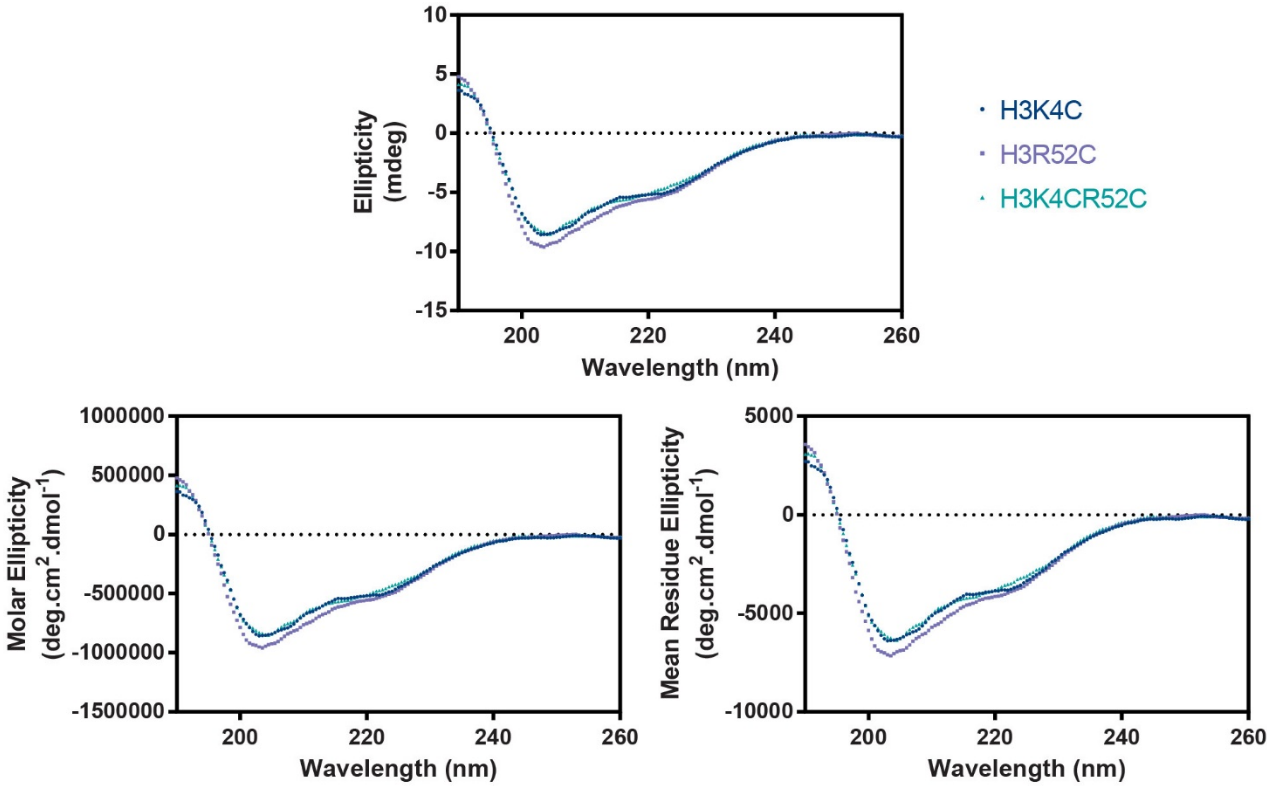

Figure S72. CD profiles of the H3K4C, H3R52C and H3K4CR52C proteins (10 µM) in sodium phosphate buffer (20 mM, pH 7.4) at 20 °C represented as ellipticity (θ), molar ellipticity ($[\theta]_{molar,\lambda}$) and mean residue ellipticity ($[\theta]_{mrw,\lambda}$).

7. Confirmation of site-specific acetylation in the H3K4C**K9Ac protein by MS/MS analysis

High resolution MS/MS was performed to confirm that acetylation was site-specific for position K9 of the modified H3K4C protein. To this end, a solution of the H3K4C**K9Ac protein was diluted to 5 μM in a 50:50 mixture of water: acetonitrile with 0.1% formic acid and injected. Data was acquired in a SolariX XR at 7 Tesla (25 1MB transients added per spectrum). For MS/MS spectra, ions were accumulated for 2 s in the external collision cell before CID using Ar (15V) or ECD (cathode current 1.5 A, 0.02 s pulse). Data was processed in Data Analysis 5.0 and monoisotopic masses calculated with the SNAP algorithm (Bruker Daltonics). MS/MS Data analysis was performed with Prosight Lite (The Kelleher Research Group, Pacific Northwest University).

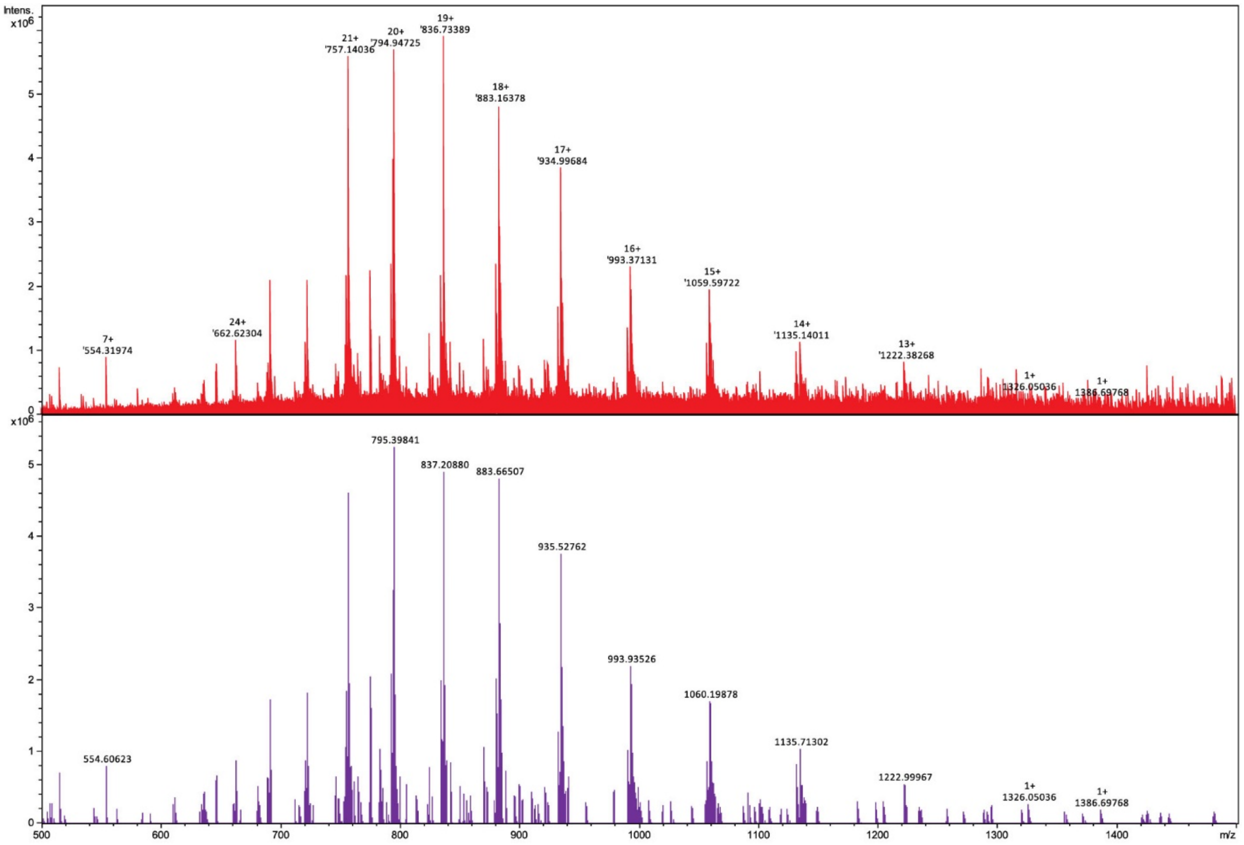

Figure S73. Broadband mass spectrum showing charge state distribution of the H3K4C**K9Ac protein (red measured; purple calculated).

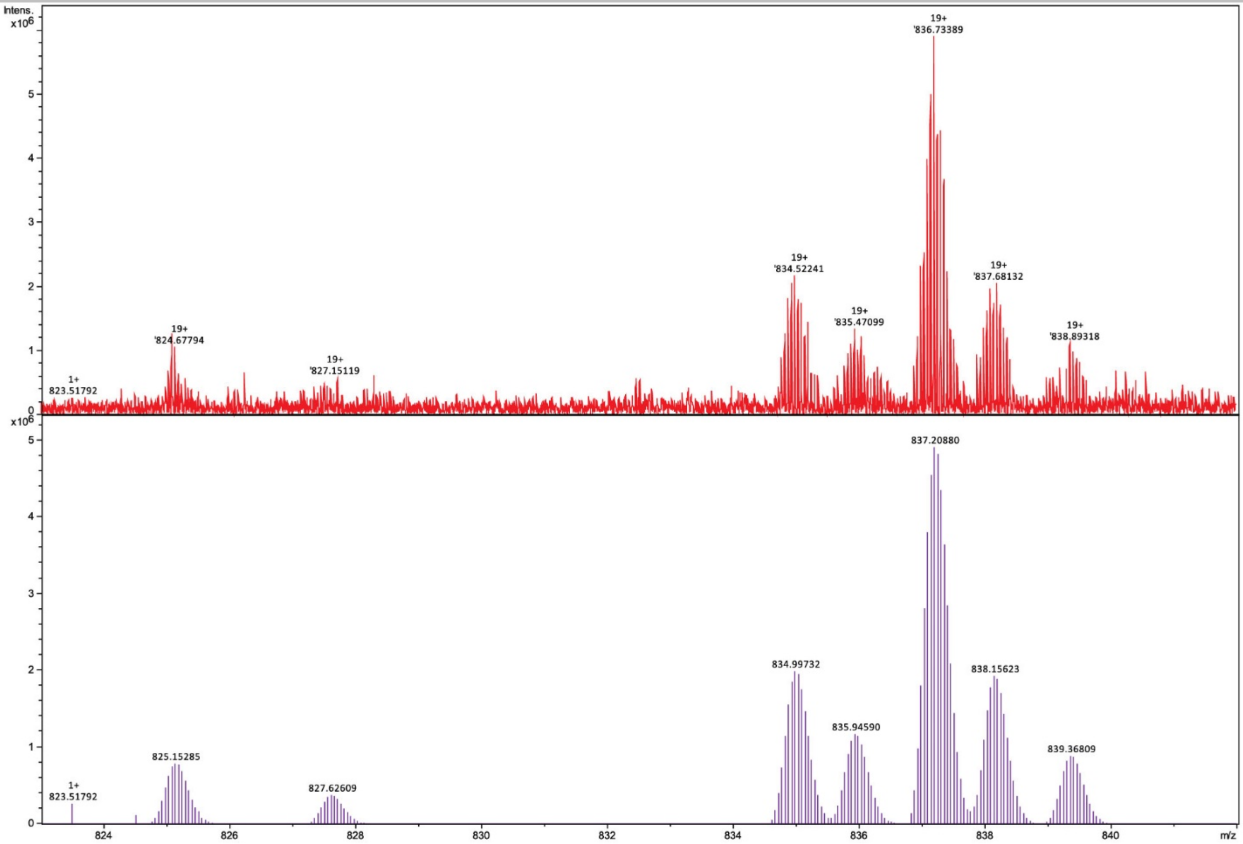

Figure S74. 19^+ charge state of the H3K4C**K9Ac protein, showing isotopic distribution of carbon (red measured; purple calculated). Seven major forms are present, corresponding to the molecular masses (single charged ions) indicated in Table S3. Monoisotopic mass of the neutral modified protein is 15877.665 Da according to the sequence and including the modification at C4 and one acetylation.

Table S3. Molecular masses and corresponding abundances of the major forms present in the injected sample

**Mass**	Abundance	Relative Abundance	Standard Deviation
15879.81181	24282774	100.00	0.017447
15837.80705	8987362	37.01	0.016178
15896.82382	8051163	33.16	0.023637
15650.73088	5443948	22.42	0.022362
15897.82785	5284286	21.76	0.024171
15917.81366	4674036	19.25	0.016074
15854.80362	2228403	9.18	0.019087

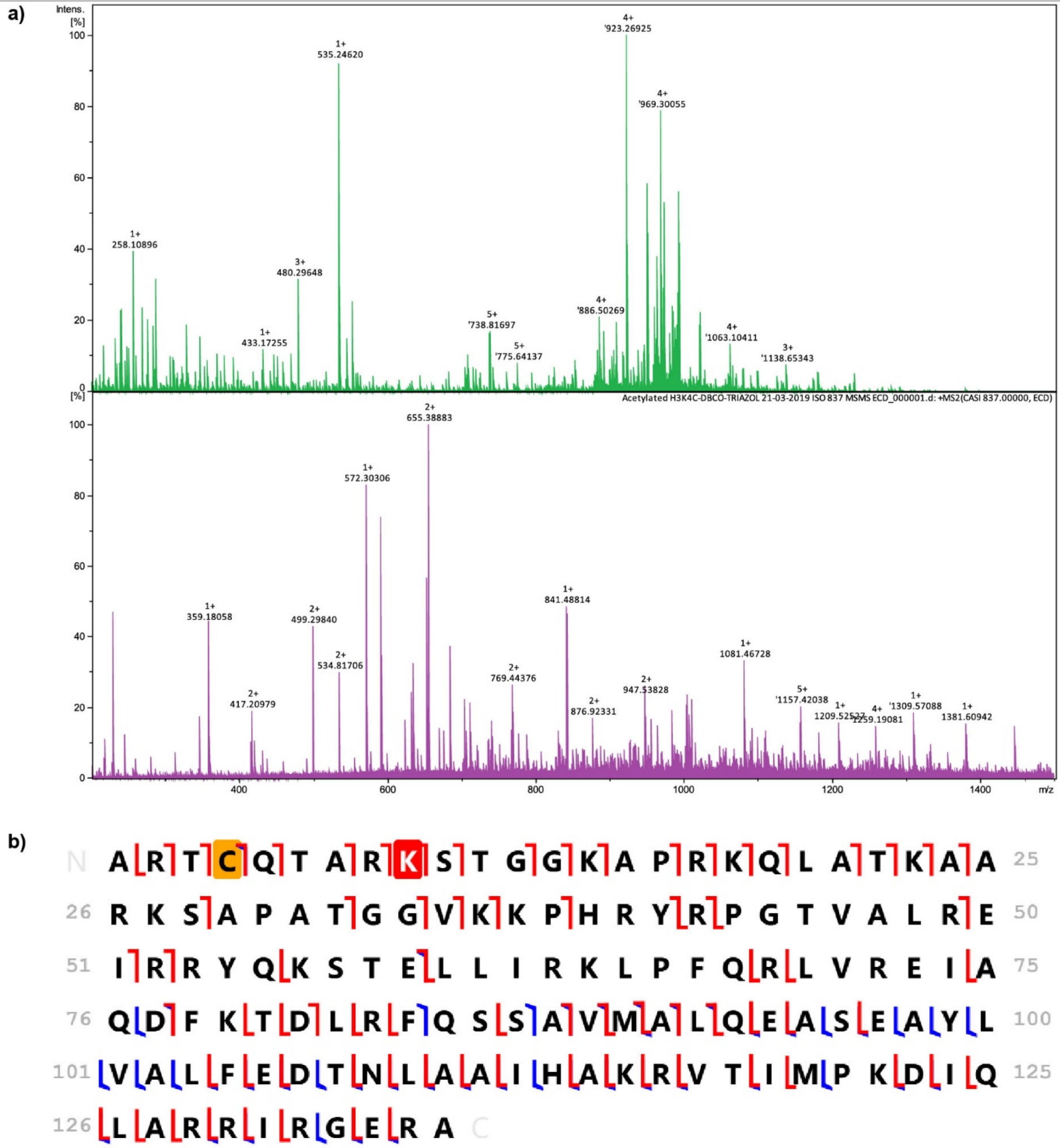

Figure S75. a) MS/MS spectra of the most abundant 19$^+$ charged ion corresponding to the H3K4C**K9Ac protein. CID (green) and ECD (purple) were used. Monoisotopic masses (single charged) were determined and combined before matching to the modified sequence; **b)** Predicted versus calculated mass difference is 0.134 Da or 8.5 ppm. Combining all fragment ions from CID (b, y, blue) and ECD (c, z, red) and matching the modified sequence with an allowed maximum deviation of 5 ppm, a sequence coverage of 67% was achieved comprising the modified C4 and K9 residues.

8. Western blot analysis of K9 and K56 acetylation

Western blot experiments were performed to establish whether the chemically acetylated H3K4C**K9Ac, H3R52C**K56Ac and H3K4C**K9AcR52C**K56Ac proteins can be recognized by antibodies raised against the natural acetyl-lysine at positions 9 and 56 of histone H3.

H3K4C**K9Ac

Sample preparation and SDS-PAGE

Lyophilized H3K4C, H3K4C** and H3K4C**K9Ac proteins were resuspended in milli-Q water and quantified using the Bradford assay. Two SDS-PAGE gels were prepared, one for Coomassie staining and another for Western Blot. Aliquots of the three proteins (400 ng/μL for Coomassie staining and 60 ng/μL for Western Blot) were diluted in Tris-HCl (20 mM, pH 7.5) and 5 μL of each were mixed with 2x Laemmli sample buffer. *β*-mercaptoethanol (0.5 μL) was added as the reducing agent and samples were boiled at 96 ºC for 4 min. Samples were loaded into a 15% SDS-PAGE gel (2 μg per well for Coomassie staining and 300 ng per well for Western Blot), together with the molecular weight marker (PageRuler^{TM} Plus Prestained Protein Ladder, 10 to 250 kDa, ThermoFisher).

Coomassie staining

After running in Tris-Glycine-SDS buffer, the first gel was fixed for 30 min with 40% of methanol in milli-Q water and then stained with InstantBlue Coomassie Stain (Abcam) for 1 h. Excess staining was removed with milli-Q water and the gel was imaged with Amersham Imager 680 RGB (GE Healthcare) (Figure S76).

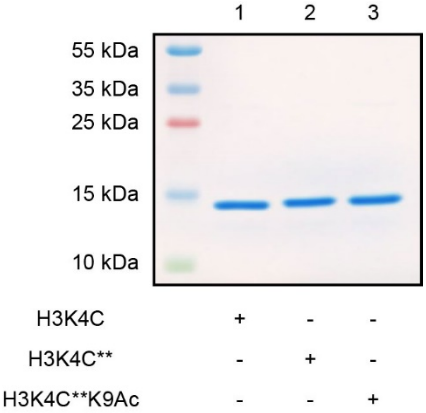

Figure S76. Coomassie staining of the 15% SDS-PAGE gel loaded with 2 μg each of H3K4C (lane 1), H3K4C** (lane 2) and H3K4C**K9Ac (lane 3) proteins. The protein ladder and the protein bands were visualized by colorimetric detection.

Membrane transfer and probing with the H3K9Ac antibody

After running the second gel in Tris-Glycine-SDS buffer, proteins were transferred to a membrane (Amersham Protran Supported Nitrocellulose 0.2 μm) in wet conditions with 20% methanol under constant current for 2 h at 200 mV. The membrane was then washed (1 x 5 min) in TBS (20 mM Tris HCl, 150 mM NaCl, pH 7.5) and then blocked with 5% non-fat dry milk in TBST (TBS with 0.05% tween-20) for 1 h at room temperature. The membrane was then washed with TBST (3 x 5 min) and incubated overnight at 4 ºC with primary antibody (1:6000, anti-H3K9Ac, rabbit monoclonal, C5B11, cat. # 9649S, Cell Signaling Technologies) diluted in 5% BSA in TBST. After washing with TBST (5 x 5 min), the membrane was then incubated at room temperature for 1 h with secondary antibody (1:10000, goat HRP-conjugated anti-rabbit IgG, Invitrogen) diluted in 5% non-fat dry milk in TBST. After washing with TBST (5 x 5 min) and TBS (1 x 5 min), the membrane was developed using the Clarity Western ECL Substrate (Bio-Rad) and imaged with Amersham Imager 680 RGB (GE Healthcare). A clear band with a molecular weight of approximately 15 kDa corresponding to the acetylated H3 histone at position K9 was observed in the lane loaded with H3K4C**K9Ac, while no bands were visible in the control lanes containing the H3K4C and H3K4C** proteins (Figure S77a).

Membrane stripping and probing with the H3 antibody

The membrane was re-probed with the H3 antibody, which served as a control to confirm that absence of the K9Ac acetylation signal in lanes 1 and 2 is not related to any technical issue (such as variations in protein loading between wells, uneven membrane transfer or antibody probing). Therefore, stripping (2 x 5 min) of the membrane was performed using an acidic buffer (200 mM glycine, 0.1 % (w/v) SDS, 1% (v/v) Tween-20, pH 2.2). After washing with PBS (2 x 10 min) and TBS (2 x 5 min), the membrane was blocked with 5% non-fat dry milk in TBST for 1 h at room temperature. The membrane was then washed with TBST (3 x 5 min) and incubated overnight

at 4 ºC with primary antibody (1:1000, anti-H3, mouse monoclonal, 1B1B2, cat. #14269S, Cell Signaling Technologies) diluted in 5% BSA in TBST (0.05% tween-20). After washing with TBST (6 x 5 min, 0.1% tween-20), the membrane was incubated at room temperature for 1 h with secondary antibody (1:5000, goat HRP-conjugated anti-mouse IgG H+L, Invitrogen) diluted in 5% non-fat dry milk in TBST (0.1% tween-20). After washing with TBST (7 x 5 min, 0.1% tween-20) and TBS (1 x 5 min), the membrane was developed using the Clarity Western ECL Substrate (Bio-Rad) and imaged with Amersham Imager 680 RGB (GE Healthcare). Clear bands with equivalent densitometry values and a molecular weight of roughly 15 kDa corresponding to histone H3 were observed in the lanes loaded with H3K4C, H3K4C** and H3K4C**K9Ac (Figure S77b).

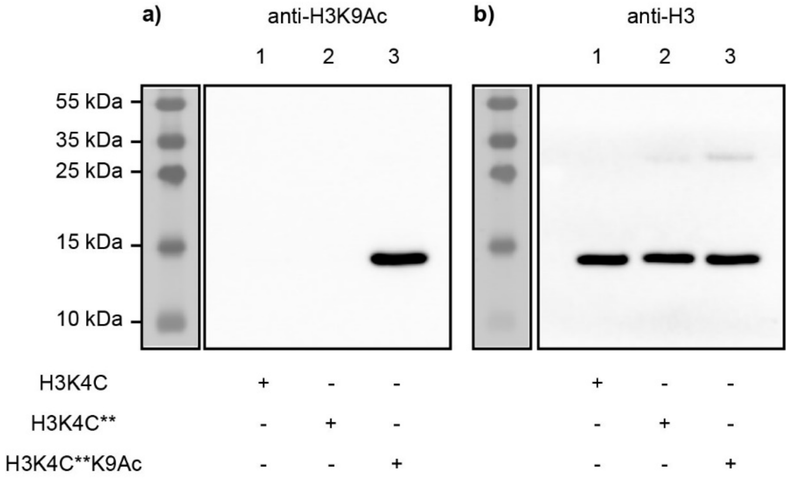

Figure S77. Western blot analysis to confirm K9 acetylation in the H3K4C**K9Ac histone. 300 ng each of H3K4C (lane1), H3K4C** (lane 2) and H3K4C**K9Ac (lane 3) was loaded into the wells of a 15% SDS-PAGE gel. After electrophoresis, proteins were transferred to a nitrocellulose membrane, which was probed with **a)** an anti-acetyl-histone H3 (K9) antibody, and **b)** an anti-histone H3 antibody following acidic stripping. The protein ladder was visualized by colorimetric detection and the protein bands were visualized by chemiluminescence.

H3R52C**K56Ac

Sample preparation and SDS-PAGE

Lyophilized H3R52C, H3R52C** and H3R52C**K56Ac proteins were resuspended in milli-Q water and quantified using the Bradford assay. Two SDS-PAGE gels were prepared, one for Coomassie staining and another for Western Blot. Aliquots of the three proteins (400 ng/µL for Coomassie staining and Western Blot) were diluted in Tris-HCl (20 mM, pH 7.5) and 5 µL of each were mixed with 2x Laemmli sample buffer. *β*-mercaptoethanol (0.5 µL) was added as the reducing agent and samples were boiled at 96 ºC for 4 min. Samples were loaded into a 15% SDS-PAGE gel (2 µg per well), together with the molecular weight marker (PageRuler^{TM} Plus Prestained Protein Ladder, 10 to 250 kDa, ThermoFisher).

Coomassie staining

After running in Tris-Glycine-SDS buffer, the first gel was fixed for 30 min with 40% of methanol in milli-Q water and then stained with InstantBlue Coomassie Stain (Abcam) for 1 h. Excess staining was removed with milli-Q water and the gel was imaged with Amersham Imager 680 RGB (GE Healthcare) (Figure S78).

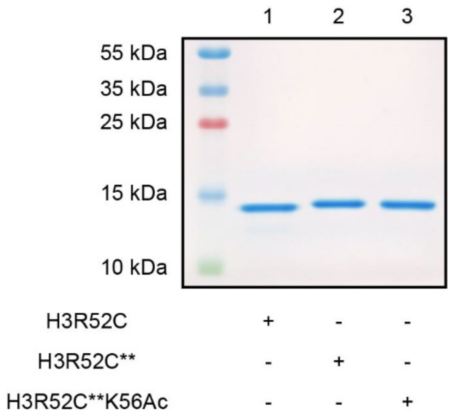

Figure S78. Coomassie staining of the 15% SDS-PAGE gel loaded with 2 µg each of H3R52C (lane 1), H3R52C** (lane 2) and H3R52C**K56Ac (lane 3) proteins. The protein ladder and the protein bands were visualized by colorimetric detection.

Membrane transfer and probing with the H3K56Ac antibody

After running the second gel in Tris-Glycine-SDS buffer, proteins were transferred to a membrane (Amersham Protran Supported Nitrocellulose 0.2 μm) in wet conditions with 20% methanol under constant current for 2 h at 200 mV. The membrane was then washed (1 x 5 min) in TBS (20 mM Tris HCl, 150 mM NaCl, pH 7.5) and then blocked with 5% non-fat dry milk in TBST (TBS with 0.05% tween-20) for 1 h at room temperature. The membrane was then washed with TBST (3 x 5 min) and incubated overnight at 4 ºC with primary antibody (1:1000, anti-H3K56Ac, rabbit monoclonal, cat. #4243S, Cell Signaling Technologies) diluted in 5% BSA in TBST. After washing with TBST (5 x 5 min), the membrane was then incubated at room temperature for 1 h with secondary antibody (1:3000, goat HRP-conjugated anti-rabbit IgG, Invitrogen) diluted in 5% non-fat dry milk in TBST. After washing with TBST (5 x 5 min) and TBS (1 x 5 min), the membrane was developed using the Clarity Western ECL Substrate (Bio-Rad) and imaged with Amersham Imager 680 RGB (GE Healthcare). A clear band with a molecular weight of approximately 15 kDa corresponding to the acetylated H3 histone at position K56 was observed in the lane loaded with H3R52C**K56Ac, while no bands were visible in the control lanes containing H3R52C and H3R52C** proteins (Figure S79a).

Membrane stripping and probing with the H3 antibody

The membrane was re-probed with the H3 antibody, which served as a control to confirm that absence of the K56Ac acetylation signal in lanes 1 and 2 is not related to any technical issue (such as variations in protein loading between wells, uneven membrane transfer or antibody probing). Therefore, stripping (2 x 5 min) was performed using an acidic buffer (200 mM glycine, 0.1 % (w/v) SDS, 1% (v/v) Tween-20, pH 2.2). After washing with PBS (2 x 10 min) and TBS (2 x 5 min), the membrane was blocked with 5% non-fat dry milk in TBST for 1 h at room temperature. The membrane was then washed with TBST (3 x 5 min) and incubated overnight at 4 ºC with primary antibody (1:10000, anti-H3, mouse monoclonal, 1B1B2, cat. #14269S, Cell Signaling Technologies) diluted in 5% BSA in TBST (0.05% tween-20). After washing with TBST (6 x 5 min, 0.1% tween-20), the membrane was incubated at room temperature for 1 h with secondary antibody (1:10000, goat HRP-conjugated anti-mouse IgG H+L, Invitrogen) diluted in 5% non-fat dry milk in TBST (0.1% tween-20). After washing with TBST (7 x 5 min, 0.1% tween-20) and TBS (1 x 5 min), the membrane was developed using the Clarity Western ECL Substrate (Bio-Rad) and imaged with Amersham Imager 680 RGB (GE Healthcare). Clear bands with equivalent densitometry values and a molecular weight of roughly 15 kDa corresponding to histone H3 were observed in the lanes loaded with H3R52C, H3R52C** and H3R52C**K56Ac (Figure S79b).

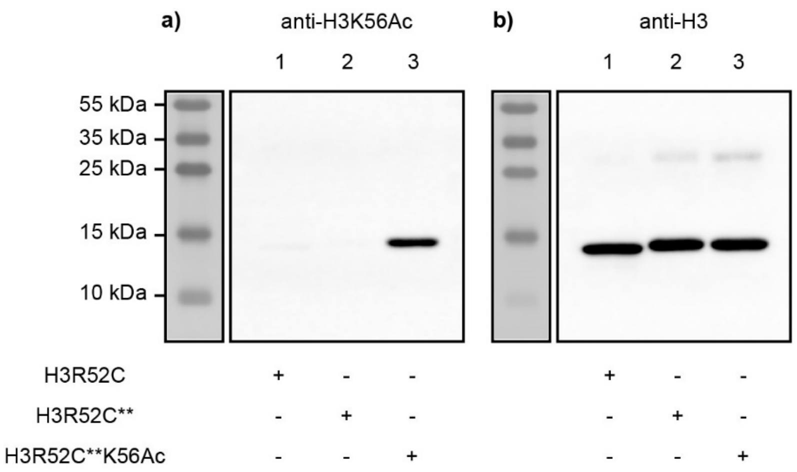

Figure S79. Western blot analysis to confirm K56 acetylation in the H3R52C**K56Ac histone. 2 μg each of H3R52C (lane 1), H3R52C** (lane 2) and H3R52C**K56Ac (lane 3) was loaded into the wells of a 15% SDS-PAGE gel. After electrophoresis, proteins were transferred to a nitrocellulose membrane, which was probed with **a)** an anti-acetyl-histone H3 (K56) antibody, and **b)** an anti-histone H3 antibody following acidic stripping. The protein ladder was visualized by colorimetric detection and the protein bands were visualized by chemiluminescence.

H3K4C**K9Ac and H3R52C**K56Ac

Sample preparation and SDS-PAGE

Lyophilized H3K4C**K9Ac and H3R52C**K56Ac proteins were resuspended in millI-Q water and quantified using the Bradford assay. One SDS-PAGE gel was prepared for Western Blot. Aliquots of the two proteins (60 ng/μL) were diluted in Tris-HCl (20 mM, pH 7.5) and 5 μL of each were mixed with 2x Laemmli sample buffer. *β*-mercaptoethanol (0.5 μL) was added as the reducing agent and samples were boiled at 96 ºC for 4 min. Samples were loaded into a 15% SDS-PAGE gel (300 ng per well), together with the molecular weight marker (PageRuler^{TM} Plus Prestained Protein Ladder, 10 to 250 kDa, ThermoFisher).

Membrane transfer and probing with the H3K9Ac antibody

After running the gel in Tris-Glycine-SDS buffer, proteins were transferred to a membrane (Amersham Protran Supported Nitrocellulose 0.2 μm) in wet conditions with 20% methanol under constant current for 2 h at 200 mV. The membrane was then washed (1 x 5 min)

in TBS (20 mM Tris HCl, 150 mM NaCl, pH 7.5) and then blocked with 5% non-fat dry milk in TBST (TBS with 0.05% tween-20) for 1 h at room temperature. The membrane was then washed with TBST (3 x 5 min) and incubated overnight at 4 ºC with primary antibody (1:6000, anti-H3K9Ac, rabbit monoclonal, C5B11, cat. # 9649S, Cell Signaling Technologies) diluted in 5% BSA in TBST. After washing with TBST (5 x 5 min), the membrane was then incubated at room temperature for 1 h with secondary antibody (1:10000, goat HRP-conjugated anti-rabbit IgG, Invitrogen) diluted in 5% non-fat dry milk in TBST. After washing with TBST (5 x 5 min) and TBS (1 x 5 min), the membrane was developed using the Clarity Western ECL Substrate (Bio-Rad) and imaged with Amersham Imager 680 RGB (GE Healthcare). A clear band with a molecular weight of approximately 15 kDa corresponding to the acetylated H3 histone at position K9 was observed only in the lane loaded with H3K4C**K9Ac, while no band was visible in the lane containing the H3R52C**K56Ac protein (Figure S80a).

Membrane stripping and probing with the H3 antibody

The membrane was re-probed with the H3 antibody, which served as a control to confirm that absence of the K9Ac acetylation signal in lane 2 is not related to any technical issue (such as variations in protein loading between wells, uneven membrane transfer or antibody probing). Therefore, stripping (2 x 5 min) of the membrane was performed using an acidic buffer (200 mM glycine, 0.1 % (w/v) SDS, 1% (v/v) Tween-20, pH 2.2). After washing with PBS (2 x 10 min) and TBS (2 x 5 min), the membrane was blocked with 5% non-fat dry milk in TBST for 1 h at room temperature. The membrane was then washed with TBST (3 x 5 min) and incubated overnight at 4 ºC with primary antibody (1:1000, anti-H3, mouse monoclonal, 1B1B2, cat. #14269S, Cell Signaling Technologies) diluted in 5% BSA in TBST (0.05% tween-20). After washing with TBST (6 x 5 min, 0.1% tween-20), the membrane was incubated at room temperature for 1 h with secondary antibody (1:5000, goat HRP-conjugated anti-mouse IgG H+L, Invitrogen) diluted in 5% non-fat dry milk in TBST (0.1% tween-20). After washing with TBST (7 x 5 min, 0.1% tween-20) and TBS (1 x 5 min), the membrane was developed using the Clarity Western ECL Substrate (Bio-Rad) and imaged with Amersham Imager 680 RGB (GE Healthcare). Clear bands with equivalent densitometry values and a molecular weight of roughly 15 kDa corresponding to histone H3 were observed in the lanes loaded with H3K4C**K9Ac and H3R52C**K9Ac (Figure S80b).

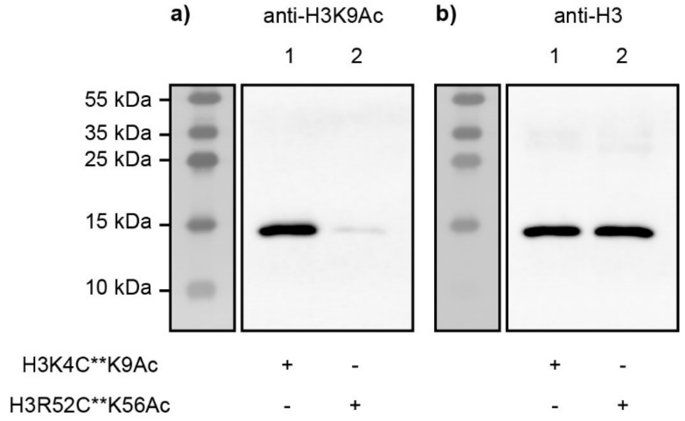

Figure S80. Western blot analysis to confirm specific K9 acetylation in the H3K4C**K9Ac histone. 300 ng each of H3K4C**K9Ac (lane 1) and H3R52C**K56Ac was loaded into the wells of a 15% SDS-PAGE gel. After electrophoresis, proteins were transferred to a nitrocellulose membrane, which was probed with **a)** an anti-acetyl-histone H3 (K9) antibody, and **b)** an anti-histone H3 antibody following acidic stripping. The protein ladder was visualized by colorimetric detection and the protein bands were visualized by chemiluminescence.

H3K4C**K9AcR52C**K56Ac

Sample preparation and SDS-PAGE

Lyophilized H3K4CR52C, H3K4C**R52C** and H3K4C**K9AcR52C**K56Ac proteins were resuspended in milli-Q water and quantified using the Bradford assay. Three SDS-PAGE gels were prepared, one for Coomassie staining and two for Western Blot. Aliquots of the three proteins (60 ng/µL for Western Blot analysis with the anti-H3K9Ac antibody; 400 ng/µL for Western Blot analysis with the anti-H3K56Ac antibody and Coomassie staining) were diluted in Tris-HCl (20 mM, pH 7.5) and 5 µL of each were mixed with 2x Laemmli sample buffer. *β*-mercaptoethanol (0.5 µL) was added as the reducing agent and samples were boiled at 96 ºC for 4 min. Samples were loaded into a 15% SDS-PAGE gel (300 ng per well for Western Blot with the anti-H3K9Ac antibody; 2 µg per well for Coomassie staining and Western Blot with the anti-H3K56Ac antibody), together with the molecular weight marker (PageRuler^{TM} Plus Prestained Protein Ladder, 10 to 250 kDa, ThermoFisher).

Coomassie staining

After running in Tris-Glycine-SDS buffer, the first gel was fixed for 30 min with 40% of methanol in milli-Q water and then stained with InstantBlue Coomassie Stain (Abcam) for 1 h. Excess staining was removed with milli-Q water and the gel was imaged with Amersham Imager 680 RGB (GE Healthcare) (Figure S81).

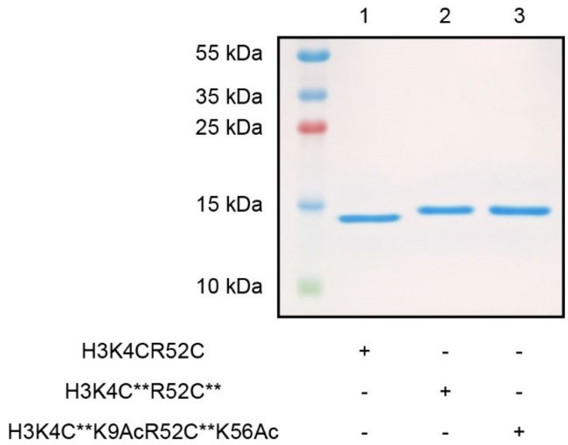

Figure S81. Coomassie staining of the 15% SDS-PAGE gel loaded with 2 µg each of H3K4CR52C (lane 1), H3K4C**R52C** (lane 2) and H3K4C**K9AcR52C**K56Ac (lane 3) proteins. The protein ladder and the protein bands were visualized by colorimetric detection.

Membrane transfer and probing with the H3K9Ac antibody

After running the second gel in Tris-Glycine-SDS buffer, proteins were transferred to a membrane (Amersham Protran Supported Nitrocellulose 0.2 µm) in wet conditions with 20% methanol under constant current for 2 h at 200 mV. The membrane was then washed (1 x 5 min) in TBS (20 mM Tris HCl, 150 mM NaCl, pH 7.5) and then blocked with 5% non-fat dry milk in TBST (TBS with 0.05% tween-20) for 1 h at room temperature. The membrane was then washed with TBST (3 x 5 min) and incubated overnight at 4 ºC with primary antibody (1:6000, anti-H3K9Ac, rabbit monoclonal, C5B11, cat. # 9649S, Cell Signaling Technologies) diluted in 5% BSA in TBST. After washing with TBST (5 x 5 min), the membrane was then incubated at room temperature for 1 h with secondary antibody (1:10000, goat HRP-conjugated anti-rabbit IgG, Invitrogen) diluted in 5% non-fat dry milk in TBST. After washing with TBST (5 x 5 min) and TBS (1 x 5 min), the membrane was developed using the Clarity Western ECL Substrate (Bio-Rad) and imaged with Amersham Imager 680 RGB (GE Healthcare). A clear band with a molecular weight of approximately 15 kDa corresponding to the acetylated H3 histone at position K9 was observed in the lane loaded with H3K4C**K9AcR52C**K56Ac, while no bands were visible in the control lanes containing the H3K4CR52C and H3K4C**R52C** proteins (Figure S82a).

Membrane stripping and probing with the H3 antibody

The membrane was re-probed with the H3 antibody, which served as a control to confirm that absence of the K9Ac acetylation signal in lanes 1 and 2 is not related to any technical issue (such as variations in protein loading between wells, uneven membrane transfer or antibody probing). Therefore, stripping (2 x 5 min) of the membrane was performed using an acidic buffer (200 mM glycine, 0.1 % (w/v) SDS, 1% (v/v) Tween-20, pH 2.2). After washing with PBS (2 x 10 min) and TBS (2 x 5 min), the membrane was blocked with 5% non-fat dry milk in TBST for 1 h at room temperature. The membrane was then washed with TBST (3 x 5 min) and incubated overnight at 4 ºC with primary antibody (1:1000, anti-H3, mouse monoclonal, 1B1B2, cat. #14269S, Cell Signaling Technologies) diluted in 5% BSA in TBST (0.05% tween-20). After washing with TBST (6 x 5 min, 0.1% tween-20), the membrane was incubated at room temperature for 1 h with secondary antibody (1:5000, goat HRP-conjugated anti-mouse IgG H+L, Invitrogen) diluted in 5% non-fat dry milk in TBST (0.1% tween-20). After washing with TBST (7 x 5 min, 0.1% tween-20) and TBS (1 x 5 min), the membrane was developed using the Clarity Western ECL Substrate (Bio-Rad) and imaged with the Amersham Imager 680 RGB (GE Healthcare). Clear bands with equivalent densitometry values and a molecular weight of roughly 15 kDa corresponding to histone H3 were observed in the lanes loaded with H3K4CR52C, H3K4C**R52C** and H3K4C**K9AcR52C**K56Ac (Figure S82b).

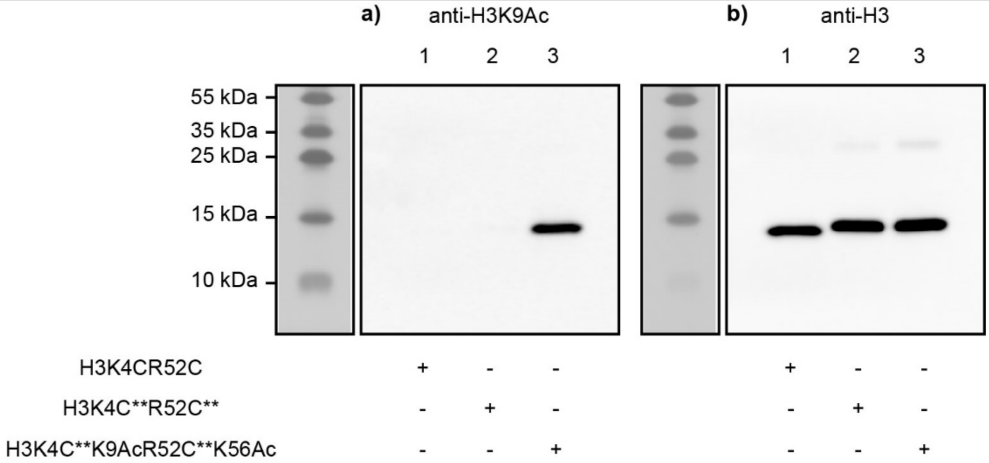

Figure S82. Western blot analysis to confirm K9 acetylation in the H3K4C**K9AcR52C**K56Ac histone. 300 ng each of H3K4CR52C (lane 1), H3K4C**R52C** (lane 2) and H3K4C**K9AcR52C**K56Ac (lane 3) was loaded into the wells of a 15% SDS-PAGE gel. After electrophoresis, proteins were transferred to a nitrocellulose membrane, which was probed with **a)** an anti-acetyl-histone H3 (K9) antibody, and **b)** an anti-histone H3 antibody following acidic stripping. The protein ladder was visualized by colorimetric detection and the protein bands were visualized by chemiluminescence.

Membrane transfer and probing with the H3K56Ac antibody

After running the third gel in Tris-Glycine-SDS buffer, proteins were transferred to a membrane (Amersham Protran Supported Nitrocellulose 0.2 µm) in wet conditions with 20% methanol under constant current for 2 h at 200 mV. The membrane was then washed (1 x 5 min) in TBS (20 mM Tris HCl, 150 mM NaCl, pH 7.5) and then blocked with 5% non-fat dry milk in TBST (TBS with 0.05% tween-20) for 1 h at room temperature. The membrane was then washed with TBST (3 x 5 min) and incubated overnight at 4 ºC with primary antibody (1:1000, anti-H3K56Ac, rabbit monoclonal, cat. #4243S, Cell Signaling Technologies) diluted in 5% BSA in TBST. After washing with TBST (5 x 5 min), the membrane was then incubated at room temperature for 1 h with secondary antibody (1:3000, goat HRP-conjugated anti-rabbit IgG, Invitrogen) diluted in 5% non-fat dry milk in TBST. After washing with TBST (5 x 5 min) and TBS (1 x 5 min), the membrane was developed using the Clarity Western ECL Substrate (Bio-Rad) and imaged with Amersham Imager 680 RGB (GE Healthcare). A clear band with a molecular weight of approximately 15 kDa corresponding to the acetylated H3 histone at position K56 was observed in the lane loaded with H3K4C**K9AcR52C**K56Ac, while no bands were visible in the control lanes containing H3K4CR52C and H3K4C**R52C** proteins (Figure S83a).

Membrane stripping and probing with the H3 antibody

The membrane was re-probed with the H3 antibody, which served as a control to confirm that absence of the K56Ac acetylation signal in lanes 1 and 2 is not related to any technical issue (such as variations in protein loading between wells, uneven membrane transfer or antibody probing). Therefore, stripping (2 x 5 min) was performed using an acidic buffer (200 mM glycine, 0.1 % (w/v) SDS, 1% (v/v) Tween-20, pH 2.2). After washing with PBS (2 x 10 min) and TBS (2 x 5 min), the membrane was blocked with 5% non-fat dry milk in TBST for 1 h at room temperature. The membrane was then washed with TBST (3 x 5 min) and incubated overnight at 4 ºC with primary antibody (1:10000, anti-H3, mouse monoclonal, 1B1B2, cat. #14269S, Cell Signaling Technologies) diluted in 5% BSA in TBST (0.05% tween-20). After washing with TBST (6 x 5 min, 0.1% tween-20), the membrane was incubated at room temperature for 1 h with secondary antibody (1:10000, goat HRP-conjugated anti-mouse IgG H+L, Invitrogen) diluted in 5% non-fat dry milk in TBST (0.1% tween-20). After washing with TBST (7 x 5 min, 0.1% tween-20) and TBS (1 x 5 min), the membrane was developed using the Clarity Western ECL Substrate (Bio-Rad) and imaged with Amersham Imager 680 RGB (GE Healthcare). Clear bands with equivalent densitometry values and a molecular weight of roughly 15 kDa corresponding to histone H3 were observed in the lanes loaded with H3K4CR52C, H3K4C**R52C** and H3K4C**K9AcR52C**K56Ac (Figure S83b).

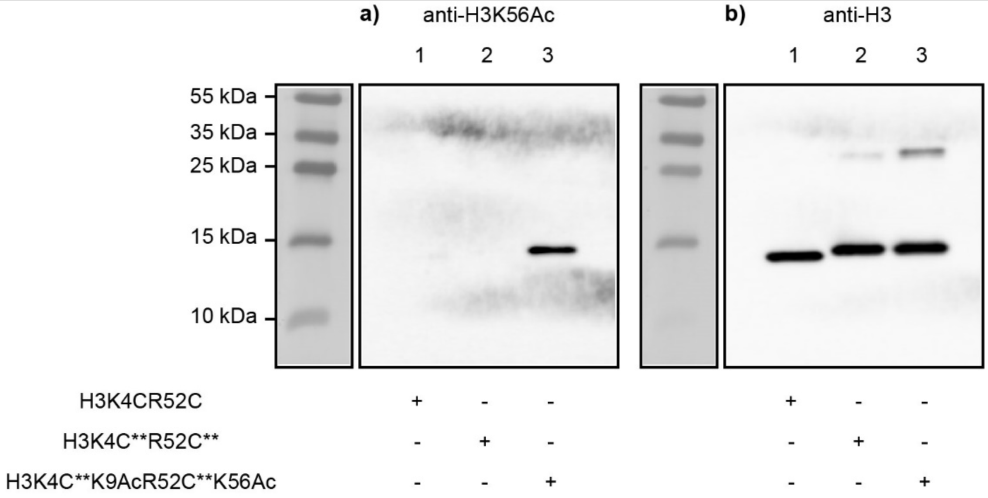

Figure S83. Western blot analysis to confirm K56 acetylation in the H3K4C**K9AcR52C**K56Ac histone. 2 µg each of H3K4CR52C (lane 1), H3K4C**R52C** (lane 2) and H3K4C**K9AcR52C**K56Ac (lane 3) was loaded into the wells of a 15% SDS-PAGE gel. After electrophoresis, proteins were transferred to a nitrocellulose membrane, which was probed with **a)** an anti-acetyl-histone H3 (K56) antibody, and **b)** an anti-histone H3 antibody following acidic stripping. The protein ladder was visualized by colorimetric detection and the protein bands were visualized by chemiluminescence.

9. Development of an ELISA protocol for the detection and quantitation of K9 acetylation

In addition to Western blot analysis, an enzyme-linked immunosorbent assay (ELISA) was also performed to detect K9 acetylation in the H3K4C**K9Ac protein. This two-day assay was developed in-house using the same antibodies as those previously described for Western blot and ultimately served to quantitate the level of K9 acetylation following incubation with Sirt3.

Lyophilized H3K4C, H3K4C** and H3K4C**K9Ac proteins were resuspended in milli-Q water and quantified using the Bradford assay. Two-fold serial dilutions of each protein were made from 8 μg/mL to 0.5 μg/mL in carbonate buffer (100 mM, pH 9.6). Nunc MaxiSorp 96 well ELISA plates were coated with duplicate serial dilutions of H3K4C, H3K4C** and H3K4C**K9Ac and incubated overnight at 4 ºC. After washing the plates (3 x 5 min) with PBST (0.05% tween-20), blocking buffer (200 μL per well) was added to each well (3% and 5% milk in PBST for anti-H3 and anti-H3K9Ac probing, respectively). After incubating for 2 h at room temperature, the plate was washed (1 x 5 min). Primary antibody dilutions (100 μL per well) of anti-H3 (1:2000 in 3% BSA in PBST) and anti-H3K9Ac (1:2000 in 5% BSA in PBST) were then added. Following incubation at room temperature for 2 h, the plates were washed with PBST (3 x 5 min). Secondary goat anti-mouse and anti-rabbit HRP-conjugated antibodies (100 μL per well, Invitrogen) were added at 1:2000 and 1:4000 dilutions in 3% and 5% milk in TBST, respectively. After incubating for 1 h at room temperature, the plate was washed (5 x 5 min) with PBST. TMB substrate (90 μL per well, Invitrogen) was added and the plate was left to incubate at room temperature. After sufficient colour development, the colorimetric reaction was stopped with a 1M sulphuric acid solution (50 μL per well). Absorbance at 450 nm was then recorded on a Tecan Infinite M200 plate reader.

ELISA results were analysed using GraphPad Prism software (version 6.01) by fitting the data of each combination of target protein (H3K4C, H3K4C** and H3K4C**K9Ac) and probe (anti-H3 and anti-H3K9Ac) to a four-parameter logistic (4PL) regression of a standard curve, with the logarithm of the concentration plotted on the x-axis and the absorbance plotted on the y-axis. The generated standard curves are shown in Figure S84. As observed, the developed ELISA protocol can specifically detect H3K9 acetylation, since the colorimetric signal is only significantly observed for H3K4C**K9Ac and not for H3K4C or H3K4C** proteins. A basal signal of K9 acetylation can be observed in the wells containing a very high amount of H3K4C and H3K4C** proteins, but its value is of the same order of magnitude as the one registered for the lowest amounts of H3K4C**K9Ac. Moreover, a strong H3 colorimetric signal can be observed in all three proteins, indicating that the absence of acetyl-lysine detection at position 9 in the H3K4C and H3K4C** proteins is not related to assay or technical issues.

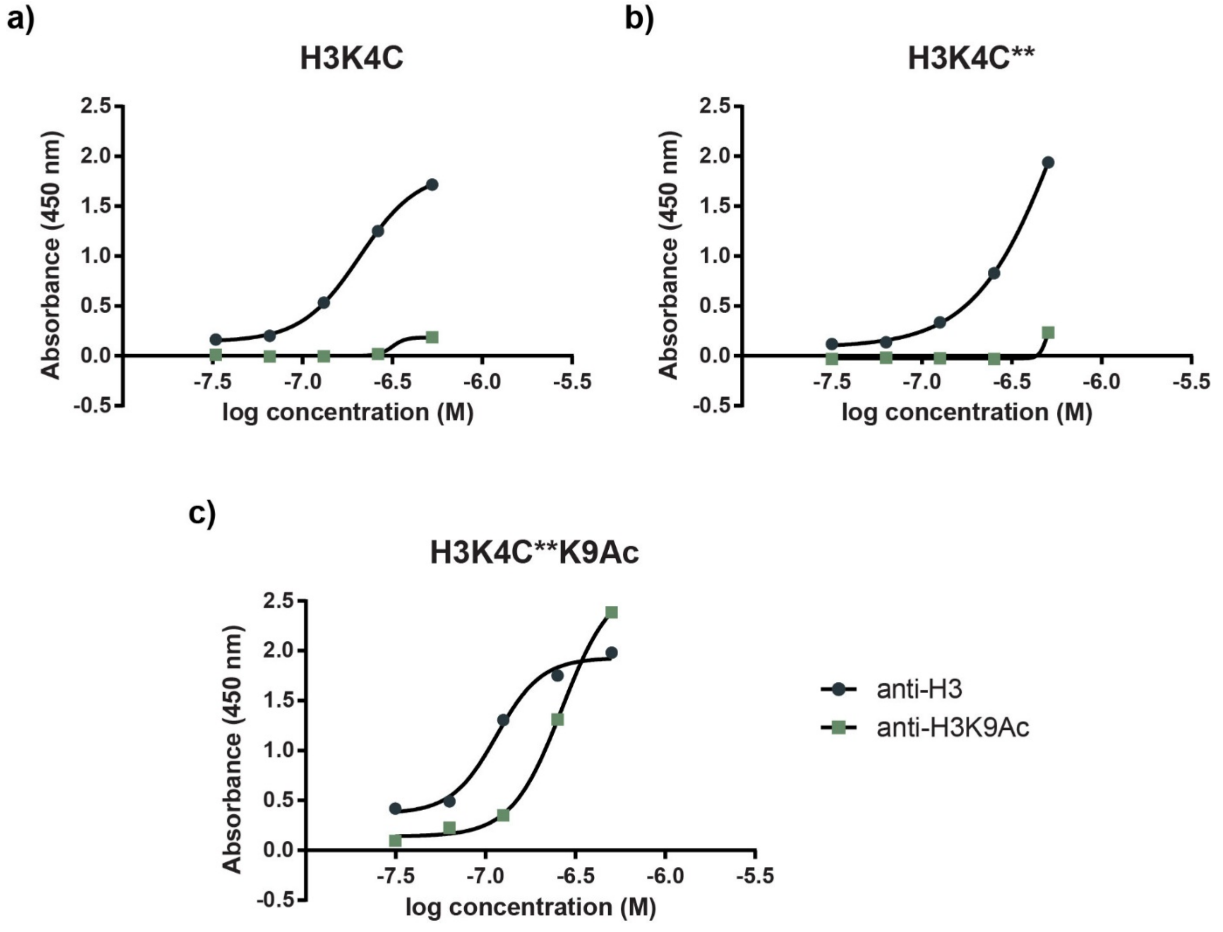

Figure S84. Development of an ELISA protocol for the detection and quantitation of K9 acetylation in histone H3. Standard curves obtained following probing of the **a)** H3K4C, **b)** H3K4C** and **c)** H3K4C**K9Ac proteins with anti-H3 and anti-H3K9Ac antibodies.

10. Deacetylation assays of the H3K4C**K9Ac and H3R52C**K56Ac proteins with Sirt3

To establish that H3K4C**K9Ac and H3R52C**K56Ac proteins can be recognized by histone-modifying enzymes, these chemically acetylated histones were used as substrates in NAD^+-dependent deacetylation assays with Sirt3.

H3K4C**K9Ac

Lyophilized H3K4C**K9Ac protein was resuspended in milli-Q water and quantified using the Bradford assay. The control and reaction samples were then prepared with a final volume of 45 µL. For both samples, the 10-times concentrated deacetylation buffer (250 mM Tris, 1.37 M NaCl, 27 mM KCl, 10 mM $MgCl_2$, pH 8.0) was diluted 1:10 in milli-Q water. DTT (90 mM in milli-Q water) and NAD^+ (50 mM in 50 mM Tris-HCl, pH 8.0, 137 mM NaCl, 2.7 mM KCl, 1 mM $MgCl_2$ from Enzo Life Sciences) were added to a final concentration of 1 mM and 3.5 mM, respectively. An aliquot of H3K4C**K9Ac was added to the mixture at a final concentration of 60 ng/µL. For the reaction sample, a 0.5 µL aliquot containing 20 units of recombinant Sirt3 (1 µg/µL in 25 mM Tris pH 7.5, 100 mM NaCl, 5 mM DTT, 10% glycerol from Enzo Life Sciences) was also added. The control and reaction samples were then incubated at 37 ºC and 400 rpm for 2 h. Samples were spun down and placed on ice. K9 deacetylation was subsequently evaluated by Western blot and ELISA.

<u>Western blot analysis</u>

5 µL each of control and reaction samples were mixed with 2x Laemmli sample buffer. *β*-mercaptoethanol (0.5 µL) was added as the reducing agent and samples were boiled at 96 ºC for 4 min. Samples were loaded into a 15% SDS-PAGE gel (300 ng per well), together with the molecular weight marker (PageRuler^{TM} Plus Prestained Protein Ladder, 10 to 250 kDa, ThermoFisher). After running the gel in Tris-Glycine-SDS buffer, proteins were transferred to a membrane (Amersham Protran Supported Nitrocellulose 0.2 µm) in wet conditions with 20% methanol under constant current for 2 h at 200 mV. The membrane was then washed (1 x 5 min) in TBS (20 mM Tris HCl, 150 mM NaCl, pH 7.5) and then blocked with 5% non-fat dry milk in TBST (TBS with 0.05% tween-20) for 1 h at room temperature. The membrane was then washed with TBST (3 x 5 min) and incubated overnight at 4 ºC with primary antibody (1:6000, anti-H3K9Ac, rabbit monoclonal, C5B11, cat. #9649S, Cell Signaling Technologies) diluted in 5% BSA in TBST. After washing with TBST (5 x 5 min), the membrane was then incubated at room temperature for 1 h with secondary antibody (1:10000, goat HRP-conjugated anti-rabbit IgG, Invitrogen) diluted in 5% non-fat dry milk in TBST. After washing with TBST (5 x 5 min) and TBS (1 x 5 min), the membrane was developed using the Clarity Western ECL Substrate (Bio-Rad) and imaged with Amersham Imager 680 RGB (GE Healthcare). As shown in Figure S85a, a clear band with a molecular weight of approximately 15 kDa corresponding to the acetylated H3 histone at position K9 was observed in the lane loaded with control sample. However, no band was visible in the lane loaded with the reaction sample, indicating that deacetylation was complete following incubation with Sirt3 for 2 h at 37 ºC. Thus, the chemically acetylated H3K4C**K9Ac protein is recognized by Sirt3 and the presence of the SPAAC product at the cysteine is not detrimental to this process.

The membrane was then re-probed with the H3 antibody, which served as a control to confirm that absence of the K9 acetylation signal in the reaction sample is not related to any technical issue (such as variations in protein loading between wells, uneven membrane transfer or antibody probing) and due only to the deacetylase activity of Sirt3. Therefore, stripping (2 x 5 min) of the membrane was performed using an acidic buffer (200 mM glycine, 0.1 % (w/v) SDS, 1% (v/v) Tween-20, pH 2.2). After washing with PBS (2 x 10 min) and TBS (2 x 5 min), the membrane was blocked with 5% non-fat dry milk in TBST for 1 h at room temperature. The membrane was then washed with TBST (3 x 5 min) and incubated overnight at 4 ºC with primary antibody (1:1000, anti-H3, mouse monoclonal, 1B1B2, cat. # 14269S, Cell Signaling Technologies) diluted in 5% BSA in TBST (0.05% tween-20). After washing with TBST (6 x 5 min, 0.1% tween-20), the membrane was incubated at room temperature for 1 h with secondary antibody (1:5000, goat HRP-conjugated anti-mouse IgG H+L, Invitrogen) diluted in 5% non-fat dry milk in TBST (0.1% tween-20). After washing with TBST (7 x 5 min, 0.1% tween-20) and TBS (1 x 5 min), the membrane was developed using the Clarity Western ECL Substrate (Bio-Rad) and imaged with Amersham Imager 680 RGB (GE Healthcare). Clear bands with equivalent densitometry values and a molecular weight of roughly 15 kDa corresponding to histone H3 were observed in the lanes loaded with the control and reaction samples (Figure S85b).

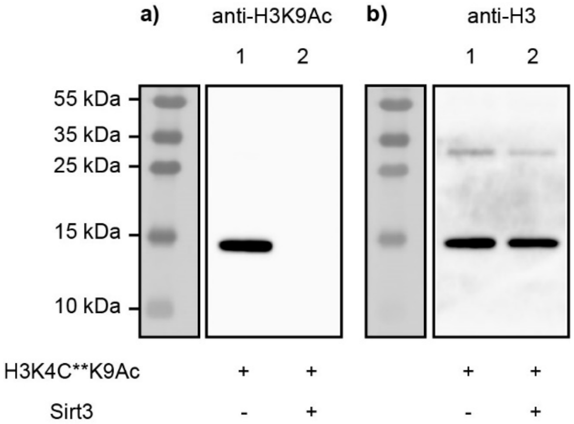

Figure S85. Western blot analysis to evaluate K9 deacetylation following incubation of H3K4C**K9Ac with Sirt3 for 2 h at 37 ºC and 400 rpm. 300 ng each of H3K4C**K9Ac protein from control (lane 1) and reaction (lane 2) samples was loaded into the wells of a 15%

SDS-PAGE gel. After electrophoresis, proteins were transferred to a nitrocellulose membrane, which was probed with **a)** an anti-acetyl-histone H3 (K9) antibody, and **b)** an anti-histone H3 antibody following acidic stripping. The protein ladder was visualized by colorimetric detection and the protein bands were visualized by chemiluminescence.

ELISA

An ELISA was also performed to confirm the Western blot results showing K9 deacetylation following incubation with Sirt3. To this end, a standard curve of the H3K4C**K9Ac protein was prepared by making two-fold serial dilutions from 16 to 0.5 µg/mL in carbonate buffer (100 mM, pH 9.6). Aliquots (28 µl) of the same control and reaction samples as those used for the corresponding Western blot analysis were diluted with 182 µL of carbonate buffer (100 mM, pH 9.6) to a final concentration of 8 µg/mL. These diluted control and reaction samples were plated together with the H3K4C**K9Ac serial dilutions.

Nunc MaxiSorp 96 well ELISA plates were coated with duplicate serial dilutions of H3K4C**K9Ac and incubated overnight at 4 ºC. After washing the plates (3 x 5 min) with PBST (0.05% tween-20), blocking buffer (200 µL per well) was added to each well (3% and 5% milk in PBST for anti-H3 and anti-H3K9Ac probing, respectively). After incubating for 2 h at room temperature, the plate was washed (1 x 5 min). Primary antibody dilutions (100 µL per well) of anti-H3 (1:2000 in 3% BSA in PBST) and anti-H3K9Ac (1:2000 in 5% BSA in PBST) were then added. Following incubation at room temperature for 2 h, the plates were washed with PBST (3 x 5 min). Secondary goat anti-mouse and anti-rabbit HRP-conjugated antibodies (100 µL per well, Invitrogen) were added at 1:2000 and 1:4000 dilutions in 3% and 5% milk in TBST, respectively. After incubating for 1 h at room temperature, the plate was washed (5 x 5 min) with PBST. TMB substrate (90 µL per well, Invitrogen) was added and the plate was left to incubate at room temperature. After sufficient colour development, the colorimetric reaction was stopped with a 1M sulphuric acid solution (50 µL per well). Absorbance at 450 nm was then recorded on a Tecan Infinite M200 plate reader.

ELISA results were analysed using GraphPad Prism software (version 6.01) by fitting the data of each combination of target protein (H3K4C**K9Ac) and probing (anti-H3 and anti-H3K9Ac) to a four-parameter logistic (4PL) regression of a standard curve, with the logarithm of the concentration plotted on the x-axis and the absorbance plotted on the y-axis. The generated standard curves are shown in Figure S86a. Subsequently, the level of K9 acetylation in control and reaction samples was interpolated from the standard curve of H3K4C**K9Ac probed with anti-H3K9Ac based on the absorbance values shown in Figure S86b. As observed in Figure S86b and S86c, incubation with Sirt3 for 2 h at 37 ºC resulted in the complete deacetylation of the H3K4C**K9Ac protein. Moreover, the interpolated value of K9 acetylation obtained for the control sample is very similar to the concentration of H3K4C**K9Ac protein loaded into the ELISA plate wells. To confirm that the absence of K9 acetylation signal in the reaction sample is not related to any technical issue (such as variations in protein loading or antibody probing between wells) and due only to the deacetylase activity of Sirt3, the H3 concentration in control and reaction samples was interpolated from the standard curve of H3K4C**K9Ac probed with anti-H3. As observed in Figure S86d, while the interpolated H3 concentration values are less than half of those that we would expect, these are quite similar between control and reaction samples. By normalizing the individual values to the average H3 signal of the control, we observe that both samples generate similar colorimetric signals following H3 probing, which indicates that similar protein quantities were loaded into the wells. Therefore, the remarkable differences in K9 acetylation levels between control and reaction samples following Sirt3 incubation can only be explained by enzymatic deacetylation.

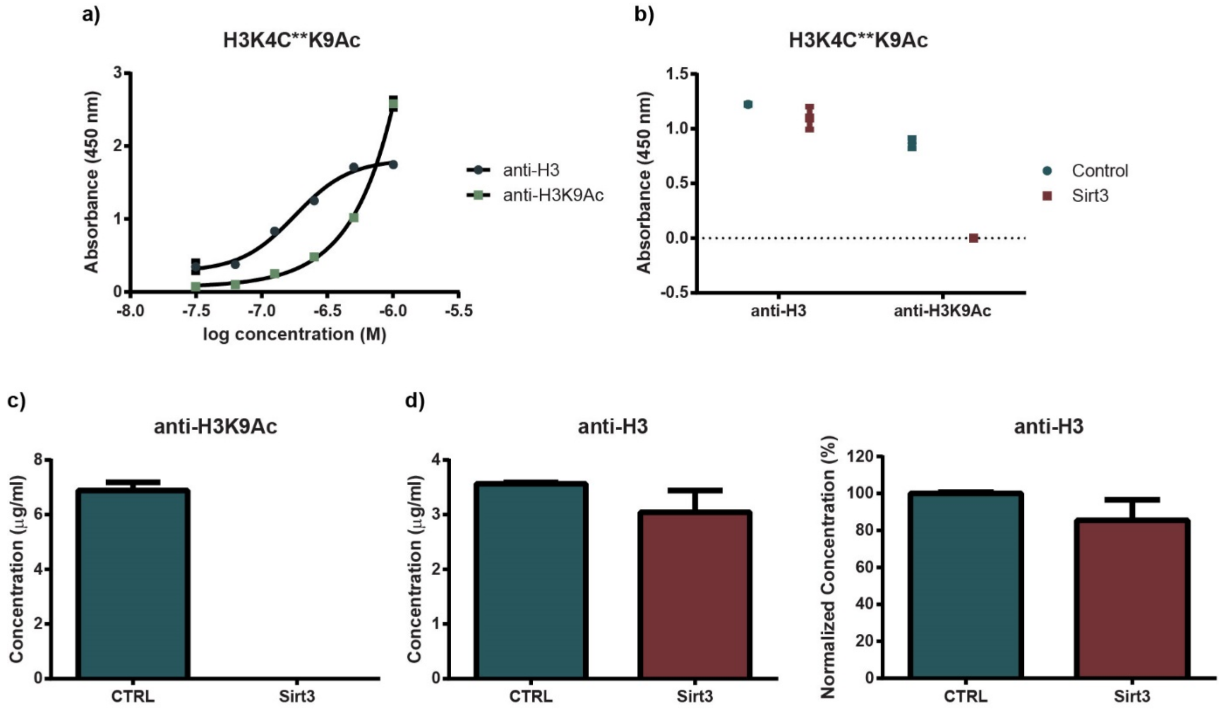

Figure S86. ELISA experiments to evaluate K9 deacetylation following incubation of H3K4C**K9Ac with Sirt3 for 2 h at 37 ºC and 400 rpm. **a)** Standard curves obtained for the H3K4C**K9Ac protein following probing with anti-H3 and anti-H3K9Ac antibodies, **b)**

absorbance values obtained following anti-H3 and anti-H3K9Ac probing in control and Sirt3-treated samples, and **c)** interpolated H3 concentration values (in µg/mL) in control and Sirt3-treated samples and respective normalization (in percentage) to the average H3 control signal.

H3R52C**K56Ac

Lyophilized H3R52C**K56Ac protein was resuspended in milli-Q water and quantified using the Bradford assay. The control and reaction samples were then prepared with a final volume of 20 µL. For both samples, the 10-times concentrated deacetylation buffer (250 mM Tris, 1.37 M NaCl, 27 mM KCl, 10 mM $MgCl_2$, pH 8.0) was diluted 1:10 in milli-Q water. DTT (40 mM in milli-Q water) and NAD^+ (50 mM in 50 mM Tris-HCl, pH 8.0, 137 mM NaCl, 2.7 mM KCl, 1 mM $MgCl_2$ from Enzo Life Sciences) were added to a final concentration of 1 mM and 3.5 mM, respectively. An aliquot of H3R52C**K56Ac was added to the mixture at a final concentration of 200 ng/µL. For the reaction sample, a 0.5 µL aliquot containing 20 units of recombinant Sirt3 (1 µg/µL in 25 mM Tris, pH 7.5, 100 mM NaCl, 5 mM DTT, 10% glycerol from Enzo Life Sciences) was also added. The control and reaction samples were then incubated at 37 ºC and 400 rpm for 4 h. Samples were spun down and placed on ice. K56 deacetylation was subsequently evaluated by Western blot.

Western blot analysis

10 µL each of control and reaction samples were mixed with 2x Laemmli sample buffer. *β*-mercaptoethanol (0.5 µL) was added as the reducing agent and samples were boiled at 96 ºC for 4 min. Samples were loaded into a 15% SDS-PAGE gel (2 µg of protein per well), together with the molecular weight marker (PageRuler^{TM} Plus Prestained Protein Ladder, 10 to 250 kDa, ThermoFisher). After running the gel in Tris-Glycine-SDS buffer, proteins were transferred to a membrane (Amersham Protran Supported Nitrocellulose 0.2 µm) in wet conditions with 20% methanol under constant current for 2 h at 200 mV. The membrane was then washed (1 x 5 min) in TBS (20 mM Tris HCl, 150 mM NaCl, pH 7.5) and then blocked with 5% non-fat dry milk in TBST (TBS with 0.05% tween-20) for 1 h at room temperature. The membrane was then washed with TBST (3 x 5 min) and incubated overnight at 4 ºC with primary antibody (1:1000, anti-H3K56Ac, rabbit monoclonal, cat. #4243S, Cell Signaling Technologies) diluted in 5% BSA in TBST. After washing with TBST (5 x 5 min), the membrane was then incubated at room temperature for 1 h with secondary antibody (1:3000, goat HRP-conjugated anti-rabbit IgG, Invitrogen) diluted in 5% non-fat dry milk in TBST. After washing with TBST (5 x 5 min) and TBS (1 x 5 min), the membrane was developed using the Clarity Western ECL Substrate (Bio-Rad) and imaged with Amersham Imager 680 RGB (GE Healthcare). A strong and clear band with a molecular weight of approximately 15 kDa corresponding to the acetylated H3 histone at position K56 was observed in the lane loaded with the control sample. While the corresponding band in the lane loaded with the reaction sample was still visible, a very significant reduction in its intensity was also seen (Figure S87a). Therefore, although deacetylation was not complete under these experimental conditions, the results indicate that Sirt3 also recognizes the chemically acetylated H3R52C**K56Ac protein, even in the presence of the SPAAC product at the cysteine.

The membrane was then re-probed with the H3 antibody, which served as a control to confirm that reduction of the K56 acetylation signal in the reaction sample is not related to any technical issue (such as variations in protein loading between wells, uneven membrane transfer or antibody probing) and due only to the deacetylase activity of Sirt3. Therefore, stripping (2 x 5 min) of the membrane was performed using an acidic buffer (200 mM glycine, 0.1 % (w/v) SDS, 1% (v/v) Tween-20, pH 2.2). After washing with PBS (2 x 10 min) and TBS (2 x 5 min), the membrane was blocked with 5% non-fat dry milk in TBST for 1 h at room temperature. The membrane was then washed with TBST (3 x 5 min) and incubated overnight at 4 ºC with primary antibody (1:10000, anti-H3, mouse monoclonal, 1B1B2, cat. #14269S, Cell Signaling Technologies) diluted in 5% BSA in TBST (0.05% tween-20). After washing with TBST (6 x 5 min, 0.1% tween-20), the membrane was incubated at room temperature for 1 h with secondary antibody (1:10000, goat HRP-conjugated anti-mouse IgG H+L, Invitrogen) diluted in 5% non-fat dry milk in TBST (0.1% tween-20). After washing with TBST (7 x 5 min, 0.1% tween-20) and TBS (1 x 5 min), the membrane was developed using the Clarity Western ECL Substrate (Bio-Rad) and imaged with Amersham Imager 680 RGB (GE Healthcare). Clear bands with equivalent densitometry values and a molecular weight of roughly 15 kDa corresponding to histone H3 were observed in the lanes loaded with the control and reaction samples (Figure S87b).

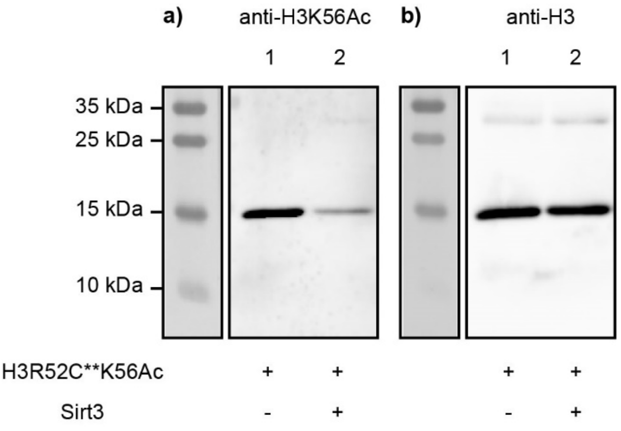

Figure S87. Western blot analysis to evaluate K56 deacetylation following incubation of H3R52C**K56Ac with Sirt3 for 4 h at 37 ºC and 400 rpm. 2 µg of H3R52C**K56Ac protein from control (lane 1) and reaction (lane 2) samples were loaded into the wells of a 15% SDS-PAGE gel. After electrophoresis, proteins were transferred to a nitrocellulose membrane, which was probed with **a)** an anti-acetyl-

histone H3 (K56) antibody, and **b)** an anti-histone H3 antibody following acidic stripping. The protein ladder was visualized by colorimetric detection and the protein bands were visualized by chemiluminescence.

11. NMR spectra

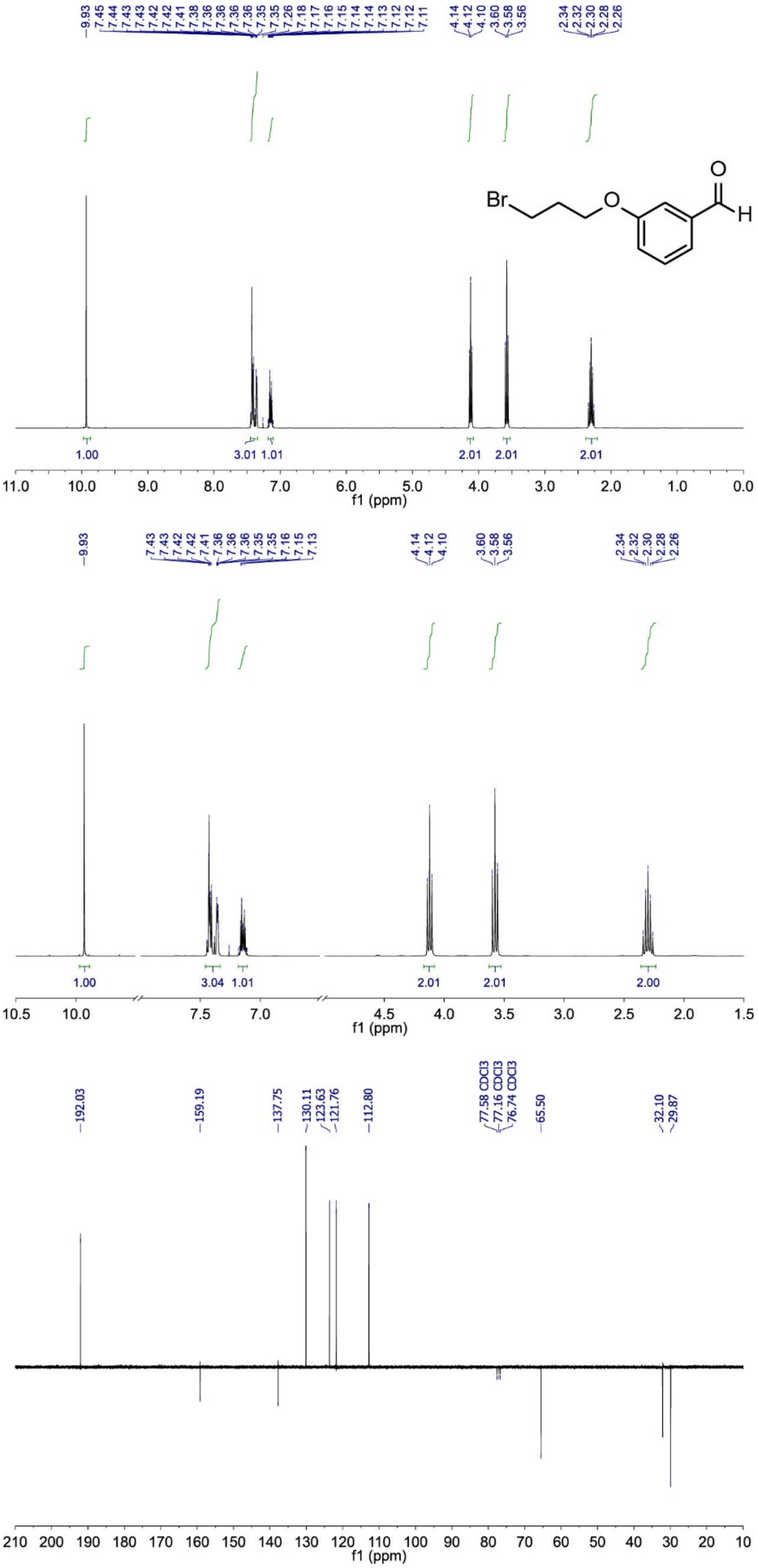

Figure S88. ^{1}H- and ^{13}C-NMR spectrum of compound **1**.

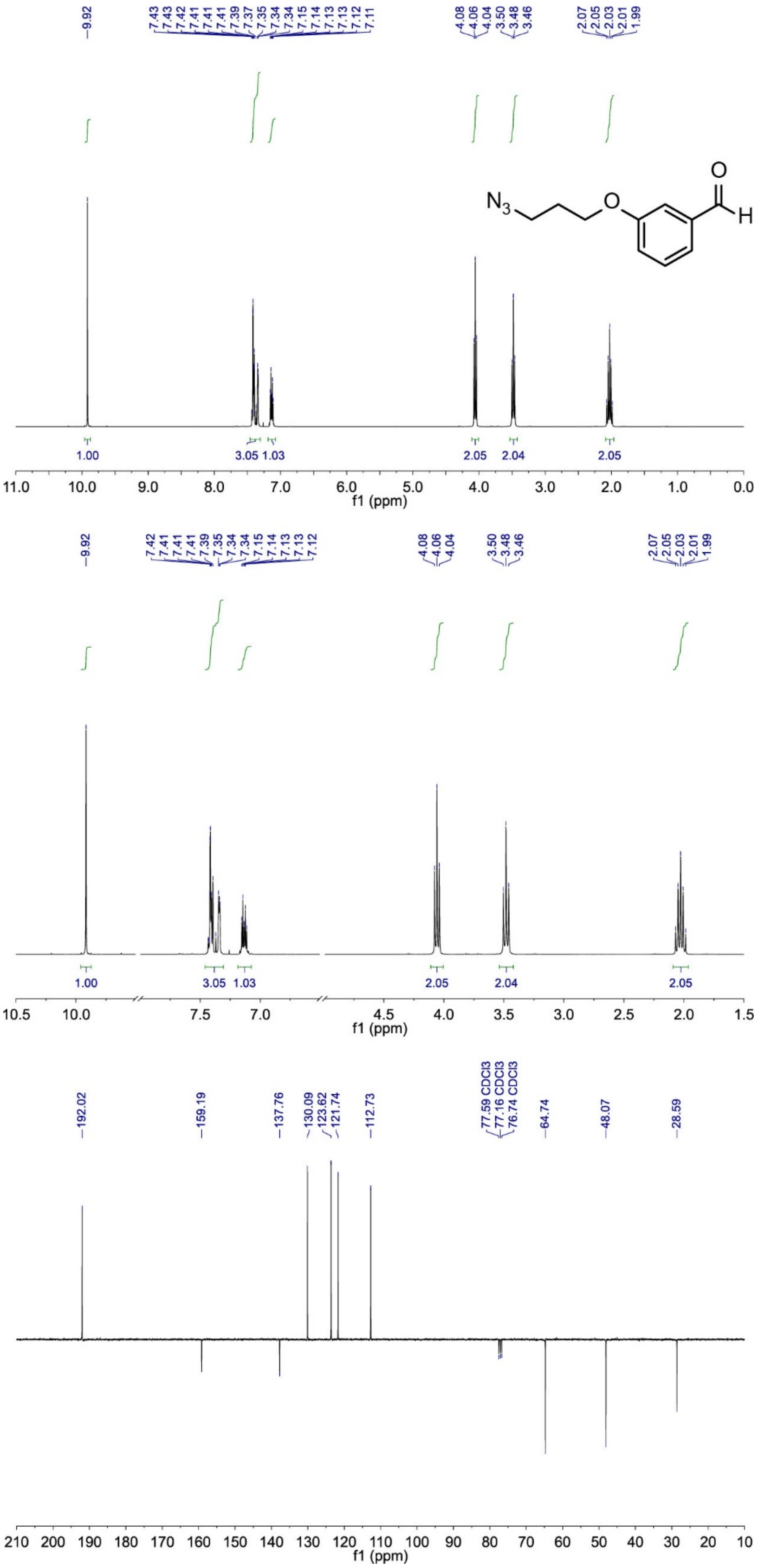

Figure S89. ^{1}H- and ^{13}C-NMR spectrum of compound **2**.

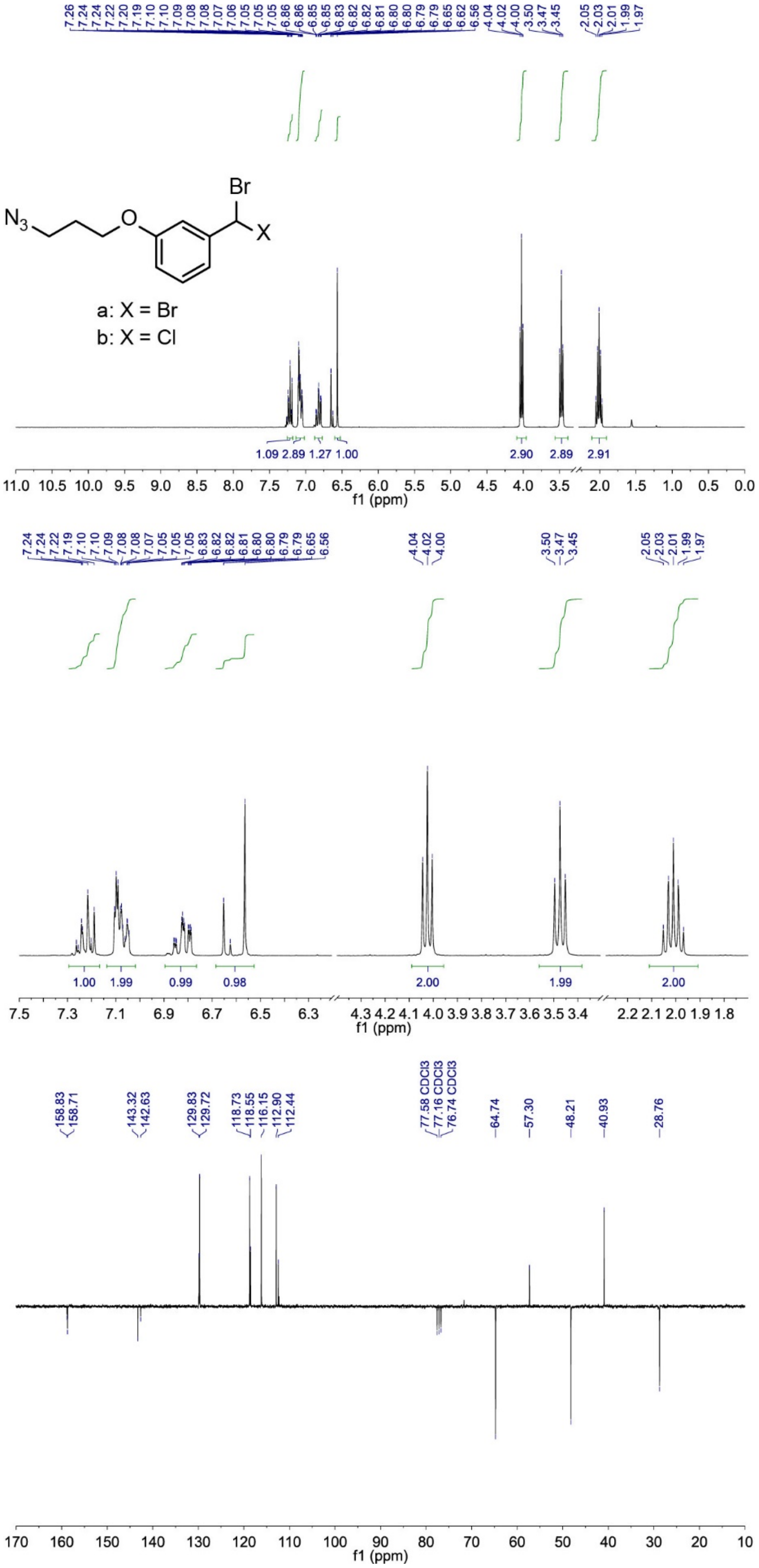

Figure S90. ^{1}H- and ^{13}C-NMR spectrum of compound **3**.

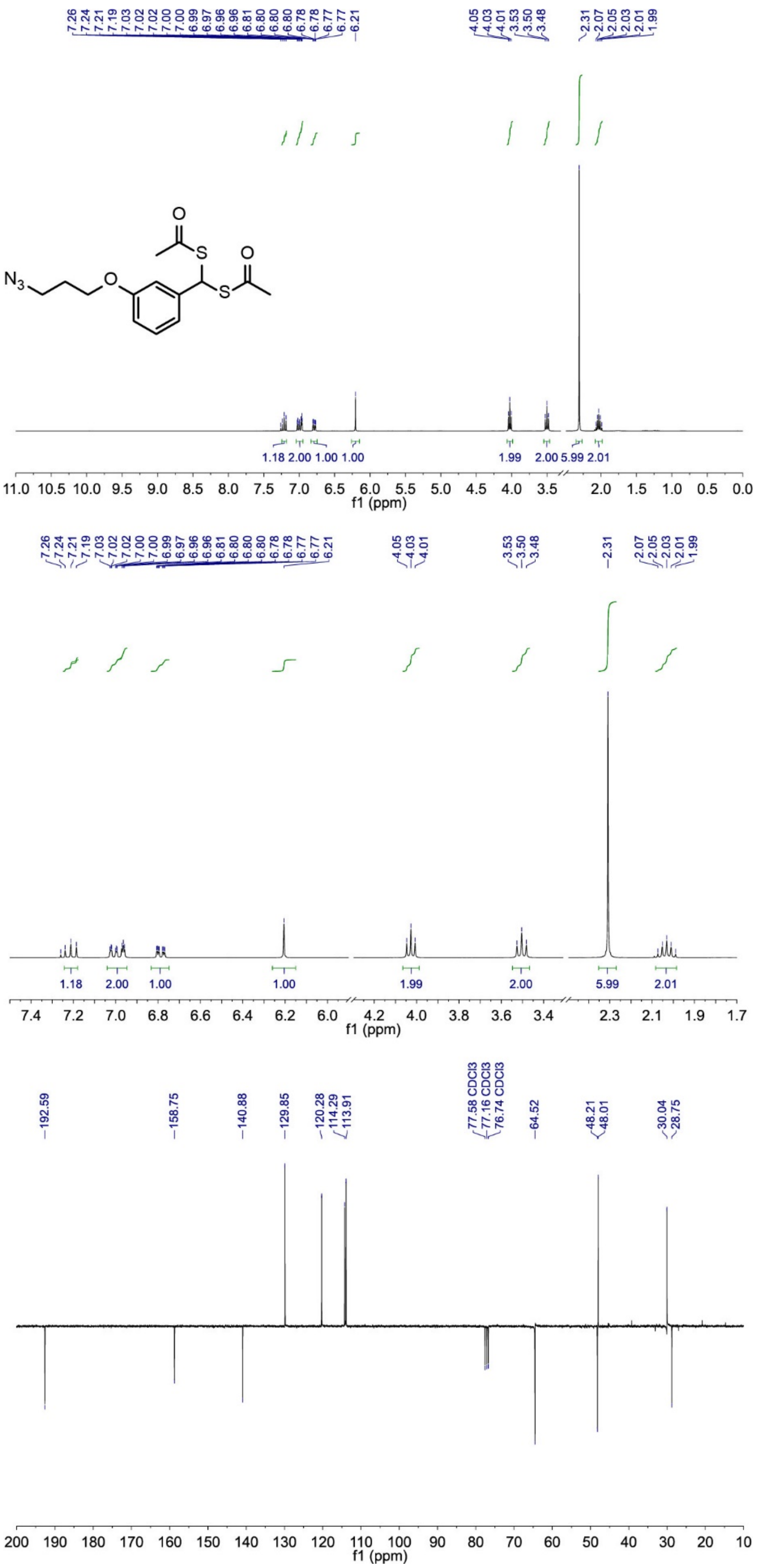

Figure S91. ^{1}H- and ^{13}C-NMR spectrum of compound **4**.

12. References

[1] Y. Zhao, J. Kang, C. M. Park, P. E. Bagdon, B. Peng, M. Xian, *Org. Lett.* **2014**, *16*, 4536–4539.

[2] H. Klinker, C. Haas, N. Harrer, P. B. Becker, F. Mueller-Planitz, *PLoS One* **2014**, *9*, e104029.

[3] J. M. Chalker, L. Lercher, N. R. Rose, C. J. Schofield, B. G. Davis, *Angew. Chem. Int. Ed.* **2012**, *51*, 1835–1839.

[4] S. M. Kelly, T. J. Jess, N. C. Price, *Biochim. Biophys. Acta* **2005**, *1751*, 119–139.
